# In Situ Construction of Imidazopyridinium Fluorescent Labels for Bioconjugation

**DOI:** 10.1002/anie.6231674

**Published:** 2026-04-09

**Authors:** Dongchen Du, László Albert, Milan Weitzel, Linda E. Eijsink, Elena R. Cotroneo, Dennis Marzin, Felipe Opazo, Nadja A. Simeth

**Affiliations:** ^1^ Institute of Organic and Biomolecular Chemistry University of Goettingen Goettingen Germany; ^2^ Cluster of Excellence “Multiscale Bioimaging: From Molecular Machines to Networks of Excitable Cells” (MBExC) University of Goettingen Göttingen Germany; ^3^ Institute of Neuro‐ and Sensory Physiology University Medical Center Goettingen Goettingen Germany; ^4^ Center For Biostructural Imaging of Neurodegeneration University Medical Center Goettingen Göttingen Germany; ^5^ NanoTag Biotechnologies GmbH Goettingen Germany

**Keywords:** bioimaging, fluorogenic labels, in situ labeling, peptide conjugation, Stokes shift

## Abstract

Simple, efficient transformations of fluorogenic nature that proceed under biocompatible conditions without the formation of byproducts are of high interest for in situ labeling and bioconjugation. Following these criteria, we describe in this work the discovery and optimization of imidazopyridinium dyes, obtained through in situ labeling of primary amines with pyridine, quinoline, and isoquinoline aldehydes. The so‐generated dyes are excited with near‐UV to violet light and emit in the orange region of the electromagnetic spectrum with Stokes shifts up to 12170 cm^−1^. We employed the reaction to obtain fluorescently labeled amino acids, lipids, and sugars; furthermore, we expanded the scope to proteins and tags for bioimaging. The robustness of the chemistry also allowed us to on‐resin staple peptides, cleanly generating fluorescent, cyclic analogs, which showcase the broad future impact of our transformation.

## Graphical abstract

Chromophore engineering of isoquinoline aldehydes generates TICT‐based fluorogenic dyes for in situ labeling of diverse (bio)molecules with large Stokes shifts compatible with cell imaging.

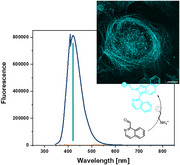

## 1 Introduction

Fluorescence‐based techniques have become indispensable for studying biological structures and processes [1, 2]. Utilizing fluorescent probes allows real‐time monitoring of cellular activities and reveals dynamic interactions between biomolecules [3, 4, 5]. Notably, the use of small, organic fluorophores of a noninvasive nature has drastically promoted advances in optical imaging and super‐resolution microscopy [6, 7].

However, only organic dyes with specific characteristics can meet the demands of fluorescence imaging, consequently, the diversity of suitable fluorescent labels is limited to well‐established fluorophores like rhodamines, fluoresceines, coumarins, cyanines, and boron dipyrromethene [8, 9]. These dyes are typically introduced on proteins through labeling of lysine or (genetically introduced) cysteine residues [10]. This often requires tedious modifications of the fluorophores and/or the biomolecules to make them compatible with the conjugation reaction [11]. Moreover, unspecific interactions can cause unwanted background signals due to the intrinsic fluorescence of the labels if the fluorophore cannot be fully washed out after conjugation [11]. Only a limited set of methods involves fluorogenic transformations [12, 13, 14, 15, 16, 17, 18], which could enhance the signal‐to‐noise ratio since a fluorescence signal will be clearly correlated with successful labeling [19]. Moreover, dyes with large Stokes shifts are favored because they minimize the overlap between excitation and emission spectra, thereby reducing background noise and improving imaging quality [20].

One approach to address these aspects is to directly construct a fluorophore on biomolecules. However, these methods mainly focus on the labeling of peptides, and the emission wavelength does not yet span the orange to red range of the electromagnetic spectrum, which would be essential to avoid competing with classical biological‐associated autofluorescence [21].

**FIGURE 1:**
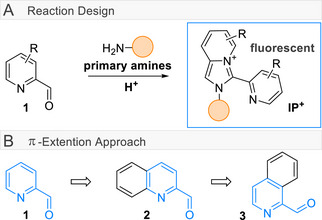
(A) Formation of imidazo[1,5‐α]pyridinium (**IP^+^**). (B) Extending the π‐system of picolinal **1** to quinoline aldehyde **2**, and isoquinoline aldehyde **3**.

Recently, several novel methods based on classical Schiff base formation have been developed for bioconjugation [22, 23, 24] and peptide stapling [25, 26]. In this context, we became interested in the chemistry of picolinal (**1**, Figure 1A), which was reported independently for its use in peptide cyclization [27], material sciences [28], and its weak fluorescence properties once reacted into imidazo[1,5‐α]pyridinium (**IP^+^**) [29]. More interestingly, a related scaffold, imidazo[1,2‐α]pyridinium, has been recently studied by Maulide et al. for fluorescence microscopy underlying the potential of this family of dyes [30].

In this work, we draw our attention to the in situ construction of fluorescent **IP^+^** labels to provide fluorogenic labeling in a simple, one‐step procedure under mild conditions from facile accessible picolinal derivatives. Moreover, the fluorogenic nature of the reaction could facilitate improved signal‐to‐noise ratios, as only labeled target molecules will show a pronounced fluorescence signal, while the use of picolinal as a core scaffold allows for tuning of the reaction and its outcome both by π‐extension and substitution (Figure 1B).

## 2 Results and Discussion

### 2.1 Selection of the Chromophore Class

We started our investigation comparing the reactivity of picolinal **1**, quinoline aldehyde **2**, and isoquinoline aldehyde **3** toward primary amines under acidic conditions. We incubated *N*_α_‐acetyl‐*L*‐lysine (200 mM) with each aldehyde (400 mM) in AcOH/H_2_O/D_2_O (2:1:1, v/v/v) for 16 h at room temperature and followed the reaction by in situ NMR spectroscopy (see Supporting Information). All three aldehydes reacted with the primary amine to form the respective **IP^+^** at a comparable rate, with quinoline aldehyde **2** clearly reacting more slowly than picolinal **1** and isoquinoline aldehyde **3**.

**FIGURE 2:**
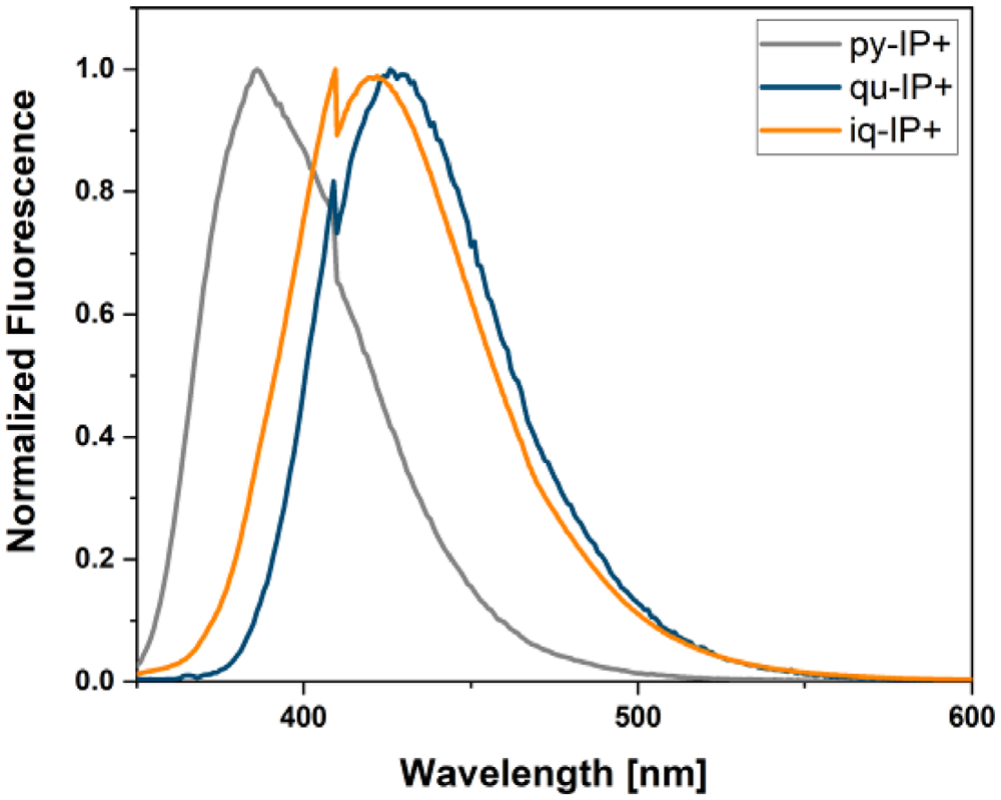
Normalized emission spectra (in MeOH) of the **IP^+^** from picolinal **1** (**py**‐**IP^+^**), quinoline aldehyde **2** (**qu**‐**IP^+^**), and isoquinoline aldehyde **3** (**iq**‐**IP^+^**) reacted with *N*_α_‐acetyl‐*L*‐lysine.

Next, we compared the emission profiles of the three obtained **IP^+^** s and compared them to each other, as well as the fluorescence of the respective starting materials. We found that both the products formed by **2** and **3** showed bathochromically shifted fluorescence emission maxima compared to **1** (Figure 2 and Table 1).

**TABLE 1:** Photophysical properties (in MeOH and PBS buffer) of different **IP^+^** s formed from isoquinoline aldehyes **3a–i** with *N*_α_‐acetyl‐*L*‐lysine.

IP^+^	*λ*_max_ ^a^ (nm)	*λ*_em_ (nm)	Stokes shift^b^ (cm^−1^)	*ε^λ^* ^max^ (10^3^*M^−1^ cm^−1^)	*Φ* (%)	*σ^λ^* ^max^ (10^3^*M^−1^ cm^−1^)
**8**	334^c^	421^c^	6187^c^	9.51^c^ 9.81^d^	8^c^ 16^d^	0.76^c^ 1.57^d^
**8a**	277^c^, 269^d^ (368^c^, 354)	607^c^ 614^d^	10699^c^ 11962^d^	18.87^c^ 23.02^d^	4^c^ 2^d^	0.75^c^ 0.46^d^
**8d**	275^c^, 268 ^d^ (364^c^, 356^d^)	602^c^ 603^d^	10861^c^ 10814^d^	17.55^c^ 20.26^d^	5^c^ 1^d^	0.73^c^ 0.20^d^
**8b**	285^c^, 262^d^ (346^c^, 378^d^)	541^c^ 540^d^	10417^c^ 7321^d^	36.77^c^ 34.55^d^	5^c^ 2^d^	1.84^c^ 0.69^d^
**8e**	282^c^, 277^d^ (365^c^, 357^d^)	540^c^ 529^d^	8879^c^ 9108^d^	40.04^c^ 37.63^d^	4^c^ 2^d^	1.16^c^ 0.75^d^
**8c**	270^c^, 263^d^ (350^c^, 379^d^)	554^c^ 569^d^	10521^c^ 8811^d^	35.35^c^ 32.37^d^	4^c^ 4^d^	1.41^c^ 1.29^d^
**8f**	267^c^, 261^d^ (360^c^, 382^d^)	548^c^ 551^d^	9530^c^ 8029^d^	36.77^c^ 39.21^d^	5^c^ 2^d^	1.84^c^ 0.78^d^
**8g**	273^c^, 270^d^ (396^c^, 367^d^)	616^c^ 663^d^	9020^c^ 12170^d^	15.31^c^ 18.41^d^	29^c^ 18^d^	5.9^c^ 7.0^d^
**8h**	269^c^, 267^d^ (393^c^, 378^d^)	622^c^ 679^d^	9373^c^ 11735^d^	20.09^c^ 20.32^d^	15^c^ 7^d^	4.0^c^ 3.0^d^
**8i**	267^c^, 266^d^ (395^c^, 373^d^)	651^c^ 662^d^	9960^c^ 11710^d^	21.16^c^ 21.71^d^	37^c^ 26^d^	8.1^c^ 8.3^d^

*Note*: For simplicity, the compound number of the respective aldehyde is given in order according to the position of the amino (piperidine or morpholine) substituent.

^a^ The lowest lying absorption maximum is given together with the wavelength of the energetically lowest shoulder, in brackets (the first number refers to MeOH, and the second to PBS (1% MeOH).

^b^ The Stokes shift was calculated based on the emission maximum and the wavelength of the energetically lowest shoulder in the UV–vis absorption spectrum around which the excitation maximum was found.

^c^ In MeOH.

^d^ In PBS:MeOH (99:1).

Due to its favorable reaction kinetics and promising emission properties, we selected **3** as the core scaffold to continue our investigation. To demonstrate the chemoselectivity of the labeling reaction to primary amines, isoquinoline **3** was incubated with other nucleophilic amino acids, including tyrosine, serine, arginine, asparagine, and methionine, resulting in no reaction (see Section S9 and Figures S50–S56). This indicates that isoquinoline aldehyde **3** is highly lysine‐specific and thus a suitable choice to undergo further chromophore engineering.

To explore whether we could shift the emission wavelength further into the red region of the electromagnetic spectrum, we introduced different electron‐donating substituents to the isoquinoline scaffold to construct an electronic push–pull system in the final chromophore, a well‐investigated strategy for lowering the HOMO–LUMO gaps of chromophores [31, 32]. As electron‐donating substituents, we selected cyclic secondary amines of different ring sizes, including morpholine, piperidine, pyrrolidine, azetidine, and methyl azetidine‐3‐carboxylate. While all substituents are expected to induce similar bathochromic shifts, the smaller rings, that is, azetidine and pyrrolidine, are reported to enhance the brightness of the fluorophores additionally [33]. Thus, we used the six‐membered rings to explore the energy profiles of the different positions and the smaller rings for further fine‐tuning.

### 2.2 Synthesis

**SCHEME 1:**
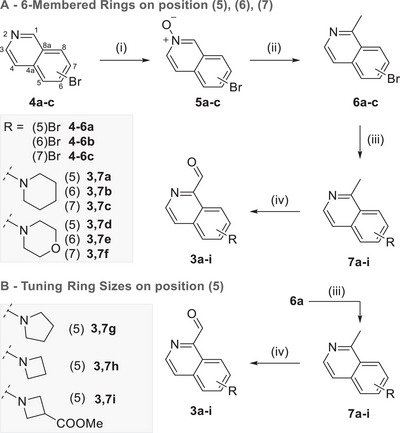
Synthesis of substituted isoquinolines **3a–f**. (i) *m*CPBA, CH_2_Cl_2_, 0°C to 25°C, 13 h (90%–97%); (ii) PPh_3_MeI, *t*BuOK, THF, 65°C, 12 h, (70%–83%); (iii) Pd_2_dba_3_, BINAP, secondary amines, Cs_2_CO_3_, toluene, 80°C, 16 h, (70%–88%); (iv) SeO_2_, dioxane, 80°C, 4 h, (53%–87%).

To synthesize the corresponding aldehydes, we started from commercially available isoquinolines bearing a bromo‐substituent at different positions (Scheme 1). The compounds were transferred into their respective *N*‐oxides, following known procedures [34] (90%–97% yield) and subsequently selectively methylated at position 1 using PPh_3_MeI (70%–83% yield). Then, the bromo substituent was replaced with the elected secondary amine in a Buchwald–Hartwig cross‐coupling reaction, affording the target compounds **7a–i** in 66%–92% yield, respectively. Finally, the methylisoquinolines were oxidized with SeO_2_ providing aldehydes **3a–i** in 53%–87% yield, respectively.

### 2.3 Photophysical Properties

Next, we explored the differently substituted aldehydes **3a–f** as labeling reagents with *N*_α_‐acetyl‐*L*‐lysine. Then, the isolated products (*cf*. Figure 3) were studied regarding their photophysical properties in MeOH (Table 1). The compounds have first, clear electronic transition maximum at 267–285 nm, which was used for determining the molar absorption coefficient, but trails with two shoulders (around 320 and 360 nm) up to 430 nm into the visible light range of the spectrum. Scanning the excitation profile revealed that the shoulder range can be used to efficiently excite the dyes (see Section S4.2 and Figures S24–S44) and was thus used to calculate the Stokes shifts of the molecules. The in situ formed labels showed good emission properties (spectra are displayed in Figures S24–S44, and an example is shown in Figure 3).

**FIGURE 3:**
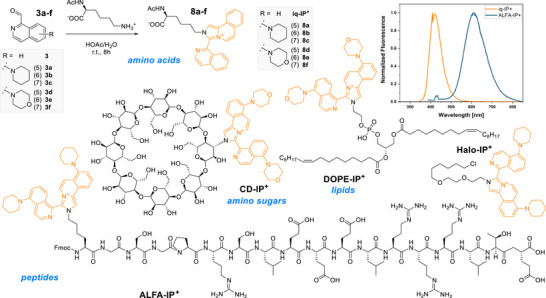
Overview of the different labeling products investigated in this study with the fluorophore highlighted in orange. The insert shows the normalized emission spectra of **iq‐IP^+^** and **ALFA‐IP^+^** (10 µM in PBS, 1% MeOH) showing the impact of the electron‐donating substituents.

Strikingly, introducing morpholine and piperidine substituents in positions 5, 6, and 7 of the isoquinoline core influenced the UV–vis absorption properties only slightly but shifted the emission maxima from 421 nm (**3**, 6187 nm Stokes shift) to 540–607 nm with a Stokes shift of up to 11962 cm^−1^ when employing isoquinoline aldehyde **3a** as labeling agent. By investigating the same compounds in PBS buffer (1% MeOH), we found that the photophysical properties of the molecules were hardly affected (*cf*. Table 1).

Next, we determined the fluorescence quantum yield (QY) *Φ* in both MeOH and PBS buffer (1% MeOH). We found that the emission QYs were in the range of 1%–16% depending on the scaffold and are mostly in the range of reported molecules [17]. Interestingly, unsubstituted **8** showed the highest QYs of 8% in pure MeOH and 16% in PBS buffer (1% MeOH), while the compounds substituted with six‐membered nitrogen‐based heterocycles showed low single digit values. Both the large Stokes shift and the low QYs can be explained when considering the substituted **IP^+^** chromophore as a twisted intramolecular charge transfer (TICT) dye [35, 36]. Here, the imidazopyridinium cation would act as an electron acceptor, while amino substituents function as electron donors. Upon excitation, the electron donor and acceptor groups twist relative to each other, creating a highly polar charge‐separated state that can affect the fluorescence. The TICT effect is most pronounced in **IP^+^** s with piperidine and morpholine in position 5, giving them the largest Stokes shift, though compromising their QYs.

### 2.4 Biomolecular Labeling

To explore the practical relevance of our transformation, we moved our attention to the scope of the reaction and explored different synthetic transformations with relevance in biological imaging. Thus, we explored whether we could also observe the formation of **IP^+^** with different classes of biomacromolecules and subjected the amino‐group‐containing lipid 1,2‐di‐(9*Z*‐octadecenoyl)‐sn‐glycero‐3‐phosphoethanolamin (DOPE) and amino‐α‐cyclodextrin to the labeling reaction. While the aromatic amine in adenosine did not allow the full condensation of the dye (MS analysis showed a +18 signal), we found that all aliphatic, primary amines could form the corresponding **IP^+^** showing comparable emission spectra as the model compounds (Figures 3 and S24–S44; Section S4.2). These results underline the broad applicability toward biomolecule labeling and the high selectivity of the reaction toward primary amines.

To also develop potential bioimaging agents, we generated both an **IP^+^**‐derivative of the Halo‐tag ligand and modified the ALFA epitope tag (Figure 3). The latter is comprised of a 14‐amino acid peptide sequence with high helical character, biologically compatible, hydrophilic, and with no net charge at physiological pH [37]. The peptide was synthesized by microwave‐assisted solid‐phase peptide synthesis (MA‐SPPS) and decorated with a short GSG linker on its *N*‐terminus. There, a lysine residue was coupled, selectively deprotected, and subsequently reacted with isoquinoline aldehyde **3a** on‐resin. After labeling, the fluorescent peptide was obtained directly after cleavage from the resin and purification via RP‐HPLC.

The fluorescence profiles did not deviate significantly from those of the nonconjugated *N*_α_‐acetyl‐*L*‐lysine (*cf*. Section S4.2 and Figures S38 and S44), indicating that our method provides an easy way to form charged fluorescent peptides, as well as a high predictability of the in situ‐formed fluorophores.

**FIGURE 4:**
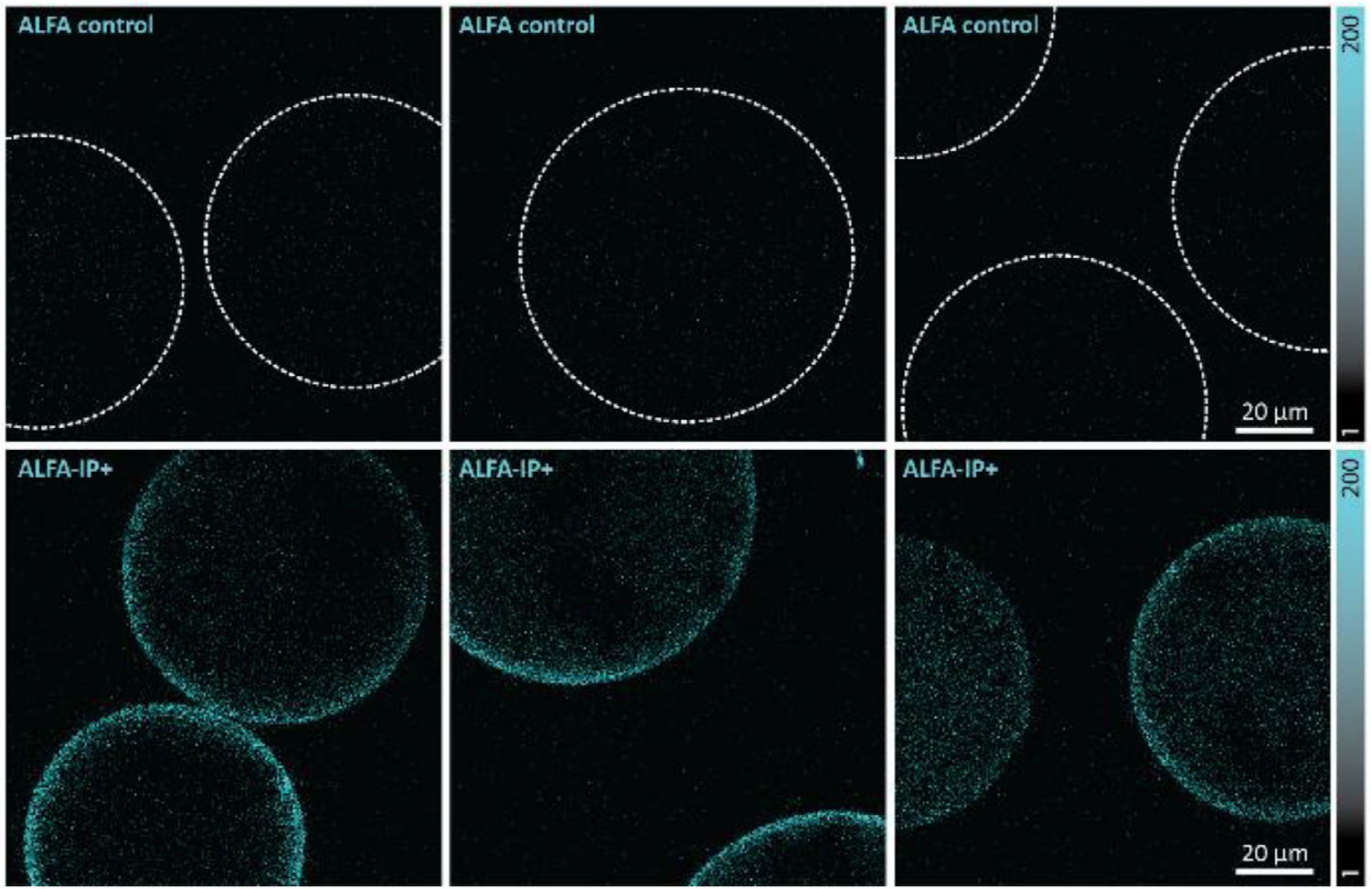
Scanning confocal microscopy of immobilized **ALFA‐IP^+^**. Exemplary images of magnetic agarose beads (ALFA Selector) loaded with unconjugated ALFA peptide or ALFA‐IP^+^ and imaged under confocal microscopy. The yellow dotted lines in the top panel denote bead positions when loaded with nonfluorescent ALFA peptide. All images were scaled to arbitrary units between 1 and 200, as indicated in the look‐up table at the right, to allow a direct visual comparison. The scale bar represents 20 µm.

The so‐obtained **ALFA‐IP^+^** was then used to explore whether our initial set of dyes could be visualized through confocal microscopy. For this, we employed magnetic beads carrying a nanobody anti‐ALFA‐tag [37] and compared images with unlabeled and labeled ALFA peptides (Figure 4). We could clearly see a distinct fluorescent signal, demonstrating that our **IP^+^** dyes showed their potential for bioimaging.

While the above‐mentioned labeling reactions underline the synthetic applicability of our reaction toward biomolecules, we wanted to further explore its practicality in the direct labeling of proteins. As lysine side chains are typically protonated in cellular environments, introducing a charged label might be beneficial [38, 39]. Thus, we tested the labeling of lysozyme (LYZ) and bovine serum albumin (BSA) with aldehyde **3b** in acetate buffer pH = 4 and pH = 5 (with 20% MeCN as cosolvent) and in a mixture of water/AcOH/MeCN (9:1:10, v/v/v) at 30°C for 18 h (*cf*. Section S7 and Figure S48). Gel electrophoresis revealed fluorescently labeled proteins under the latter conditions, while labeling in buffer was ineffective, likely limited by the low water solubility of the aldehydes.

Our experiments showed that the initial set of **IP^+^** dyes **3a–f** have the potential to label biomolecules and to generate fluorescent tags that can be visualized through confocal microscopy.

However, we also identified two key properties that needed to be optimized: on the one hand, the low fluorescence signal of the labeling products would make it challenging to perform imaging in a cellular environment. On the other hand, the addition of organic cosolvents was required to realize sufficient solubility for protein labeling, conditions that might pose issues for future biological‐based applications.

### 2.5 Fine Tuning of Dye Properties for Optical Imaging

#### 2.5.1 Brightness

To further fine‐tune the properties of our labeling reagents toward imaging applications, we first investigated the impact of the ring size of the electron‐donating substituent on the fluorescence QY in **IP^+^**. We elected position 5 of the isoquinoline core as an attachment point, as **3a** and **3b** showed the largest Stokes Shifts indicating a pronounced TICT, which is expected to be beneficial for the optical contrast the fluorogenic reaction will generate.

Thus, pyrrolidine, azetidine, and methyl azetidine‐3‐carboxylate were attached to the brominated isoquinoline **6a**, analogously to the six‐membered rings (Scheme 1). As expected, the absorption properties of the new **IP^+^** dyes **8g–i** are similar to **8a** and **8d** (Table 1). In contrast, the position of the respective *λ*_em_ deviates from the larger ring analogues by 59–76 nm, now lying well beyond 600 nm. Moreover, the fluorescence QY *Φ* increased from morpholine <piperidine <azetidine <pyrrolidine, with the latter having 18 times higher QY in buffer than **IP^+^** with a morpholine substituent at the same position. This trend agrees with substituent effects observed in quinolinium‐functionalized cyanine dyes [41] but stands in slight contrast to trends in rhodamine ones, where the four‐membered rings are generally more beneficial [33]. However, introducing a carboxylic ester group as a handle for further functionalization onto azetidine was a way to enhance QY [42]. Indeed, **8i** had a QY of 26% in PBS buffer and thus showed a pronounced fluorescence‐ON signal upon in situ construction of the **IP^+^** fluorophore. Varying pH values of the buffer left the emission spectrum of **8i** largely unaffected, only showing minor quenching above pH 10 (Section S4 and Figures S45 and S46).

**FIGURE 5:**
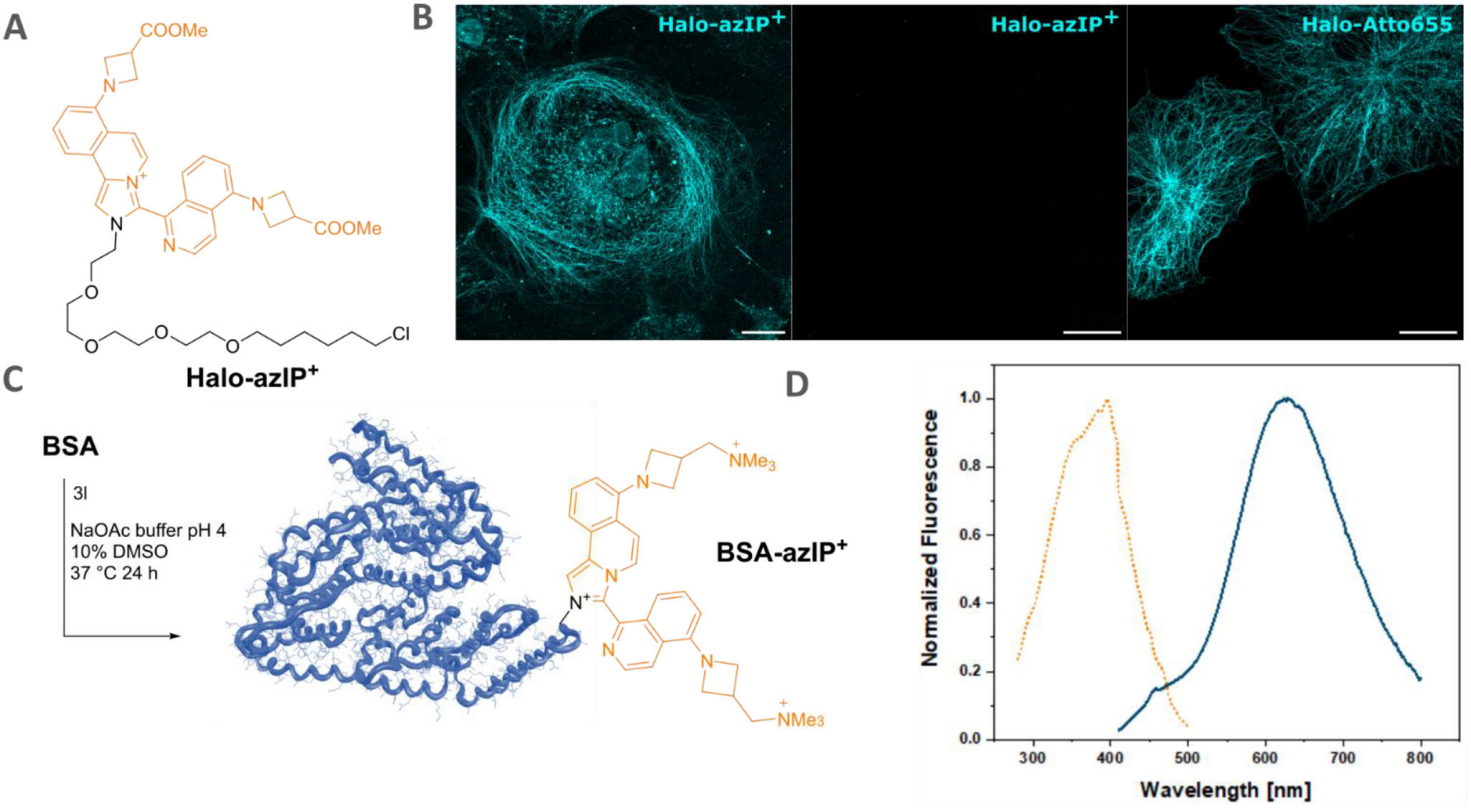
(A) Chemical structure of **Halo‐azIP^+^**. (B) Confocal laser‐scanning images obtained using **Halo‐azIP^+^**. The images show α‐tubulin staining visualized with HaloTag‐conjugated nanobodies and **Halo‐azIP^+^** (ligand), a negative control using only **Halo‐azIP^+^**, and a positive control using the same staining complex with Halo ligand conjugated to ATTO655 (Halo‐ATTO655), respectively. **Halo‐azIP^+^** and negative‐control images were acquired using the same microscopy settings. The negative control uses a fivefold higher concentration (300 nM) of **Halo‐azIP^+^** to increase stringency. Scale bars represent 20 µm. (C) Representation of the labeling reaction on bovine serum albumin (BSA, protein structure from pdb 3V03 [40]) using compound **3l** in sodium acetate buffer (0.1 M, 10% DMSO, 24 h, 37°C). (D) UV–vis absorption (orange dotted line) and emission spectrum (blue solid line) of **BSA‐azIP^+^** in PBS buffer (pH = 7.4).

We next used the optimized labeling agent **3i** to synthesize the fluorescent HaloTag ligand **Halo‐azIP^+^** (Figure 5A; for details see Section S5). The compound showed comparable photophysical properties to **8i** (Table 1 and Figures S42 and S43; Section S4) and was thus employed for staining *cells* using a secondary nanobody (2.NbHT) fused to HaloTag. The so‐obtained 2.NbHT‐IP^+^ was then used for confocal microscopy to reveal primary antibodies (1.Abs) binding alpha‐tubulin in COS‐7 cells (Figure 5B). Halo ligand functionalized with Atto655 was used as a positive control. Incubating the cells with **Halo‐azIP^+^** in the absence of 1.Ab and 2.NbHT, as a negative control, confirmed the specificity of the **Halo‐azIP^+^**. This result highlights the potential of the developed dyes for advanced protein labeling applications, including precise imaging of sub‐cellular structures.

#### 2.5.2 Water‐Solubility

**SCHEME 2:**
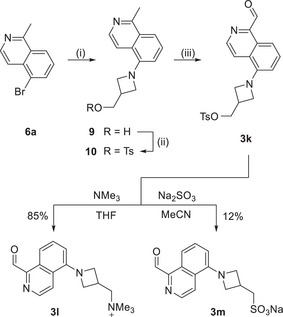
Synthesis of water‐soluble isoquinolines **3l** and **3m**. (i) Pd_2_dba_3_, BINAP, 3‐azetidinemethanol, Cs_2_CO_3_, dioxane, 90°C, 24 h, 71%; (ii) TsCl, CH_2_Cl_2_, 0°C to r.t., 12 h, 58%). (iii) SeO_2_, dioxane, 90°C, 3 h, 51%.

Next, we thus aimed to synthesize water‐soluble isoquinoline aldehyde derivatives (Scheme 2 and Section S5) to reduce the amount of organic cosolvent required for protein labeling. Both a cationic (**3l**) and an anionic (**3m**) labeling agent could be prepared. Both water‐soluble labeling reagents were reacted with protected *N*_α_‐acetyl‐*L*‐lysine to result in the corresponding **IP^+^** derivates **8l** and **8m**. Analyzing the characteristics using UV–vis absorption and fluorescence spectroscopy, we found absorption and emission profiles comparable to those of **8** **h**. However, the compound differed significantly in the fluorescence QY (*cf*. Table 2). While **8l** exhibited a Stokes shift of 10906 cm^−1^ and a QY of 5%, **8m** showed a Stokes shift of 8695 cm^−1^ with a QY of only 1% in PBS buffer. These results were counterintuitive, as sulfonates are frequently used to increase the aqueous compatibility of fluorescence dyes. However, considering that our dyes work based on a TICT mechanism, charged functional groups might cause different effects than in classical fluorophores.

**TABLE 2:** Photophysical properties (in MeOH and PBS buffer) of water soluble **IP^+^** dyes **8l** and **8m**.

IP^+^	*λ*_max_ ^a^ (nm)	*λ*_em_ (nm)	Stokes Shift^b^ (cm^−1^)	*ε^λ^* ^max^ (10^3^*M^−1^ cm^−1^)	*Φ* (%)	*σ^λ^* ^max^ (10^3^*M^−1^ cm^−1^)
**8l**	266^c^, 265^d^ (382^c^, 379^d^)	623^c^ 646^d^	10128^c^ 10906^d^	20.95^c^ 19.15^d^	18^c^ 5^d^	8.0^c^ 7.3^d^
**8m**	266^c^, 266^d^ (395^c^, 393^d^)	550^c^ 597^d^	7135^c^ 8695^d^	20.15^c^ 18.85^d^	3^c^ 1^d^	2.4^c^ 0.7^d^

^a^ The lowest lying absorption maximum is given together with the wavelength of the energetically lowest shoulder, in brackets (the first number refers to MeOH, and the second to PBS (1% MeOH).

^b^ The Stokes shift was calculated based on the emission maximum and the wavelength of the energetically lowest shoulder in the UV–vis absorption spectrum around which the excitation maximum was found.

^c^ In MeOH.

^d^ In PBS:MeOH (99:1).

Based on the photophysical characterization, we elected **3l** as labeling agent to test protein functionalization. We incubated a large excess of the aldehyde (10 mM) together with BSA (3 mg/mL, ∼45 µM) in 0.1 M sodium acetate buffer containing 10% DMSO for 24 h at 37°C. Dialysis into PBS buffer (pH 7.4) allowed us to separate the fluorogenically labeled protein from the excess labeling agent. We then recorded the fluorescence emission of the labeled protein and determined the fluorescence QY. The fluorescence spectrum is displayed in Figure 5D and is almost identical to the one of **8l**, underscoring that the same **IP^+^** dye was indeed constructed in situ on the protein as it was on the model compounds. A QY of 3.5% was determined, which is slightly lower than the one determined for **8I** in PBS of 5%. This difference may be explained by self‐quenching due to the proximity of fluorophores on the protein surface.

### 2.6 On‐Resin Construction of Fluorescent Peptides

**FIGURE 6:**
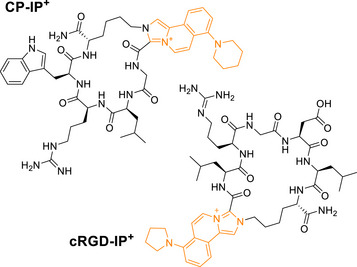
Chemical structures of the on‐resin cyclized peptides **CP‐IP^+^** and **cRGD‐IP^+^**.

Imine conjugations, followed by the irreversible formation of heterocycles, can be used not only for labeling chemistry but have also been exploited for the generation of peptide staples and macrocycles [27, 43]. Naturally, we were curious to see whether our fluorogenic **IP^+^** formation could be applied in this context and, in this manner, directly generate fluorescent peptide conjugates, which could make the need for additional, post‐synthetic labeling dispensable. We thus synthesized the linear peptide H‐SGLRWK‐NH_2_ via MA‐SPPS. Then, the terminal, unprotected serine was oxidized using NaIO_4_, adapting known protocols [44], and the Mmt‐protected lysine was selectively deprotected to form the macrocyclic peptide **CP‐IP^+^** on‐resin (Figure 6). After cleavage from the resin, the peptide was obtained in high purity after HPLC‐purification. The cyclized peptide retained the fluorescence from the **IP^+^** unit. To further validate the feasibility of this on‐resin **IP^+^** formation, we also synthesized a fluorescent cyclic RGD peptide (**cRGD‐IP^+^**) containing the RGD motif, which is responsible for cell adhesion to the extracellular matrix [45]. The relatively weak emission of the cyclic conjugates likely arises from the TICT nature of the **IP^+^** scaffold in proximity to a carbonyl acceptor. Interestingly, this sensitivity to local polarity and conformation may render such conjugates responsive to environmental changes, opening opportunities for designing conformational or interaction‐sensitive fluorescent peptides in future applications.

## 3 Conclusion

In summary, we have discovered an efficient fluorogenic labeling reaction involving isoquinoline aldehydes. The in situ formation of **IP^+^** dyes is specific toward primary amines and can be applied to a wide range of biomolecules, including peptides, sugars, and lipids. By altering the ring size of the donors, the emission maxima can be shifted far beyond 600 nm, and fluorescence QYs of nearly 30% in buffer can be achieved. These properties make **IP^+^** dyes, attached to small epitopes (**ALFA‐IP^+^**) or protein tags (**Halo‐azIP^+^**), compatible with conventional confocal microscopy and cell imaging. While introducing solubilizing groups facilitated direct protein labeling in buffer, more lipophilic analogs could be engineered into reagents for the synthesis of unnatural peptides. Indeed, two exemplary fluorescent cyclic peptides bearing an IP^+^ linker could be directly constructed on‐resin, indicating the potential of our protocol for fluorogenic peptide macrocyclization applications. Our reaction is thus readily applicable in a wide variety of on‐target syntheses of fluorophores, is biocompatible, and allows for optical imaging.

## Conflicts of Interest

D.D. and N.A.S. are inventors on a patent application on the synthesis of fluorogenic reagents and the resulting fluorophores (EP26159845.2). F.O. is a shareholders of NanoTag Biotechnologies GmbH. All other authors declare no competing interest.

## Supporting information




**Supporting File 1**: The authors have cited additional references within the Supporting Information [46, 47, 48, 49, 50, 51].

## Data Availability

The data that supports the findings of this study are available in the Supporting Information of this article.
