# Supplementary material for: In Situ Construction of Imidazopyridinium Fluorescent Labels for Bioconjugation
Source: Angew Chem Int Ed Engl. 2026 Apr 9;65(21):e6231674. doi: 10.1002/anie.6231674 (PMC13182221; doi:10.1002/anie.6231674)
Supplement: Supplementary file 1 — Supporting File 1: The authors have cited additional references within the Supporting Information [46, 47, 48, 49, 50, 51]. [file ANIE-65-e6231674-s001.pdf]

Supporting Information

FOR

In Situ Construction of Imidazopyridinium Fluorescent Labels for Bioconjugation

Dongchen Du,[a,b] László Albert,[c,d],‡ Milan Weitzel,[a],‡ Linda E. Eijsink,[a,b] Elena R. Cotroneo,[a] Dennis Marzin,[a] Felipe Opazo,[c,d,e] and Nadja A. Simeth*[a,b]

[a] Dongchen Du, Milan Weitzel, Dr. Linda Eijsink, Dr. Elena R. Cotroneo, Dennis Marzin, Prof. Dr. Nadja A. Simeth
Institute of Organic and Biomolecular Chemistry
University of Goettingen, Goettingen, Germany.
E-mail: nadja.simeth@uni-goettingen.de

[b] Dongchen Du, Dr. Linda Eijsink, Prof. Dr. Nadja A. Simeth
Cluster of Excellence "Multiscale Bioimaging: from Molecular Machines to Networks of Excitable Cells" (MBExC)
University of Goettingen, Germany.

[c] Dr. László Albert, Prof. Dr. Felipe Opazo
Institute of Neuro- and Sensory Physiology
University Medical Center Goettingen, Goettingen, Germany.

[d] Dr. László Albert, Prof. Dr. Felipe Opazo
Center for Biostructural Imaging of Neurodegeneration,
University Medical Center Goettingen, Göttingen, Germany.

[e] Prof. Dr. Felipe Opazo
NanoTag Biotechnologies GmbH, Goettingen, Germany,

‡These authors contributed equally.

Table of Contents

1. Author Contributions ... 3

2. General Information ... 3

3. NMR Kinetic Experiments ... 7

4. Photophysical Properties of IP⁺s ... 11

4.1. UV-Vis Absorption Spectroscopy ... **11**

4.1.1. MeOH ... 11

4.1.2. PBS (1% MeOH) ... 14

4.2. Fluorescence Spectroscopy ... **16**

4.2.1. MeOH ... 16

4.2.2. PBS:MeOH (99:1) ... 22

4.2.3. PBS:MeOH (1:1) ... 26

4.2.4. pH Dependency ... 27

5. Chemical Synthesis ... **29**

5.1. General Procedures ... **29**

5.2. Compound Characterization ... **38**

6. Peptide Synthesis and Labelling ... 59

7. Protein Labelling ... 64

8. Bioimaging ... 68

9. LCMS Analysis for Chemoselectivity ... 70

10. Small Molecular Analysis: NMR spectra ... 74

11. Peptide Analysis: HPLC Traces and MS Spectra ... 109

12. References ... 115

1. Author Contributions

DD and NAS conceptualized the project. DD synthesized and characterized all small molecules. ERC and DM synthesized the peptides. DD, ERC, and DM performed labelling reactions and small molecules and peptides, performed HPLC, and characterized the products. LEE and DD performed UV-Vis absorption and emission spectroscopy and data analysis together with NAS. LEE and MW conducted NMR experiments and data analysis. MW and LA performed labelling reactions on proteins and analysed the results. LA and FO designed and performed optical imaging experiments. NAS and FO acquired funding. All authors contributed to writing the manuscript and the Supporting Information under the lead of DD and NAS. All authors agreed on the final version of the manuscript and the Supporting Information.

2. General Information

Chemical Synthesis

Commercially available chemicals were used as received. Dry solvents were dried using activated 3 Å molecular sieves or from commercial sources. Phosphate Buffered Saline (PBS) buffer was purchased from Carl Roth (Roti-CELL 10x PBS CELLPURE, pH 7.3–7.5).

Flash column chromatography was performed using silica gel (pore size 60 Å, 400 mesh, 40-63 µm particle size) on a Biotage Isolera One using eluent gradients or by manual flash column chromatography.

Thin Layer Chromatography (TLC)

The progression of reactions was monitored by thin layer chromatography on Merck (Darmstadt, Germany) silica gel 60 F254 coated aluminium sheets. Spots were detected by fluorescence quenching at 254 nm or fluorescence at 366 nm or visualized by TLC staining solutions and heating with a heat gun. Amines were identified with ninhydrin solution (1.5 g ninhydrin and 3 mL acetic acid in 100 mL *n*butanol).

Solid-Phase Peptide Synthesis

For the synthesis of different peptide sequences, a Liberty Blue peptide synthesizer from CEM (Matthews, North Carolina, USA) was used. For the synthesis, the following protected amino acids were dissolved in DMF: Fmoc-Arg(Pbf)-OH, Fmoc-Glu(^{t}Bu)-OH, Fmoc-Gly-OH, Fmoc-Leu-OH, Fmoc-Lys(Mtt)-OH, Fmoc-Pro-OH, Fmoc-Ser(O^{t}Bu)-OH, Fmoc-Ser-OH , Fmoc-Thr(^{t}Bu)-OH, Fmoc-Trp(Boc)-OH, Fmoc-Val-OH.

Depending on the synthesis scale, different coupling conditions, different stock solutions for the amino acids, deprotection solution, activator and activator base were used. For the deprotection of the Fmoc group, 20% piperidine in DMF (*v/v*) was used. The amino acids were activated by DIC as activator and Oxyma or HOBt as activator base.

The non-preloaded resin (1.0 eq) was placed in the reaction vessel of the peptide synthesizer and was swollen for 30 min in DMF. First, the *N*-terminal Fmoc protecting group was removed by addition of piperidine (20 % in DMF, *v/v*). To achieve a complete cleavage of the Fmoc-group, the deprotection step was repeated twice. After washing of the resin with DMF (5 x 4 mL) the amino acid, DIC, and activator base were added to the reaction vessel, and the coupling reaction was performed. For the coupling of Fmoc-Arg(Pbf)-OH, specific coupling cycles were used to suppress the possible γ-lactam formation.

After the peptide synthesis was completed, the resin was transferred into a BD Syringe with PE-frit and was washed with DMF (6 x 4 mL) and DCM (6 x 4 mL). Following, the resin was dried under reduced pressure.

Table S1 – Overview of coupling and deprotection microwave parameters on the Liberty Blue Peptide Synthesizer utilized for the syntheses.

**Synthetic Step**	**Temperature [°C]**	**Power [W]**	**Time [s]**	**Δ Temperature [°C]**
Coupling step 1	75	170	15	2
Coupling step 2	90	30	225	1
Arg Coupling step 1	25	0	1500	2
Arg Coupling step 2	70	30	300	1
Deprotection step 1	75	155	15	2
Deprotection step 2	90	30	50	1

(Automatic) Flash Column Chromatography

Purification by flash chromatography was performed using Merck (Darmstadt, Germany) silica gel type 60 (particle size 40-63 µm) and a pressure of 0.1-1 bar. Silica gel was suspended in the eluent of choice and packed in an appropriately sized glass column equipped with a glass frit. Crude samples were loaded in a thin layer preabsorbed to the fivefold amount by weight of silica gel.

Automatic flash column chromatography was performed on a Biotage® Isolera One column machine with manually packed columns. The used gradients are indicated with each experiment.

NMR Spectroscopy

^{1}H and ^{13}C NMR spectra were recorded on a Bruker Avance Neo 400, Avance III HD 400, Avance III 400 or Avance III HD 300 at 25 °C. Chemical shifts (δ) are given in parts per million (ppm) relative to the residual solvent signal (for ^{1}H detection, δ = 7.26 ppm ($CDCl_3$), 1.94 (CD_3CN), 3.31 (CD_3OD), 2.50 (DMSO-d6); for ^{13}C detection, δ = 77.16 ppm ($CDCl_3$), 1.32 ppm (CD_3CN), 49.00 (CD_3OD), 39.25 (DMSO-d6). The splitting pattern of peaks is designated as followed: s (singlet), d (doublet), t (triplet), q (quartet), m (multiplet), br (broad), or combinations of these signals. ^{13}C APT NMR is indicated with a (+) for positive or upwards pointing signals with an odd number of attached protons and is indicated (-) for negative or downwards pointing signals. Coupling constants (J) are given in Hz. ^{1}H-NMR kinetic experiments were performed with NMR samples in $CH_3COOH/H_2O/D_2O$ at room temperature (~25 °C).

The NMR-spectra was evaluated with MestReNova 14.2.0-26256 from Mestrelab Research S.L.

Infrared Spectroscopy

The infrared spectra have been recorded on a spectrometer from BRUKER of the type Alpha-P ATR. Liquid samples were measured as film, and solid samples were placed as pure substances. The evaluation of the spectra was done with Opus 6.5 from BRUKER. Therefore, the spectral range from 4000 cm-1 to 400 cm-1 was recorded.

High Performance Liquid Chromatography (HPLC)

Semi-preparative reverse-phase HPLC purifications were performed with a system from JASCO (Tokyo, Japan), consisting of two pumps PU-2020Plus, a 3-line degasser DG2080-53, and a diode array detector MD-2010Plus.

Crude compounds were eluted with a linear gradient of two phases: phase A (MilliQ + 0.1 % TFA) and phase B (MeCN + 0.1 % TFA). An MN Nucleodor 100-5-C18, 250mm x 10mm, 5µm column from MACHEREY-NAGEL (Düren, Germany) was used for purification, with a flow rate of 3mL/min. UV detection was measured at 215 and 365nm wavelength.

For the preparation of the samples, they were dissolved in a 5:95 or 70:30 solution of MilliQ/MeCN, according to the elution gradient used for purification. Samples were filtered through a Chromafil filter from MACHEREY-NAGEL before injection.

Gradient A – 30 to 70% of MeCN (+0.1% TFA) in MilliQ (+0.1% TFA) in 25 min.

Gradient B – 5 to 95% of MeCN (+0.1% TFA) in MilliQ (+0.1% TFA) in 20 min.

UHPLC

Analysis of product purity and reaction monitoring was done by UHPLC using a Thermo Fisher Scientific UltiMate 3000 system with Dionex UltiMate 3000 pump, autosampler, column compartment and diode array detector.

Either 10 µL of purified products in H_2O/MeCN were injected right after semi-preparative HPLC purification, or small aliquots of purified lyophilized products were dissolved in 100 µL of H_2O + 0.1 % TFA or a mixture of H_2O/MeCN 3:1 + 0.1 % TFA, filtered, and 10 µL of this solution were injected.

A C18 RP column (ACE® ExcelTM 100-2 C18 from Advanced Chromatography Technologies Ltd., column size: 100x2.1 mm, pore size: 100 Å, particle size: 2 µM) was used with H_2O + 0.1 % TFA as solvent A and MeCN + 0.085 % TFA as solvent B, running linear gradients of 30−70 over 11 min. Flow rate was set to 0.3 mL/min and temperature to 50 °C. Detection was performed at 220 and 365 nm. Data were recorded and analyzed applying the software Chromeleon 7.

LC-MS

LC-MS was performed on an Agilent 1260 Infinity II combined with an InfinityLab LC/MSD iQ mass detector (ESI) using an InfinityLab Poroshell 120 EC-C18 column at 40 °C eluted with MeCN/H2O + 0.1% formic acid.

Lyophilization

Aqueous or dioxane solutions were frozen in liquid nitrogen and freeze-dried using a Christ Alpha 2-4 LD plus lyophilizer (Osterode in Harz, Germany) connected to a high vacuum pump. In fractions collected from the HPLC the organic content was reduced to a minimum amount with a flow of nitrogen and they were likewise freeze-dried. For small volumes (< 2 mL) a Christ RVC 2-18 CD plus vacuum centrifuge (Osterode in Harz, Germany) connected to the lyophilizer was used.

Melting Points

The melting point was determined with a Stuart® Melting Point Apparatus SMP10 from BARLOWORLD SCIENTIFIC.

ESI-Mass Spectroscopy

Electrospray ionization (ESI) mass spectra and high-resolution ESI (HR-MS) spectra were recorded at a maXis or MicroTOF spectrometer by BRUKER 42 DALTONIK GMBH (Bremen, Germany). The data were analyzed with Compass Data analysis software (version 4.0) by Bruker. The values are given in m/z ratio, along with the relative intensity of the peak.

The samples were dissolved in MilliQ, MilliQ/MeCN, or MeCN and filtered through a Chromafil filter from MACHEREY-NAGEL before analysis.

UV-Vis Absorption Spectroscopyp Absorption spectroscopy was done on a Specord S600 or Jasco V-670 in quartz cuvettes (path 1.00 cm) at a controlled temperature of 20 °C. Irradiation was performed with a home-built LED setup at a fixed distance, orthogonal to the detector light path. Molar attenuation coefficients (ε) were determined by fitting the slope of absorbance vs. concentration taken from at least three separate dilutions. The evaluation was performed with Spectragryph v1.2.15 and OriginPro 2020.

Fluorescence Spectroscopy and Quantum Yields

Fluorescence spectra were recorded using an Edinburgh Instruments FS5 Spectrofluorometer using a SC-05 cuvette holder. Emission quantum yields were determined using the instrument's integration sphere and software.

The evaluation was performed with Spectragryph v1.2.15 and OriginPro 2020.

3. NMR Kinetic Experiments

Aldehyde consumption and product formation were obtained from the respective intervals of samples measured 60 times at an approximate interval of 5 min, resulting in a total time of 15 hours. For this, 300 µL of a 0.4 M solution of N_{α}-acetyl-L-lysine in 50/50 v% acetic acid in water was mixed with 300 µL of a 0.8 M solution of the respective aldehyde (picolinal **1**, quinoline aldehyde **2**, and isoquinoline aldehyde **3**) in 50/50 v% acetic acid in D_2O. The samples were shaken and submitted for ^{1}H-NMR measurements immediately. Spectra were referenced to the C**H**$_3$COOH/D shift and integrated after phase- and baseline correction using Mestrenova. The resulting spectra and the integrals of some representative peaks over time are given below.

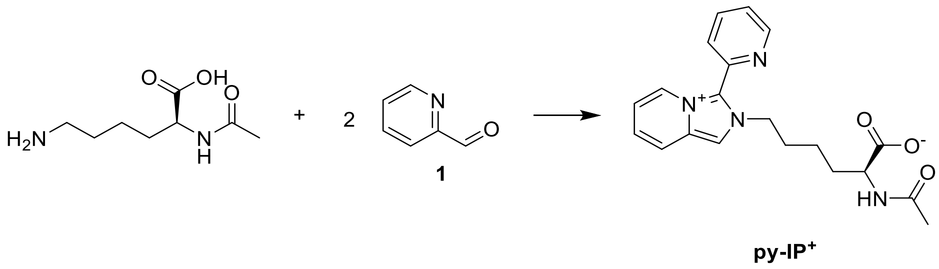

Figure S1. Chemical structures of the reaction between *N*$_{\alpha}$-acetyl-*L*-lysine and picolinal.

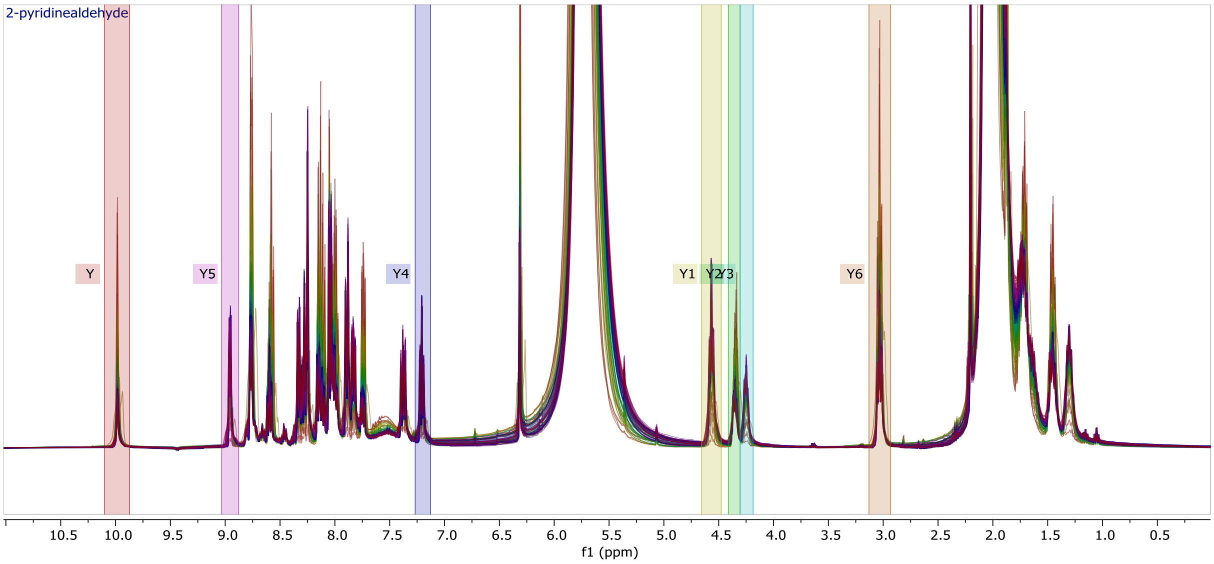

Figure S2. NMR evolution of the reaction between *N*$_{\alpha}$-acetyl-*L*-lysine and picolinal.

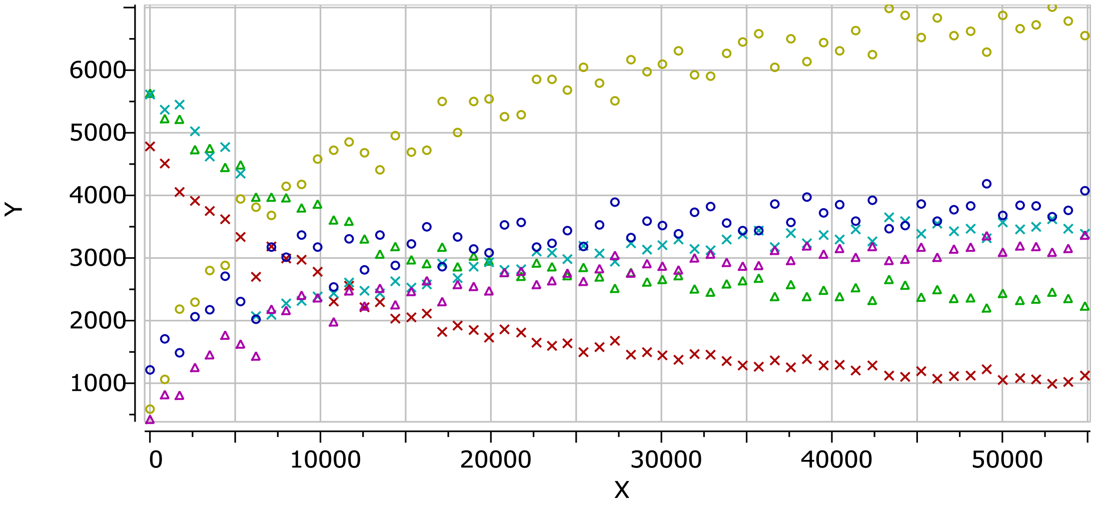

Figure S3. Traces extracted from Figure S2.

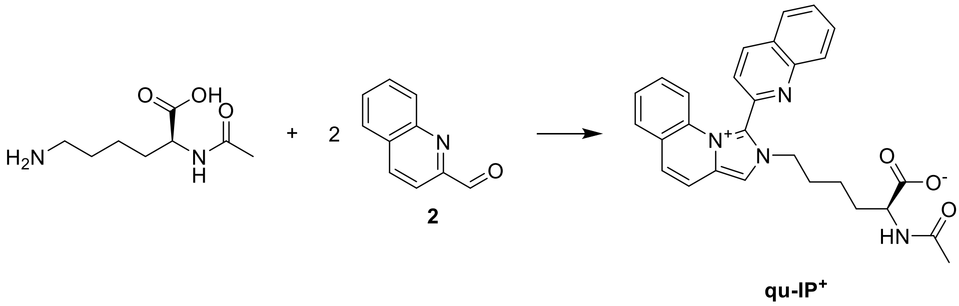

Figure S4. Chemical structures of the reaction between *N*α-acetyl-*L*-lysine and quinoline aldehyde.

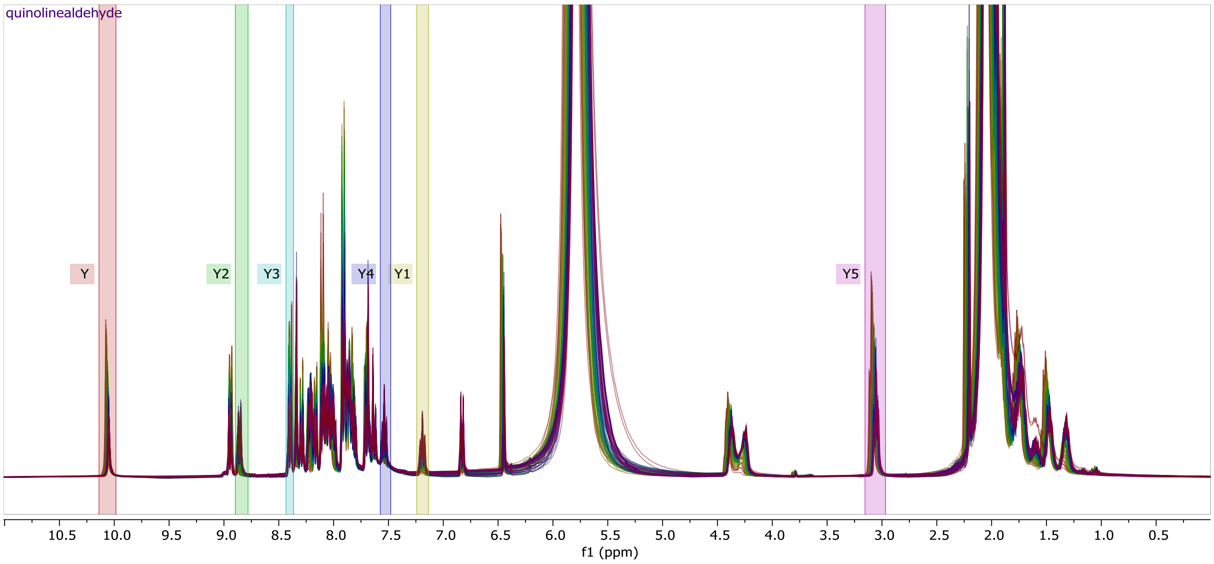

Figure S5. NMR evolution of the reaction between *N*α-acetyl-*L*-lysine and quinoline aldehyde

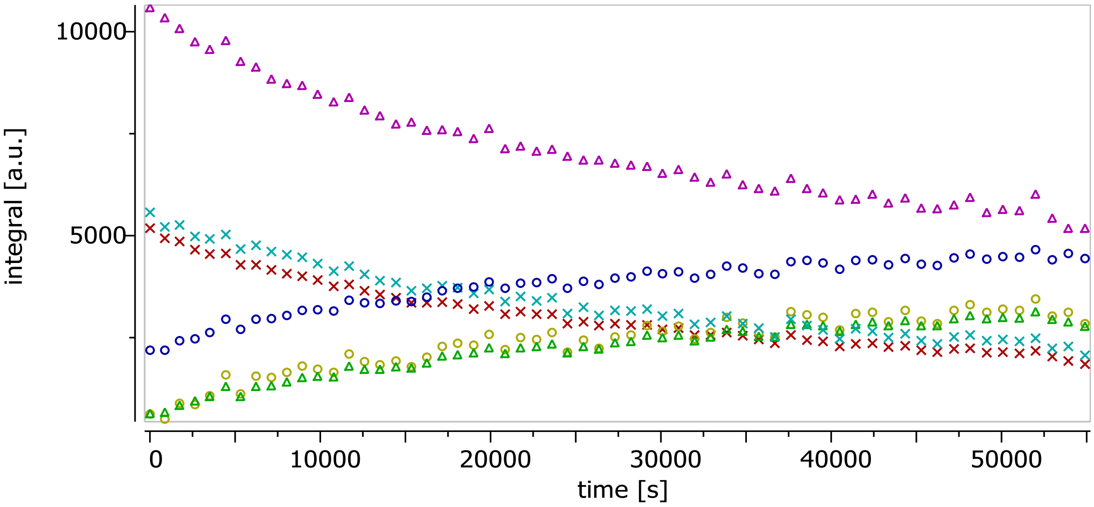

Figure S6. Traces extracted from Figure S5.

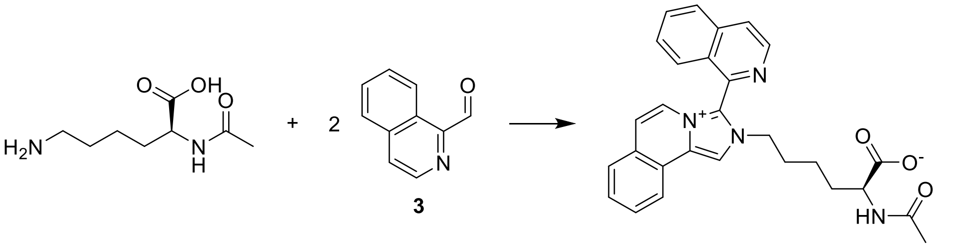

Figure S7. Chemical structures of the reaction between $N_α$-acetyl-L-lysine and isoquinoline aldehyde.

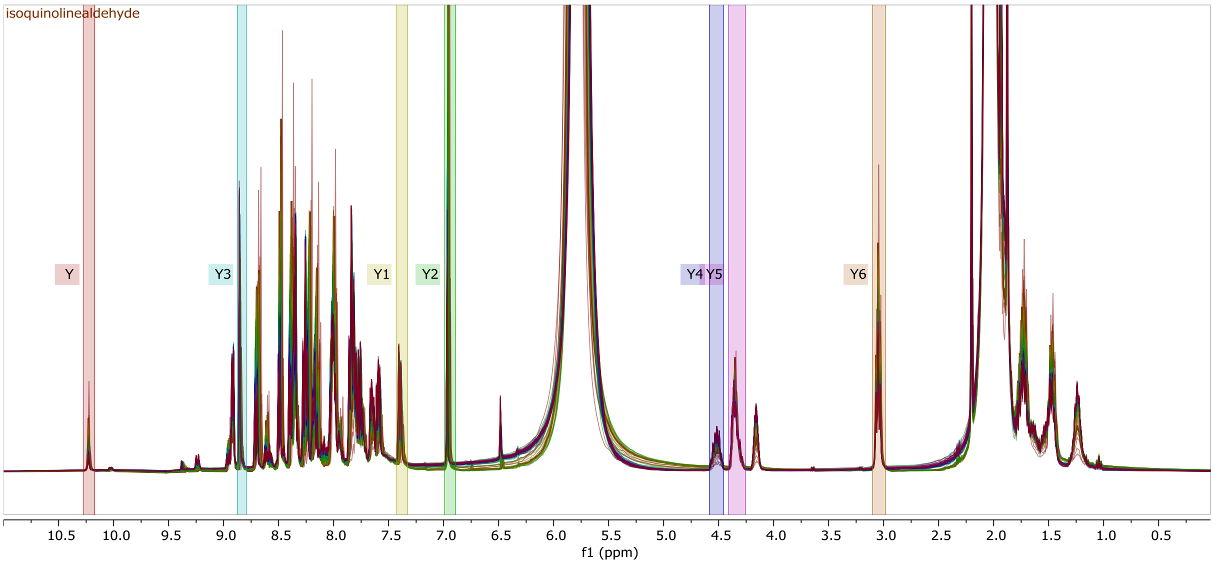

Figure S8. NMR evolution of the reaction between $N_α$-acetyl-L-lysine and isoquinoline aldehyde

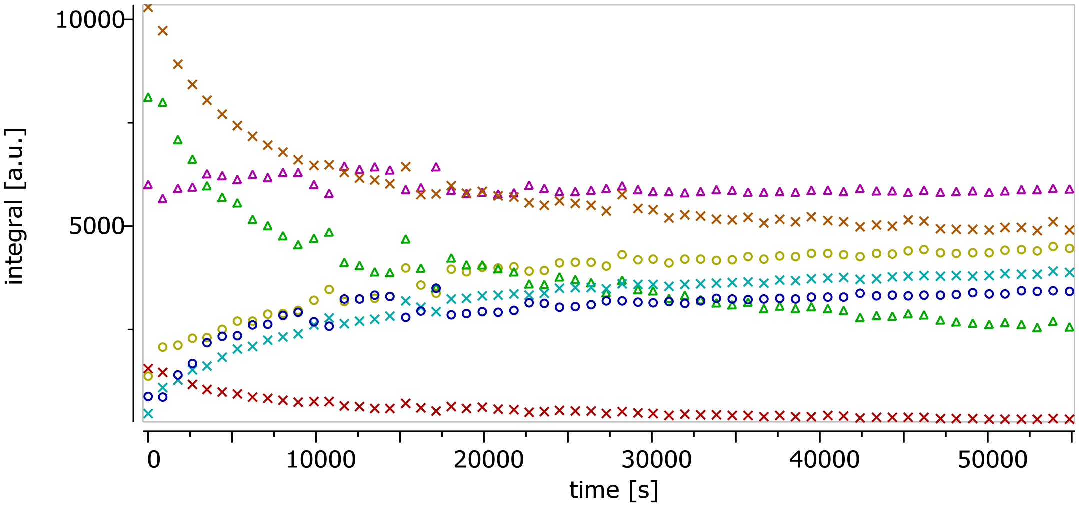

Figure S9. Traces extracted from Figure S8.

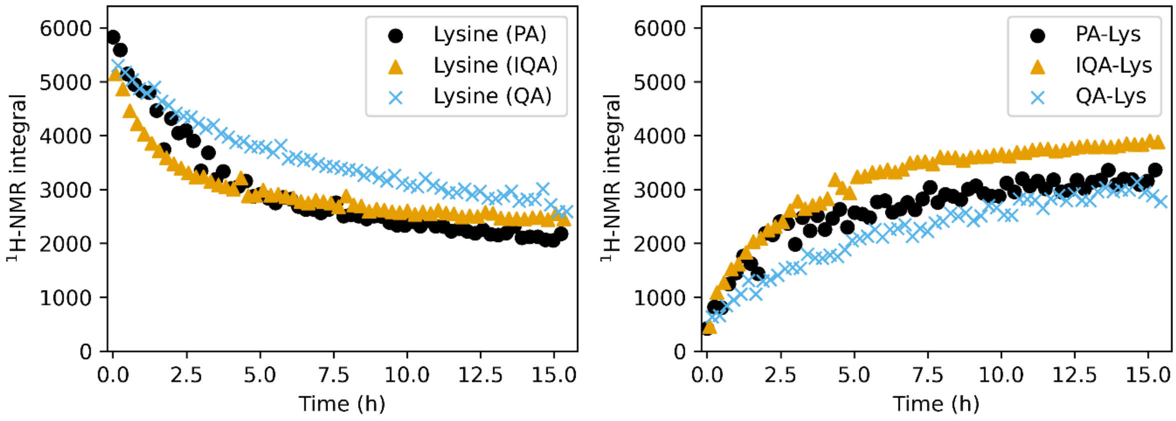

Figure S10. For rate comparisons, the integrals of the respective peaks at about 3.0 ppm belonging to the ε-CH_2 protons of *N*$_α$-acetyl-L-lysine and the respective peaks at 8.9 ppm from the 3-CH group of the newly formed imidazopyridinium ring were plotted against the time of acquisition in the graphs shown below. While rate of conversion is comparable for all three derivatives, the quinoline aldehyde 2 clearly reacts slower than the other two aldehydes.

4. Photophysical Properties of IP⁺s

4.1. UV-Vis Absorption Spectroscopy

To evaluate the UV-Vis absorption profiles of our compounds and to determine their molar absorption coefficients ε, we performed concentration-dependent measurements. For this, a stock-solution (2 mM, MeOH) of each dye was prepared and diluted to *ca*. 2 mL to eventually record UV-Vis absorption spectra at six different concentrations. All measurements were performed in triplicates, the data was processed using Spectragryph (v1.2.15), and OriginPro2020, and the molar absorption coefficients ε was determined at the lowest electronic transition maximum λ_{max}. The so-obtained spectra are displayed below for each compound in MeOH and in PBS (1% MeOH).

4.1.1. MeOH

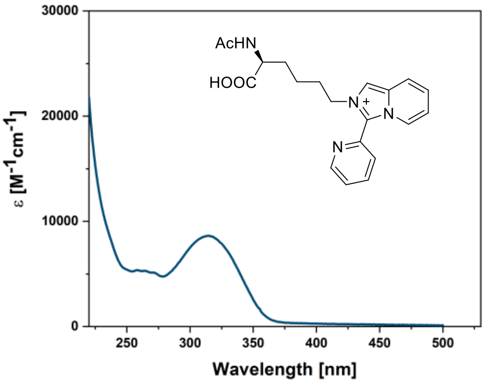

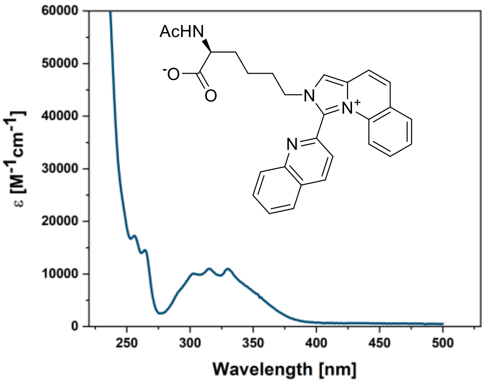

Figure S11. UV-Vis absorption spectra of py-ip⁺ and qu-ip⁺ in MeOH.

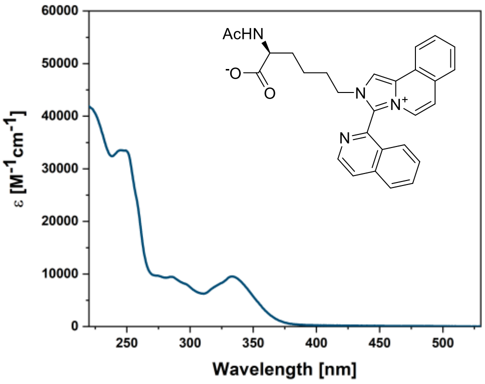

Figure S12. UV-Vis absorption spectra of 8 in MeOH.

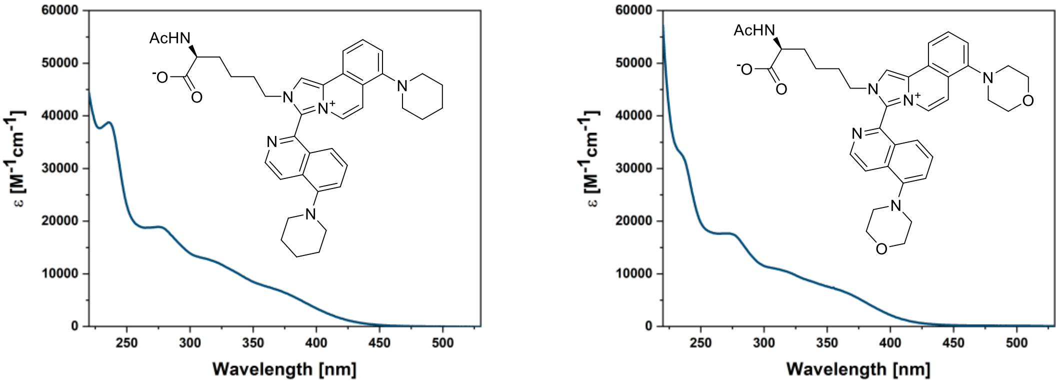

Figure S13. UV-Vis absorption spectra of 8a and 8d in MeOH.

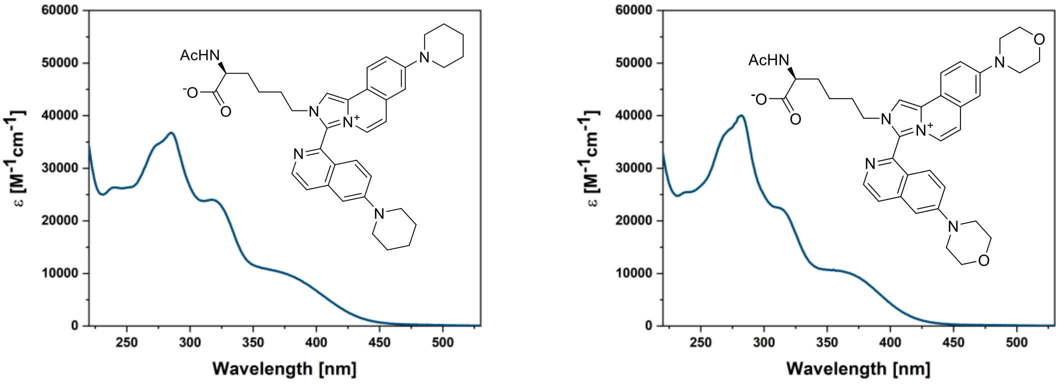

Figure S14. UV-Vis absorption spectra of 8c and 8d in MeOH.

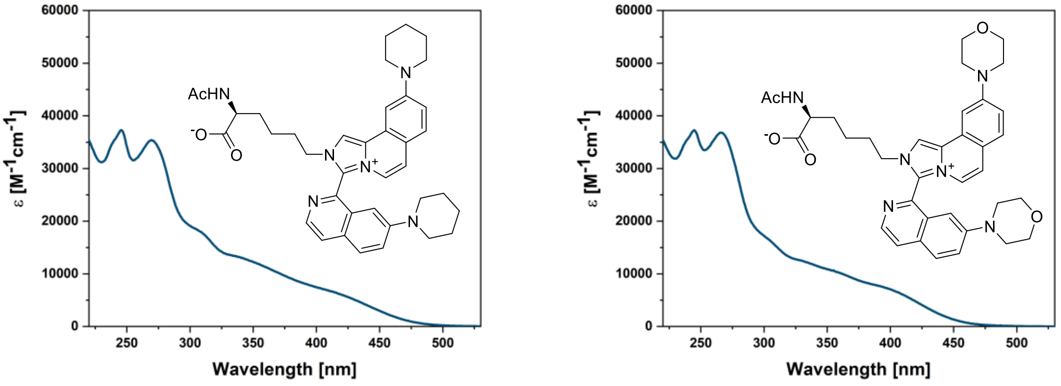

Figure S15. UV-Vis absorption spectra of 8e and 8f in MeOH.

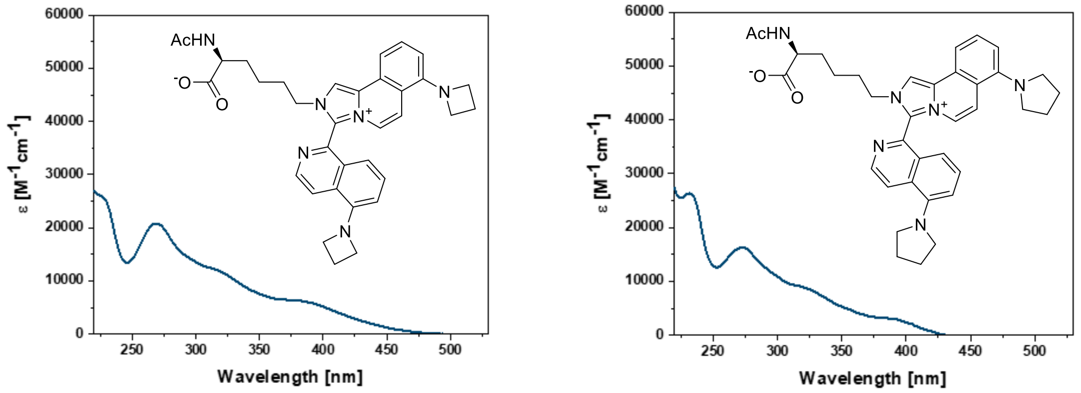

Figure S16. UV-Vis absorption spectra of 8e and 8f in MeOH.

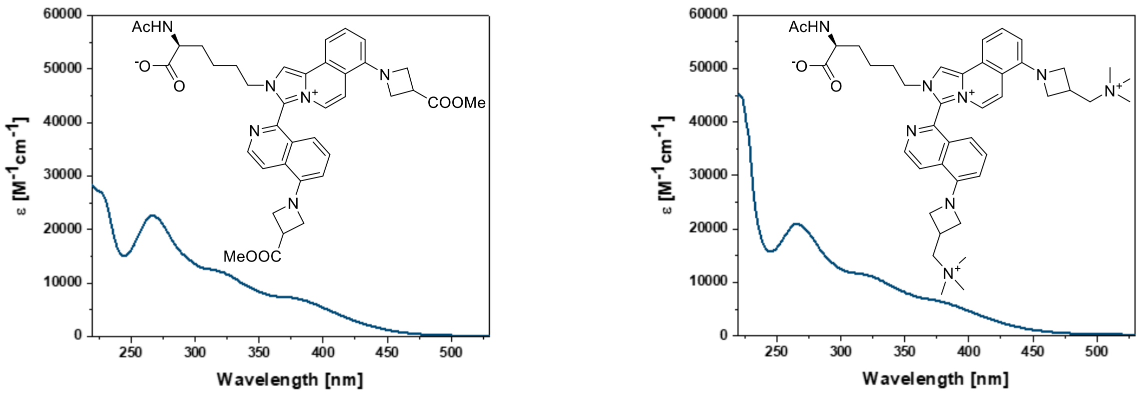

Figure S17. UV-Vis absorption spectra of 8e and 8f in MeOH.

4.1.2. PBS (1% MeOH)

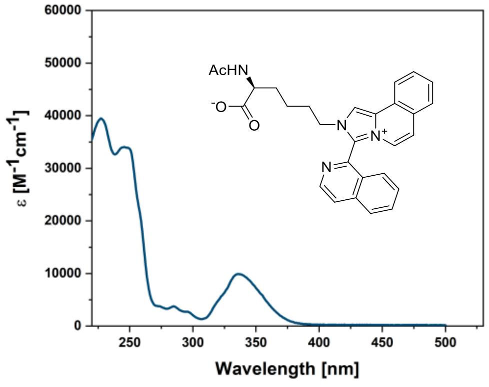

Figure S18. UV-Vis absorption spectra of 3 in PBS (1% MeOH).

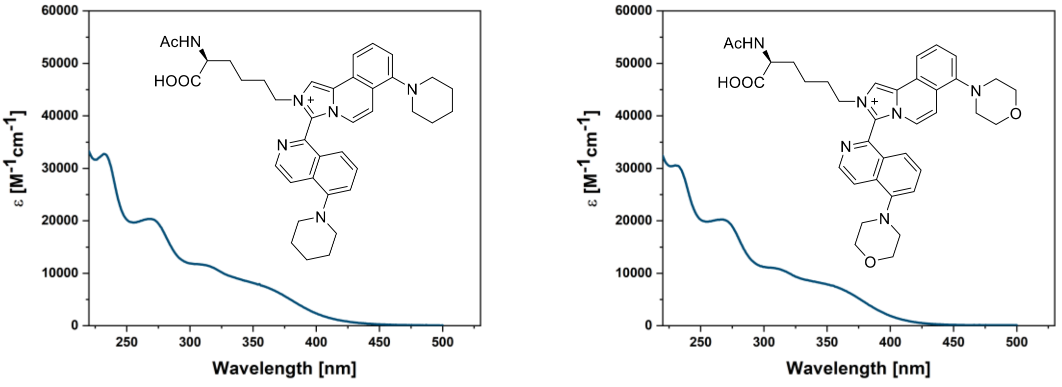

Figure S19. UV-Vis absorption spectra of 8a and 8d in PBS (1% MeOH).

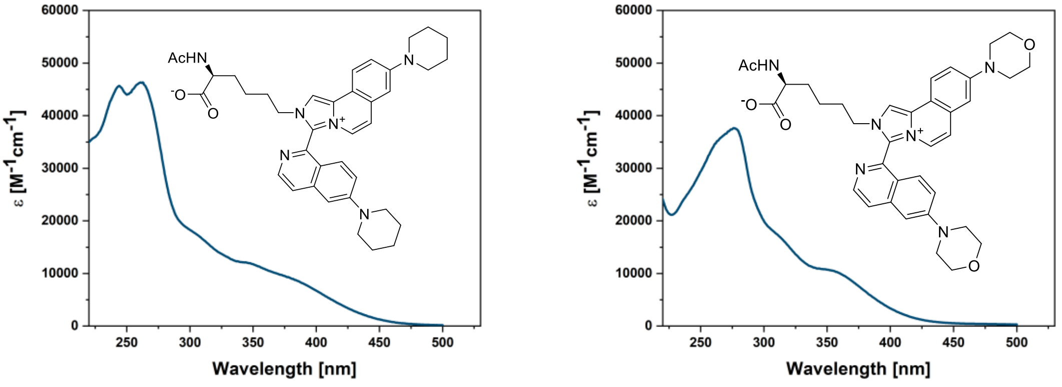

Figure S20. UV-Vis absorption spectra of 8b and 8e in PBS (1% MeOH).

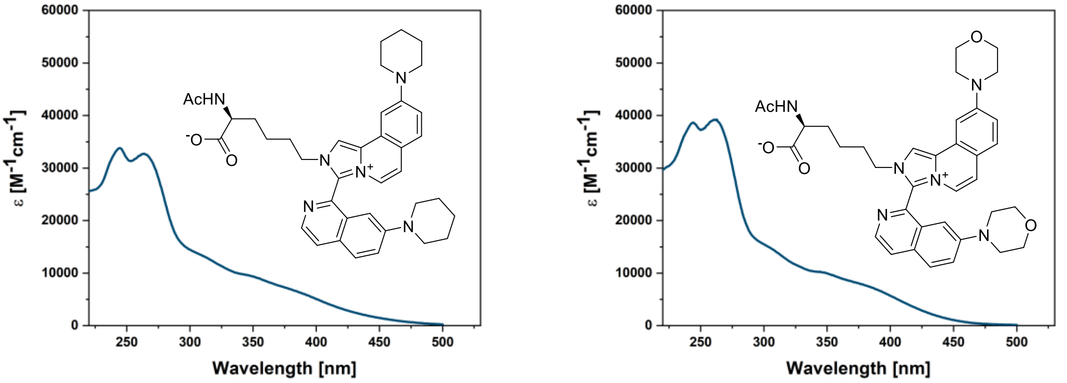

Figure S21. UV-Vis absorption spectra of 8c and 8f in PBS (1% MeOH).

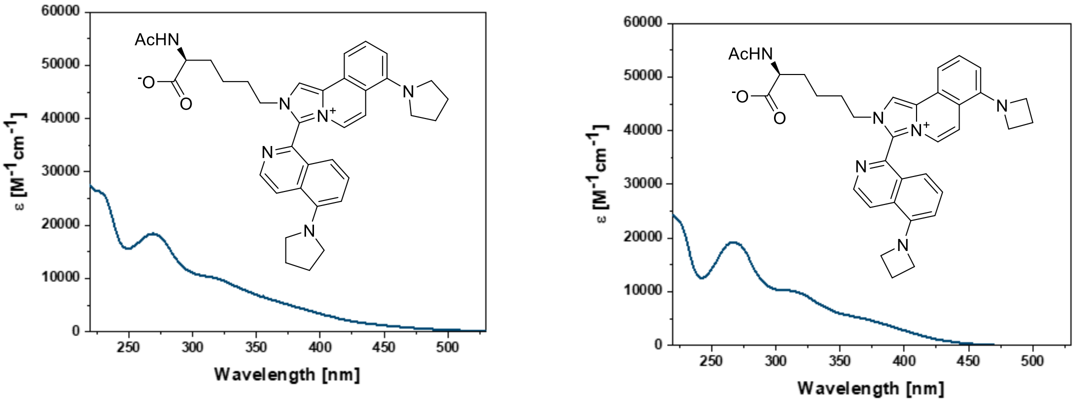

Figure S22. UV-Vis absorption spectra of 8g and 8h in PBS (1% MeOH).

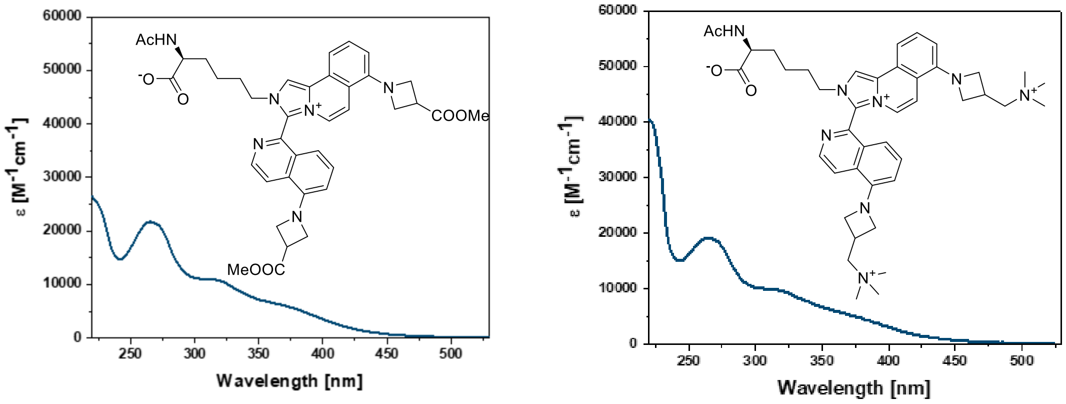

Figure S23. UV-Vis absorption spectra of 8i and 8l in PBS (1% MeOH).

4.2. Fluorescence Spectroscopy

Emission Spectra

To evaluate the UV-Vis emission profiles and the quantum yield of emission of our compounds, we conducted fluorescence measurements. For this, a stock-solution (2 mM, MeOH) of each dye was prepared and diluted to a final concentration of 10 µM at the solvent specified. Then, the excitation profile was determined, and the maximum excitation was chosen to record the emission spectrum of each dye. All measurements were processed using Spectragryph (v1.2.15), and OriginPro2020.

4.2.1. MeOH

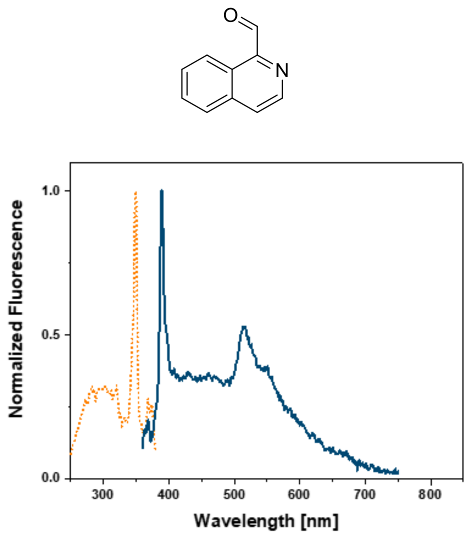

Figure S24. Excitation (orange dotted line) and emission (blue solid line) spectra of 3 in MeOH (10 µM).

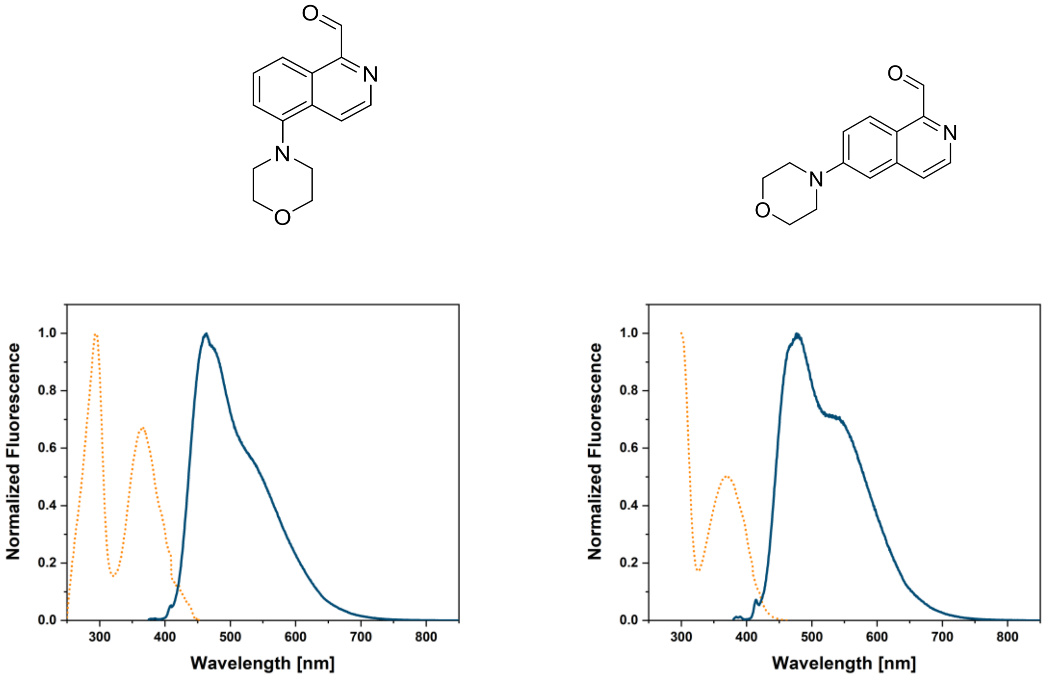

Figure S25. Excitation (orange dotted line) and emission (blue solid line) spectra of 3b and 3d in MeOH (10 µM).

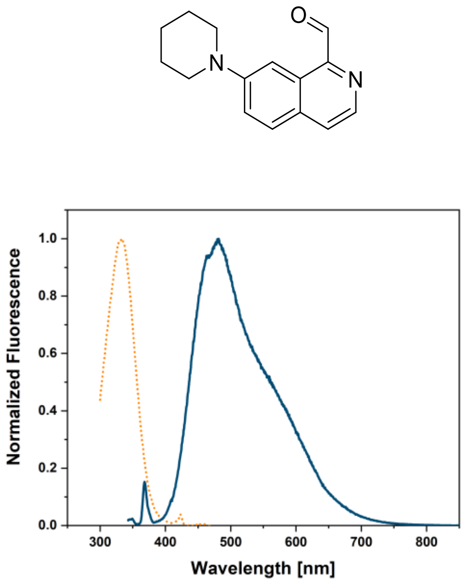

Figure S26. Excitation (orange dotted line) and emission (blue solid line) spectra of 3f in MeOH (10 μM).

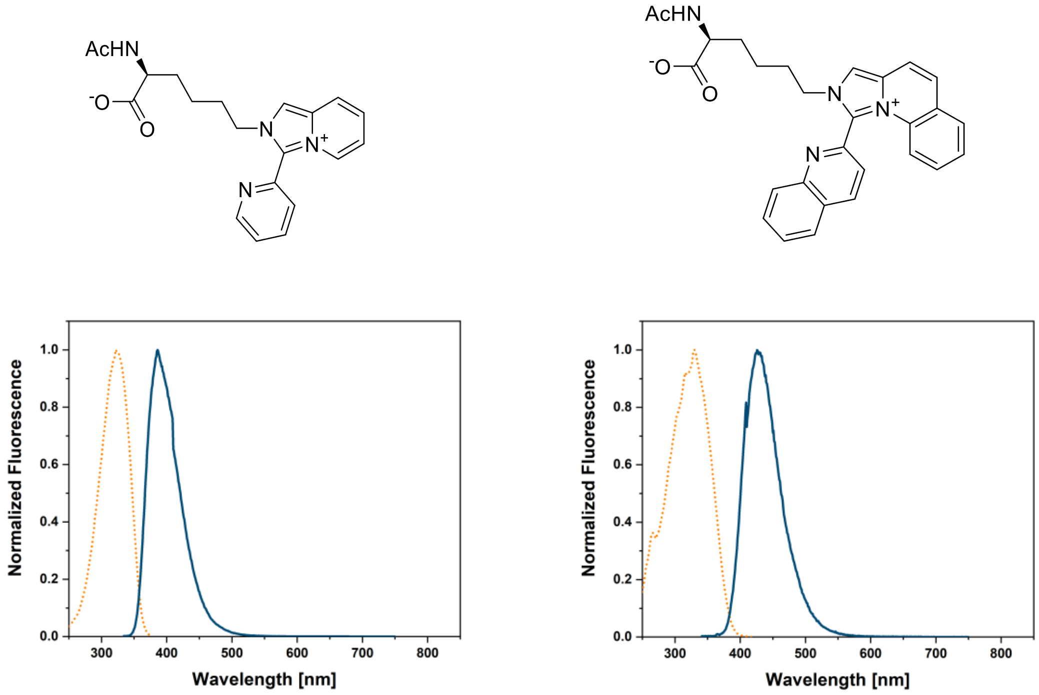

Figure S27. Excitation (orange dotted line) and emission (blue solid line) spectra of py-ip$^+$ and qu-ip$^+$ in MeOH (10 μM).

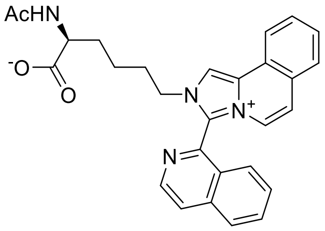

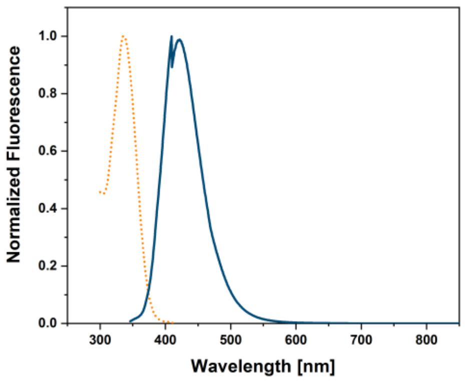

Figure S28. Excitation (orange dotted line) and emission (blue solid line) spectra of iqu-ip+ in MeOH (10 μM).

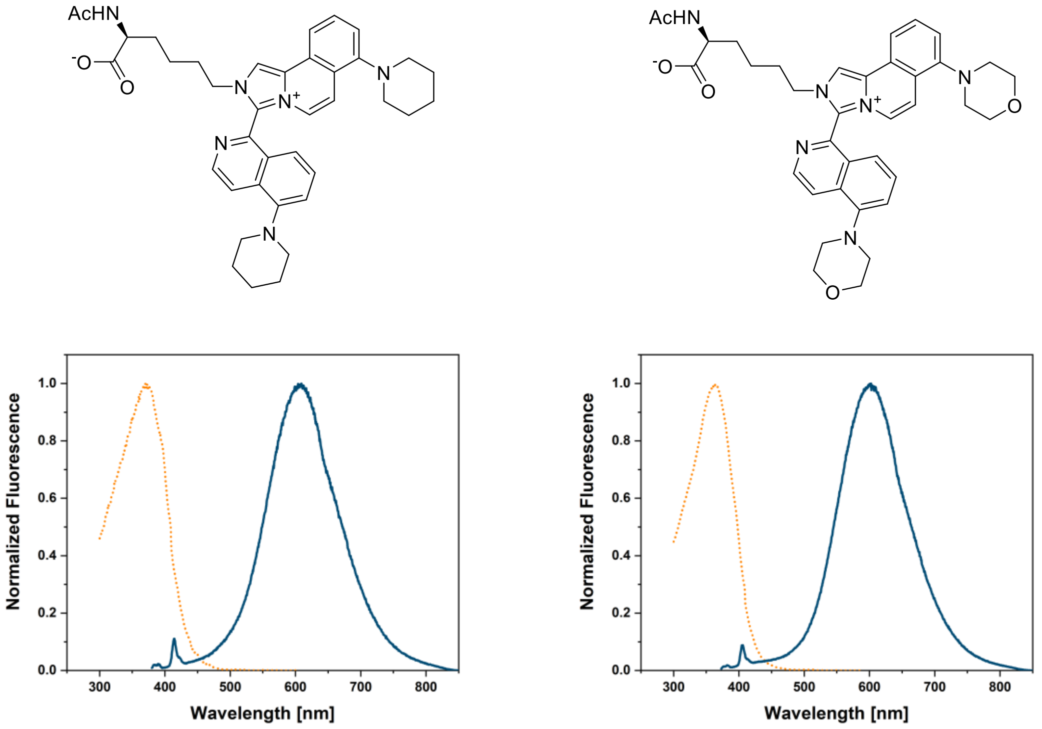

Figure S29. Excitation (orange dotted line) and emission (blue solid line) spectra of 8a and 8d in MeOH (10 μM).

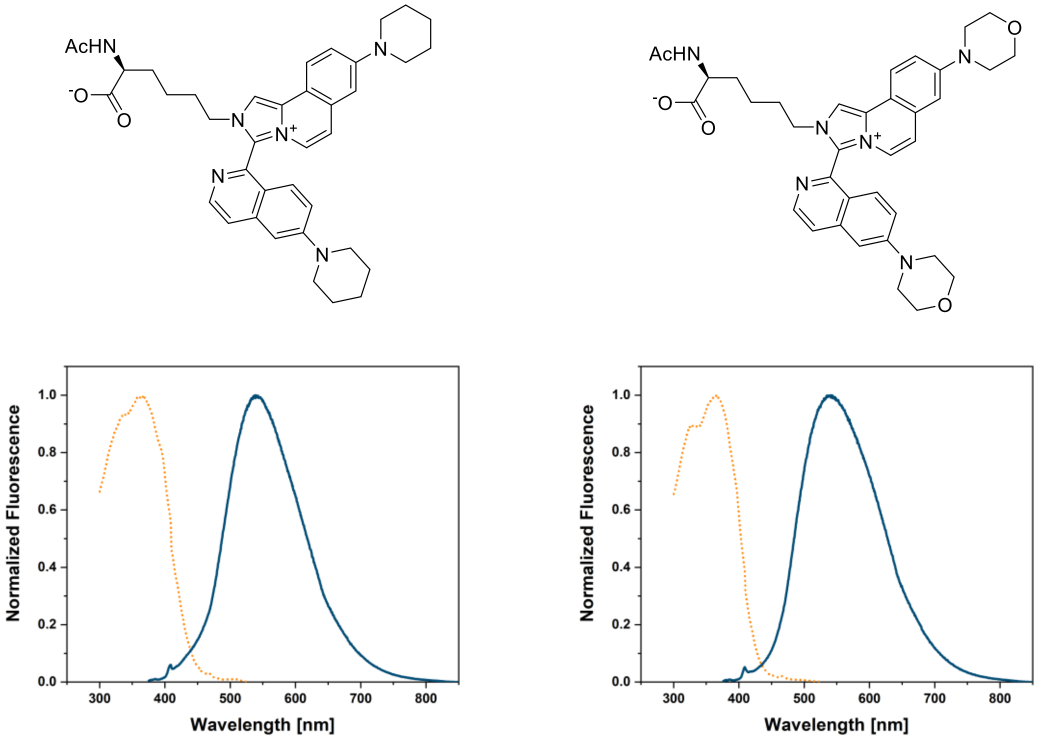

Figure S30. Excitation (orange dotted line) and emission (blue solid line) spectra of 8b and 8e in MeOH (10 μM).

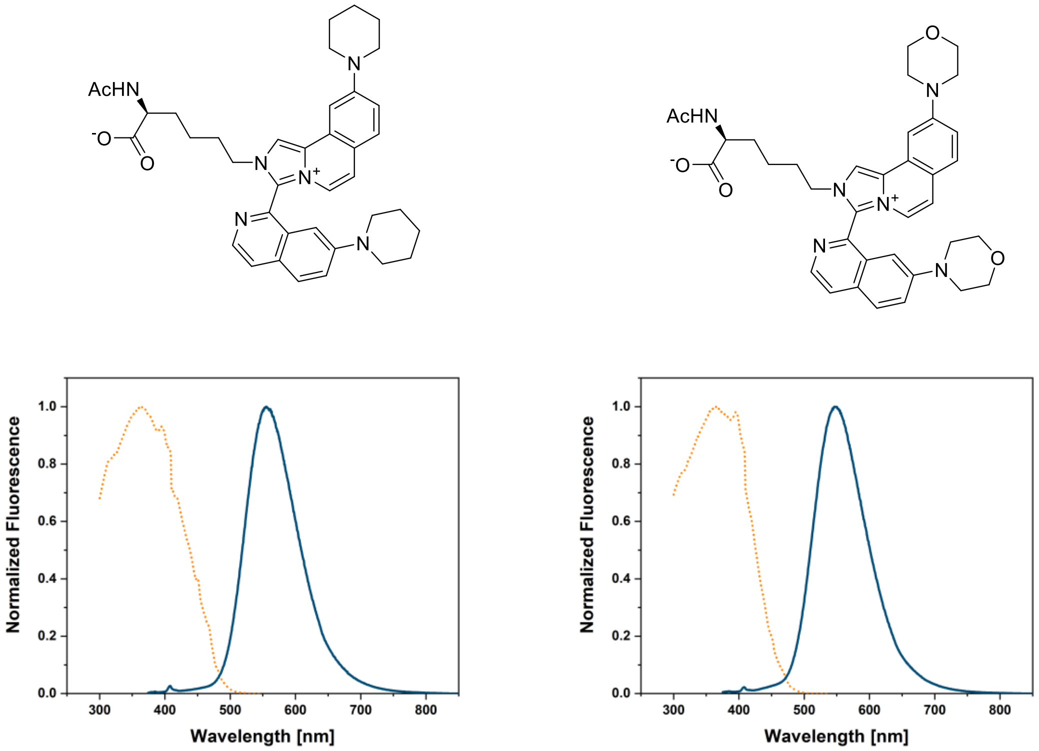

Figure S31. Excitation (orange dotted line) and emission (blue solid line) spectra of 8c and 8f in MeOH (10 μM).

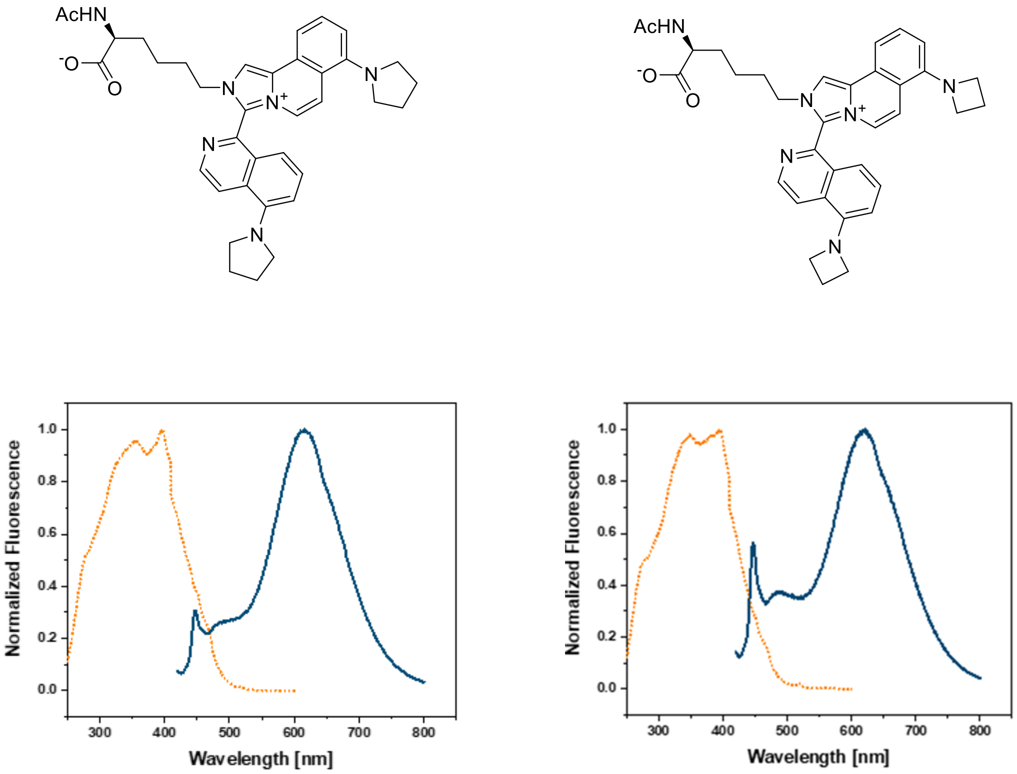

Figure S32. Excitation (orange dotted line) and emission (blue solid line) spectra of 8g and 8h in MeOH (10 μM).

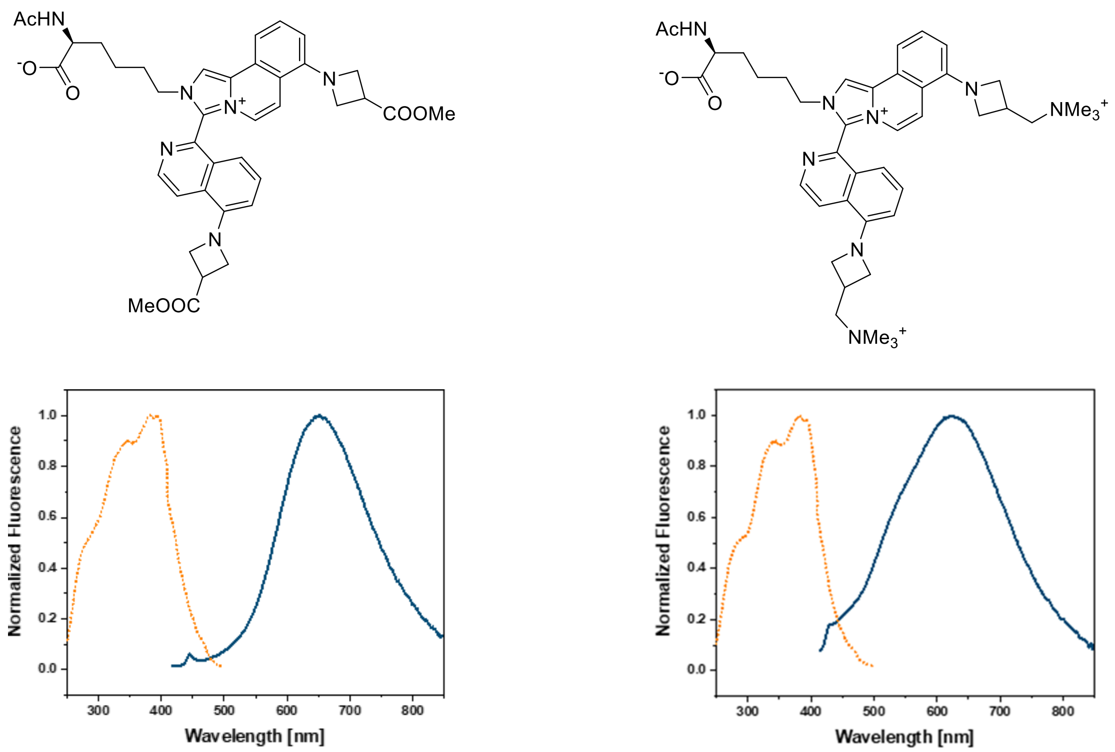

Figure S33. Excitation (orange dotted line) and emission (blue solid line) spectra of 8i and 8l in MeOH (10 μM).

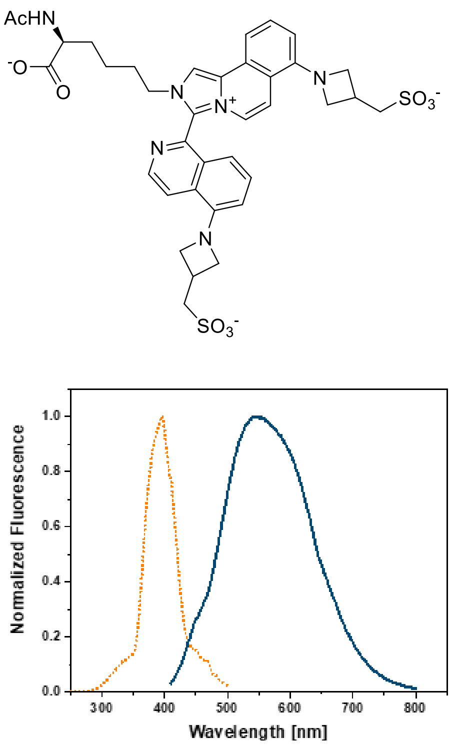

Figure S34. Excitation (orange dotted line) and emission (blue solid line) spectra of 8m in MeOH (10 μM).

4.2.2. PBS:MeOH (99:1)

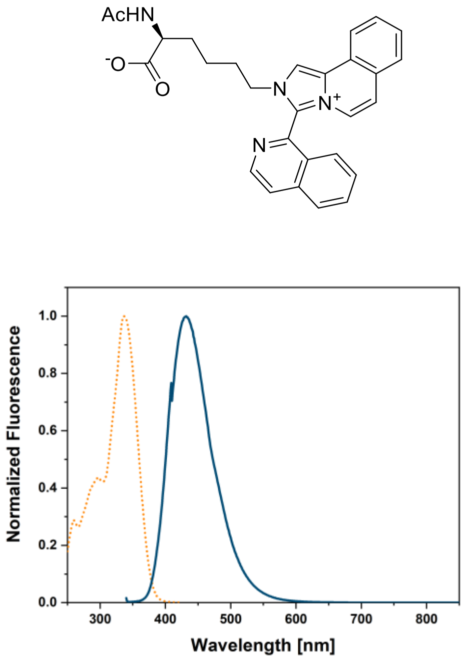

Figure S35. Excitation (orange dotted line) and emission (blue solid line) spectra of 8 in PBS (1% MeOH, 10 µM).

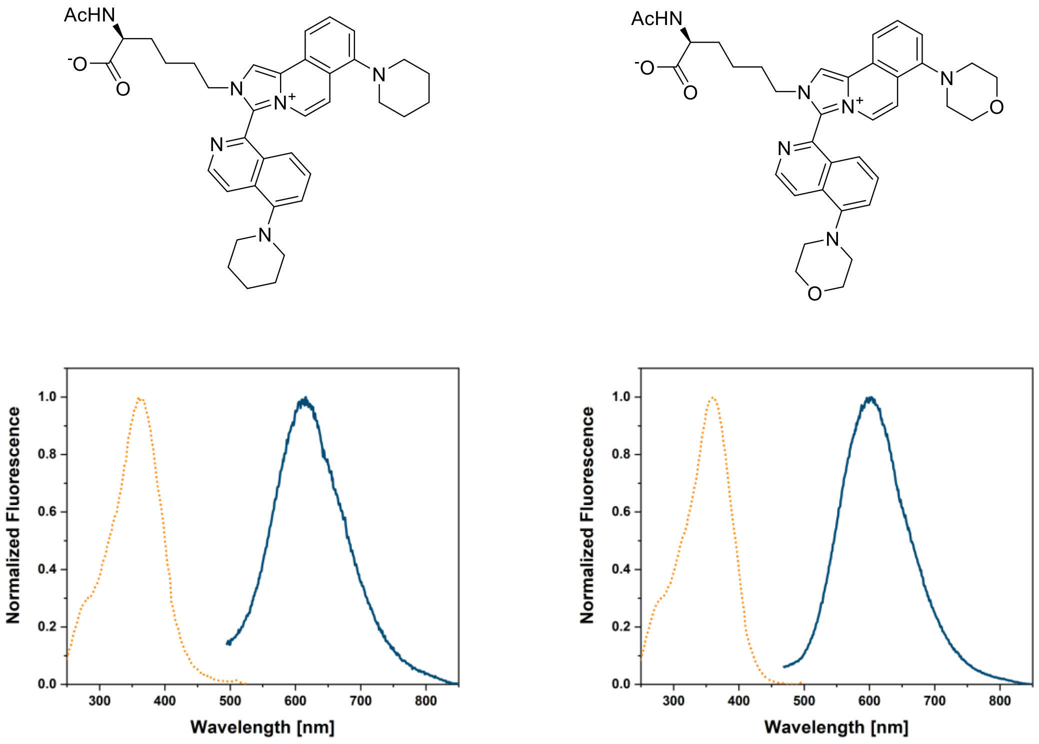

Figure S36. Excitation (orange dotted line) and emission (blue solid line) spectra of 8a and 8d in PBS (1% MeOH, 10 µM).

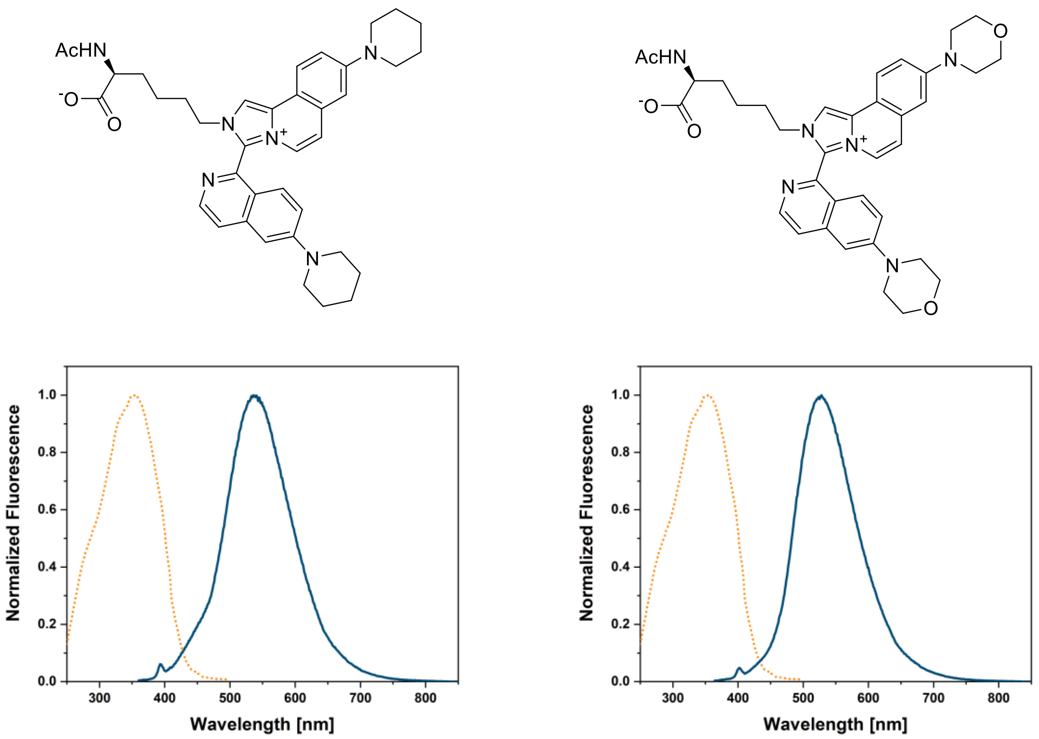

Figure S37. Excitation (orange dotted line) and emission (blue solid line) spectra of 8b and 8e in PBS (1% MeOH, 10 μM).

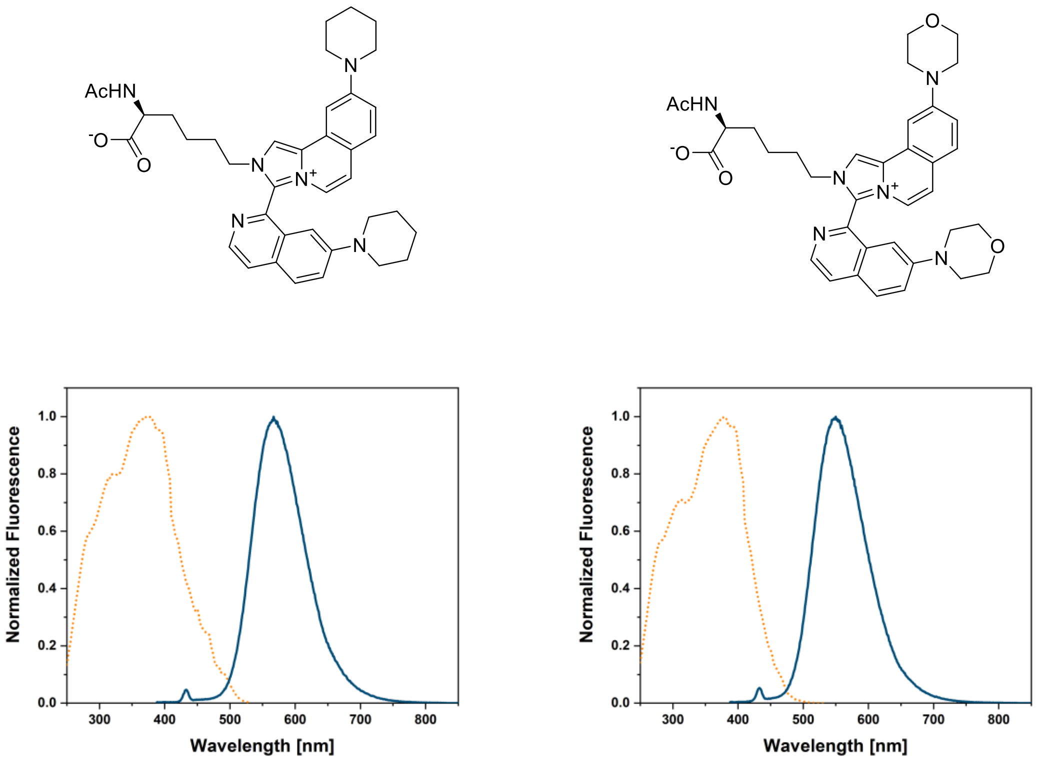

Figure S38. Excitation (orange dotted line) and emission (blue solid line) spectra of 8c and 8g in PBS (1% MeOH, 10 μM).

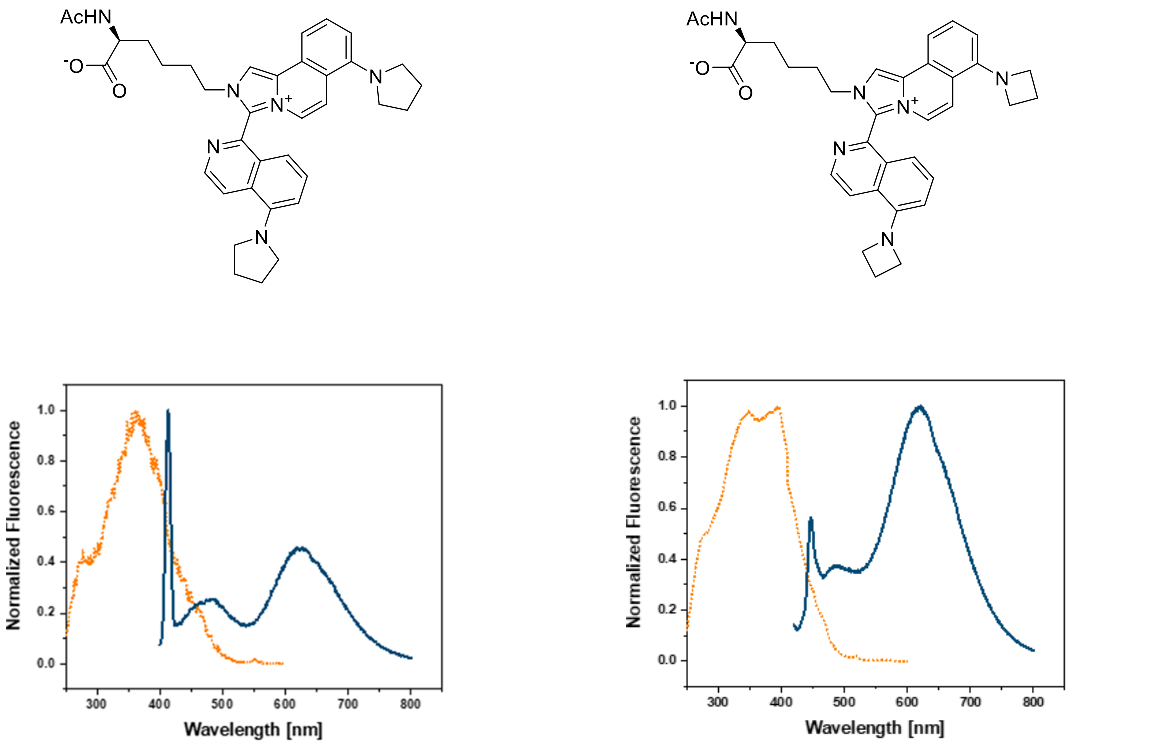

Figure S39. Excitation (orange dotted line) and emission (blue solid line) spectra of 8h and 8g in PBS (1% MeOH, 10 μM).

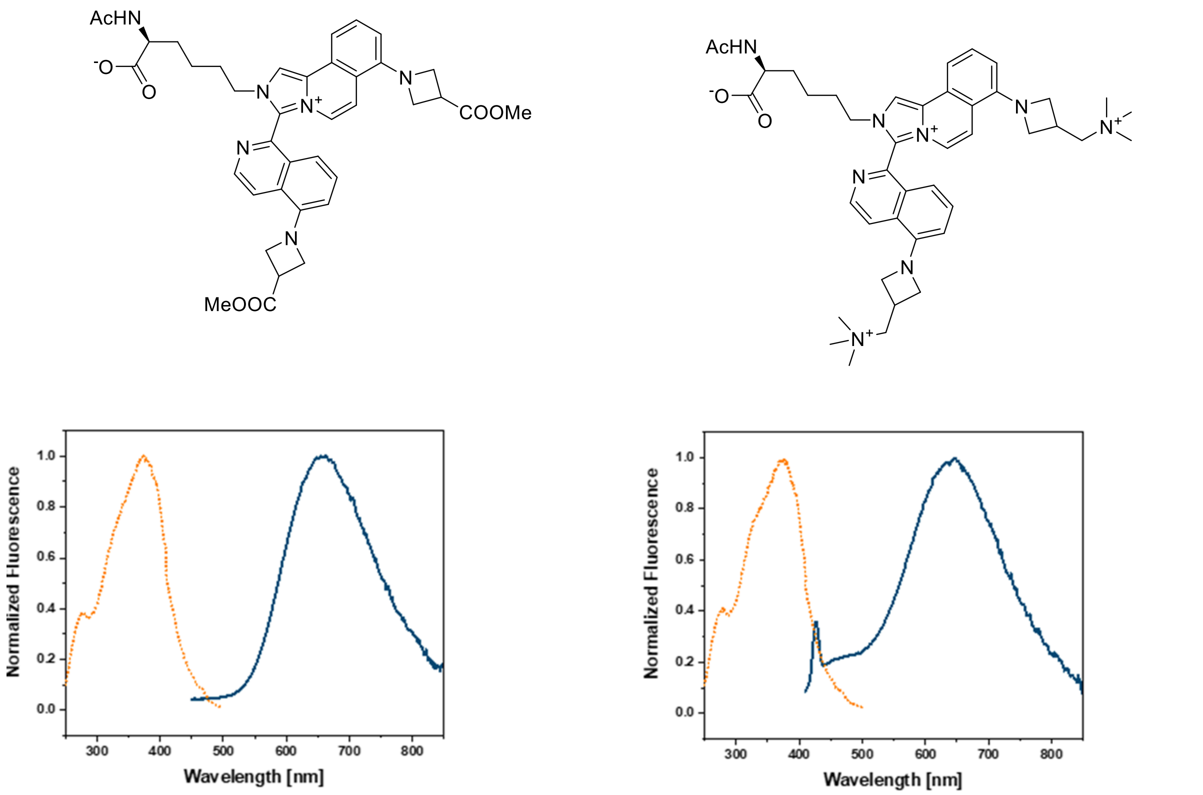

Figure S40. Excitation (orange dotted line) and emission (blue solid line) spectra of 8i and 8l in PBS (1% MeOH, 10 μM).

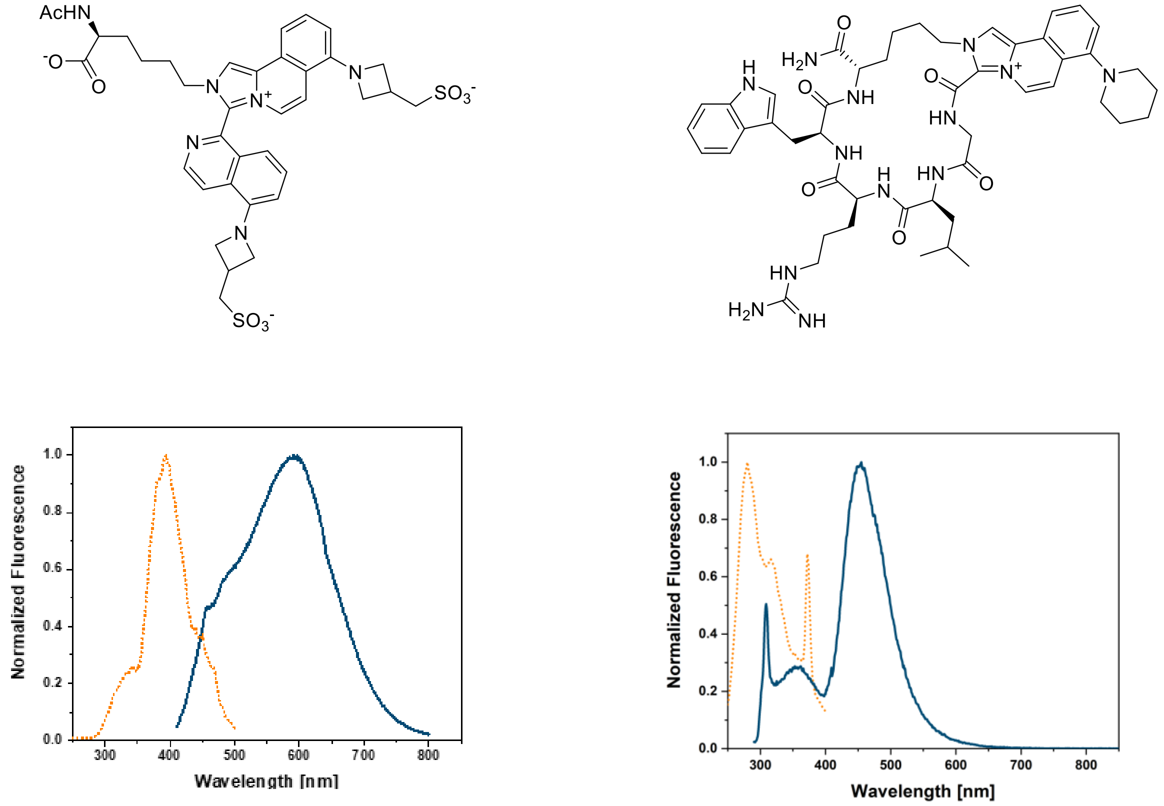

Figure S41. Excitation (orange dotted line) and emission (blue solid line) spectra of 8m and CP-IP⁺ in PBS (1% MeOH, 10 μM).

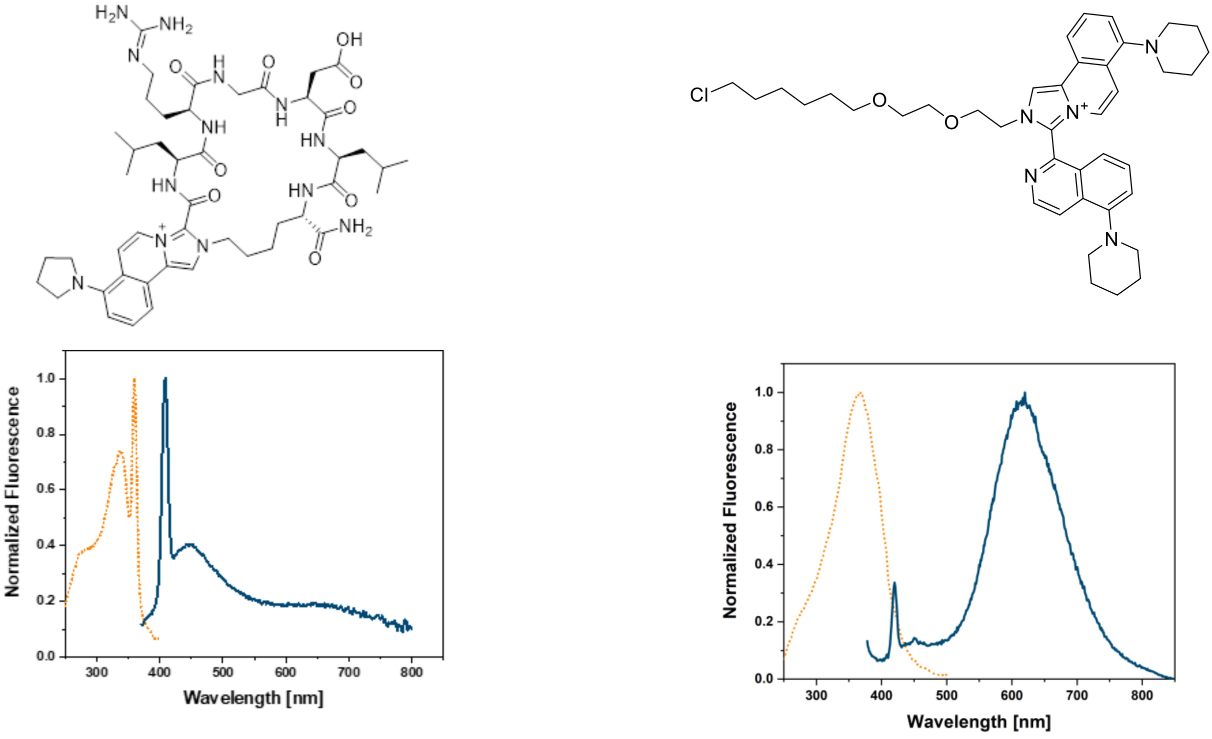

Figure S42. Excitation (orange dotted line) and emission (blue solid line) spectra of cRGD-IP⁺ and Halo-IP⁺ in PBS (1% MeOH, 10 μM).

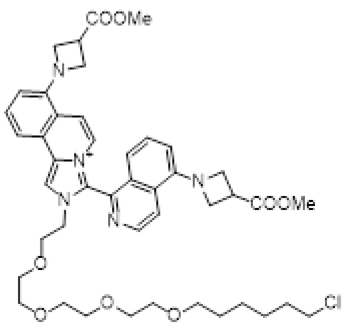

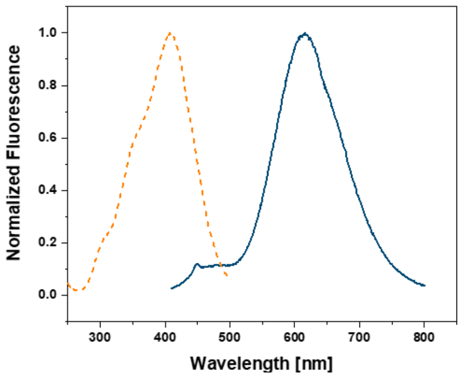

Figure S43. Excitation (orange dotted line) and emission (blue solid line) spectra of Halo-azIP⁺ in PBS (1% MeOH, 10 µM).

4.2.3. PBS:MeOH (1:1)

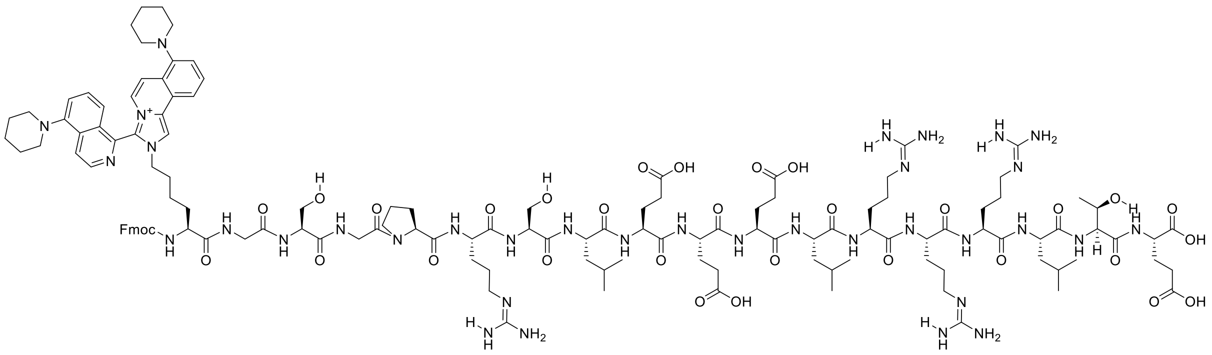

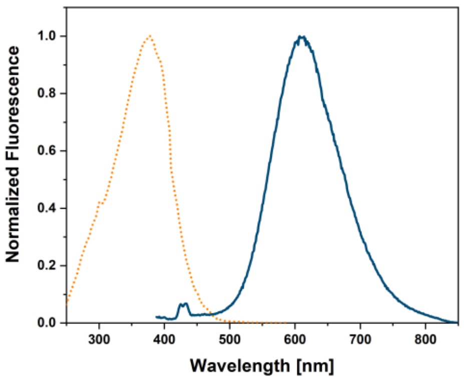

Figure S44. Excitation (orange dotted line) and emission (blue solid line) spectra of ALFA-IP⁺ in PBS (50% MeOH, 10 µM).

4.2.4. pH Dependency of **8i** in buffer:MeOH (99:1)

To evaluate the pH dependency of emission of our compounds, we conducted fluorescence measurements. For this, a stock-solution (2 mM, MeOH) of 8i was prepared and diluted to a final concentration of 10 µM at the pH buffer specified. Then, the excitation profile was determined, and the maximum excitation was chosen to record the emission spectrum of each dye. All measurements were processed using Spectragryph (v1.2.15), and OriginPro2020

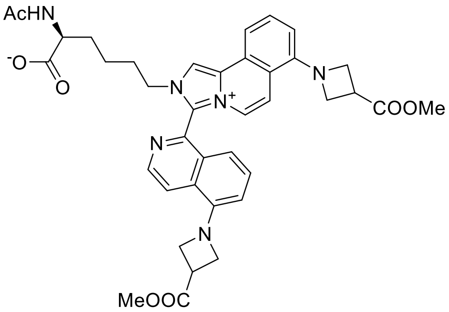

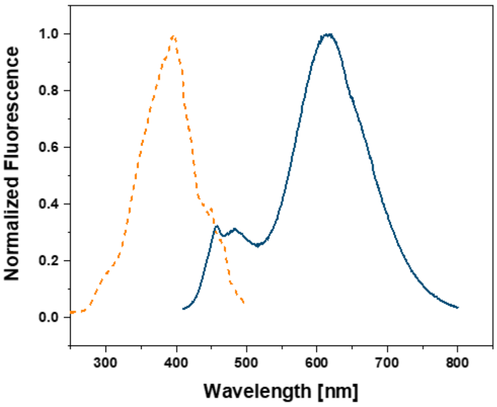

pH 4.0 sodium acetate buffer solution

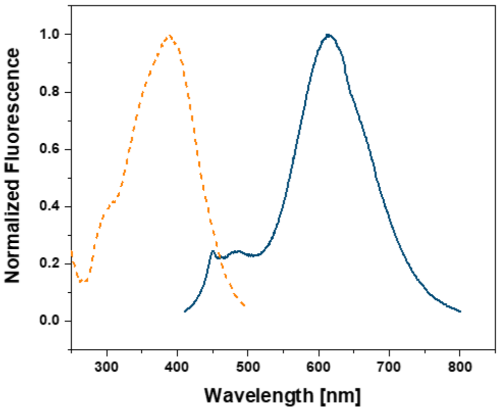

pH 5.6 sodium acetate buffer solution

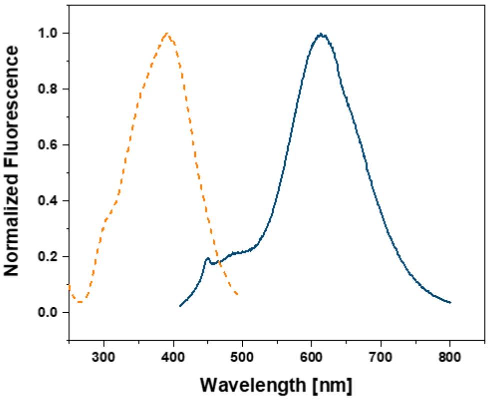

pH 9.2 sodium bicarbonate buffer solution

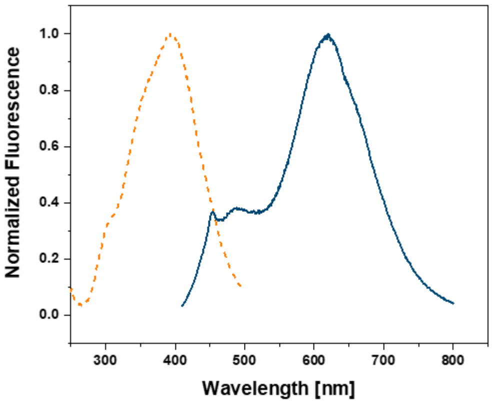

pH 10.6 sodium bicarbonate buffer solution

Figure S45. Excitation (orange dotted line) and emission (blue solid line) spectra of 8i in buffers at different pH values (1% MeOH, 10 µM).

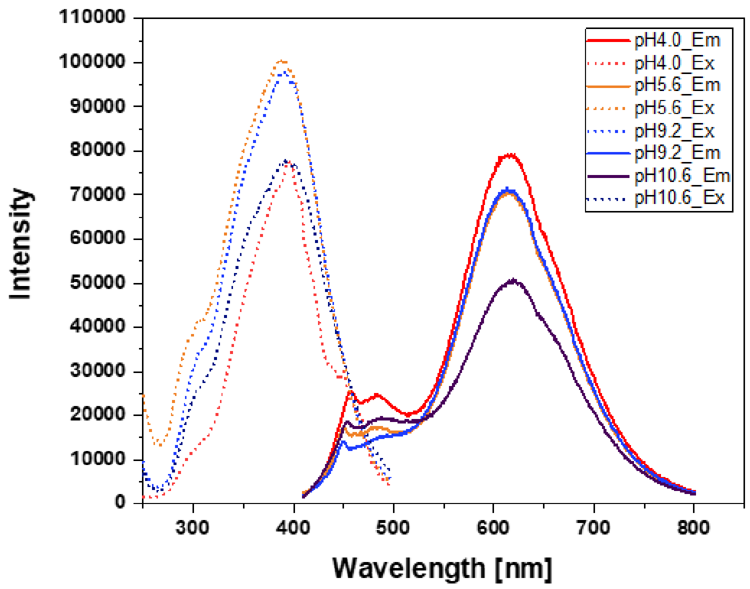

Figure S46. Overlaps of the excitation (orange dotted line) and emission (blue solid line) spectra of 8i in buffers at different pH values (1% MeOH, 10 μM) without normalizing.

5. Chemical Synthesis

5.1. General Procedures

General Procedure A — Synthesis of bromoisoquinoline-2-oxides 5a–c.

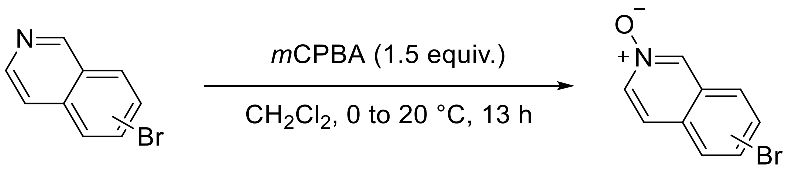

4 **5**

Bromoisoquinoline (1.0 equiv.) was dissolved in CH_2Cl_2 (0.5 M), added to a 250 mL round bottom flask, and cooled to 0 °C. Then, *meta*-chloroperoxybenzoic acid (*m*CPBA, 1.5 equiv.) was added at 0 °C. The reaction mixture was left stirring at 0 °C for 1 h and then, was slowly allowed to warm to room temperature and stirred for 12 h. The reaction mixture was subsequently filtered through a pad of celite, and the filter cake was washed with CH_2Cl_2. The collected organic layer was washed successively with saturated aqueous solution of sodium bicarbonate and then brine, dried over anhydrous sodium sulfate, filtered, and concentrated under reduced pressure to afford the desired bromoisoquinoline-2-oxide **5a–c**.

Compounds **5a**,[1] **5b**,[2] and **5c**[3] were obtained *via* Method A and ^{1}H NMR analysis was in agreement with literature.

General Procedure B — Synthesis of 1-methyl-bromoisoquinolines 6a–c.

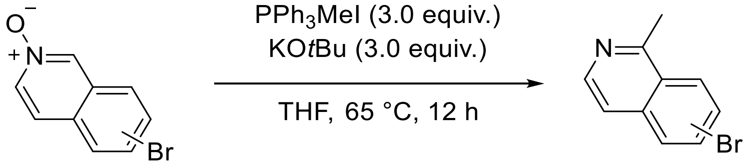

5 **6**

Methyltriphenylphosphonium iodide (PPh_3MeI, 3.0 equiv.) and potassium *tert*-butoxide (KO*t*Bu, 3.0 equiv.) were added to a 250 mL three necked round bottom flask equipped with a condenser and dissolved in dry THF (1.2 M) under N_2 atmosphere at room temperature. The mixture was stirred for 30 minutes and bromoisoquinoline-2-oxide (1.0 equiv.) was added. The reaction mixture was stirred at 65 °C for 16 h. The mixture was poured onto water and extracted with CH_2Cl_2. The collected organic layers were washed with brine, dried over anhydrous sodium sulfate, filtered, and concentrated under reduced pressure. The remaining mixture was purified by flash column chromatography (silica gel, acetone in pentane 10–25%) to afford the desired 1-methyl-bromoisoquinoline **6a–c**.

Compounds **6a,**[4] **6b**,[5] and **6c** [5] were obtained *via* Method B and ^{1}H NMR analysis was in agreement with literature.

General Procedure C — Buchwald-Hartwig Coupling to obtain compounds 7a–f.

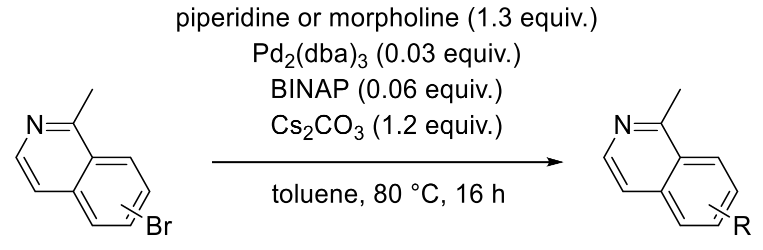

6 **7**

1-Methyl-bromoisoquinoline (1.0 equiv.), bis(dibenzylidenaceton)-palladium(0) (Pd_2dba_3, 0.06 equiv. equiv.), 2,2'-bis(diphenylphosphino)-1,1'-binaphthyl (BINAP, 0.06 equiv.), caesium carbonate (1.2 equiv.), and piperidine or morpholine, respectively, (1.3 equiv.) were added to a 250 mL three necked round bottom flask equipped with a condenser and dissolved in dry toluene (1.0 M) under N_2 atmosphere at room temperature. The reaction mixture was stirred at 80 °C for 16 h and subsequently filtered through a pad of celite, and the filter cake was washed with CH_2Cl_2. The collected organic layers were washed successively with saturated aqueous solution of sodium bicarbonate and then brine, dried over anhydrous sodium sulfate, filtered, and concentrated under reduced. The remaining mixture was purified by automated flash column chromatography (silica gel, ethyl acetate in pentane 20–50%) to afford the 1-methyl-isoquinoline derivatives **7a-f**.

Compounds **7a**, **7b**, **7c**, **7d**, **7e**, and **7f** were obtained *via* Method C.

General Procedure D — Synthesis of isoquinoline-1-aldehydes 3 and 3a–f.

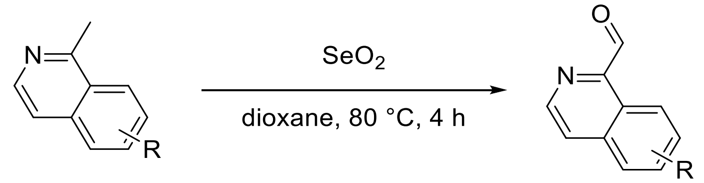

7 **3**

Selenium dioxide (1.3 equiv.) was added to a 100 mL round bottom flask, dissolved in dioxane (0.5 M), and heated to 80 °C for 1 h. Then, 1-methyl-isoquinoline derivative **3** (1.0 equiv.) was added. The reaction mixture was left stirring at 80 °C for 3 h. The reaction mixture was subsequently filtered through a pad of celite, and the filter cake was washed with CH_2Cl_2. The collected organic layers were washed successively with saturated aqueous solution of sodium bicarbonate and then brine, dried over anhydrous sodium sulfate, filtered, and concentrated under reduced. The remaining mixture was purified by automated flash column chromatography (silica gel, ethyl acetate in pentane 10%) to afford the isoquinoline derivatives **3**.

Compounds **3a**, **3b**, **3c**, **3d**, **3e**, **3f**, and **3**[6] were obtained *via* Method D. ^{1}H NMR analysis of **3** was in agreement with literature.

General Procedure E — Labelling Reaction of Small Molecules

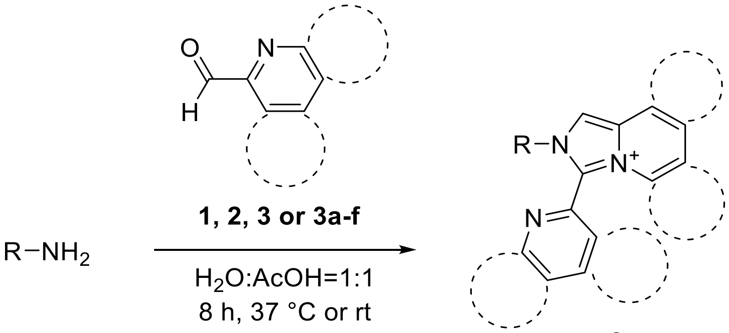

8

A suitable amine (1.0 equiv.) was added to a 25 mL round bottom flask and dissolved in AcOH: H_2O = 1:1 (v/v, 25 mM). Then, the corresponding pyridine, quinoline, or isoquinoline aldehyde (2.0 equiv.) was added. The reaction mixture was left stirring at 37 °C or room temperature. After the full conversion, the solvent was removed under reduced pressure and the remaining mixture was purified by automated reverse phase column chromatography (C18, 5 to 95% of ACN+0.1% AcOH in H_2O+0.1% AcOH) to afford the desired *in situ* constructed fluorophores **8**.

An overview of the labelling products is displayed in Table S2.

Table S2. Overview of labelling products.

Starting Material ($R{-}NH_2$)	Labellin g Reagent	Condition s [°C/h]	Product
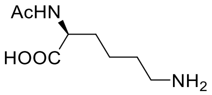	**1**	37 °C/24h	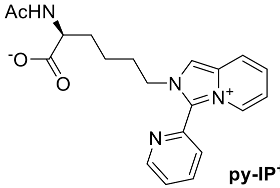 **py-IP$^+$**
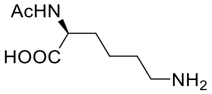	**2**	37 °C/48h	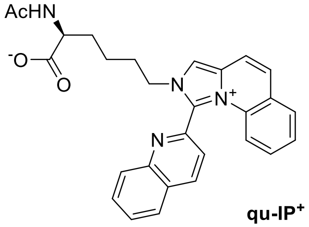 **qu-IP$^+$**
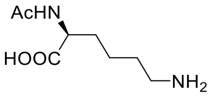	**3**	37 °C/12h	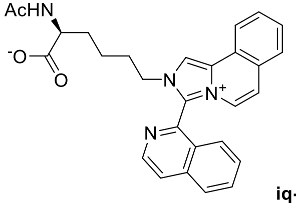 **iq-IP$^+$**
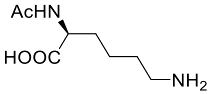	**3a**	37 °C/12h	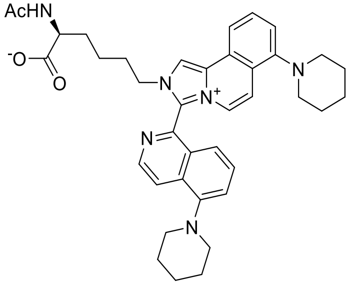 **8a**

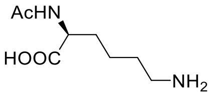	**3b**	37 °C/12h	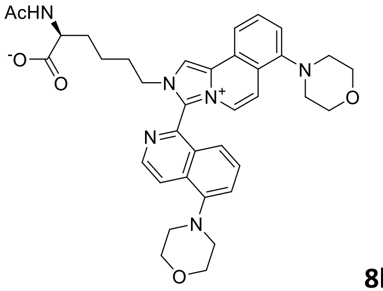 **8b**
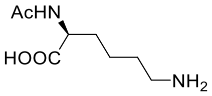	**3c**	37 °C/12h	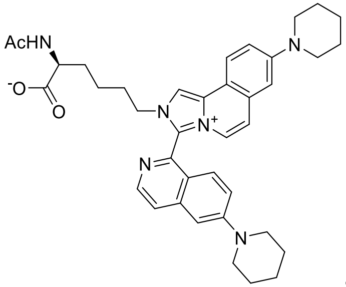 **8c**
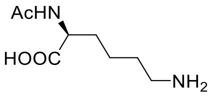	**3d**	37 °C/12h	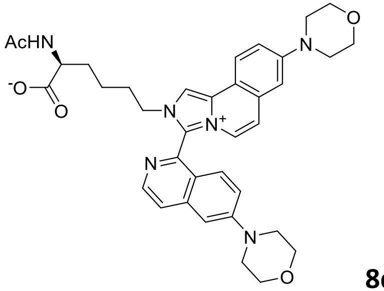 **8d**
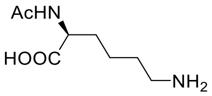	**3e**	37 °C/12h	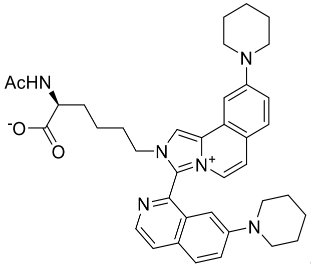 **8e**

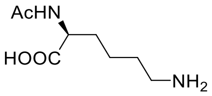	**3f**	37 °C/12h	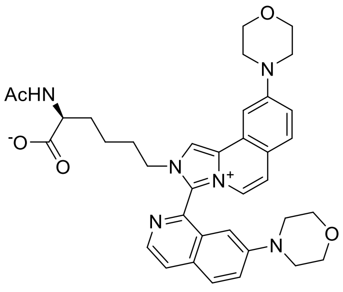 **8f**
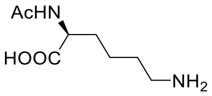	**3g**	37 °C/12h	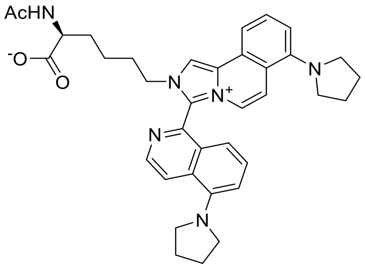 **8g**
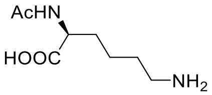	**3h**	37 °C/12h	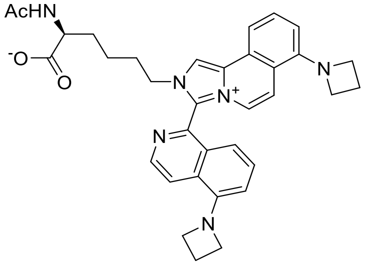 **8h**
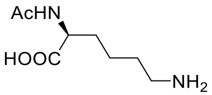	**3i**	37 °C/12h	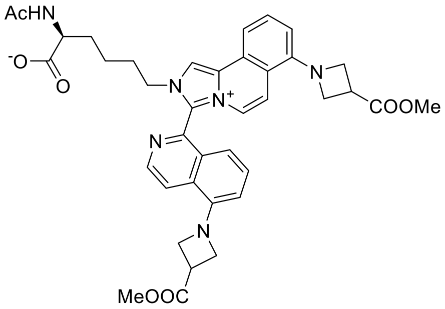 **8i**

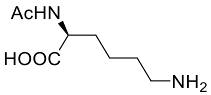	**3l**	37 °C/12h	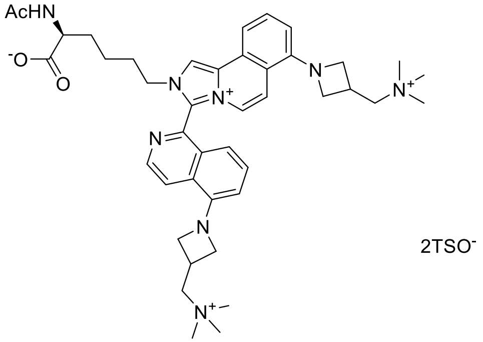
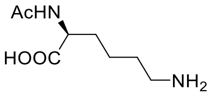	**3m**	37 °C/12h	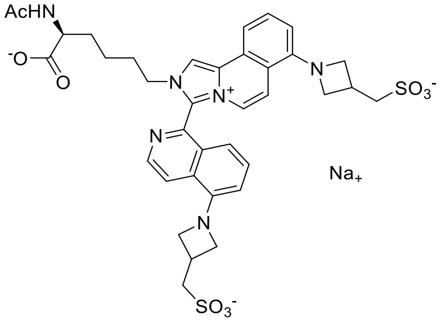
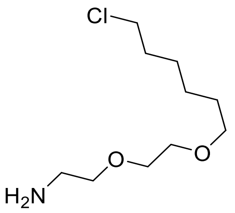	**3a**	rt/12h	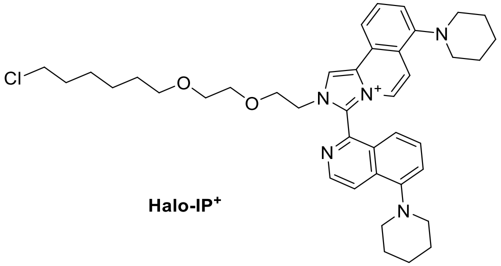

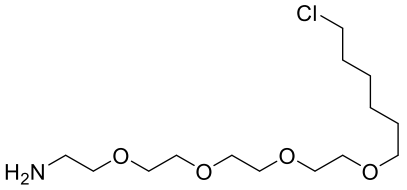	**3i**	rt/12h	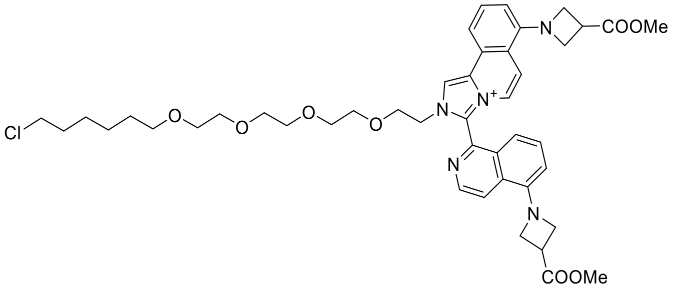 **Halo-azIP$^+$**
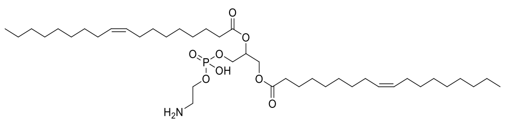	**3b**	40°C/4h	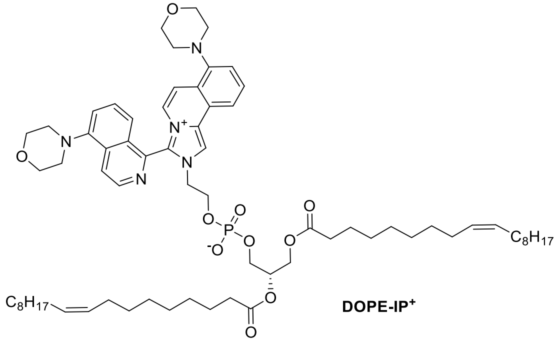 **DOPE-IP$^+$**
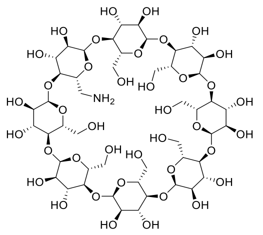 amino-α-cyclodextrin	**3b**	rt/24h	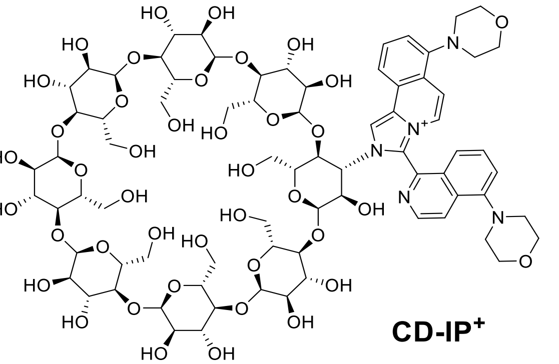 **CD-IP$^+$**

**Fmoc-K-GSG-ALFA-OH**	**3a**	rt/12h	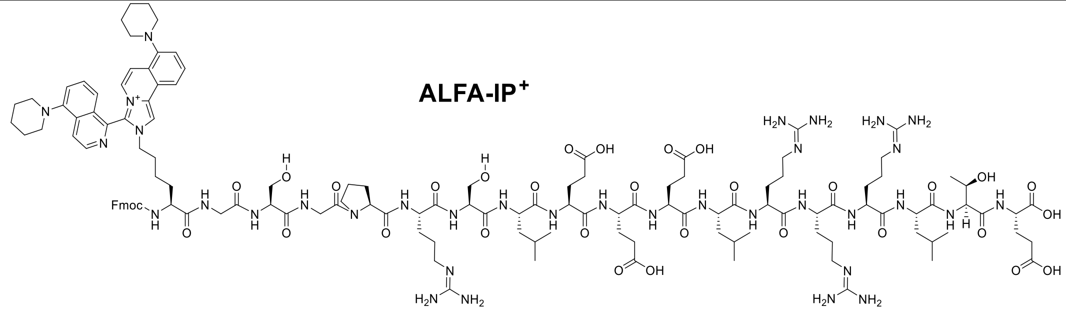 **ALFA-IP$^+$**
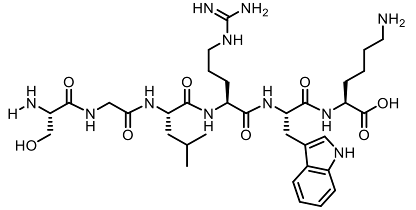 **NH_2-SGLRWK-OH**	**3a**	rt/12h	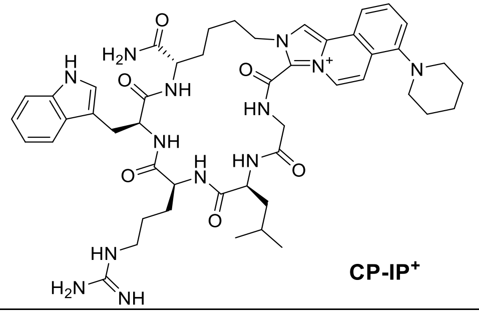 **CP-IP$^+$**
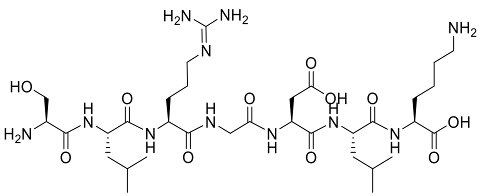 **NH_2-SRGDLK-OH**	**3g**	rt/12h	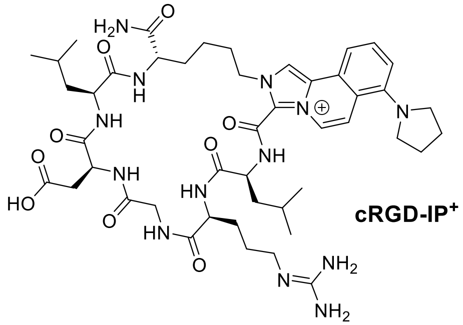 **cRGD-IP$^+$**

5.2. **Compound Characterization**

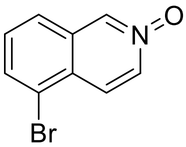

5-Bromoisoquinoline 2-oxide (**5a**).
5a was synthesized *via* method A from **4a** (4.1 g, 19.8 mmol) and obtained as a colorless powder in 90% yield (4.0 g, 18.0 mmol). ^{1}H NMR analysis was in agreement with literature.[1]

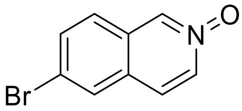

6-Bromoisoquinoline 2-oxide (**5b**).
5b was synthesized *via* method A from **4b** (4.1 g, 19.8 mmol) and obtained as a colorless powder in 97% yield (4.3 g, 19.2 mmol). ^{1}H NMR analysis was in agreement with literature.[2]

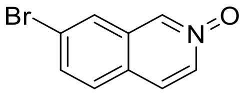

7-Bromoisoquinoline 2-oxide (5c).
5c was synthesized *via* method A from **4c** (1.9 g, 7.7 mmol) and obtained as a colorless powder in 95 % yield (4.2 g, 18.8 mmol). ^{1}H NMR analysis was in agreement with literature.[3]

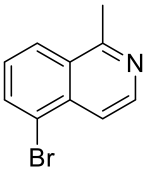

5-Bromo-1-methylisoquinoline (6a).
6a was synthesized *via* method B from **5a** (4.0 g, 18.0 mmol) and obtained as colorless crystals in 76 % yield (3.0 g, 13.7 mmol). ^{1}H NMR analysis was in agreement with literature.[4]

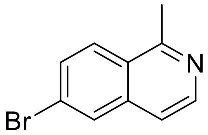

6-Bromo-1-methylisoquinoline (6b).

6b was synthesized *via* method B from **5b** (4.0 g, 18.0 mmol) and obtained as a colorless crystal in 70 % yield (2.8 g, 12.6 mmol). ^{1}H NMR analysis was in agreement with literature.[5]

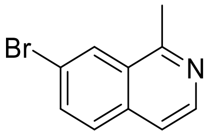

5-bromo-1-methylisoquinoline (6c).
6c was synthesized *via* method B from **5c** (4.0 g, 18.0 mmol) and obtained as a colorless crystal in 83 % yield (3.3 g, 14.9 mmol). ^{1}H NMR analysis was in agreement with literature.[5]

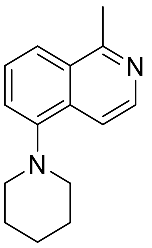

1-Methyl-5-(piperidin-1-yl)isoquinoline (7a).

7a was synthesized *via* method C from **6a** (882 mg, 4.0 mmol) and obtained as a yellow solid in 88 % yield (815 mg, 3.5 mmol). R_f = 0.29 (*n*-pentane: ethyl acetate, 1:1).

^{1}H NMR (400 MHz, CDCl3) δ 8.37 (d, J = 6.0 Hz, 1H), 7.84 – 7.79 (m, 1H), 7.77 (dq, J = 8.5, 1.1 Hz, 1H), 7.49 (ddd, J = 8.1, 7.4, 1.5 Hz, 1H), 7.21 (dq, J = 7.4, 1.0 Hz, 1H), 3.03 (m, 4H), 2.94 (d, J = 1.4 Hz, 3H), 1.84 (p, J = 5.6 Hz, 4H), 1.73 – 1.58 (m, 2H).

^{13}C NMR (101 MHz, $CDCl_3$) δ 158.8, 150.7, 141.1, 132.1, 128.7, 127.0, 119.9, 118.0, 115.5, 54.5, 26.5, 24.5, 22.8.

HRMS (ESI Pos): for [C15H19N2]+ calcd. m/z = 227.1548, found m/z = 227.1543.

IR (ATR): $\tilde{\nu}$ [cm-1] = 3895, 2848, 2796, 1580, 1493, 1415, 1383, 1233, 936, 747.

M_p: 84–86 °C

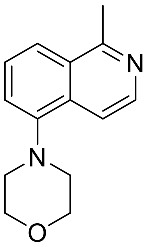

4-(1-Methylisoquinolin-5-yl)morpholine (7b).

7b was synthesized *via* method C from **6a** (882 mg, 4.0 mmol) and obtained as a yellow solid in 80 % yield (733 mg, 3.2 mmol). R_f = 0.25 (*n*-pentane: ethyl acetate, 1:1).

^{1}H NMR (400 MHz, $CDCl_3$) δ 8.39 (d, *J* = 6.0 Hz, 1H), 7.87 – 7.80 (m, 2H), 7.52 (dd, *J* = 8.5, 7.5 Hz, 1H), 7.26 (dd, *J* = 7.5, 1.0 Hz, 1H), 4.01 – 3.95 (m, 4H), 3.13 – 3.06 (m, 4H), 2.96 (s, 3H).

^{13}C NMR (101 MHz, $CDCl_3$) δ 159.1, 149.1, 141.6, 131.8, 128.7, 127.0, 120.9, 118.2, 115.0, 67.3, 53.4, 22.9.

HRMS (ESI Pos): for [C14H17N2O]+ calcd. m/z = 229.1341, found m/z = 229.1335.

IR (ATR): $\tilde{\nu}$ [cm^{-1}] = 2952, 2853, 2816, 1580, 1495, 1449, 1416, 1382, 1296, 1235, 1112, 941, 854, 755, 696.

M_p 78–80°C

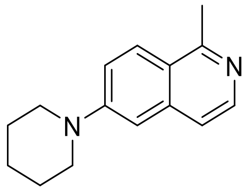

1-Methyl-6-(piperidin-1-yl)isoquinoline (7c).

7c was synthesised via method C from **6b** (882 mg, 4.0 mmol) and the product obtained after workup was used directly for the next step without further purification.

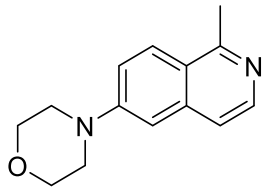

4-(1-Methylisoquinolin-6-yl)morpholine (7d).

7d was synthesized *via* method C from **6b** (882 mg, 4.0 mmol) and obtained as a yellow solid in 70 % yield (642 mg, 2.8 mmol). R_f = 0.25 (*n*-pentane: ethyl acetate, 1:1).

^{1}H NMR (400 MHz, $CDCl_3$) δ 8.25 (d, *J* = 5.8 Hz, 1H), 8.08 – 7.90 (m, 1H), 7.34 (d, *J* = 5.9 Hz, 1H), 7.30 (dd, *J* = 9.3, 2.6 Hz, 1H), 6.98 (d, *J* = 2.6 Hz, 1H), 3.98 – 3.83 (m, 4H), 3.42 – 3.25 (m, 4H), 2.89 (s, 3H).

^{13}C NMR (101 MHz, $CDCl_3$) δ 157.7, 151.8, 142.1, 137.8, 127.0, 122.3, 118.6, 118.5, 107.8, 66.7, 48.5, 22.0.

HRMS (ESI Pos): for [C14H17N2O]+ calcd. m/z = 229.1341, found m/z = 229.1335.

IR (ATR): $\tilde{\nu}$ [cm^{-1}] = 3249, 2853, 2816, 1580, 1495, 1449, 1416, 1382, 1296, 1235, 1112, 941, 854, 755, 696.

M_p 94–96 °C

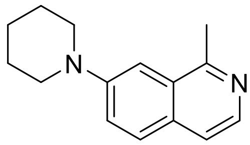

1-Methyl-7-(piperidin-1-yl)isoquinoline (7e).

7e was synthesized *via* method C from **6c** (882 mg, 4.0 mmol) and obtained as an orange oil in 85 % yield (772 mg, 3.4 mmol). R_f = 0.18 (*n*-pentane: ethyl acetate, 1:1).

^{1}H NMR (400 MHz, $CDCl_3$) δ 8.21 (d, *J* = 5.7 Hz, 1H), 7.67 (d, *J* = 9.0 Hz, 1H), 7.47 (dd, *J* = 9.1, 2.5 Hz, 1H), 7.39 (d, *J* = 5.7 Hz, 1H), 7.26 (s, 1H), 3.45 – 3.15 (m, 4H), 2.90 (s, 3H), 1.82 – 1.74 (m, 4H), 1.70 – 1.59 (m, 2H).

^{13}C NMR (101 MHz, $CDCl_3$) δ 156.6, 151.9, 139.0, 130.4, 128.8, 127.9, 123.9, 119.0, 107.4, 50.7, 25.8, 24.3, 22.4.

HRMS (ESI Pos): for [C15H19N2]+ calcd. m/z = 227.1548, found m/z = 227.1543.

IR (ATR): $\tilde{\nu}$ [cm^{-1}] = 2936, 1619, 1556, 1446, 1416, 1384, 1244, 943, 838, 552.

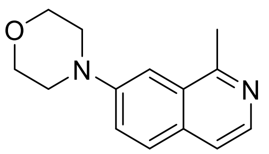

4-(1-Methylisoquinolin-7-yl)morpholine (7f).

7f was synthesized *via* method C from **6c** (882 mg, 4.0 mmol) and obtained as a yellow oil in 92 % yield (843 mg, 3.7 mmol). R_f = 0.15 (*n*-pentane: ethyl acetate, 1:1).

^{1}H NMR (400 MHz, $CDCl_3$) δ 8.25 (dd, *J* = 5.7, 1.1 Hz, 1H), 7.71 (dd, *J* = 9.1, 1.2 Hz, 1H), 7.50 – 7.38 (m, 2H), 7.28 – 7.21 (m, 1H), 3.93 (ddd, *J* = 5.9, 3.4, 0.9 Hz, 4H), 3.33 – 3.26 (m, 4H), 2.90 (d, *J* = 0.9 Hz, 3H).

^{13}C NMR (101 MHz, $CDCl_3$) δ 156.8, 150.0, 139.4, 130.9, 128.6, 128.3, 122.7, 119.0, 107.2, 66.9, 49.5, 22.4.

HRMS (ESI Pos): for [C14H17N2O]+ calcd. m/z = 229.1341, found m/z = 229.1335.

IR (ATR): $\tilde{\nu}$ [cm^{-1}] = 2835, 1625, 1593, 1557, 1512, 1452, 1308, 1268, 1229, 1120, 955, 840.

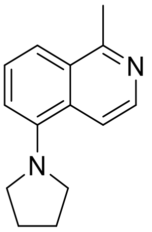

1-Methyl-5-(pyrrolidin-1-yl)isoquinoline (7g).

7g was synthesized via method C from **6a** (882 mg, 4.0 mmol) and the product obtained after workup was used directly for the next step without further purification.

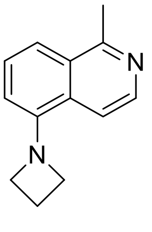

5-(Azetidin-1-yl)-1-methylisoquinoline (7h).

7h was synthesized via method C from **6a** (882 mg, 4.0 mmol) and the product obtained after workup was used directly for the next step without further purification.

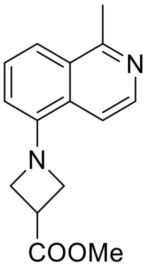

Methyl 1-(1-methylisoquinolin-5-yl)azetidine-3-carboxylate (7i).

7i was synthesised via method C from **6a** (882 mg, 4.0 mmol) and the product obtained after workup was used directly for the next step without further purification.

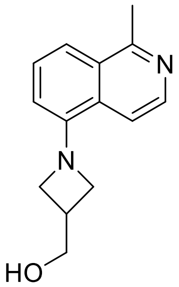

(1-(1-Methylisoquinolin-5-yl)azetidin-3-yl)methanol (7j).

7j was synthesized *via* method C from **6a** (882 mg, 4.0 mmol) and obtained as a yellow solid in 79 % yield (724 mg, 3.2 mmol). R_f = 0.35 (*n*-pentane: ethyl acetate, 1:3).

^{1}H NMR (400 MHz, $CDCl_3$) δ 8.24 (d, *J* = 6.0 Hz, 1H), 7.62 – 7.51 (m, 2H), 7.45 (t, *J* = 8.0 Hz, 1H), 6.67 (dd, *J* = 7.7, 1.0 Hz, 1H), 4.25 (t, *J* = 7.7 Hz, 2H), 4.02 – 3.91 (m, 4H), 3.03 – 2.92 (m, 1H), 2.92 (s, 3H).

^{13}C NMR (101 MHz, $CDCl_3$) δ 158.7, 148.0, 139.4, 128.5, 127.2, 127.2, 116.1, 115.4, 111.2, 77. 3, 77.2, 77.0, 76.7, 64.6, 57.1, 32.3, 22.9.

HRMS (ESI Pos): for [C14H17N2O]+ calcd. m/z = 229.1341, found m/z = 229.1335.

IR (ATR): $\tilde{\nu}$ [cm^{-1}] = 2839, 1625, 1598, 1547, 1510, 1453, 1268, 1120, 955, 844.

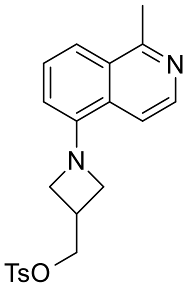

(1-(1-Methylisoquinolin-5-yl)azetidin-3-yl)methyl 4-methylbenzenesulfonate (7k).

7j (1.0 equiv.) was dissolved in CH_2Cl_2 (0.5 M), added to a 100 mL round bottom flask, and cooled to 0 °C. Then, 4-toluenesulfonyl chloride (TsCl, 1.5 equiv.) and triethylamine (NEt_3, 1.5 equiv.) was added at 0 °C. The reaction mixture was left stirring at 0 °C for 2 h and then, was slowly allowed to warm to room temperature and stirred for 10 h. After the full conversion, the solvent was removed under reduced pressure and the reaction mixture was redissolved by 50 mL CH_2Cl_2 and then subsequently washed with water and then brine, dried over anhydrous sodium sulfate, filtered, and concentrated under reduced pressure to afford the desired **7k** as a black solid, the crude product obtained after workup was used directly for the next step without further purification.

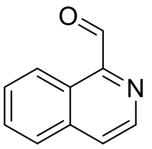

Isoquinoline-1-carbaldehyde (3).

3 was synthesized *via* method D from 1-methylisoquinolin (572 mg, 4.0 mmol) and obtained as a pale red solid in 77 % yield (484 mg, 3.1 mmol). R_f = 0.68 (*n*-pentane: ethyl acetate, 9:1).

The ^{1}H-NMR was in agreement with published data.[6]

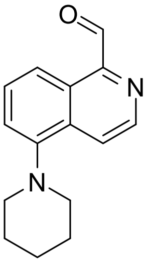

5-(Piperidin-1-yl)isoquinoline-1-carbaldehyde (3a).

3a was synthesized *via* method D from **7a** (452 mg, 2.0 mmol) and obtained as a yellow solid in 69 % yield (333 mg, 1.4 mmol). R_f = 0.54 (*n*-pentane: ethyl acetate, 9:1).

^{1}H NMR (400 MHz, $CDCl_3$) δ 10.43 (s, 1H), 9.01 (dt, *J* = 8.6, 1.0 Hz, 1H), 8.79 (d, *J* = 5.7 Hz, 1H), 8.28 (dd, *J* = 5.6, 0.9 Hz, 1H), 7.69 (dd, *J* = 8.6, 7.5 Hz, 1H), 7.33 (dd, *J* = 7.5, 1.0 Hz, 1H), 3.09 (s, 4H), 1.90 (p, *J* = 5.6 Hz, 4H), 1.72 (s, 2H).

^{13}C NMR (101 MHz, $CDCl_3$) δ 195.7, 150.5, 150.1, 141.9, 133.5, 130.2, 127.7, 121.8, 119.8, 118.8, 54.7, 26.5, 24.4.

HRMS (ESI Pos): for [C15H16N2O]+ calcd. m/z = 241.1341, found m/z = 241.1335.

IR (ATR): $\tilde{\nu}$ [cm^{-1}] = 2943, 2810, 1701, 1567, 1453, 1224, 1133, 1026, 888, 759.

M_p 86–88 °C

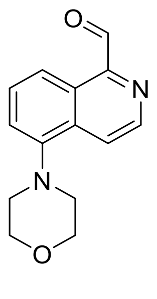

5-Morpholinoisoquinoline-1-carbaldehyde (3b).

3b was synthesized *via* method D from **7b** (456 mg, 2.0 mmol) and obtained as a red solid in 88 % yield (358 mg, 1.5 mmol). R_f = 0.51 (*n*-pentane: ethyl acetate, 9:1).

^{1}H NMR (400 MHz, $CDCl_3$) δ 10.38 (s, 1H), 9.04 (dt, *J* = 8.6, 1.0 Hz, 1H), 8.76 (d, *J* = 5.7 Hz, 1H), 8.27 (dd, *J* = 5.6, 0.9 Hz, 1H), 7.68 (dd, *J* = 8.7, 7.5 Hz, 1H), 7.35 (dd, *J* = 7.5, 1.0 Hz, 1H), 4.03 – 3.96 (m, 4H), 3.14 – 3.08 (m, 4H).

^{13}C NMR (101 MHz, $CDCl_3$) δ 195.6, 150.2, 148.9, 142.2, 133.1, 130.2, 127.7, 121.3, 120.9, 119.2, 67.3, 53.5.

HRMS (ESI Pos): for [C14H15N2O2]+ calcd. m/z = 243.1134, found m/z = 243.1128.

IR (ATR): $\tilde{\nu}$ [cm^{-1}] = 2817, 1702, 1448, 1263, 1226, 1169, 1111, 1027, 959, 887.

M_p 78–80 °C

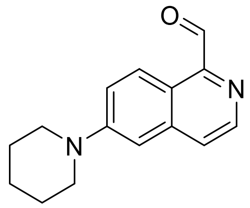

6-(Piperidin-1-yl)isoquinoline-1-carbaldehyde (3c).

3c was synthesized *via* method D from **7c** (452 mg, 2.0 mmol) and obtained as a yellow solid in 79 % yield (379 mg, 1.6 mmol). R_f = 0.48 (*n*-pentane: ethyl acetate, 9:1).

^{1}H NMR (400 MHz, $CDCl_3$) δ 10.30 (s, 1H), 9.10 (dd, *J* = 9.6, 0.8 Hz, 1H), 8.54 (d, *J* = 5.6 Hz, 1H), 7.61 (dd, *J* = 5.6, 0.8 Hz, 1H), 7.45 (dd, *J* = 9.6, 2.6 Hz, 1H), 6.98 (d, *J* = 2.6 Hz, 1H), 3.52 – 3.27 (m, 4H), 1.78 – 1.71 (m, 4H), 1.70 – 1.63 (m, 2H).

^{13}C NMR (101 MHz, $CDCl_3$) δ 196.3, 152.2, 149.1, 142.8, 139.4, 126.6, 124.0, 122.1, 120.6, 106.1, 49.1, 25.5, 24.5, 24.4.

HRMS (ESI Pos): for [C15H16N2O]+ calcd. m/z = 241.1341, found m/z = 241.1335.

IR (ATR): $\tilde{\nu}$ [cm^{-1}] = 2815, 1702, 1612, 1495, 1248, 1119, 1038, 966, 873, 852.

M_p 80–82 °C.

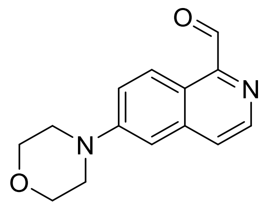

6-Morpholinoisoquinoline-1-carbaldehyde (3d).

3d was synthesized via method D from **7d** (456 mg, 2.0 mmol) and obtained as a green solid in 70 % yield (339 mg, 2.8 mmol). R_f = 0.48 (*n*-pentane: ethyl acetate, 9:1).

^{1}H NMR (400 MHz, $CDCl_3$) δ 10.32 (s, 1H), 9.17 (d, *J* = 9.5 Hz, 1H), 8.60 (d, *J* = 5.6 Hz, 1H), 7.67 (d, *J* = 5.6 Hz, 1H), 7.45 (dd, *J* = 9.6, 2.6 Hz, 1H), 7.02 (d, *J* = 2.6 Hz, 1H), 3.94 – 3.89 (m, 4H), 3.42 – 3.35 (m, 4H).

^{13}C NMR (101 MHz, $CDCl_3$) δ 196.1, 151.9, 149.2, 142.9, 139.1, 126.9, 124.2, 121.4, 121.2, 106.6, 66.6, 48.1.

HRMS (ESI Pos): for [C14H15N2O2]+ calcd. m/z = 243.1134, found m/z = 243.1128.

IR (ATR): $\tilde{\nu}$ [cm^{-1}] = 2817, 1703, 1572, 1449, 1263, 1226, 959, 888, 853, 817.

M_p 84–86 °C.

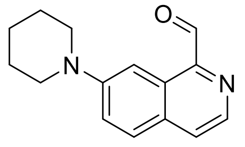

7-(Piperidin-1-yl)isoquinoline-1-carbaldehyde (3e).

3e was synthesized *via* method D from **7e** (452 mg, 2.0 mmol) and obtained as a yellow solid in 82 % yield (394 mg, 1.6 mmol). R_f = 0.59 (*n*-pentane: ethyl acetate, 9:1).

^{1}H NMR (400 MHz, $CDCl_3$) δ 10.36 (s, 1H), 8.63 (d, *J* = 2.5 Hz, 1H), 8.53 (d, *J* = 5.3 Hz, 1H), 7.77 – 7.68 (m, 2H), 7.53 (dd, *J* = 9.2, 2.6 Hz, 1H), 3.46 – 3.39 (m, 4H), 1.81 – 1.71 (m, 4H), 1.67 (tdd, *J* = 9.3, 5.1, 2.1 Hz, 2H).

^{13}C NMR (101 MHz, $CDCl_3$) δ 196.3, 152.8, 147.4, 139.8, 131.6, 128.7, 127.8, 125.2, 123.5, 105.4, 49.7, 25.6, 24.3.

HRMS (ESI Pos): for [C15H16N2O]+ calcd. m/z = 241.1341, found m/z = 241.1335.

IR (ATR): $\tilde{\nu}$ [cm^{-1}] = 2807, 2360, 1708, 1615, 1550, 1112, 954, 869, 798.

M_p 72–74 °C.

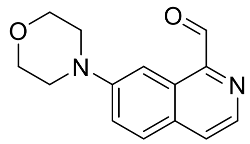

3f was synthesized *via* method D from **7f** (456 mg, 4.0 mmol) and obtained as a yellow solid in 87 % yield (421 mg, 1.7 mmol). R_f = 0.53 (*n*-pentane: ethyl acetate, 9:1).

^{1}H NMR (400 MHz, $CDCl_3$) δ 10.37 (s, 1H), 8.67 (d, *J* = 2.5 Hz, 1H), 8.59 (d, *J* = 5.3 Hz, 1H), 7.80 (d, *J* = 9.1 Hz, 1H), 7.75 (dd, *J* = 5.3, 0.8 Hz, 1H), 7.52 (dd, *J* = 9.2, 2.6 Hz, 1H), 3.96 – 3.89 (m, 4H), 3.44 – 3.37 (m, 4H).

^{13}C NMR (101 MHz, $CDCl_3$) δ 196.3, 152.2, 147.8, 140.3, 132.1, 128.4, 128.0, 125.2, 122.5, 105.8, 66.7, 48.5.

HRMS (ESI Pos): for [C14H15N2O2]+ calcd. m/z = 243.1134, found m/z = 243.1128.

IR (ATR): $\tilde{\nu}$ [cm^{-1}] = 2807, 2360, 1693, 1613, 1447, 1208, 1123, 849, 774.

M_p 72–74 °C.

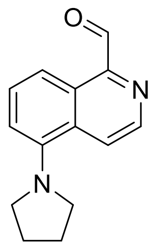

5-(Pyrrolidin-1-yl)isoquinoline-1-carbaldehyde (3g).

3g was synthesized *via* method B from **6** and obtained as an orange solid in 49 % yield (427 mg, 2.0 mmol, over two steps). R_f = 0.25 (*n*-pentane: ethyl acetate, 1:1).

^{1}H NMR (400 MHz, $CDCl_3$) δ 10.38 (s, 1H), 8.84 (d, *J* = 8.4 Hz, 1H), 8.66 (d, *J* = 5.7 Hz, 1H), 8.26 (d, *J* = 5.9 Hz, 1H), 7.60 – 7.56 (m, 0H), 7.12 (d, *J* = 7.7 Hz, 1H), 3.48 – 3.41 (m, 4H), 2.12 – 1.99 (m, *J* = 6.4, 5.3 Hz, 4H).

^{13}C NMR (101 MHz, $CDCl_3$) δ 195.7, 149.9, 140.6, 131.5, 130.4, 128.1, 122.9, 116.9, 114.4, 53.0, 25.3

HRMS (ESI Pos): for [C13H13N2O]+ calcd. m/z = 227.1184, found m/z = 227.1178.

IR (ATR): $\tilde{\nu}$ [cm^{-1}] = 2817, 1706, 1575, 1491, 959, 889, 852, 818.

M_p 78–80°C.

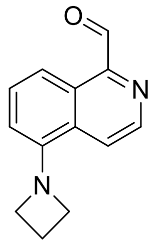

5-(Azetidin-1-yl)isoquinoline-1-carbaldehyde (3h).

3h was synthesized *via* method B from **5** (882 mg, 4.0 mmol) and obtained as a orange solid in 53 % yield (463 mg, 2.1 mmol, over two steps). R_f = 0.29 (*n*-pentane: ethyl acetate, 1:1).

^{1}H NMR (400 MHz, $CDCl_3$) δ 10.40 (s, 1H), 8.80 (dt, *J* = 8.5, 1.0 Hz, 1H), 8.68 (d, *J* = 5.7 Hz, 1H), 8.03 (dd, *J* = 5.7, 1.0 Hz, 1H), 7.64 (dd, *J* = 8.5, 7.7 Hz, 1H), 6.76 (dd, *J* = 7.8, 0.9 Hz, 1H), 4.26 (t, *J* = 7.3 Hz, 4H), 2.58 – 2.46 (m, 2H).

^{13}C NMR (101 MHz, $CDCl_3$) δ 195.7, 149.9, 148.1, 140.6, 130.5, 128.6, 127.7, 121.7, 115.5, 111.3, 55.4, 17.5.

HRMS (ESI Pos): for [C13H13N2O]+ calcd. m/z = 213.1028, found m/z = 213.1022.

IR (ATR): $\tilde{\nu}$ [cm-1] = 2838, 2796, 1383, 1225, 1132, 941, 750.

M_p: 84–86 °C.

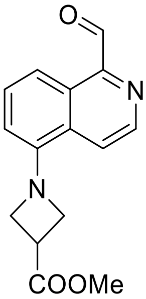

Methyl 1-(1-formylisoquinolin-5-yl)azetidine-3-carboxylate (3i).

3i was synthesized *via* method B from **5** (882 mg, 4.0 mmol) and obtained as an orange solid in 53 % yield (463 mg, 2.1 mmol, over two steps). R_f = 0.29 (*n*-pentane: ethyl acetate, 1:1).

^{1}H NMR (400 MHz, CDCl3) δ 10.35 (s, 1H), 8.85 – 8.78 (m, 1H), 8.66 (d, J = 5.8 Hz, 1H), 7.94 (dd, J = 5.9, 1.0 Hz, 1H), 7.61 (dd, J = 8.5, 7.7 Hz, 1H), 6.77 (dd, J = 7.7, 0.9 Hz, 1H), 4.44 – 4.31 (m, 5H), 3.79 (d, J = 1.7 Hz, 1H), 3.79 (s, 3H), 3.66 (tt, J = 8.5, 6.2 Hz, 1H).

^{13}C NMR (101 MHz, CDCl3) δ 195.5, 172.9, 150.0, 147.1, 140.9, 130.3, 128.6, 127.5, 121.2, 116.5, 111. 9, 77.3, 77.2, 77.0, 76.7, 57.0, 52.4, 33.8.

HRMS (ESI Pos): for [C15H15N2O3]+ calcd. m/z = 271.1083, found m/z = 271.1077.

IR (ATR): $\tilde{\nu}$ [cm-1] = 3895, 2848, 2796, 1580, 1493, 1415, 1383, 1233, 936, 747.

M_p: 68–70 °C.

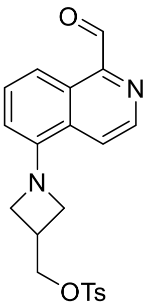

(1-(1-Formylisoquinolin-5-yl)azetidin-3-yl)methyl 4-methylbenzenesulfonate (3j).

3i was synthesized *via* method B from **6** and obtained as an red solid in 49 % yield (427 mg, 2.0 mmol, over two steps). R_f = 0.25 (*n*-pentane: ethyl acetate, 1:1).

^{1}H NMR (400 MHz, $CDCl_3$) δ 10.37 – 10.32 (m, 1H), 8.83 – 8.75 (m, 1H), 8.66 – 8.59 (m, 1H), 7.83 (dd, *J* = 13.6, 6.7 Hz, 3H), 7.63 – 7.53 (m, 1H), 7.36 (t, *J* = 6.5 Hz, 2H), 7.27 (s, 0H), 6.72 – 6.64 (m, 1H), 4.37 – 4.30 (m, 2H), 4.23 (q, *J* = 6.8 Hz, 2H), 3.93 – 3.85 (m, 2H), 3.16 – 3.06 (m, 1H), 2.48 – 2.43 (m, 3H), 1.25 (s, 1H).

^{13}C NMR (101 MHz, $CDCl_3$) δ 195.5, 150.0, 147.1, 145.2, 140.8, 132.8, 130.3, 130.0, 128.5, 127.5, 121.2, 116.2, 111.6, 77.0, 71.2, 56.6, 29.7, 21.7.

HRMS (ESI Pos): for [C21H21N2O4S]+ calcd. m/z = 397.1222, found m/z = 397.1216.

IR (ATR): $\tilde{\nu}$ [cm^{-1}] = 2827, 1702, 1548, 1463, 1226, 1113, 1027, 959, 887.

M_p 58–62°C.

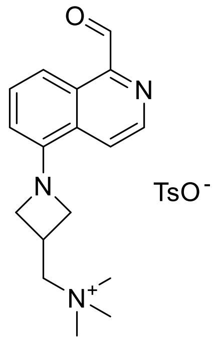

1-(1-(1-Formylisoquinolin-5-yl)azetidin-3-yl)-N,N,N-trimethylmethanaminium 4-methylbenzenesulfonate (3l).

7k (1.0 equiv.) was dissolved in 2M trimethylamine in THF, added to a 100 mL round bottom flask, and heated to 70 °C. The reaction mixture was left stirring at 70 °C for 24 h. After the full conversion, the solvent was removed under reduced pressure and the remaining mixture was purified by automated reverse phase column chromatography (C18, 5 to 95% of ACN+0.1% AcOH in H_2O+0.1% AcOH) to afford the desired **3l** as a red solid**.**

^{1}H NMR (400 MHz, DMSO) δ 10.25 (s, 1H), 8.71 (d, *J* = 5.7 Hz, 1H), 8.62 (d, *J* = 8.4 Hz, 1H), 8.12 (d, *J* = 5.8 Hz, 1H), 7.69 (t, *J* = 8.1 Hz, 1H), 6.89 – 6.81 (m, 1H), 4.49 (t, *J* = 7.8 Hz, 2H), 3.97 (q, *J* = 6.6 Hz, 2H), 3.71 (d, *J* = 6.7 Hz, 3H), 3.48 (q, *J* = 8.4 Hz, 2H), 2.31 (s, 6H).

^{13}C NMR (101 MHz, DMSO) δ 196.0, 149.8, 148.0, 141.3, 131.1, 128.1, 127.2, 122.1, 114.5, 112.0, 68.2, 59.1, 54.9, 54.8, 54.8, 52.9, 52.8, 52.8, 42.8, 40.6, 40.5, 40.4, 40.3, 40.2, 40.0, 39.8, 39. 6, 39.4, 25.3, 23.6.

HRMS (ESI Pos): for [C13H12N2O]+ calcd. m/z = 284.1757, found m/z = 284.1751.

IR (ATR): $\tilde{\nu}$ [cm^{-1}] = 2819, 1721, 1580, 1449, 1263, 876, 849, 811.

M_p 60–62°C.

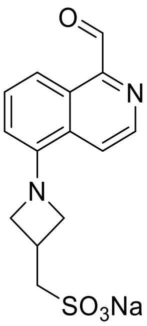

Sodium (1-(1-formylisoquinolin-5-yl)azetidin-3-yl)methanesulfonate (3m).

7k (1.0 equiv.) was added to a 25 mL round bottom flask and dissolved in EtOH: H_2O = 1:1 (v/v, 25 mM). Then, sodium sulfinate (2.0 equiv.) was added. The reaction mixture was left stirring at 70 °C for 24 h. After the full conversion, the solvent was removed under reduced pressure and the remaining mixture was purified by automated reverse phase column chromatography (C18, 5 to 95% of ACN+0.1% AcOH in H_2O+0.1% AcOH) to afford the **3m** as a red solid.

^{1}H NMR (400 MHz, DMSO) δ 10.23 (s, 1H), 8.66 (d, *J* = 5.7 Hz, 1H), 8.54 (d, *J* = 8.5 Hz, 1H), 8.12 (d, *J* = 5.9 Hz, 1H), 7.64 (t, *J* = 8.1 Hz, 2H), 6.73 (d, *J* = 7.8 Hz, 1H), 4.43 – 4.29 (m, 3H), 4.02 – 3.90 (m, 3H), 2.83 (dd, *J* = 7.6, 5.3 Hz, 4H), 2.35 (s, 9H).

^{13}C NMR (101 MHz, $CDCl_3$) δ 195.9, 148.9, 137.9, 130.4, 129.0, 128.2, 125.6, 120.4, 101.0, 47.9, 29.7, 25.6.

HRMS (ESI Neg): for [C14H13N2O4S]- calcd. m/z = 305.0602, found m/z = 305.0596.

IR (ATR): $\tilde{\nu}$ [cm^{-1}] = 2820, 1711, 1586, 1451, 1326, 901, 853, 829.

M_p 78–80°C.

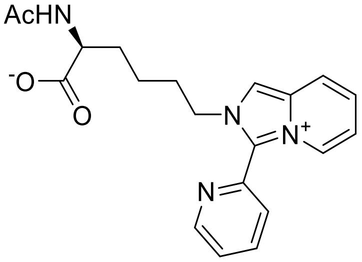

py-IP$^+$ was synthesized via method E from Ac-lys-OH (38 mg, 0.2 mmol) and obtained as a brown crystal in 93 % yield (68 mg, 0.19 mmol).

^{1}H NMR (400 MHz, MeOD) δ 8.97 (ddd, *J* = 4.8, 1.8, 0.9 Hz, 1H), 8.51 (dq, *J* = 7.4, 1.0 Hz, 1H), 8.36 (d, *J* = 0.9 Hz, 1H), 8.22 (td, *J* = 7.8, 1.8 Hz, 1H), 8.06 (dt, *J* = 7.9, 1.0 Hz, 1H), 7.92 (dt, *J* = 9.3, 1.2 Hz, 1H), 7.76 (ddd, *J* = 7.7, 4.9, 1.1 Hz, 1H), 7.38 (ddd, *J* = 9.3, 6.7, 0.9 Hz, 1H), 7.20 (ddd, *J* = 7.7, 6.7, 1.2 Hz, 1H), 4.65 – 4.52 (m, 2H), 4.21 (dd, *J* = 7.7, 5.1 Hz, 1H), 1.94 (d, *J* = 12.7 Hz, 5H), 1.79 (dtd, *J* = 13.2, 7.9, 5.1 Hz, 1H), 1.67 – 1.58 (m, 1H), 1.34 (p, *J* = 7.6 Hz, 2H).

^{13}C NMR (101 MHz, MeOD) δ 178.4, 172.4, 152.7, 142.6, 139.8, 133.4, 131.4, 128.2, 128.0, 126.7, 124.0, 119.7, 119.7, 116.0, 55.4, 51.2, 49.7, 33.1, 30.8, 23.3, 22.8.

HRMS (ESI Pos): for [C20H23N4O3]+ calcd. m/z = 367.1765, found m/z = 367.1765.

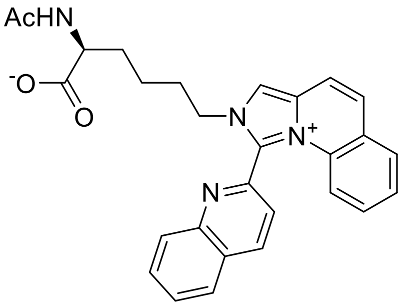

qu-IP⁺ was synthesized via method E from Ac-lys-OH (38 mg, 0.2 mmol) and obtained as a brown crystal in 71 % yield (66 mg, 0.14 mmol).

^{1}H NMR (400 MHz, MeOD) δ 8.83 (d, *J* = 8.4 Hz, 1H), 8.46 (s, 1H), 8.24 (dd, *J* = 8.5, 1.3 Hz, 2H), 8.08 (d, *J* = 8.4 Hz, 1H), 8.00 (dtd, *J* = 9.5, 7.4, 1.5 Hz, 2H), 7.91 (ddd, *J* = 8.2, 7.0, 1.2 Hz, 1H), 7.79 – 7.70 (m, 2H), 7.61 (td, *J* = 7.6, 1.0 Hz, 1H), 7.30 (ddd, *J* = 8.7, 7.3, 1.5 Hz, 1H), 6.96 (d, *J* = 8.5 Hz, 1H), 4.31 (d, *J* = 8.1 Hz, 2H), 4.19 (dd, *J* = 7.6, 5.2 Hz, 1H), 2.00 – 1.87 (m, 5H), 1.80 – 1.70 (m, 1H), 1.60 (dq, *J* = 13.5, 7.5 Hz, 1H), 1.38 – 1.26 (m, 2H).

^{13}C NMR (101 MHz, MeOD) δ 176.9, 171.0, 148.5, 143.7, 139.6, 135.9, 131.7, 130.3, 130.1, 129.8, 129.7, 129.5, 129.4, 129.1, 128.5, 128.3, 127.3, 126.1, 122.8, 117.3, 115.7, 114.9, 54.0, 49.5, 31.7, 29.3, 22.0, 21.4.

HRMS (ESI Pos): for [C28H27N4O3]+ calcd. m/z = 467.2078, found m/z = 467.2078.

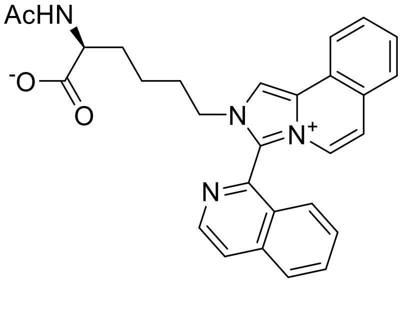

iq-IP⁺ was synthesized via method E from Ac-lys-OH (38 mg, 0.2 mmol) and obtained as a brown crystal in 91 % yield (85 mg, 0.18 mmol).

^{1}H NMR (400 MHz, MeOD) δ 9.04 (dd, *J* = 2.2, 0.9 Hz, 1H), 8.93 (dd, *J* = 5.6, 0.6 Hz, 1H), 8.52 – 8.44 (m, 1H), 8.30 – 8.22 (m, 2H), 8.01 – 7.93 (m, 1H), 7.93 – 7.70 (m, 5H), 7.67 (d, *J* = 7.5 Hz, 1H), 7.42 (d, *J* = 7.8 Hz, 1H), 4.50 (ddt, *J* = 14.2, 10.0, 7.3 Hz, 1H), 4.31 (dtd, *J* = 13.9, 7.3, 3.3 Hz, 1H), 4.16 (ddd, *J* = 7.6, 5.2, 2.7 Hz, 1H), 1.95 – 1.82 (m, 5H), 1.75 – 1.64 (m, 1H), 1.54 (dt, *J* = 13.5, 6.7 Hz, 1H), 1.34 – 1.19 (m, 2H).

^{13}C NMR (101 MHz, MeOD) δ 178.6, 172.4, 172.3, 144.7, 142.3, 138.7, 135.0, 133.4, 131.7, 131.6, 130.8, 129.8, 129.5, 129.1, 126.5, 125.4, 125.3, 125.1, 123.5, 121.3, 120.7, 116.6, 55.6, 55.5, 51.3, 33.1, 33.0, 30.7, 30. 6, 23.3, 23.2, 22.8, 22.8.

HRMS (ESI Pos): for [C28H27N4O3]+ calcd. m/z = 467.2078, found m/z = 467.2078.

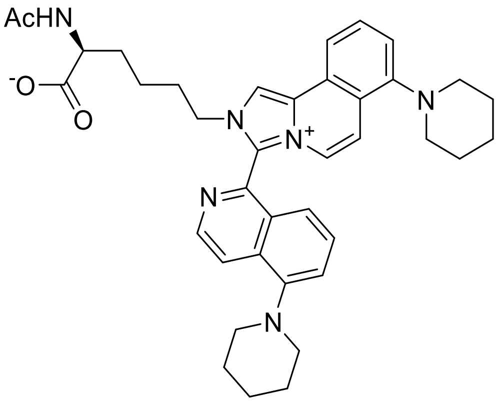

8a was synthesized via method E from Ac-lys-OH (38 mg, 0.2 mmol) and obtained as a yellow crystal in 83 % yield (105 mg, 0.17 mmol).

^{1}H NMR (400 MHz, MeOD) δ 8.97 (d, J = 2.2 Hz, 1H), 8.89 (dd, J = 5.7, 1.0 Hz, 1H), 8.46 (dt, J = 5.9, 1.1 Hz, 1H), 8.11 (d, J = 8.1 Hz, 1H), 7.80 – 7.55 (m, 4H), 7.53 – 7.38 (m, 2H), 7.32 – 7.22 (m, 1H), 4.62 – 4.23 (m, 2H), 4.16 (ddd, J = 7.6, 5.1, 2.7 Hz, 1H), 3.09 (d, J = 56.9 Hz, 8H), 1.95 – 1.51 (m, 20H) 1.32 – 1.16 (m, 2H).

^{13}C NMR (101 MHz, MeOD) δ 178.4, 172.4, 172.3, 153.5, 152.9, 144.1, 142.5, 135.1, 134.8, 132.1, 131.1, 124.6, 124.1, 122.6, 121.5, 121.1, 119.7, 119.3, 119.1, 119.0, 117.5, 116.5, 55.8, 55.6, 55.5, 51.2, 33.0, 30.7, 30.6, 27.5, 27.5, 25.4, 25.2, 23.3, 23.2, 22.8, 22.8.

HRMS (ESI Pos): for [C38H45N6O3]+ calcd. m/z = 633.3548, found m/z = 633.3548.

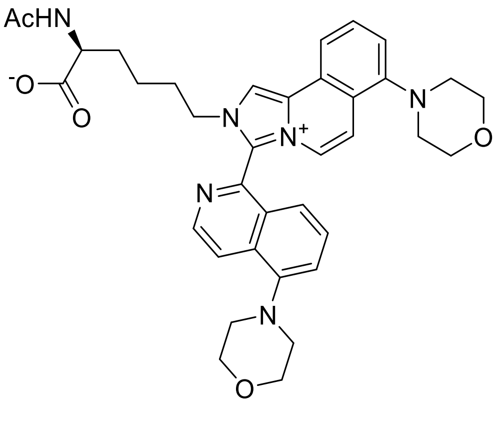

8b was synthesized via method E from Ac-Lys-OH (38 mg, 0.2 mmol) and obtained as a yellow crystal in 80 % yield (102 mg, 0.16 mmol).

^{1}H NMR (400 MHz, MeOD) δ 9.01 (d, *J* = 2.1 Hz, 1H), 8.92 (d, *J* = 5.7 Hz, 1H), 8.53 (dt, *J* = 5.7, 1.2 Hz, 1H), 8.18 (d, *J* = 7.9 Hz, 1H), 7.80 (t, *J* = 8.0 Hz, 1H), 7.76 – 7.70 (m, 2H), 7.64 (d, *J* = 7.8 Hz, 1H), 7.56 (dd, *J* = 7.3, 2.9 Hz, 1H), 7.51 (d, *J* = 7.9 Hz, 1H), 7.37 (t, *J* = 8.3 Hz, 1H), 4.49 (ddd, *J* = 14.1, 9.8, 7.2 Hz, 1H), 4.31 (dtd, *J* = 14.2, 7.1, 3.2 Hz, 1H), 4.16 (td, *J* = 5.1, 2.6 Hz, 1H), 4.06 – 4.01 (m, 4H), 3.93 – 3.88 (m, 4H), 3.21 (q, *J* = 5.1 Hz, 4H), 3.12 – 3.02 (m, 4H), 1.88 – 2.03 (m, 5H), 1.75 – 1.63 (m, 1H), 1.54 (dp, *J* = 14.7, 7.8 Hz, 1H), 1.32 – 1.20 (m, 2H).

^{13}C NMR (101 MHz, MeOD) δ 177.1, 171.0, 170.9, 150.6, 150.1, 143.0, 141.2, 133.7, 133.2, 130.9, 130.7, 129.7, 129.6, 123.4, 122.6, 121.1, 120.2, 120.0, 118.7, 118.7, 118.6, 118.5, 115.8, 115.3, 66.8, 66.7, 54.2, 54.1, 53.3, 53.2, 49.8, 31.6, 31.6, 29.3, 29.2, 21.9, 21.8, 21.5, 21.4.

HRMS (ESI Pos): for [C36H41N6O5]+ calcd. m/z = 637.3133, found m/z = 637. 3133.

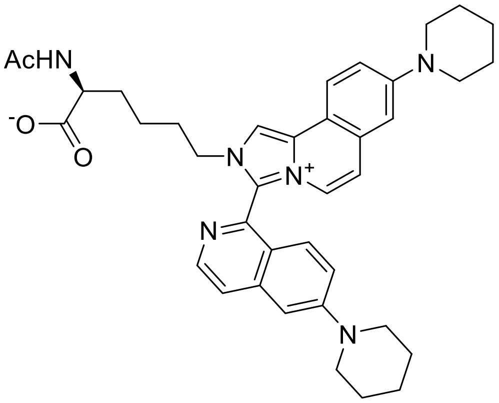

8c was synthesized via method E from Ac-Lys-OH (38 mg, 0.2 mmol) and obtained as a yellow crystal in 70 % yield (89 mg, 0.14 mmol).

^{1}H NMR (400 MHz, MeOD) δ 8.75 – 8.61 (m, 1H), 8.56 (dd, *J* = 5.8, 2.4 Hz, 1H), 8.22 (d, *J* = 8.4 Hz, 1H), 7.90 (dt, *J* = 5.9, 1.2 Hz, 1H), 7.63 – 7.37 (m, 4H), 7.32 – 7.21 (m, 3H), 4.52 – 4.21 (m, 2H), 4.21 – 4.12 (m, 1H), 3.54 (s, 4H), 3.43 (t, *J* = 5.4 Hz, 4H), 1.91 – 1.48 (m, 20H), 1.30 – 1.17 (m, 2H).

^{13}C NMR (101 MHz, MeOD) δ 177.0, 170.9, 153.0, 152.9, 143.0, 139.7, 133.0, 129.9, 129.4, 125.0, 124.9, 124.6, 122.9, 122.3, 121.9, 120.2, 119.0, 118.7, 112.2, 111.1, 105.6, 105.6, 54.2, 54.1, 49.6, 48.9, 48.3, 31.6, 29.2, 29.1, 25.2, 24.1, 24.0, 21.9, 21.4, 21.4.

HRMS (ESI Pos): for [C36H41N6O5]+ calcd. m/z = 633.3548, found m/z = 633.3548.

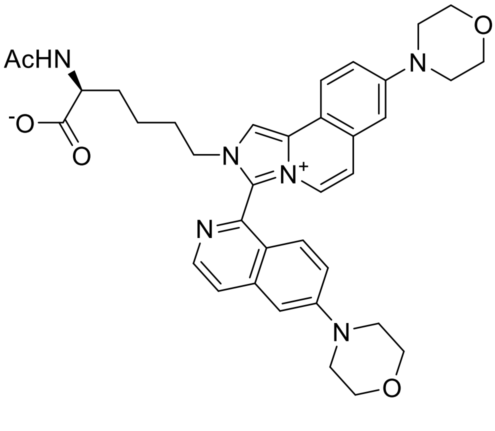

8d was synthesized via method E from Ac-Lys-OH (38 mg, 0.2 mmol) and obtained as a yellow crystal in 67 % yield (85 mg, 0.13 mmol).

^{1}H NMR (400 MHz, MeOD) δ 8.74 (d, *J* = 2.5 Hz, 1H), 8.63 (dd, *J* = 5.7, 1.9 Hz, 1H), 8.28 (d, *J* = 9.2 Hz, 1H), 7.97 (dd, *J* = 5.9, 1.2 Hz, 1H), 7.63 – 7.43 (m, 4H), 7.37 (d, *J* = 3.4 Hz, 1H), 7.32 – 7.26 (m, 2H), 4.44 (dq, *J* = 13.9, 6.6 Hz, 1H), 4.33 – 4.22 (m, 1H), 4.16 (ddd, *J* = 7.9, 5.1, 3.0 Hz, 1H), 3.92 – 3.79 (m, 8H), 3.47 (t, *J* = 4.8 Hz, 4H), 3.41 – 3.33 (m, 4H), 1.91 – 1.81 (m, 5H), 1.77 – 1.63 (m, 1H), 1.59 – 1.47 (m, 1H), 1.31 – 1.18 (m, 2H).

^{13}C NMR (101 MHz, MeOD) δ 176.8, 171.0, 153.0, 152.7, 143.2, 139.8, 139.5, 133.0, 129.7, 129.3, 125.1, 125.0, 124.7, 123.3, 123.0, 121.5, 120.1, 119.2, 118.3, 113.4, 112.7, 112.7, 111.1, 106.3, 66.4, 66.3, 54.0, 53.9, 49.6, 31.5, 29.2, 29.1, 21.9, 21.9, 21.4, 21.3.

HRMS (ESI Pos): for [C36H41N6O5]+ calcd. m/z = 637.3133, found m/z = 637. 3133.

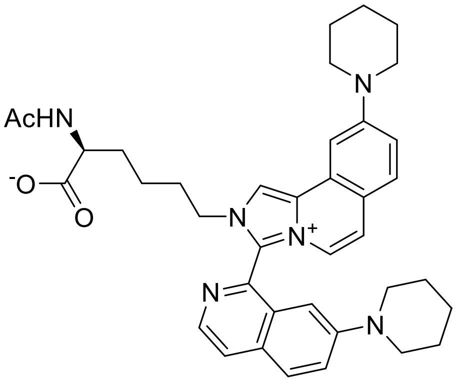

8e was synthesized via method E from Ac-Lys-OH (38 mg, 0.2 mmol) and obtained as a yellow crystal in 91 % yield (115 mg, 0.18 mmol).

^{1}H NMR (400 MHz, MeOD) δ 8.96 (d, *J* = 4.4 Hz, 1H), 8.60 (d, *J* = 5.4 Hz, 1H), 8.06 – 7.99 (m, 2H), 7.85 – 7.74 (m, 2H), 7.69 (dd, *J* = 8.9, 1.8 Hz, 1H), 7.44 – 7.33 (m, 2H), 7.25 (dd, *J* = 7.5, 2.1 Hz, 1H), 6.61 – 6.49 (m, 1H), 4.55 – 4.23 (m, 2H), 4.19 (dt, *J* = 7.6, 5.2 Hz, 1H), 3.51 (t, *J* = 5.2 Hz, 4H), 3.30 – 3.20 (m, 4H), 1.97 – 1.59 (m, 20H), 1.31 (p, *J* = 7.5 Hz, 2H).

^{13}C NMR (101 MHz, MeOD) δ 176.9, 171.0, 170.9, 152.7, 152.4, 140.0, 137.6, 133.6, 131.5, 130.4, 129.3, 129.0, 128.8, 124.7, 124.3, 123.6, 119.5, 118.7, 118.6, 115.5, 114.8, 106.4, 101.7, 54.0, 49.6, 49.6, 48.9, 31.7, 29.3, 29.1, 25.2, 25.1, 24.1, 23.8, 22.0, 22.0, 21.4, 20.3.

HRMS (ESI Pos): for [C36H41N6O5]+ calcd. m/z = 633.3548, found m/z = 633.3548.

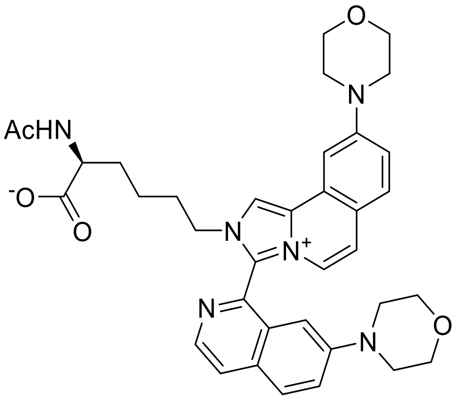

8f was synthesized via method E from Ac-Lys-OH (38 mg, 0.2 mmol) and obtained as a yellow crystal in 96 % yield (122 mg, 0.19 mmol).

^{1}H NMR (400 MHz, MeOD) δ 8.97 (d, *J* = 5.7 Hz, 1H), 8.65 (d, *J* = 5.4 Hz, 1H), 8.11 – 8.05 (m, 2H), 7.86 (s, 1H), 7.81 (dd, *J* = 9.3, 2.4 Hz, 1H), 7.75 (d, *J* = 8.8 Hz, 1H), 7.48 – 7.39 (m, 2H), 7.30 (d, *J* = 7.5 Hz, 1H), 6.66 (dd, *J* = 20.0, 2.6 Hz, 1H), 4.57 – 4.43 (m, 1H), 4.29 (dq, *J* = 13.5, 7.3 Hz, 1H), 4.22 – 4.15 (m, 1H), 3.92 (t, *J* = 4.8 Hz, 4H), 3.75 (t, *J* = 5.0 Hz, 4H), 3.45 (t, *J* = 4.8 Hz, 4H), 3.26 – 3.16 (m, 4H), 1.97 – 1.84 (m, 5H), 1.79 – 1.67 (m, 1H), 1.57 (dq, *J* = 13.6, 6.6 Hz, 1H), 1.31 (dd, *J* = 14.2, 7.0 Hz, 3H).

^{13}C NMR (101 MHz, MeOD) δ 176.9, 171.0, 170.9, 152.5, 152.2, 140.6, 138.1, 133.7, 132.0, 130.1, 129.4, 129.1, 124.4, 123.9, 123.5, 119.6, 119.4, 118.2, 116.1, 115.0, 106.6, 102.2, 66.4, 66.2, 54.1, 53.9, 49.6, 49.6, 47.9, 31.7, 31.6, 29.3, 29.1, 22.0, 21.9, 21.4, 21.4.

HRMS (ESI Pos): for [C36H41N6O5]+ calcd. m/z = 637.3133, found m/z = 637. 3133.

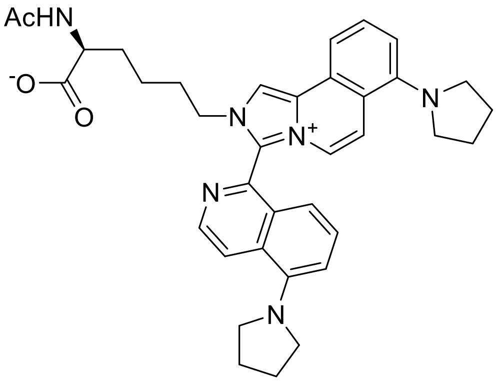

8g was synthesized via method E from Ac-Lys-OH (38 mg, 0.2 mmol) and obtained as a yellow crystal in 94 % yield (120 mg, 0.19 mmol).

^{1}H NMR (400 MHz, MeOD) δ 8.94 (d, *J* = 1.8 Hz, 1H), 8.78 (dd, *J* = 5.9, 1.0 Hz, 1H), 8.57 (dt, *J* = 5.9, 1.2 Hz, 1H), 7.92 (d, *J* = 7.8 Hz, 1H), 7.75 – 7.58 (m, 3H), 7.52 (d, *J* = 7.7 Hz, 1H), 7.31 – 7.20 (m, 2H), 7.02 (t, *J* = 7.6 Hz, 1H), 4.56 – 4.43 (m, 1H), 4.31 (dtd, *J* = 14.0, 7.2, 4.2 Hz, 1H), 4.19 (ddd, *J* = 7.7, 5.0, 2.8 Hz, 1H), 3.62 (tdd, *J* = 9.3, 6.9, 3.0 Hz, 5H), 3.42 (s, 3H), 3.42 (dtt, *J* = 13.0, 9.1, 4.4 Hz, 2H), 2.97 – 2.86 (m, 0H), 2.14 (td, *J* = 5.9, 3.1 Hz, 4H), 2.09 – 1.96 (m, 5H), 1.96 – 1.81 (m, 5H), 1.71 (dtt, *J* = 16.6, 8.6, 4.0 Hz, 1H), 1.56 (dp, *J* = 15.4, 8.0 Hz, 1H), 1.33 – 1.22 (m, 2H).

^{13}C NMR (101 MHz, MeOD) δ 176.9, 171.0, 171.0, 148.8, 148.0, 140.9, 140.7, 133.6, 130.9, 130.7, 130.6, 130.1, 129.7, 123.5, 122.6, 118.9, 117.3, 116.3, 116.2, 115.0, 114.8, 114.1, 113.6, 113.5, 54.0, 53.9, 52.5, 52.4, 31.8, 31.5, 29.3, 29.2, 25.1, 24.9, 21.9, 21.4.

HRMS (ESI Pos): for [C36H40N6O3]+ calcd. m/z = 604.3162, found m/z = 604.3162.

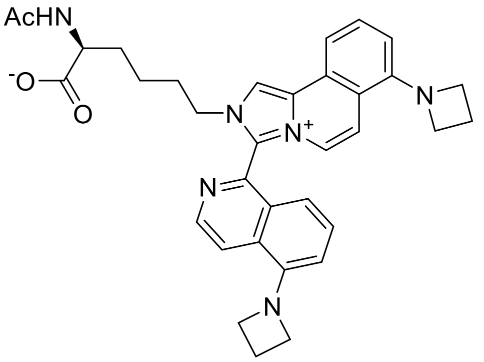

8h was synthesized via method E from Ac-Lys-OH (38 mg, 0.2 mmol) and obtained as a yellow crystal in 96 % yield (122 mg, 0.19 mmol).

^{1}H NMR (400 MHz, MeOD) δ 8.91 (d, *J* = 1.7 Hz, 1H), 8.76 (dd, *J* = 6.0, 1.0 Hz, 1H), 8.32 (dt, *J* = 5.9, 1.1 Hz, 1H), 7.82 (d, *J* = 7.8 Hz, 1H), 7.70 – 7.56 (m, 2H), 7.52 – 7.43 (m, 2H), 7.00 – 6.89 (m, 1H), 6.89 – 6.76 (m, 2H), 4.54 – 4.39 (m, 1H), 4.42 – 4.34 (m, 3H), 4.38 – 4.22 (m, 1H), 4.29 (s, 1H), 4.20 (qd, *J* = 7.2, 4.0 Hz, 5H), 2.62 – 2.39 (m, 4H), 1.98 (d, *J* = 15.1 Hz, 1H), 1.97 – 1.81 (m, 4H), 1.77 – 1.64 (m, 1H), 1.56 (dq, *J* = 14.9, 8.0 Hz, 1H), 1.29 (s, 1H), 1.33 – 1.21 (m, 1H).

^{13}C NMR (101 MHz, MeOD) δ 170.9, 149.7, 149.0, 141.1, 140.8, 133.5, 130.8, 130.5, 129.6, 128.2, 123.2, 121.3, 116.2, 116.0, 115.4, 115.0, 113.3, 113.3, 112.6, 112.6, 111.6, 55.1, 55.1, 54.1, 31.6, 29.3, 29.3, 21.9, 21.9, 21.4, 21.4, 16.9, 16.8.

HRMS (ESI Pos): for [C34H36N6O3]+ calcd. m/z = 576.2849, found m/z = 576.2849.

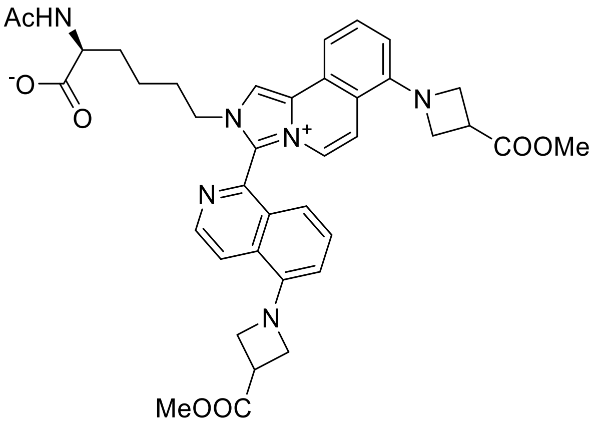

8i was synthesized via method E from Ac-lys-OH (38 mg, 0.2 mmol) and obtained as a brown crystal in 90 % yield (125 mg, 0.18 mmol).

^{1}H NMR (400 MHz, MeOD) δ 9.04 (dd, *J* = 2.2, 0.9 Hz, 1H), 8.93 (dd, *J* = 5.6, 0.6 Hz, 1H), 8.52 – 8.44 (m, 1H), 8.30 – 8.22 (m, 2H), 8.01 – 7.93 (m, 1H), 7.93 – 7.70 (m, 5H), 7.67 (d, *J* = 7.5 Hz, 1H), 7.42 (d, *J* = 7.8 Hz, 1H), 4.50 (ddt, *J* = 14.2, 10.0, 7.3 Hz, 1H), 4.31 (dtd, *J* = 13.9, 7.3, 3.3 Hz, 1H), 4.16 (ddd, *J* = 7.6, 5.2, 2.7 Hz, 1H), 1.95 – 1.82 (m, 5H), 1.75 – 1.64 (m, 1H), 1.54 (dt, *J* = 13.5, 6.7 Hz, 1H), 1.34 – 1.19 (m, 2H).

^{13}C NMR (101 MHz, MeOD) δ 178.6, 172.4, 172.3, 144.7, 142.3, 138.7, 135.0, 133.4, 131.7, 131.6, 130.8, 129.8, 129.5, 129.1, 126.5, 125.4, 125.3, 125.1, 123.5, 121.3, 120.7, 116.6, 55.6, 55.5, 51.3, 33.1, 33.0, 30.7, 30. 6, 23.3, 23.2, 22.8, 22.8.

HRMS (ESI Pos): for [C38H40N6O7]+ calcd. m/z = 692.2958, found m/z = 692.2958.

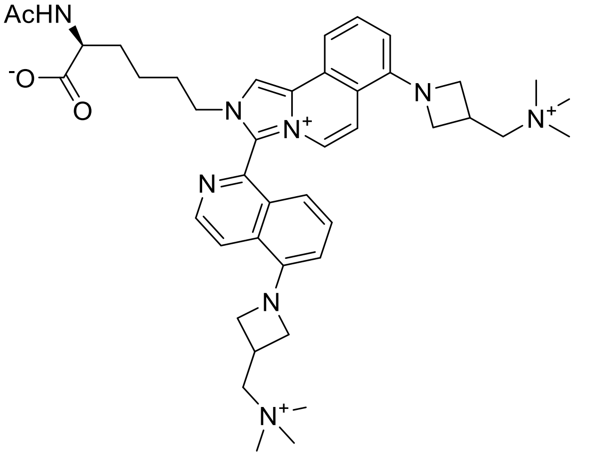

8l was synthesized via method E from Ac-lys-OH (38 mg, 0.2 mmol) and obtained as a black crystal in 91 % yield (85 mg, 0.18 mmol).

^{1}H NMR (400 MHz, MeOD) δ 9.04 (dd, *J* = 2.2, 0.9 Hz, 1H), 8.93 (dd, *J* = 5.6, 0.6 Hz, 1H), 8.52 – 8.44 (m, 1H), 8.30 – 8.22 (m, 2H), 8.01 – 7.93 (m, 1H), 7.93 – 7.70 (m, 5H), 7.67 (d, *J* = 7.5 Hz, 1H), 7.42 (d, *J* = 7.8 Hz, 1H), 4.50 (ddt, *J* = 14.2, 10.0, 7.3 Hz, 1H), 4.31 (dtd, *J* = 13.9, 7.3, 3.3 Hz, 1H), 4.16 (ddd, *J* = 7.6, 5.2, 2.7 Hz, 1H), 1.95 – 1.82 (m, 5H), 1.75 – 1.64 (m, 1H), 1.54 (dt, *J* = 13.5, 6.7 Hz, 1H), 1.34 – 1.19 (m, 2H).

^{13}C NMR (101 MHz, MeOD) δ 178.6, 172.4, 172.3, 144.7, 142.3, 138.7, 135.0, 133.4, 131.7, 131.6, 130.8, 129.8, 129.5, 129.1, 126.5, 125.4, 125.3, 125.1, 123.5, 121.3, 120.7, 116.6, 55.6, 55.5, 51.3, 33.1, 33.0, 30.7, 30. 6, 23.3, 23.2, 22.8, 22.8.

HRMS (ESI Pos): for [C38H40N6O7]$^{2+}$ calcd. m/z = 360.2234, found m/z = 360.2234.

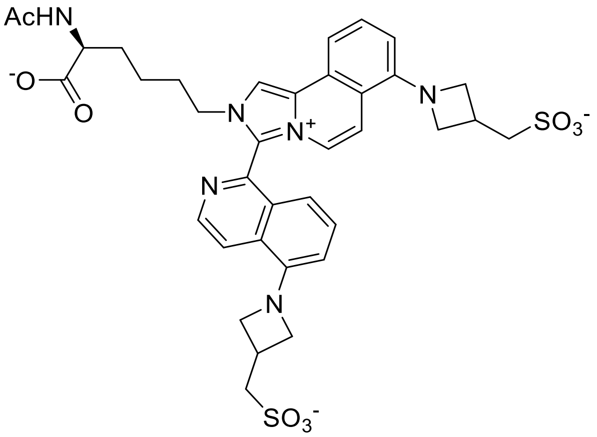

8m was synthesized via method E from Ac-lys-OH (38 mg, 0.2 mmol) and obtained as a brown crystal in 51 % yield (69 mg, 0.1 mmol).

^{1}H NMR (400 MHz, MeOD) δ 8.94 (d, *J* = 1.8 Hz, 1H), 8.78 (dd, *J* = 5.9, 1.0 Hz, 1H), 8.57 (dt, *J* = 5.9, 1.2 Hz, 1H), 7.92 (d, *J* = 7.8 Hz, 1H), 7.75 – 7.58 (m, 3H), 7.52 (d, *J* = 7.7 Hz, 1H), 7.31 – 7.20 (m, 2H), 7.02 (t, *J* = 7.6 Hz, 1H), 4.56 – 4.43 (m, 1H), 4.31 (dtd, *J* = 14.0, 7.2, 4.2 Hz, 1H), 4.19 (ddd, *J* = 7.7, 5.0, 2.8 Hz, 1H), 3.62 (tdd, *J* = 9.3, 6.9, 3.0 Hz, 5H), 3.42 (s, 3H), 3.42 (dtt, *J* = 13.0, 9.1, 4.4 Hz, 2H), 2.97 – 2.86 (m, 0H), 2.14 (td, *J* = 5.9, 3.1 Hz, 4H), 2.09 – 1.96 (m, 5H), 1.96 – 1.81 (m, 5H), 1.71 (dtt, *J* = 16.6, 8.6, 4.0 Hz, 1H), 1.56 (dp, *J* = 15.4, 8.0 Hz, 1H), 1.33 – 1.22 (m, 2H).

^{13}C NMR (101 MHz, MeOD) δ 170.9, 149.7, 149.0, 141.1, 140.8, 133.5, 130.8, 130.5, 129.6, 128.2, 123.2, 121.3, 116.2, 116.0, 115.4, 115.0, 113.3, 113.3, 112.6, 112.6, 111.6, 55.1, 55.1, 54.1, 31.6, 29.3, 29.3, 21.9, 21.9, 21.4, 21.4, 16.9, 16.8.

HRMS (ESI Pos): for $[C38H40N6O7]^{2-}$ calcd. m/z = 346.1479, found m/z = 346.1478.

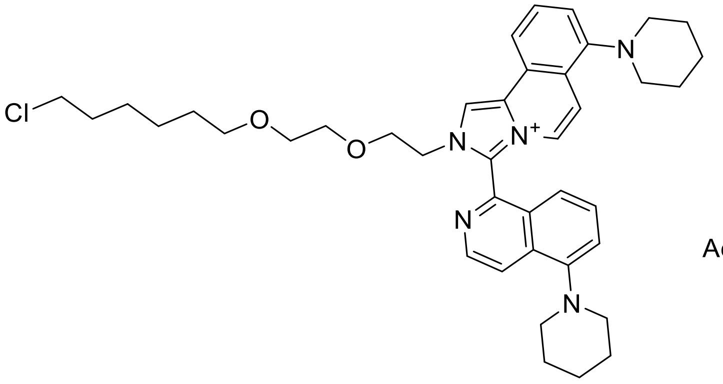

Halo-IP^{+} was synthesized *via* method E from HaloTag Amine (O2) (96 mg, 0.3 mmol) and obtained as a black crystal in 90 % yield (196 mg, 0.27 mmol).

^{1}H NMR (400 MHz, $CDCl_3$) δ 9.67 (s, 1H), 8.80 (d, *J* = 5.7 Hz, 1H), 8.31 (dd, *J* = 5.7, 0.9 Hz, 1H), 8.19 (d, *J* = 7.9 Hz, 1H), 7.70 (t, *J* = 7.9 Hz, 1H), 7.64 – 7.54 (m, 2H), 7.32 (ddd, *J* = 15.1, 7.5, 1.5 Hz, 3H), 7.18 (d, *J* = 8.5 Hz, 1H), 4.95 (ddd, *J* = 14.5, 8.0, 3.6 Hz, 1H), 4.70 – 4.60 (m, 1H), 3.98 (td, *J* = 8.1, 4.0 Hz, 1H), 3.89 – 3.80 (m, 1H), 3.47 (t, *J* = 6.7 Hz, 2H), 3.38 (td, *J* = 4.6, 1.5 Hz, 2H), 3.26 – 3.08 (m, 8H), 3.04 – 2.91 (m, 4H), 1.91 (p, *J* = 5.5 Hz, 4H), 1.77 – 1.64 (m, 6H), 1.40 – 1.13 (m, 9H), 0.91 – 0.78 (m, 1H).

^{13}C NMR (101 MHz, $CDCl_3$) δ 151.), 151.6, 142.8, 141.7, 133.9, 133.5, 131.6, 130.8, 129.7, 129.5, 123.7, 122.7, 122.3, 121.2, 120.3, 119.7, 119.3, 118.3, 117.6, 117.5, 116.7, 71.1, 70.3, 69.5, 69.0, 54.8, 50.2, 45.2, 32.6, 29.8, 29.5, 26.8, 26.6, 26.5, 25.4, 25,3, 24.3.

HRMS (ESI Pos): for [C40H51ClN5O2]+ calcd. m/z = 668.3726, found m/z = 668.3726.

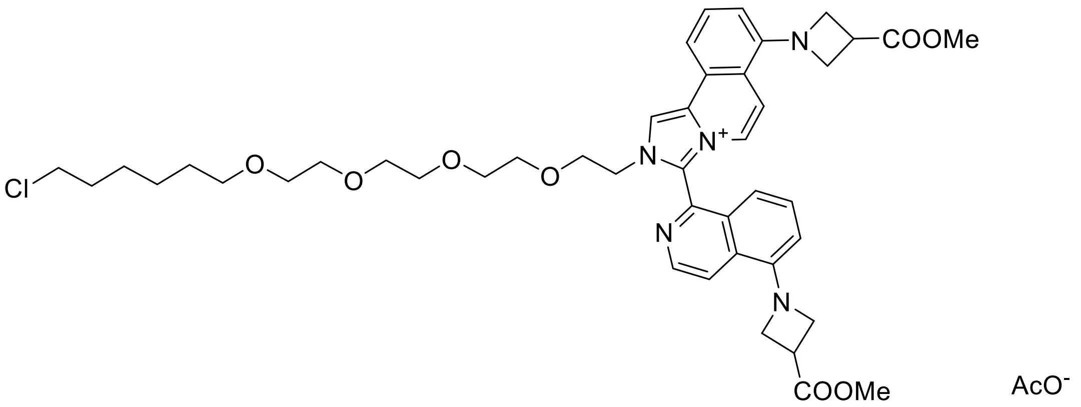

Halo-IP$^+$ was synthesized *via* method E from Halo-PEG(4)-NH2*HCl (10.0 mg, 0.2 mmol) and obtained as a black crystal in 75 % yield (129 mg, 0.15 mmol).

^{1}H NMR (400 MHz, MeOD) δ 8.93 (s, 1H), 8.79 (d, *J* = 5.8 Hz, 1H), 8.29 (d, *J* = 5.5 Hz, 1H), 7.87 (d, *J* = 7.6 Hz, 1H), 7.72 – 7.60 (m, 2H), 7.47 (q, *J* = 7.8 Hz, 2H), 7.01 (t, *J* = 7.6 Hz, 1H), 6.92 (dd, *J* = 14.4, 7.6 Hz, 2H), 4.58 (dt, *J* = 16.4, 8.1 Hz, 1H), 4.45 (t, *J* = 6.7 Hz, 2H), 4.40 (t, *J* = 8.1 Hz, 1H), 4.30 (q, *J* = 7.2 Hz, 3H), 4.18 (s, 1H), 3.84 – 3.74 (m, 5H), 3.72 – 3.64 (m, 1H), 2.93 (t, *J* = 7.4 Hz, 1H), 2.00 (s, 2H), 1.90 (d, *J* = 15.5 Hz, 5H), 1.70 (ddt, *J* = 13.9, 10.0, 7.2 Hz, 3H), 1.54 (s, 1H), 1.51 – 1.39 (m, 1H), 1.27 (dd, *J* = 17.4, 9.5 Hz, 3H).

^{13}C NMR (101 MHz, MeOD) δ 173.4, 173.4, 148.9, 148.3, 141.4, 130.8, 130.6, 129.6, 128.2, 121.1, 116.7, 115.8, 115.2, 114.1, 113.5, 112.1, 57.1, 56.8, 51.4, 51.4, 49.7, 48.2, 48.0, 47.8, 47.6, 47.4, 47.2, 47.0, 39.1, 33.6, 33.5, 31.8, 31.5, 26.6, 22.0, 21.4.

HRMS (ESI Pos): for [C44H55ClN5O8+]+ calcd. m/z = 816.3734, found m/z = 816.3734.

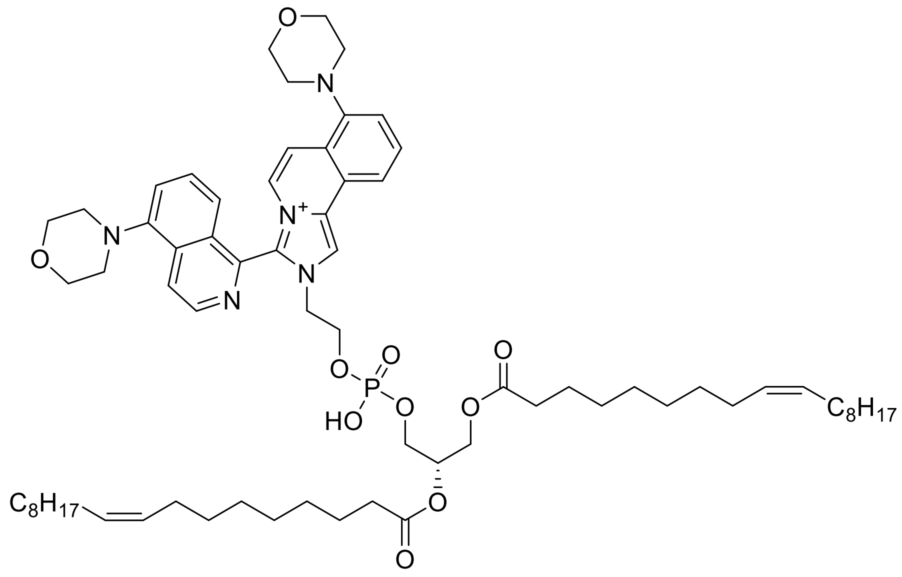

DOPE-IP+ was synthesized from DOPE (12.1 mg, 0.0163 mmol, 1 equiv.) and **3b** (21 mg, 0.087 mmol, 5.32 equiv.), which were dissolved in DCM (1.5 mL). AcOH (0.5 mL) was then added, and the mixture was heated to 40 °C for 4 hours. The volatile components were removed under reduced pressure and the remaining mixture was purified by silica gel column chromatography (stepwise gradient of MeOH in DCM, first 5% then 10% and lastly 12%) to receive **DOPE-IP+** as a yellow film in 52% yield (10.0 mg, 8.39 µmol).

^{1}H NMR (400 MHz, $CDCl_3$) δ 9.57 – 9.47 (m, 1H), 8.85 (d, *J* = 5.7 Hz, 1H), 8.38 (dd, *J* = 5.8, 0.9 Hz, 1H), 8.22 (d, *J* = 8.0 Hz, 1H), 7.77 – 7.65 (m, 2H), 7.58 (d, *J* = 7.7 Hz, 1H), 7.46 – 7.40 (m, 1H), 7.38 (dd, *J* = 8.5, 2.6 Hz, 1H), 7.35 – 7.27 (m, 2H), 5.39 – 5.25 (m, 4H), 5.20 – 5.10 (m, 1H), 4.93 – 4.79 (m, 1H), 4.66 – 4.56 (m, 1H), 4.39 – 4.26 (m, 2H), 4.21 – 3.96 (m, 8H), 3.88 (t, *J* = 4.5 Hz, 4H), 3.20 (td, *J* = 4.0, 2.0 Hz, 4H), 3.10 – 2.95 (m, 4H), 2.26 – 2.15 (m, 4H), 2.04 – 1.94 (m, 8H), 1.58 – 1.44 (m, 4H), 1.35 – 1.19 (m), 0.86 (t, 6H).

HRMS (ESI Pos): for [C69H103N5O10P]+ calcd. m/z = 1192.7437, found m/z = 1192.7437.

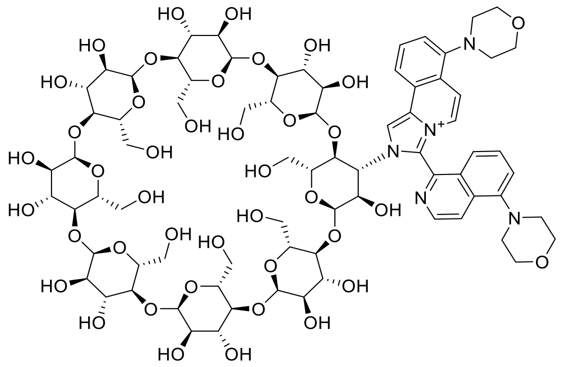

CD-IP+ was obtained by adding **3b** (24 mg, 0.10 mmol, 2.0 equiv.) o a solution of 3A-Amino-3A-deoxy-(2AS,3AS)-alpha-cyclodextrin hydrate (50 mg, 0.05 mmol, 1.0 equiv.) in 2 mL AcOH. The reaction mixture was stirred at room temperature for 24 h. Then the solvent was removed under reduced pressure and the remaining mixture was purified by automated reverse phase column chromatography (C18, 5 to 95% of ACN+0.1% AcOH in H_2O+0.1% AcOH) to afford the desired **CD-IP+** as a red poder in 27% yield (28 mg, 0.02 mmol).

HRMS (ESI Pos): for [C64H86N5O31]+ calcd. m/z = 1420.5301, found m/z = 1420.5301.

6. Peptide Synthesis and Labelling

SOP1 – Solid Phase Peptide Synthesis (50 µmol scale)
The non-preloaded resin (1.0 eq, 0.44 mmol/g, 50 µmol, 114 mg) was placed in the reaction vessel of the peptide synthesizer and was swollen for 30 min in DMF. First, the *N*-terminal Fmoc protecting group was removed by addition of piperidine (20 % in DMF, *v/v*, 2.5 mL). To achieve a complete cleavage of the Fmoc-group, the deprotection step was repeated twice. After washing of the resin with DMF (5 x 4.0 mL) the amino acid (4 eq, 0.2 M, 1.25 mL), DIC (0.5 M, 1.25 mL), and activator base (1 M, 1.25 mL) were added to the reaction vessel, and the coupling reaction was performed (see Table S1 in the General Information). For the coupling of Fmoc-Arg(Pbf)-OH, specific coupling cycles were used to suppress the possible γ-lactam formation, as reported in Table S1.
.

SOP 2 – Cleavage from resin
The cleavage of the peptides from the resin was performed in a BD syringe with a PE frit. The resin was shaken at room temperature in a TFA/Triisoppropylsilane (TIPS)/MilliQ solution (95:2.5:2.5 v/v/v).
For test cleavages, 10 mg of resin were treated with 1 mL of cleavage cocktail for 1.5 hrs. Afterwards, the solution was filtered into a 2 mL Eppendorf tube and reduced under nitrogen stream. The crude peptide was precipitated in Et_2O and centrifugated in a 1088 Palm Micro Centrifuge (centrifuging speed 6000 rpm). The supernatant was eliminated, and the peptide was redissolved into a 50:50 MeCN/MilliQ (+0.1% TFA) solution for ESI-MS and UHPLC analysis.
For full cleavage, the whole resin was treated with 4 mL of cleavage cocktail for 3 hrs. Afterwards, the solution was filtered into a 15 mL falcon tube and reduced under nitrogen stream. The crude peptide was precipitated in ice-cold Et_2O. The precipitate was isolated by centrifugation (9000 rpm, -10 °C, 10 min), washed twice with ether, redissolved in a 20:80 MeCN/MilliQ (+0.1% TFA) solution, and lyophilized.

Fmoc-GSG-ALFA-OH

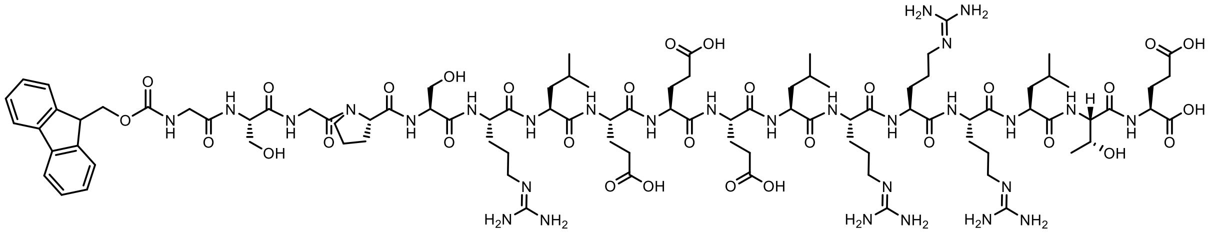

The synthesis of Fmoc-GSG-ALFA-NH_2 was carried out on a preloaded Fmoc-Glu-Wang resin (0.44 mmol/g loading) on a 50 µmol scale according to **SOP1**. DIC (0.50 M in DMF) and HOBt (0.50 M in DMF) were employed as activators, while deprotection was carried out with a 20% v/v solution of piperidine in DMF.

Table S3 – List of Fmoc-amino acids employed in the synthesis of Fmoc-GSG-ALFA-NH_2, with respective molecular weight, mass, and DMF volume required for preparing 0.2 M final solutions.

Amino Acid	Molecular Weight [g/mol]	Mass[g]	DMF Volume [mL]
Fmoc-Arg(Pbf)-OH	648.77	2.99	23
Fmoc-Glu(tBu)-OH	425.49	0.77	9
Fmoc-Gly-OH	297.31	0.72	12
Fmoc-Leu-OH	353.41	0.64	9
Fmoc-Pro-OH	337.37	0.41	6
Fmoc-Ser(tBu)-OH	383.44	0.93	12
Fmoc-Thr(tBu)-OH	397.48	0.24	3

After the synthesis, the resin was collected in a BD-Syringe and a test cleavage was performed according to **SOP2**. A small aliquot was re-dissolved in 1:1 MilliQ/MeCN for ESI-MS analysis.

HR-MS (ESI+) – m/z calculated for $C_{96}H_{151}N_{29}O_{31}$ ($[M+H]^+$): 2206.1131; found: m/z 2206.1; m/z calculated for $C_{96}H_{151}N_{29}O_{31}$ ($[M+H]^+$): 2206.1131; found: m/z 1984.0 ($[M+H-Fmoc]^+$); m/z calculated for $C_{96}H_{152}N_{29}O_{31}$ ($[M+2H]^{2+}$): 1104.0638, found: m/z 1104.0652; m/z calculated for $C_{96}H_{153}N_{29}O_{31}$

([m+3H]$^{3+}$): 736.3783, found: m/z 736.3789; m/z calculated for $C_{96}H_{154}N_{29}O_{31}$ ([M+4H]$^{4+}$): 552.5355, found: m/z 552.5366.

Fmoc-K-GSG-ALFA-OH

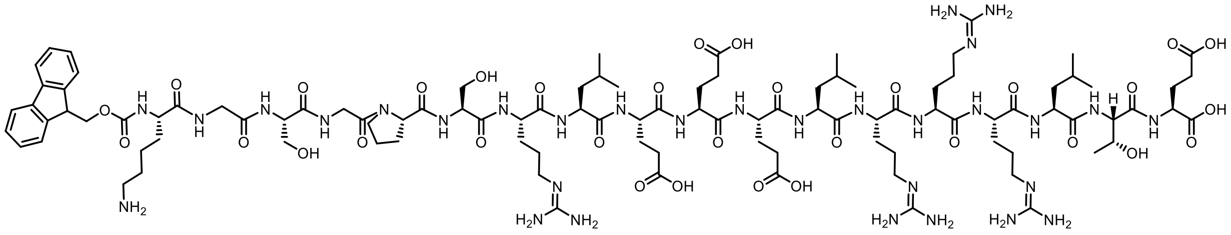

Fmoc-K-GSG-ALFA-NH$_2$ was synthesized starting from resin-bound compound **Fmoc-GSG-ALFA-OH**. In a first step, the dry Fmoc-GSG-ALFA-loaded resin (0.005 mmol, 1 eq) was swollen in NMP for 30 minutes in a BD syringe. 2 mL of a 20% v/v solution of piperidine in NMP were added to the resin, and the syringe was left shaking at room temperature for 15 minutes. The liquid was filtered off and the procedure was repeated once more. Afterwards, the resin was washed 3x with NMP/DCM/NMP.

Coupling with Fmoc-Lys(alloc)-OH (5 eq, 0.25 mmol, 92 mg) was performed twice under microwave irradiation (15 W, 70 °C, 2 x 15 min) using HATU (5 eq, 0.25 mmol, 96 mg), HOAt (5 eq, 0.25 mmol, 34 mg), and DIPEA (10 eq, 0.50 mmol, 87 µL) as activating agents dissolved in 3 mL of NMP. After the reaction, the solvent was removed, the resin was washed 3x with NMP/DCM/Et_2O, and dried under vacuum.

To the corresponding resin a solution of phenylsilane (17.0 eq., 0.6 M) in DCM was added and incubated at room temperature for five minutes. $Pd(PPh_3)_4$ (0.50 eq) was added and instantaneously placed in the microwave where the deprotection was per-formed under microwave irradiation (5 min, 38 °C, 15 W). Subsequently the resin was washed with DCM (5 x 5 mL). The deprotection process was performed three times in total.

ALFA-IP$^+$

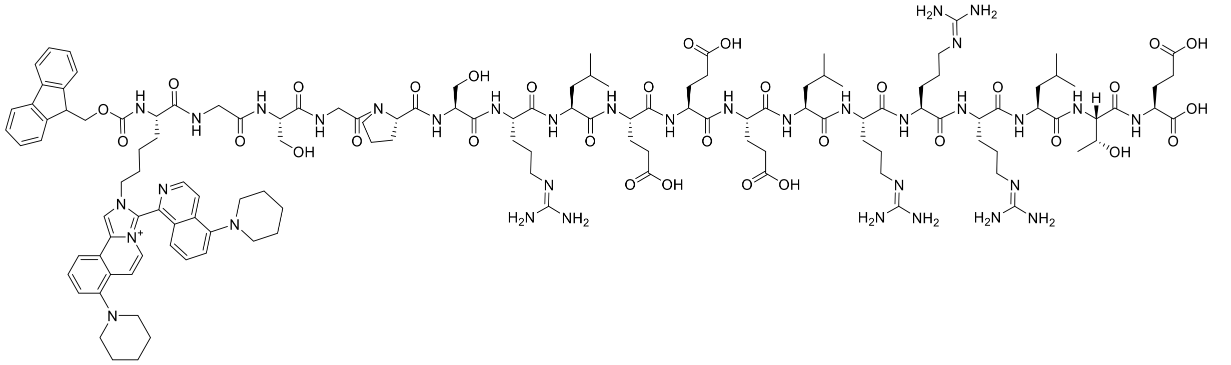

After the alloc was deprotected, the resin was treated with a solution of 25% HFIP/DCM (2 x 30 min). Then the beads were extensively washed by DCM (3 x 1 min).

To the corresponding resin a solution of **3a** (4 eq., 0.3 M) in 20% AcOH/DCM was added and shaken at room temperature for 12 hours. After the reaction, the solvent was removed, the resin was washed 3x with DCM/Et_2O, and dried under vacuum.

HR-MS (ESI+) – m/z calculated for $C_{132}H_{193}N_{36}O_{31}$, ([M+H]$^+$): 2779.5; found: m/z 2778.5; m/z calculated for $C_{132}H_{192}N_{36}O_{31}K$ ([M+K]$^+$): 2817.4; found: m/z 2816.4; m/z calculated for $C_{132}H_{194}N_{36}O_{31}$ ([M+2H]$^{2+}$): 1390.2389, found: m/z 1390.2392; m/z calculated for $C_{132}H_{196}N_{36}O_{31}Na$ ([M+2H+Na]$^{3+}$): 934.4890, found: m/z 934.4896; m/z calculated for $C_{132}H_{196}N_{36}O_{31}K$ ([M+2H+K]$^{3+}$): 939.8137, found: m/z 939.8104; m/z calculated for $C_{132}H_{195}N_{36}O_{31}$ ([M+3H]$^{3+}$): 927.1617, found: m/z 927.1655; m/z calculated for $C_{132}H_{195}N_{36}O_{31}Na$ ([M+3H+Na]$^{4+}$): 701.1186, found: m/z 701.1184; m/z calculated for $C_{132}H_{195}N_{36}O_{31}K$ ([M+3H+K]$^{4+}$): 705.1121, found: m/z 705.1147; m/z calculated for $C_{132}H_{196}N_{36}O_{31}$ ([M+4H]$^{4+}$): 695.6231, found: m/z 695.6211.

Linear Peptide NH_2-SGLRWK-OH

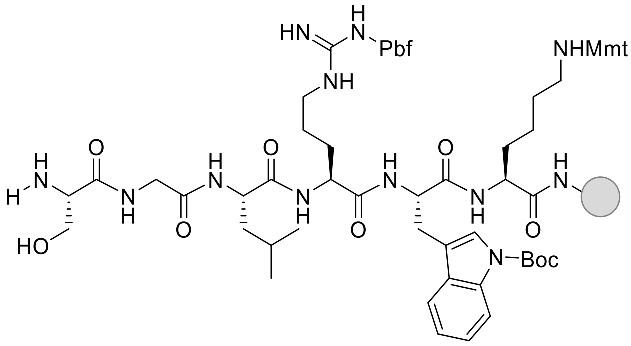

The synthesis of H-SGLRWK-NH_2 was carried out on a LL Rink Amide resin (0.36 mmol/g loading) on a 25 µmol scale according to **SOP1**. DIC (0.13 M in DMF) and Oxyma (0.25 M in DMF, + 0.1 mL DIPEA) were employed as activators, while deprotection was carried out with a 20% v/v solution of piperidine in DMF.

Table S4 – List of Fmoc-amino acids employed in the synthesis of H-SGLRWK-NH_2, with respective molecular weight, mass, and DMF volume required for preparing 0.2 M final solutions.

Amino Acid	Molecular Weight [g/mol]	Mass [g]	DMF Volume [mL]
Fmoc-Arg(Pbf)-OH	648.77	0.39	3
Fmoc-Gly-OH	297.31	0.12	2
Fmoc-Leu-OH	353.41	0.15	2
Fmoc-Lys(Mtt)-OH	624.77	0.25	2
Fmoc-Ser-OH	327.33	0.13	2
Fmoc-Trp(Boc)-OH	526.58	0.22	2

After coupling, the resin was washed by 2 x DMF, 2 x 50% DMF/H_2O and 2 x H_2O and the peptide was directly subjected to the next step.

Linear Peptide NH_2-SLRGDLK-$CONH_2$

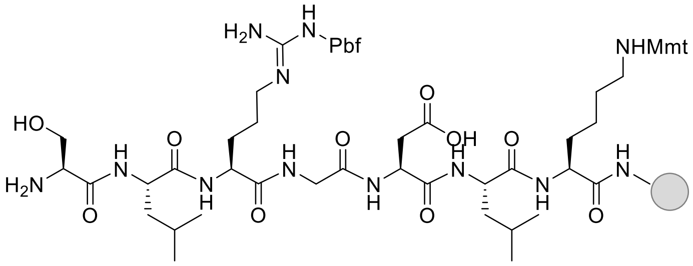

The synthesis of H-SGLRWK-NH_2 was carried out on a LL Rink Amide resin (0.36 mmol/g loading) on a 50 µmol scale according to **SOP1**. DIC (0.5 M in DMF) and Oxyma (1 M in DMF, + 0.5% v/v DIPEA) were employed as activators, while deprotection was carried out with a 20% v/v solution of piperidine in DMF.

Table S5 – List of Fmoc-amino acids employed in the synthesis of H-SGLRWK-NH_2, with respective molecular weight, mass, and DMF volume required for preparing 0.2 M final solutions.

Amino Acid	Molecular Weight [g/mol]	Mass [g]	DMF Volume [mL]
Fmoc-Arg(Pbf)-OH	648.77	0.39	3
Fmoc-Gly-OH	297.31	0.12	2
Fmoc-Leu-OH	353.41	0.15	2
Fmoc-Lys(Mtt)-OH	624.77	0.25	2
Fmoc-Ser-OH	327.33	0.13	2

After coupling, the resin was washed by 2 x DMF, 2 x 50% DMF/H_2O and 2 x H_2O and the peptide was directly subjected to the next step.

Fluorescent Cyclic Peptide from NH_2-SGLRWK-OH (CP-IP$^+$)

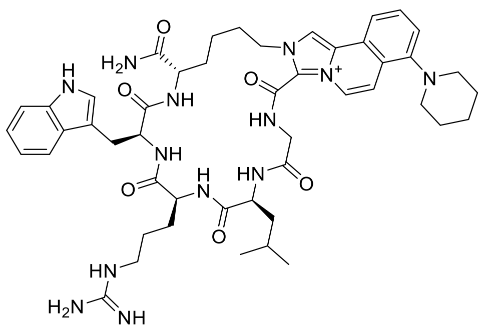

The cyclization was performed adapting a published protocol. [7]

A solution of $NaIO_4$ (0.5 M, 5.0 equiv) was added to the resin and shaken for 60 min at room temperature. After the reaction, the solvent was removed, the resin was washed by 2 x H_2O, 2 x 50% DMF/H_2O, 2 x DMF and 2 x DCM.

To the corresponding resin a solution of 25% HFIP/DCM (17.0 eq., 0.6 M) in DCM was added and incubated at room temperature (2 x 30 minutes). After the reaction, the solvent was removed, the resin was washed by 2 x DCM.

After the Mmt was cleavage, a solution of **3a** (4 eq., 0.3 M) in 50% TFE/AcOH was added to the resin and shaken at room temperature for 12 hours. After the reaction, the solvent was removed, the resin was washed 3x with DCM/Et_2O, and dried under vacuum.

HR-MS (ESI+) – m/z calculated for $C_{49}H_{65}N_{12}O_6$ ([M+H]$^+$): 918.5; found: m/z 918.5; m/z calculated for $C_{49}H_{66}N_{12}O_6$ ([M+2H]$^{+2}$): 459.7648; found: m/z 459.7632.

Fluorescent Cyclic Peptide from NH_2-SLRGDLK-OH (cRGD-IP$^+$)

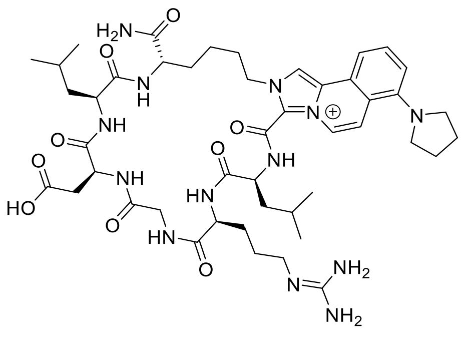

The cyclization was performed adapting a published protocol. [7]

A solution of $NaIO_4$ (0.5 M, 5.0 equiv) was added to the resin and shaken for 60 min at room temperature. After the reaction, the solvent was removed, the resin was washed by 2 x H_2O, 2 x 50% DMF/H_2O, 2 x DMF and 2 x DCM.

For the Lys-Mmt deprotection a solution of TFA (1%, v/v) in DCM was added to the corresponding resin and incubated at room temperature (4 x 15 minutes). After the reaction, the solvent was removed, the resin was washed by 5 x DCM.

After the Mmt was cleaved, a solution of **3a** (4 eq., 0.3 M) in 50% TFE/AcOH was added to the resin and shaken at room temperature for 12 hours. After the reaction, the solvent was removed, the resin was washed 3x with DCM/Et_2O, and dried under vacuum.

HR-MS (ESI+) – m/z calculated for $C_{46}H_{69}N_{13}O_9$ ($[M+2H]^{2+}$): 473.7665, found: m/z 473.7661.

7. Protein Labelling

Evaluation of IP^+ formation at pH 4

N_α-acetyl-L-lysine (20 mM) and aldehyde **3b** (40 mM) were dissolved in a mixture of 100 mM acetate buffer (pH 4) in D_2O, MeCN-d^3 and H_2O (2:2:1, v/v/v) and incubated at room temperature for 24 hours. Because the conversion was too low to quantify by NMR as initially planned, an aliquot was diluted in MeOH and analysed by LC-MS. The chromatograms and the +ESI spectrum of the peak at 5.2 min is shown below. The peak at 637.3 m/z corresponds to the expected product **8b**.

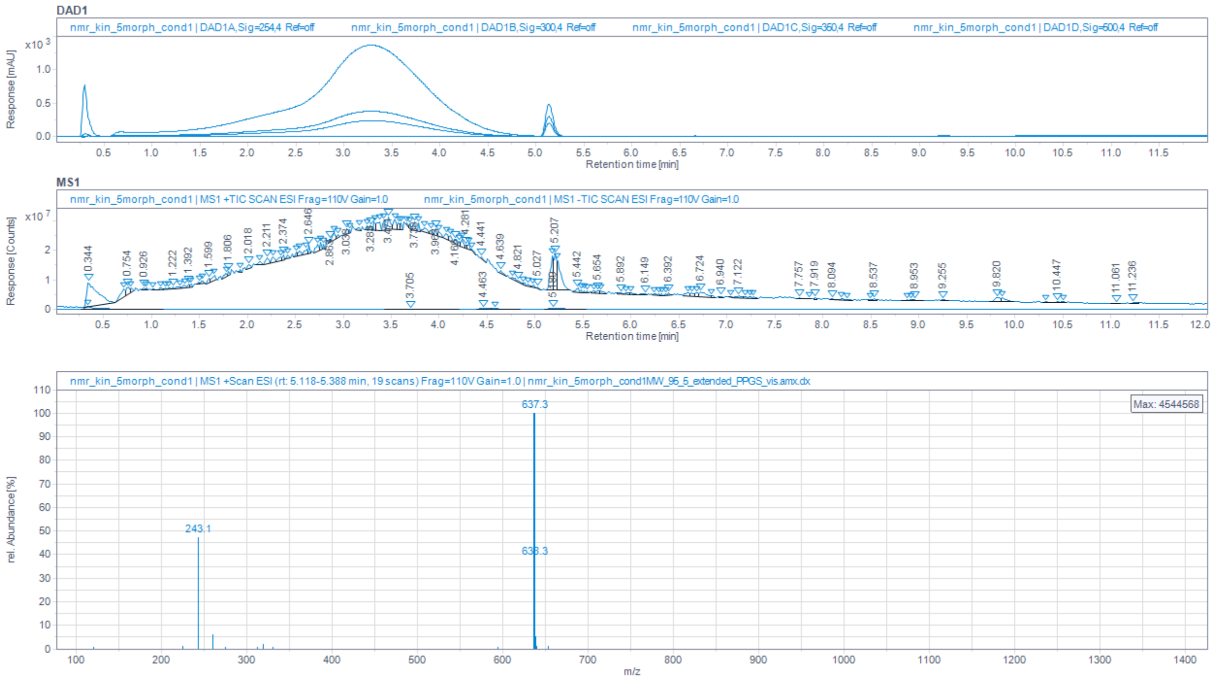

Figure S47. LC-MS Chromatograms of the labelling reaction with aldehyde 3b carried out at pH 4 after 24 hours at room temperature and the +ESI spectrum of the peak at 5.2 min.

Protein Labelling conditions 1

In 1.5 mL Eppendorf cups BSA (Sigma Aldrich A2153) or Lysozyme (from chicken egg white, Sigma Aldrich L4919) at a concentration of 50 µM and the aldehyde **3b** at a concentration of 1 mM (20 equiv.) were dissolved in a 1:4 mixture of MeCN and 100 mM sodium acetate buffer with a pH of 4 or 5 and shaken at 300 rpm and 30 °C for 5 days. The samples were then analysed by SDS-polyarclamide gel electrophoresis (wells 1, 2, 4 and 5).

Protein Labelling conditions 2

In 1.5 mL Eppendorf cups BSA (Sigma Alrdich A2153) or Lysozyme (from chicken egg white, Sigma Aldrich L4919) at a concentration of 250 µM and the aldehyde **3a** at a concentration of 25 mM (100 equiv.) were dissolved in a 45:50:5 mixture of water, MeCN and AcOH and shaken at 300 rpm and 30 °C for 18 hours. The samples were then diluted x5 with PBS and centrifuged at 6000 rcf for 5 minutes. The supernatants were dialysed against PBS in 0.5mL 3.5 kDa Slide-A-Lyzer MINI dialysis devices for 40 hours, with exchanging of the dialysate after the first 16 hours. The samples were then analysed by SDS-polyarclamide gel electrophoresis (wells 7 and 8).

SDS-polyarclamide gel electrophoresis

After centrifugation at 6000 rcf for 5 minutes, an aliquot of each sample was diluted x2 with 2x sample buffer (components) and heated to 95 °C for 5 minutes. 10 µL of each sample and a protein ladder were then loaded into the wells of a polyacrylamide gel (4% stacking gel, 12.5% resolving gel) according to the table below.

The electrophoresis was run in Tris-Glycine buffer for about 45 minutes at 205 V. The gel was then

washed twice for 5 minutes with PBS and then imaged using a phone camera and an azure biosystems c300 imager using blue light epi-illumination. The gel was then stained with coomassie blue, washed for 1 hour with demineralized water and then imaged with the imager using UV (302 nm) transillumination. The resulting images of the full gels are shown below.

Table S6. Well assignment for SDS–PAGE analysis of proteins labelled under conditions 1 and 2.

Well position	Labelling conditions
1	LYZ, conditions 1, pH 5
2	LYZ, conditions 1, pH 4
3	*
4	BSA, conditions 1, pH 5
5	BSA, conditions 1, pH 4
6	*
7	LYZ, conditions 2
8	BSA, conditions 2
9	Protein ladder

*Electrophoresis in these wells failed due to a problem with pH of the samples

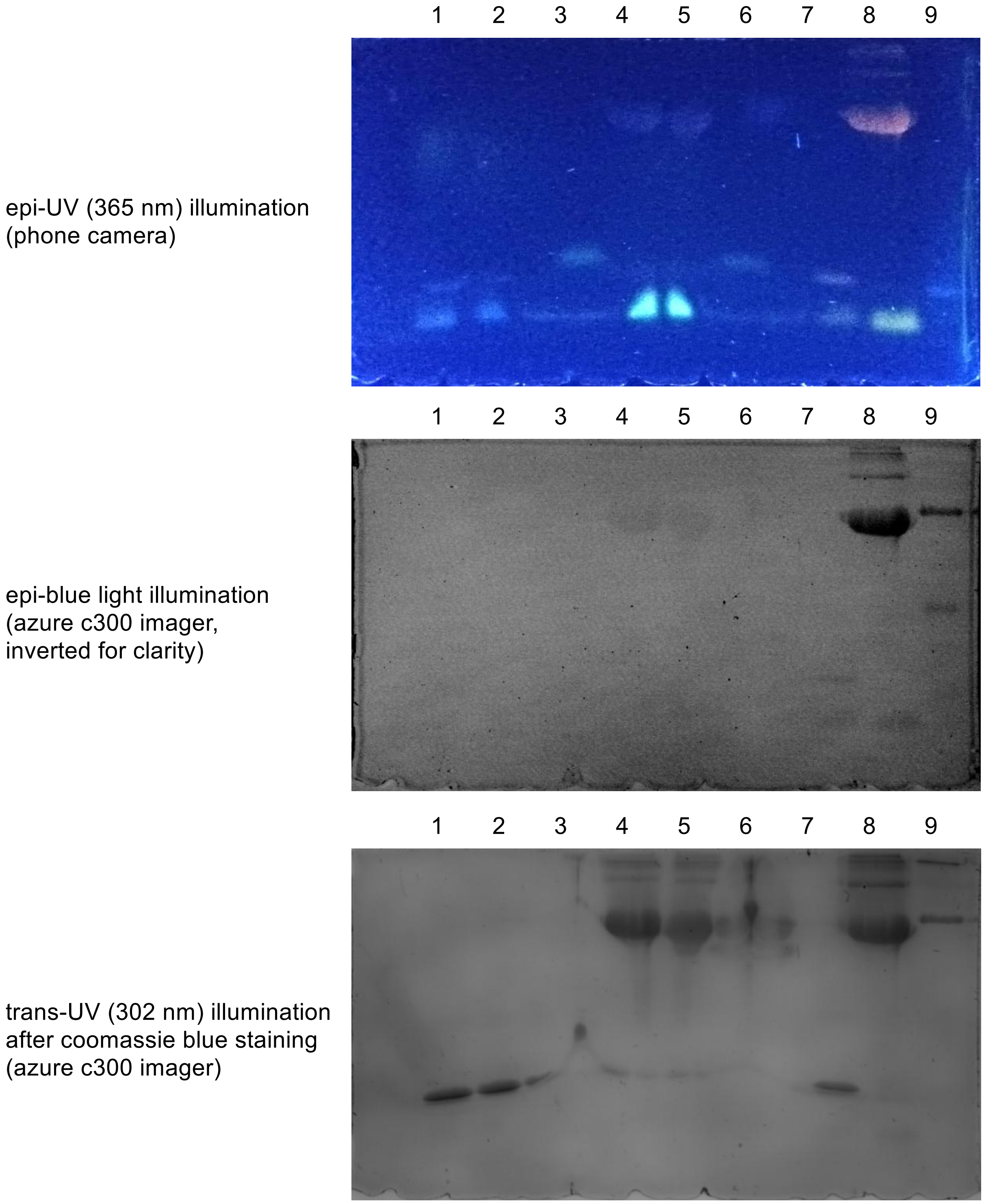

Figure S48. Images of the SDS-polyacylamide electrophoresis gel of the labelled proteins under UV and blue light illumination and imaged gel after staining with coomassie blue.

Protein Labelling conditions in buffer

In 1.5 mL Eppendorf cups BSA (Sigma Aldrich A2153) at a concentration of 3 mg/mL and aldehyde **3l** at a concentration of 10 mM were dissolved in 1 mL of a 9:1 mixture of 0.1 M aqueous sodium acetate buffer with pH 4 and DMSO. The mixture was shaken for 24 hours at 37°C in the dark, after which some precipitation had occurred. After dilution with 2 mL of PBS buffer and vortexing, everything got into solution again and the mixture was dialysed in a 3 mL 3.5 kDa Slide-A-Lyzer dialysis cassette against PBS (500 mL) for 24 hours at room temperature with 3 buffer exchanges. In the end, no absorbance of

the aldehyde could be detected in the outer liquid with a NanoDrop spectrometer. For measuring the photophysical properties of the resulting conjugated BSA, it was diluted by a factor of 2 with PBS buffer.

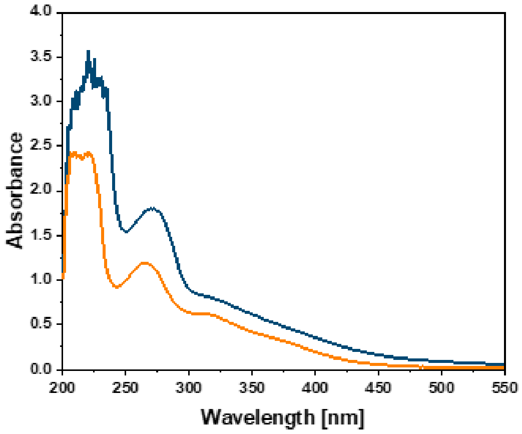

Figure S49. Absorbance spectra of the labelled BSA (blue line) and of 8l at 50 µM in PBS buffer (orange line).

An estimation of the degree of labelling was obtained from the absorbance spectrum of the conjugate (see Fig. S45). For that, the molar extinction coefficients of **8l** at 350 nm (8400 $cm^{-1}M^{-1}$) and at 280 nm (18600 $cm^{-1}M^{-1}$) were used. The extinction coefficient at 350 nm was used to estimate a dye concentration of 74 µM from the absorbance of the protein solution at 350 nm. The extinction coefficient of **8l** at 280 nm was used to subtract the contribution of the dye to the absorption of the labelled protein at 280 nm and the resulting corrected absorbance was converted to a protein concentration of 6.6 µM using the extinction coefficient of BSA at 280 nm (43824 $cm^{-1}M^{-1}$). This value is close to the expected concentration after the in total 6-fold dilution of the 3 mg/mL starting concentration (7.5 µM). This estimation, which assumes that the contribution to the extinction coefficient of each labelled Lys residue in BSA is the same as the extinction coefficient of **8l**, gave a degree of labelling of 11.2 fluorophores per protein molecule.

8. Bioimaging

Immobilizing ALFA-IP^+ magnetic agarose beads

ALFA Selector CE magnetic beads, Cat No: N1517 (NanoTag Biotechnologies GmbH) were washed twice with 500µL of phosphate buffer saline (PBS; 137mM NaCl, 2,7 mM KCl, 10 mM Na2HPO4, 2mM KH_2PO_4, pH = 7,4) using a magnetic holder. Magnetic-agarose beads (~25 µL) were finally resuspended in 100 µL of PBS containing 400 µg of unconjugated ALFA peptide (NanoTag Biotechnologies, Cat No: N1520) or 100 µL of PBS with a saturated amount of ALFA-IP^+. Tubes were placed on a fixed-angle rotator with gentle turning and incubated for 1 hour at room temperature.

After incubation, the tubes were placed on the magnetic holder, the solution removed, and washed three times with PBS. Finally, beads were resuspended in a 30 µl Mowiöl (12 mL of 0.2 M Tris buffer, 6 mL distilled water, 6 g glycerol, 2.4 g Mowiol 4-88, Merck Millipore) and placed on a microscope slide, covered with an 18 mm cover glass N°1, and kept at 40°C until the mounting medium dried. Slides were later imaged with a confocal microscope.

Acquisition:

Images were acquired with a Stellaris SP8 confocal laser scanning microscope from Leica Microsystems equipped with HC-PL APO CS2 100x/1.40 oil objective. Samples have been excited with a 405 diode at 2% power output and a line accumulation of two. The detection happened with a Leica Power HyDS (SiPM 450-601) detector with a detection range set to 450-600 nm. The pixel size was 227 nm, and the scanning speed was 400 Hz. Images were processed using ImageJ, and grayscale levels were set to 1 and 150 for all images to allow a direct comparison.

Cell culture and maintenance:

Cos-7 cells were cultured in Petri dishes under standard conditions in a humidified incubator at 37°C with 5% CO_2. Cells were grown in complete Dulbecco's Modified Eagle Medium (DMEM, ThermoFisher Scientific) supplemented with 10% fatal bovine serum (FBS; ThermoFisher), 4 mM L-glutamine, and 1% penicillin/streptomycin (ThermoFisher). For immunostaining experiments, cells were seeded onto 18 mm poly-L-lysine-coated coverslips placed in 12-well plates and incubated for 16-18 hours under the same conditions.

Cell Fixation:

The culture medium was replaced with 0.5 ml of 4% paraformaldehyde (PFA) in PBS (137 mM NaCl, 2.7 mM KCl, 10 mM Na_2HPO_4, 2 mM KH_2PO_4, pH 7.4) and incubated at room temperature (RT) for 15 minutes. After a short rinse with PBS, residual aldehyde groups were then neutralized by incubating the cells in PBS supplemented with 0.1 M glycine for an additional 15 minutes at RT.

Premixing and immunostaining:

Fixed and quenched cells were blocked and permeabilized with 3% bovine serum albumin (BSA) and 0.1% Triton X-100 (Sigma Aldrich) in PBS for 30 minutes at room temperature (RT). Before immunolabeling, mouse monoclonal anti-tubulin (SySy, Cat# 302211) primary antibody (1.Ab) was premixed in 15 µl PBS with their anti-mouse IgG1 HaloTag fusion secondary nanobody (2.NbHT; NanoTag Biotechnologies, Cat# N2041) and the fluorescent HaloTag ligand (HL) in a 1:3:8 molar ratio to achieve a final concentration of 7 nM 1.Ab, 21 nM 2.NbHT, and 56 nM HL. This mixture was then incubated for 20 minutes at RT. The premixed complex was then diluted to 300 µl with blocking buffer and applied to the cells for 1 hour at RT with gentle agitation. Following incubation, the samples were washed three times with PBS for 5 minutes each, briefly rinsed in distilled water, and then mounted with Mowiol (12 ml of 0.2 M Tris buffer, 6 ml distilled water, 6 g glycerol, and 2.4 g Mowiol 4–88, Merck Millipore). Mounted samples were allowed to dry for 30 minutes at RT before imaging.

Confocal microscopy:

All confocal images were acquired using a STELLARIS 8 PP STED FALCON microscope (Leica Microsystems, Wetzlar, Germany) equipped with an HC PL APO 100×/1.4 OIL STED W objective. Excitation was performed using a 405 nm diode laser set to 20% power, and emission was detected

with the Power HyD S detector in photon counting mode, with a detection range of 550–750 nm. Apart from adjusting grey levels, the confocal images were not further processed.

9. LCMS Analysis for Chemoselectivity

Nucleophilic amino acids (25 mM) with an Fmoc protecting group on the *N*-termi were incubated with isoquinoline aldehyde **3** in 50% AcOH/H_2O for 12 hours. Afterwards, the reaction mixture was monitored by HPLC-MS.

Fmoc-Lys-OH, Fmoc-Met-OH, Fmoc-Tyr-OH and Fmoc-Ser-OH were incubated wth 50 mM isoquinoline aldehyde **3**, while Fmoc-Arg-OH was incubated with 100 mM sioquinoline aldehyde **3**, and Fmoc-His-OH was incubated with 200 mM isoquinoline aldehyde **3.**

Especially, Fmoc-Cys-OH was incubated with 100 mM isoquinoline aldehyde **3** and 200 mM TCEP.

The HPLC-MS result of the reaction mixture and the starting material amino acids are shown as follows:

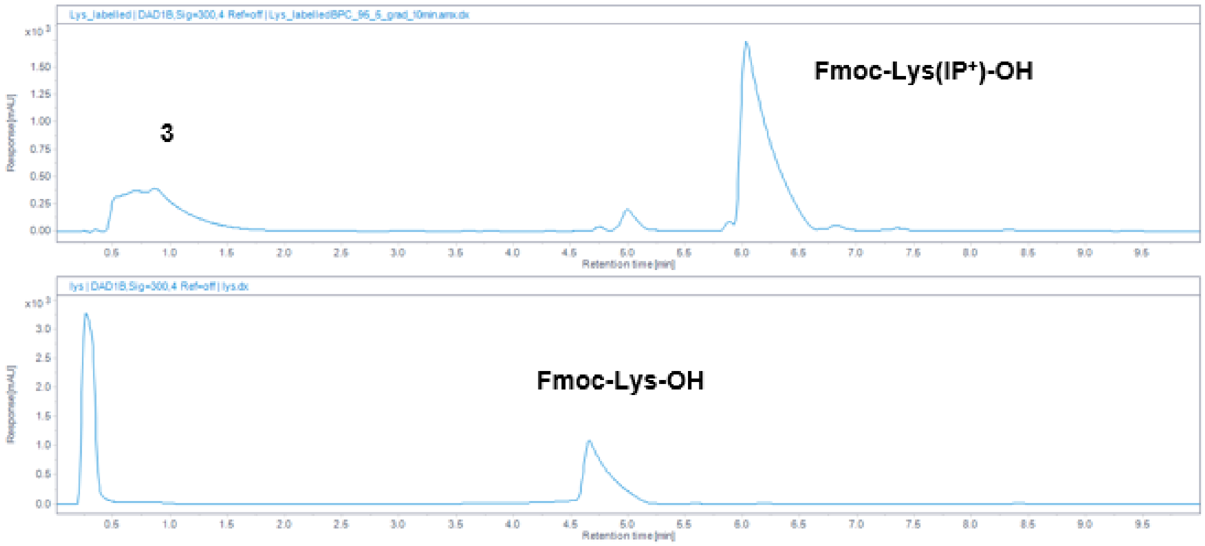

Figure S50. HPLC trace of Fmoc-Lys-OH in the presence of isoquinoline aldehyde 3 in 50% AcOH/H_2O at 0h (top) and after 12h (bottom).

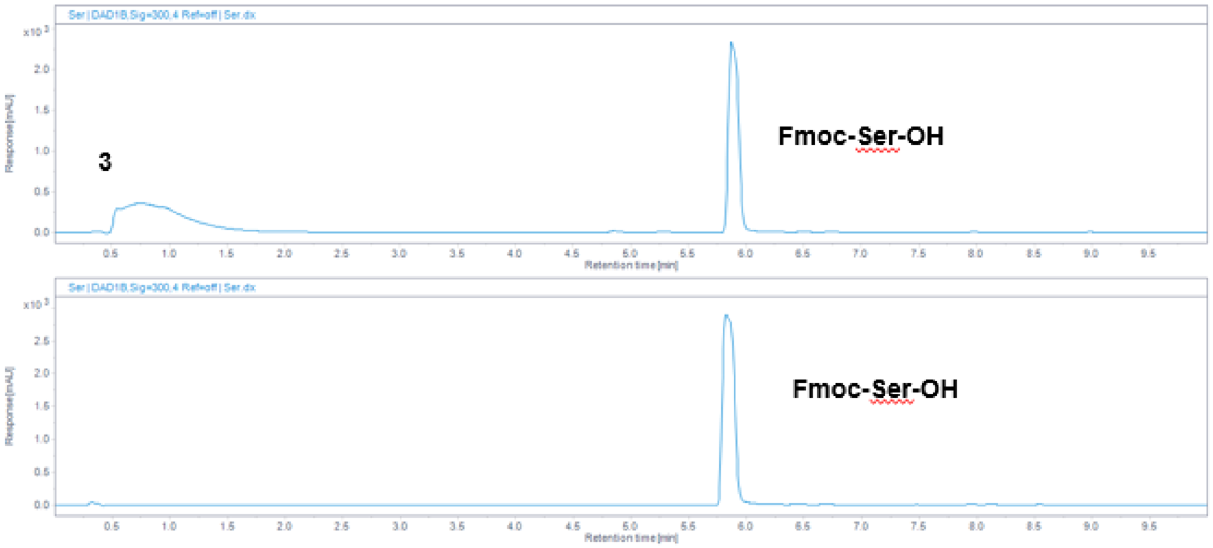

Figure S51. HPLC trace of Fmoc-Ser-OH in the presence of isoquinoline aldehyde 3 in 50% AcOH/H_2O at 0h (top) and after 12h (bottom).

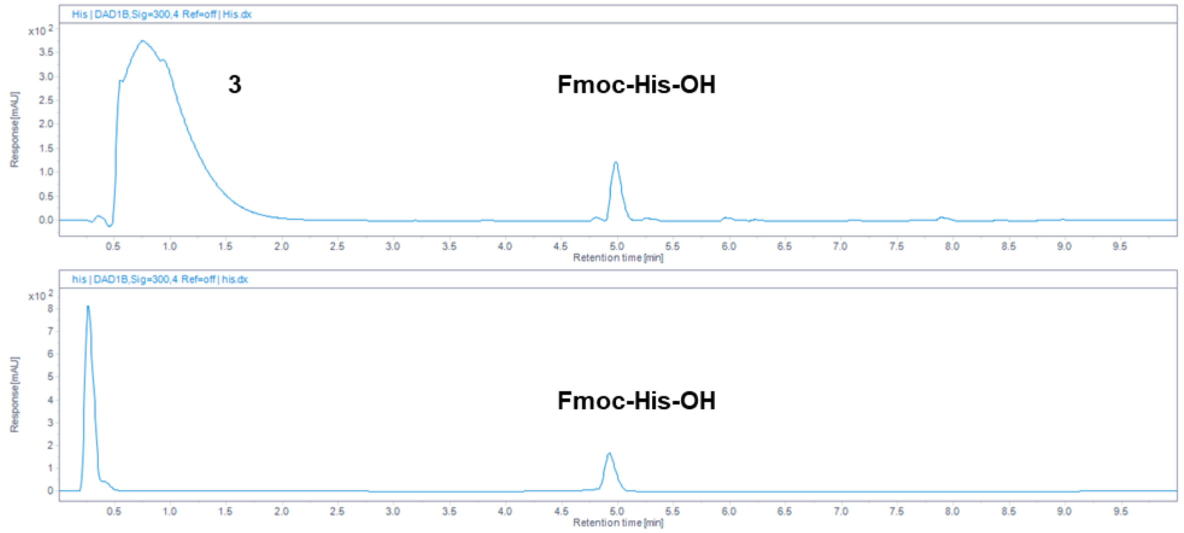

Figure S52. HPLC trace of Fmoc-His-OH in the presence of isoquinoline aldehyde 3 in 50% AcOH/H_2O at 0h (top) and after 12h (bottom).

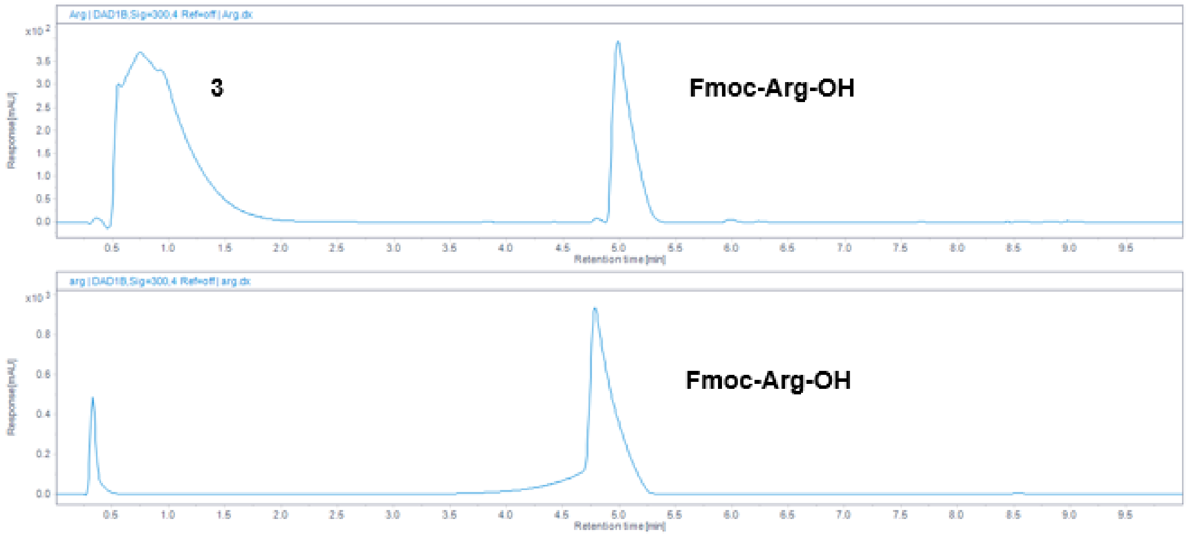

Figure S53. HPLC trace of Fmoc-Arg-OH in the presence of isoquinoline aldehyde 3 in 50% AcOH/H_2O at 0h (top) and after 12h (bottom).

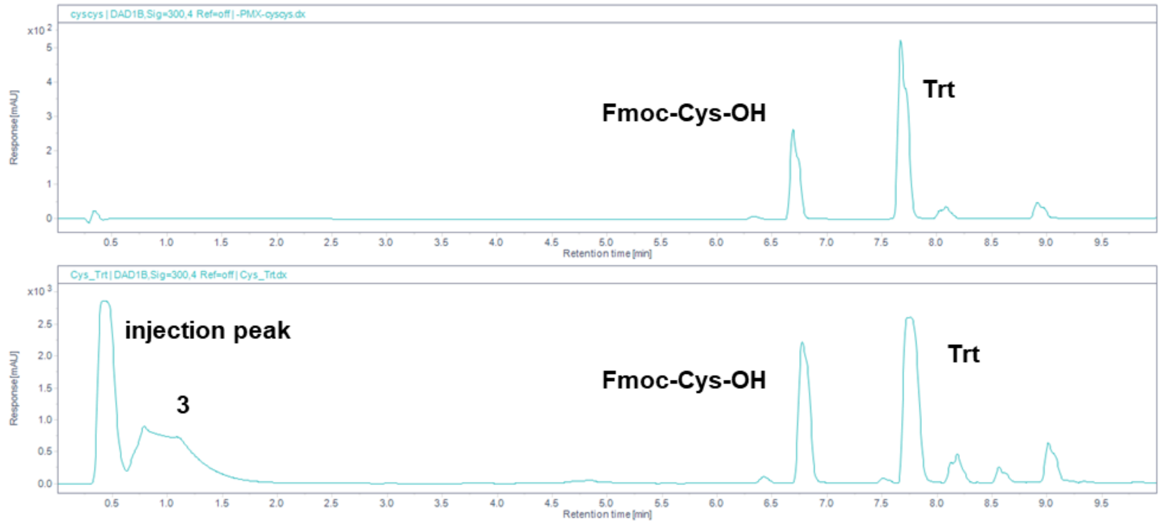

Figure S54. HPLC trace of Fmoc-Cys-OH in the presence of isoquinoline aldehyde 3 in 50% AcOH/H_2O at 0h (top) and after 12h (bottom).

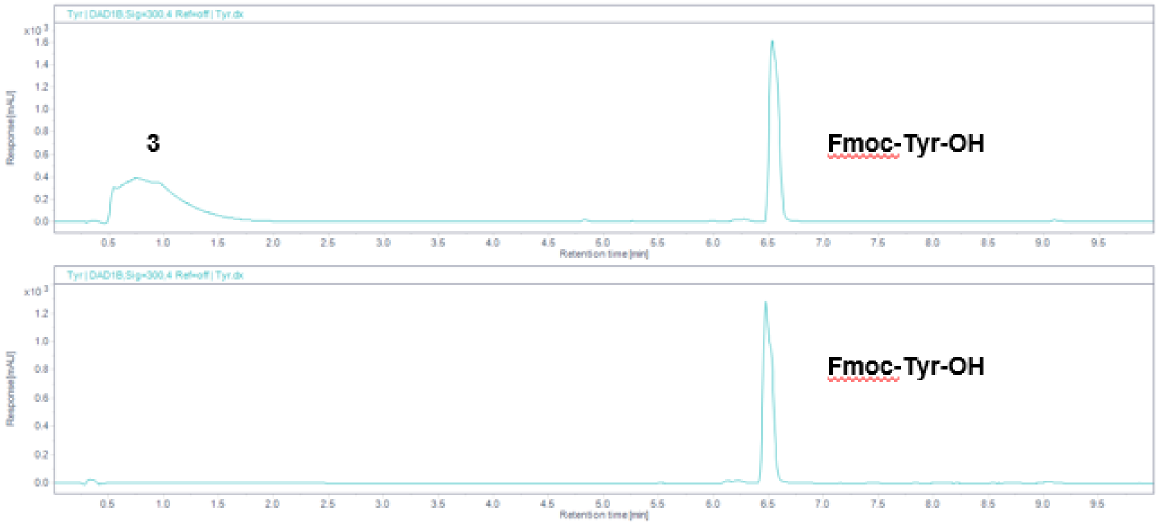

Figure S55. HPLC trace of Fmoc-Tyr-OH in the presence of isoquinoline aldehyde 3 in 50% AcOH/H_2O at 0h (top) and after 12h (bottom).

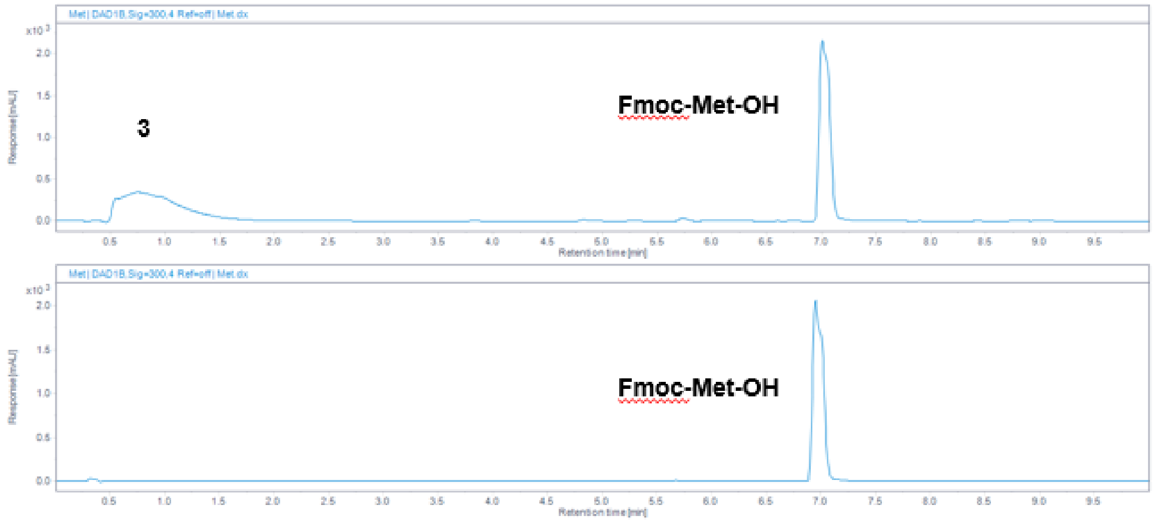

Figure S56. HPLC trace of Fmoc-Met-OH in the presence of isoquinoline aldehyde 3 in 50% AcOH/H_2O at 0h (top) and after 12h (bottom).

10. Small Molecular Analysis: NMR spectra

7a

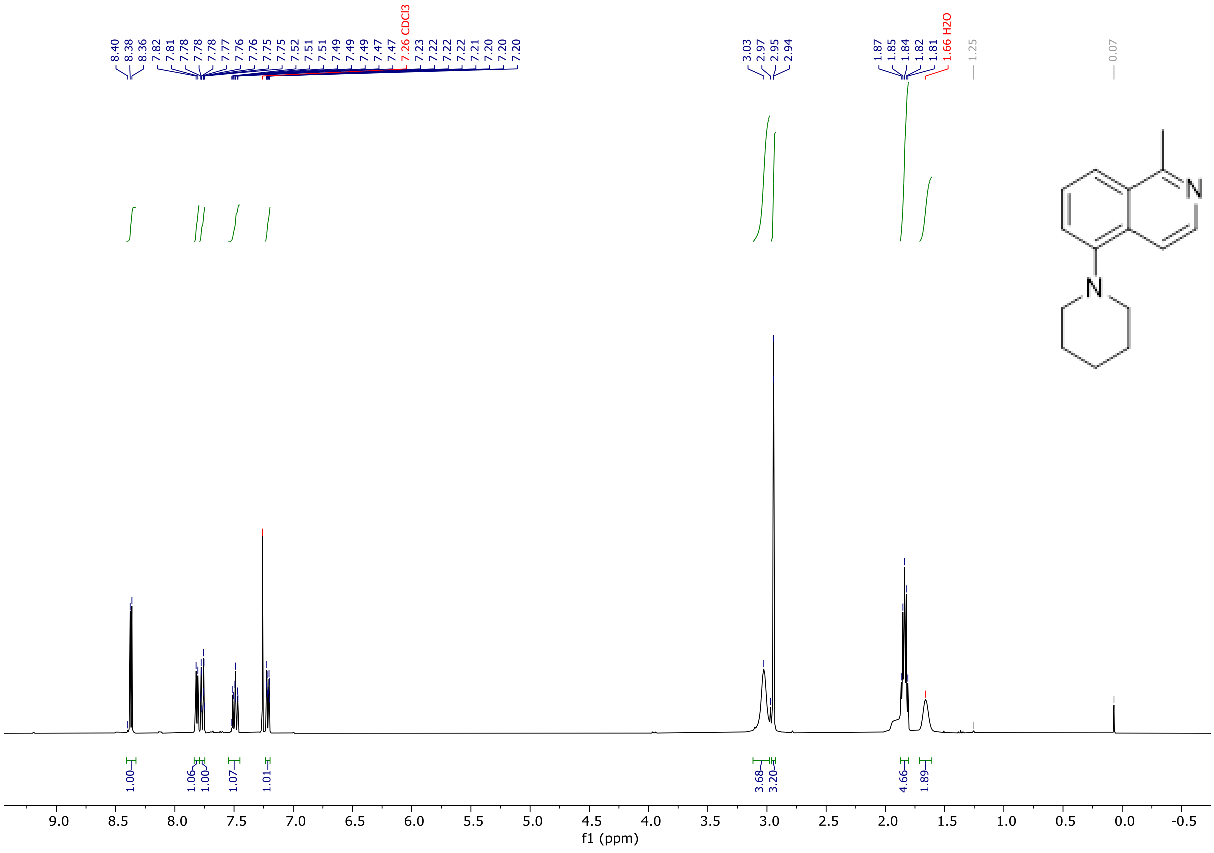

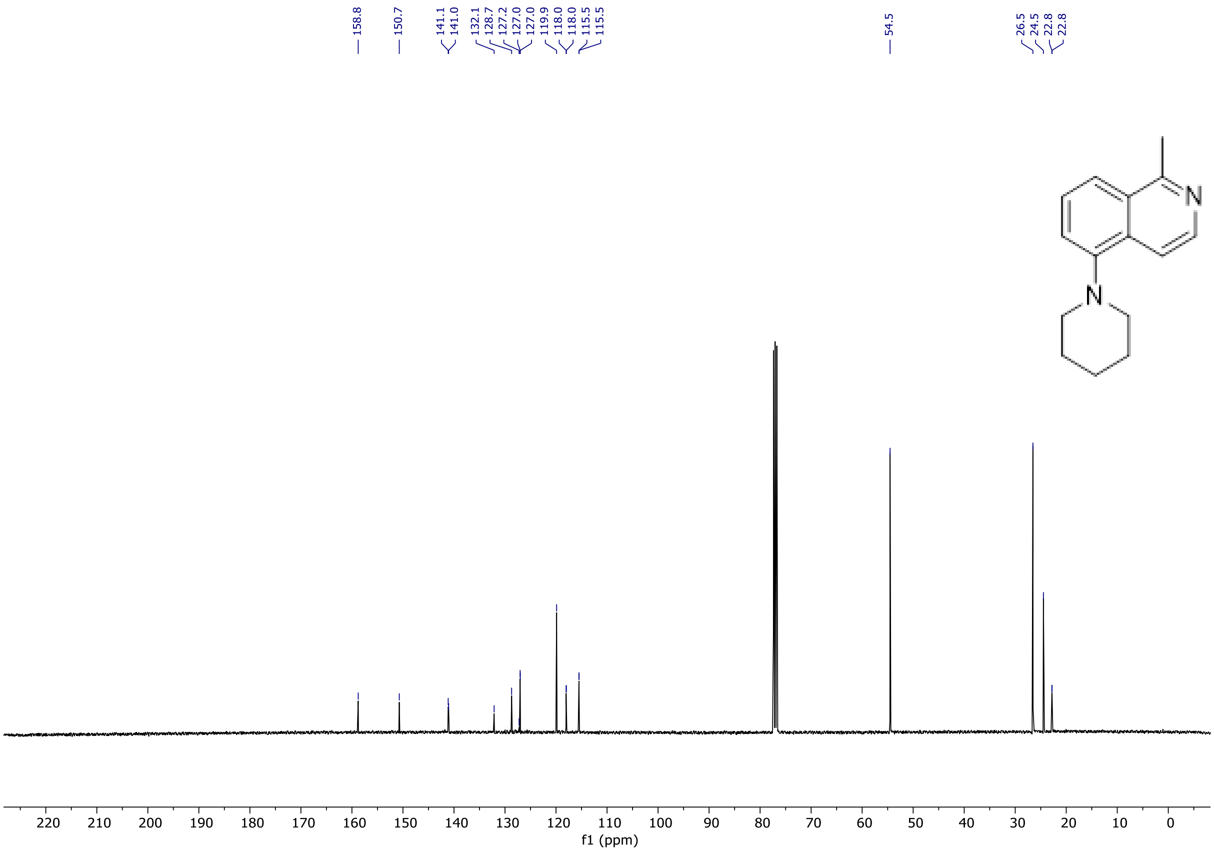

7b

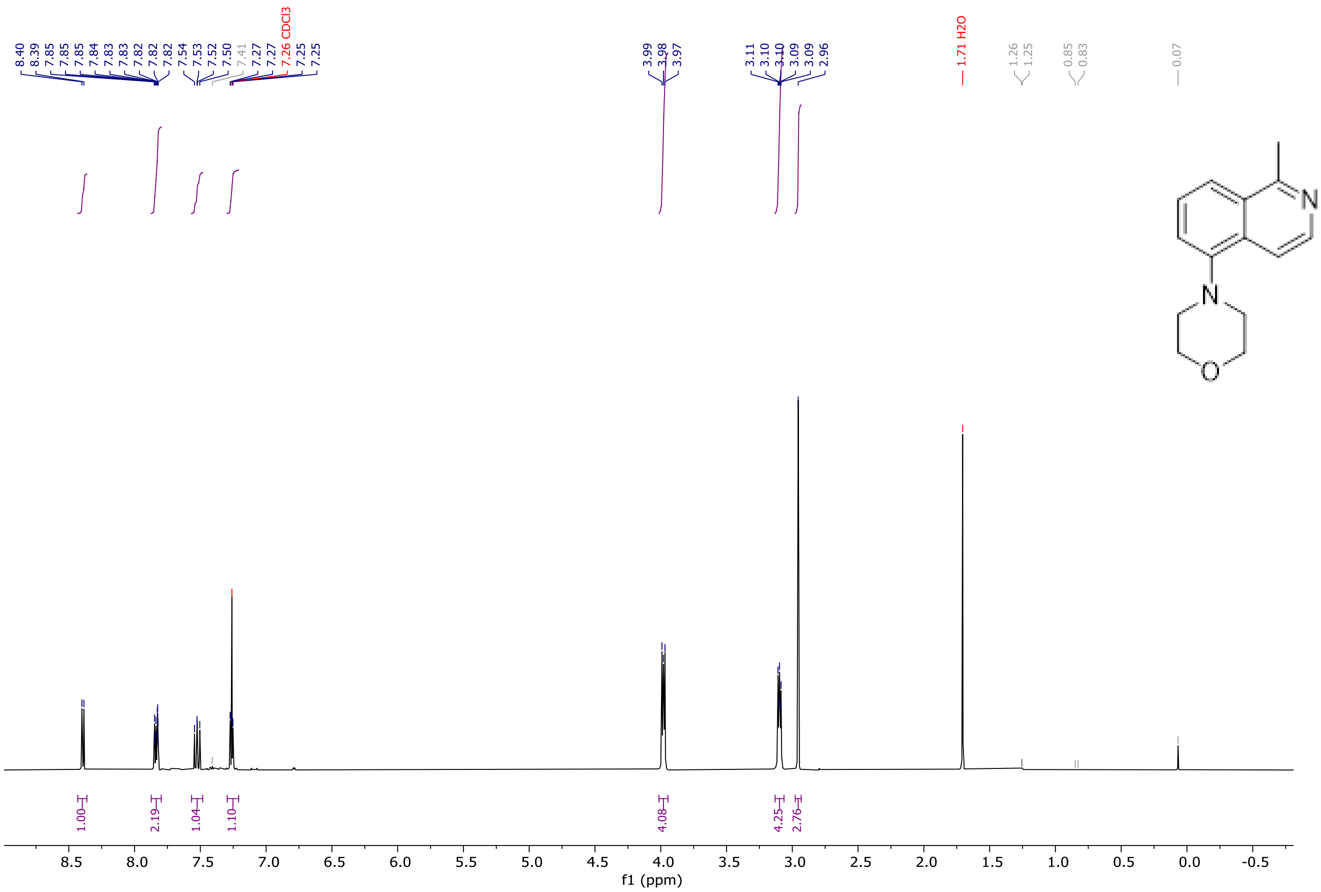
8.40
8.39
7.85
7.85
7.85
7.84
7.83
7.83
7.82
7.82
7.82
7.54
7.53
7.52
7.50
7.41
7.27
7.27
7.26 CDCl3
7.25
7.25
3.99
3.98
3.97
3.11
3.10
3.10
3.09
3.09
2.96
1.71 H2O
1.26
1.25
0.85
0.83
0.07
N
N
O
1.00
2.19
1.04
1.10
4.08
4.25
2.76
8.5
8.0
7.5
7.0
6.5
6.0
5.5
5.0
4.5
4.0
3.5
3.0
2.5
2.0
1.5
1.0
0.5
0.0
-0.5
f1 (ppm)

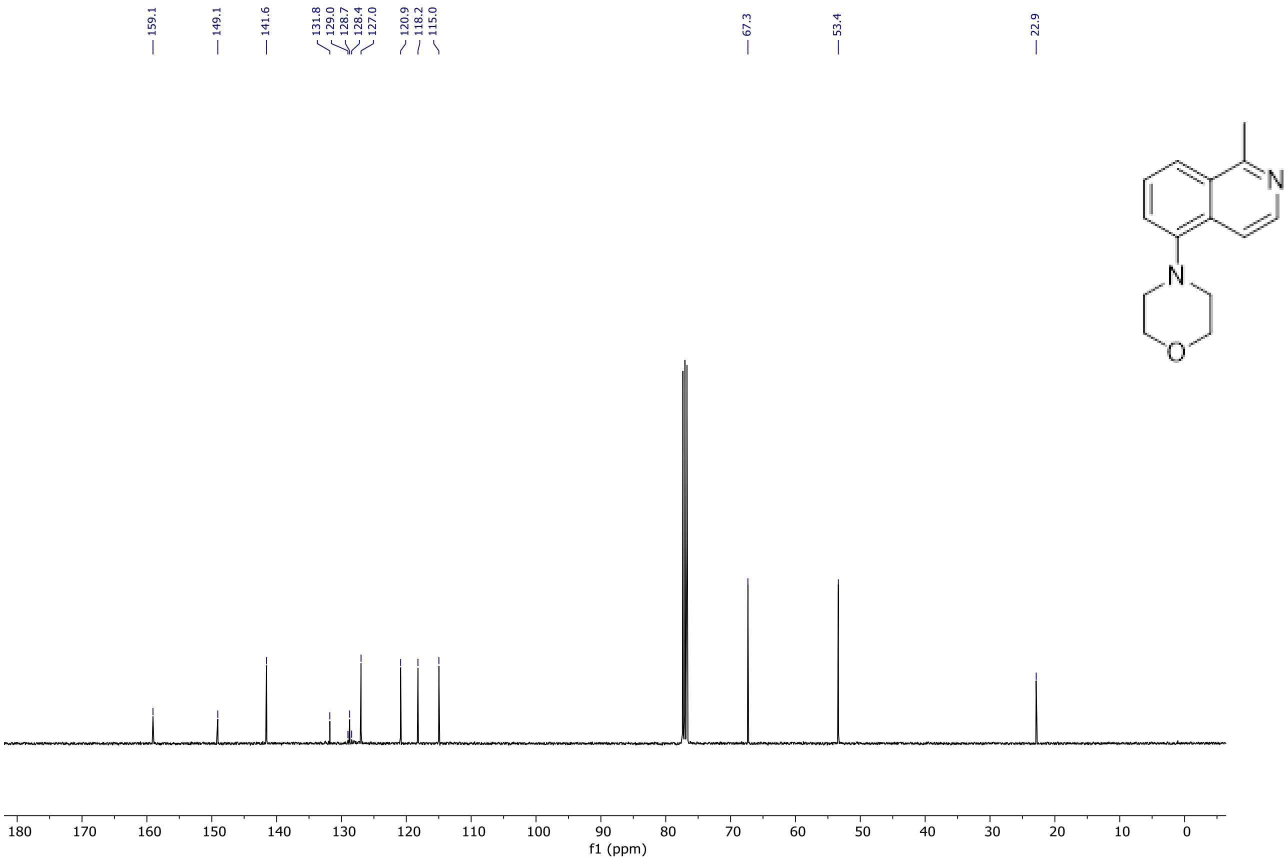
159.1
149.1
141.6
131.8
129.0
128.7
128.4
127.0
120.9
118.2
115.0
67.3
53.4
22.9
N
N
O
180
170
160
150
140
130
120
110
100
90
80
70
60
50
40
30
20
10
0
f1 (ppm)

7d

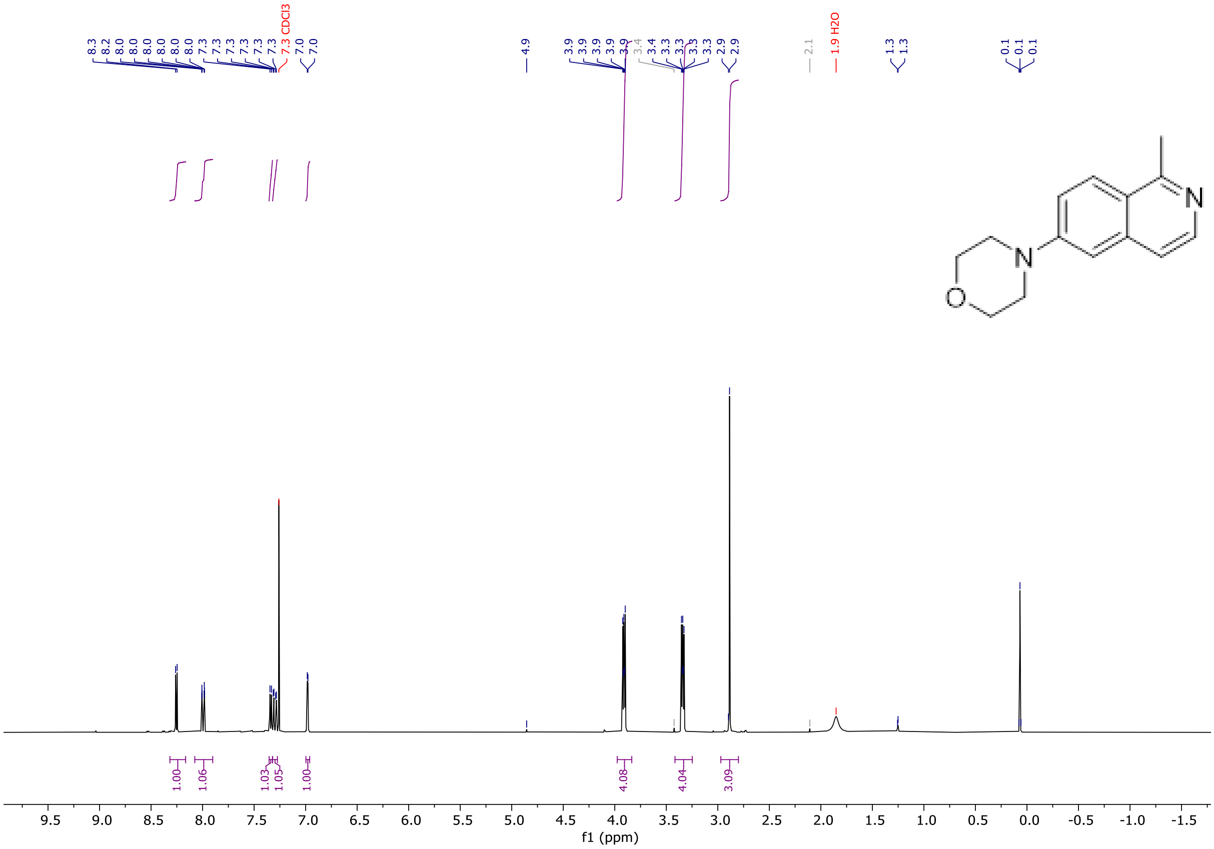

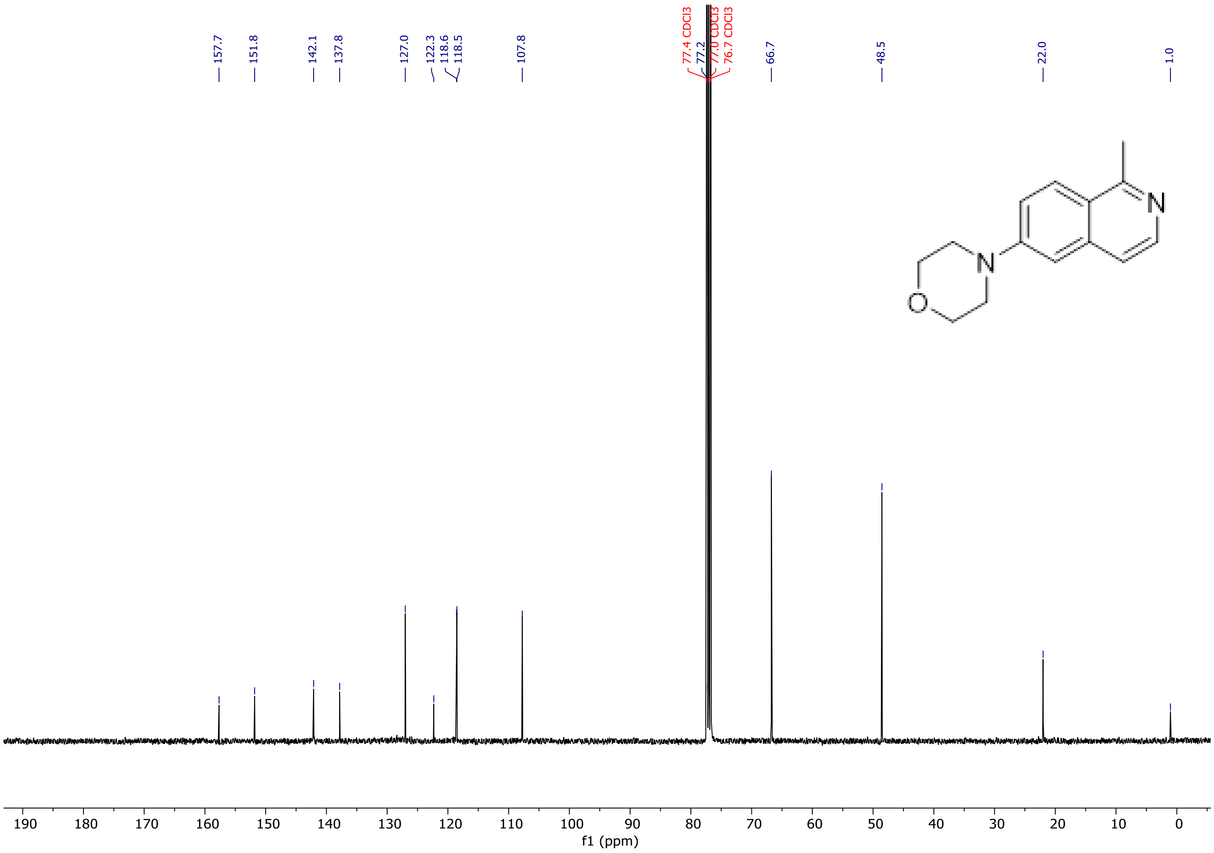

7e

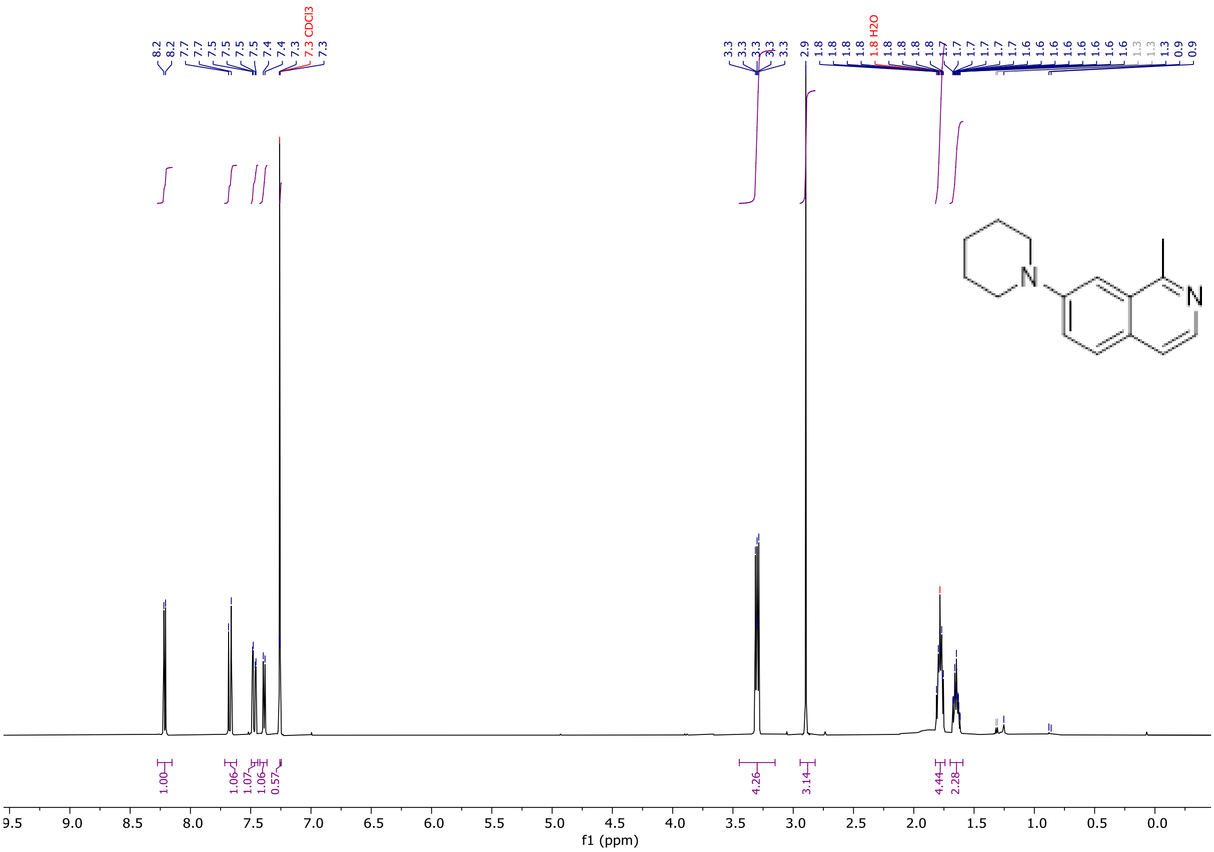

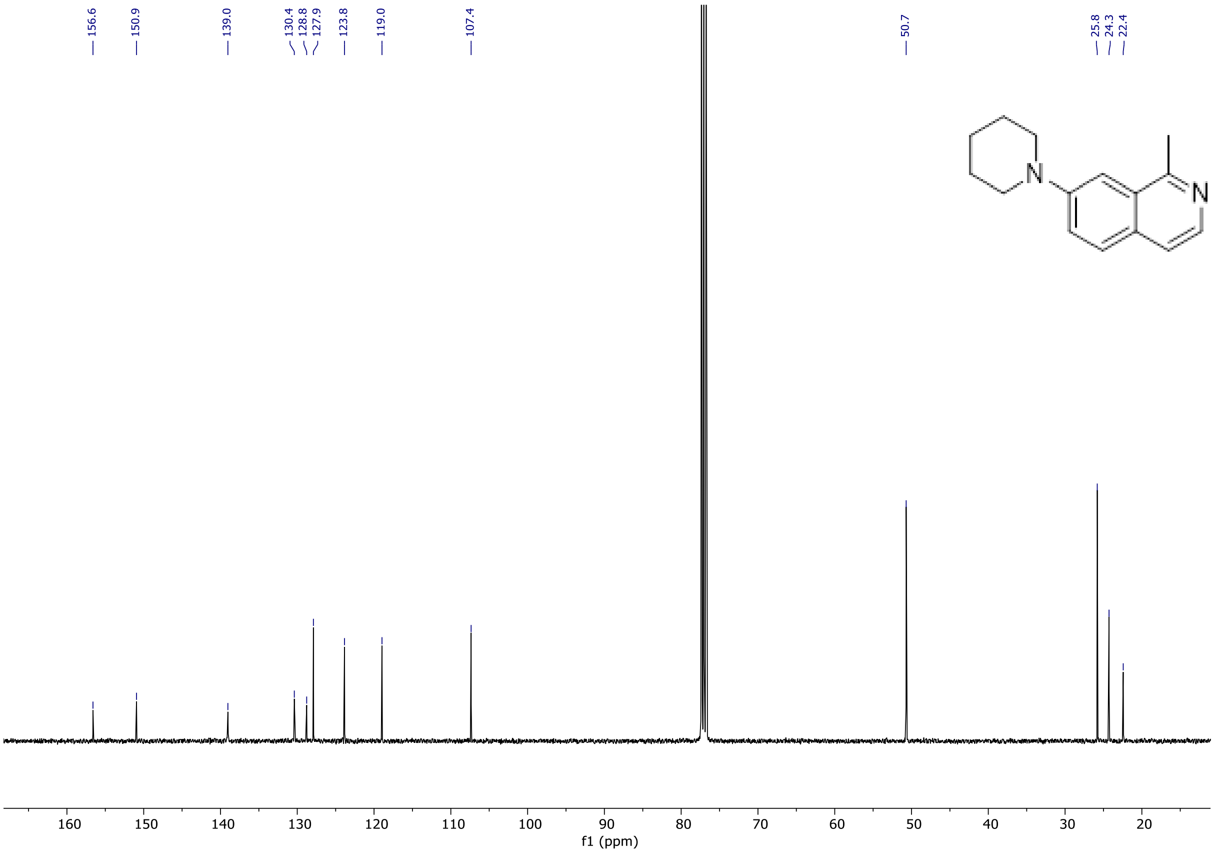

7f

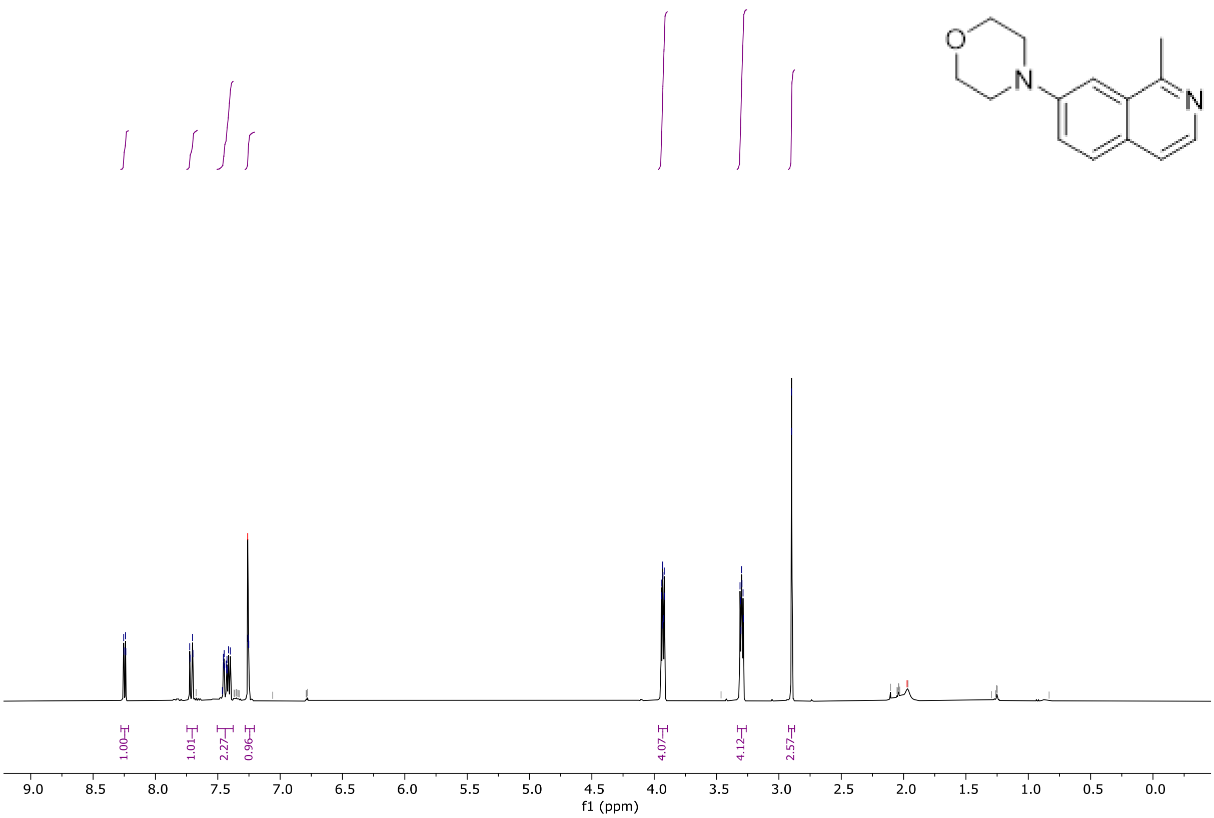

O
N
N
1.00
1.01
2.27
0.96
4.07
4.12
2.57
9.0 8.5 8.0 7.5 7.0 6.5 6.0 5.5 5.0 4.5 4.0 3.5 3.0 2.5 2.0 1.5 1.0 0.5 0.0
f1 (ppm)

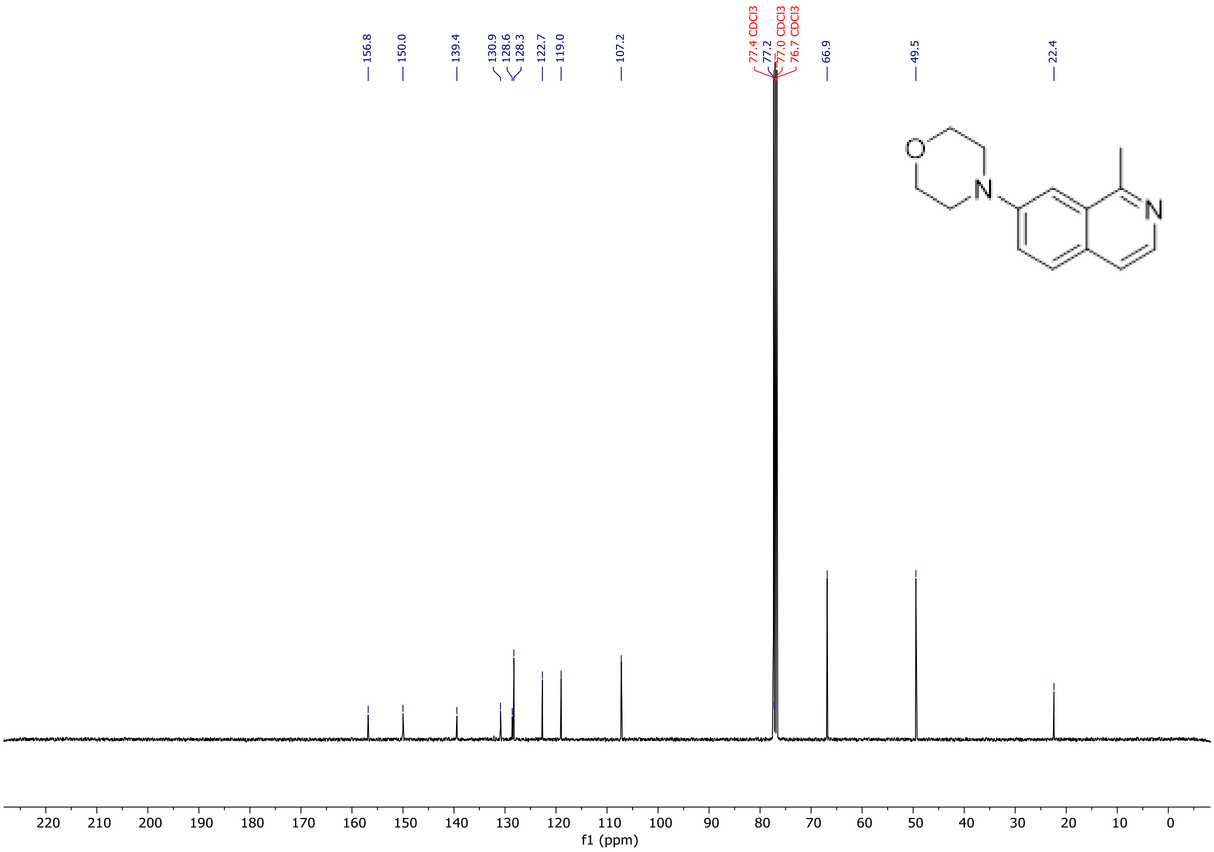

156.8
150.0
139.4
130.9
128.6
128.3
122.7
119.0
107.2
77.4 CDCl3
77.2
77.0 CDCl3
76.7 CDCl3
66.9
49.5
22.4
O
N
N
220 210 200 190 180 170 160 150 140 130 120 110 100 90 80 70 60 50 40 30 20 10 0
f1 (ppm)

7j

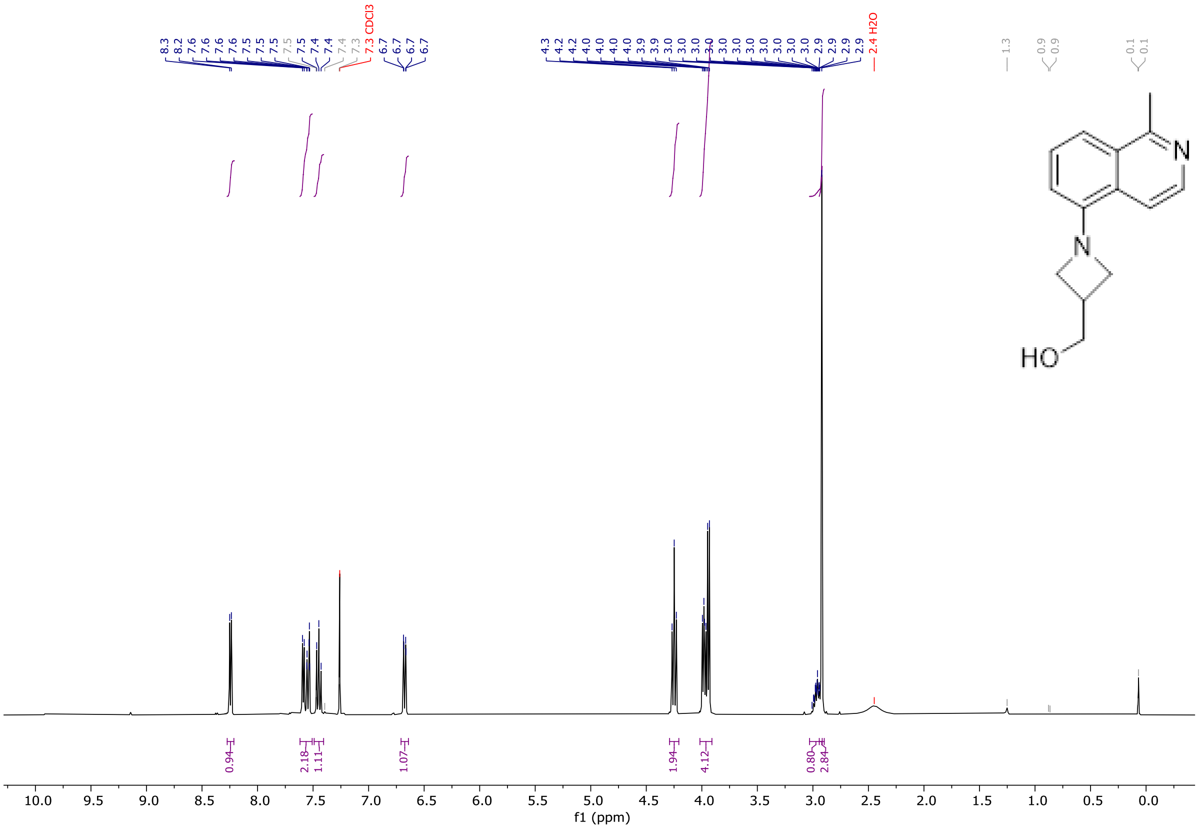

8.3
8.2
7.6
7.6
7.6
7.6
7.5
7.5
7.5
7.5
7.5
7.4
7.4
7.4
7.3
7.3 CDCl3
6.7
6.7
6.7
6.7
4.3
4.2
4.2
4.0
4.0
4.0
4.0
3.9
3.9
3.0
3.0
3.0
3.0
3.0
3.0
3.0
3.0
3.0
3.0
3.0
2.9
2.9
2.9
2.9
2.4 H2O
1.3
0.9
0.9
0.1
0.1
N
N
HO
0.94
2.18
1.11
1.07
1.94
4.12
0.80
2.84
10.0
9.5
9.0
8.5
8.0
7.5
7.0
6.5
6.0
5.5
5.0
4.5
4.0
3.5
3.0
2.5
2.0
1.5
1.0
0.5
0.0
f1 (ppm)

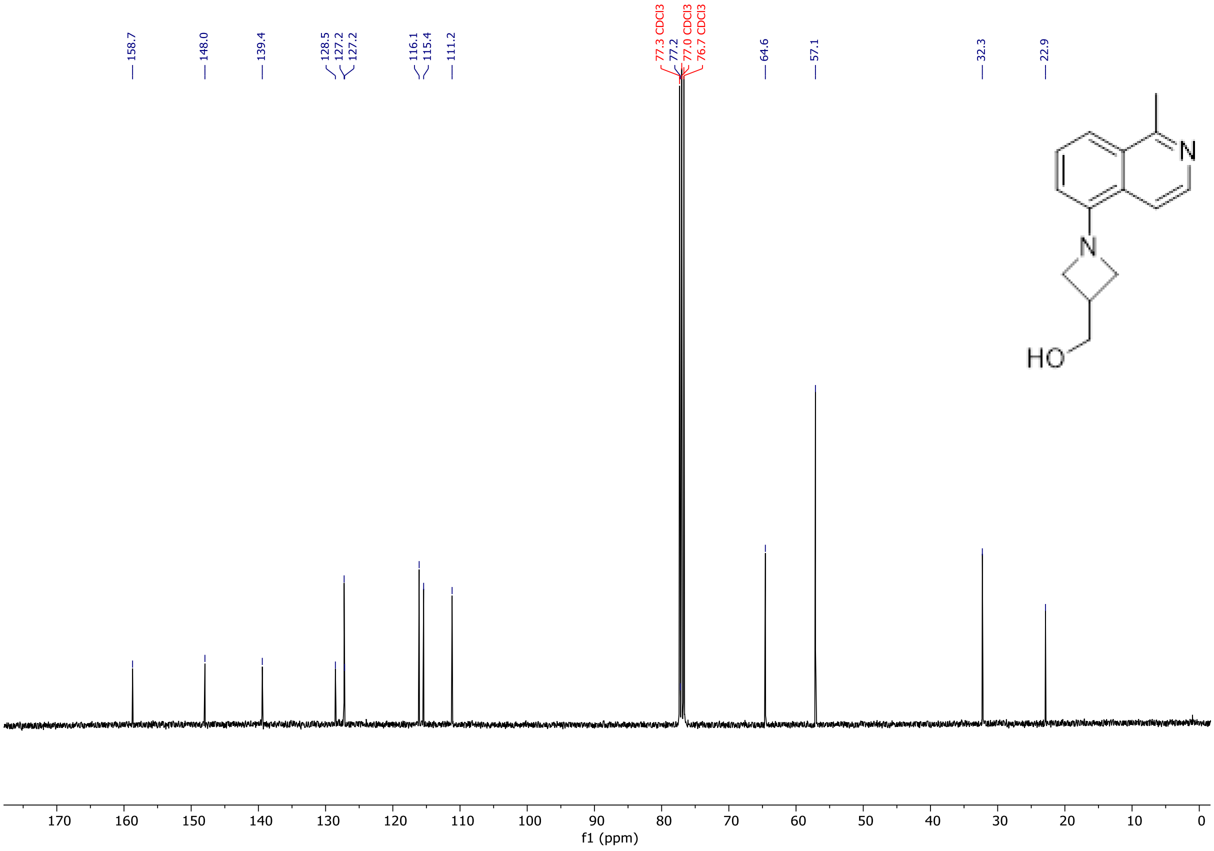

158.7
148.0
139.4
128.5
127.2
127.2
116.1
115.4
111.2
77.3 CDCl3
77.2
77.0 CDCl3
76.7 CDCl3
64.6
57.1
32.3
22.9
N
N
HO
170
160
150
140
130
120
110
100
90
80
70
60
50
40
30
20
10
0
f1 (ppm)

3a

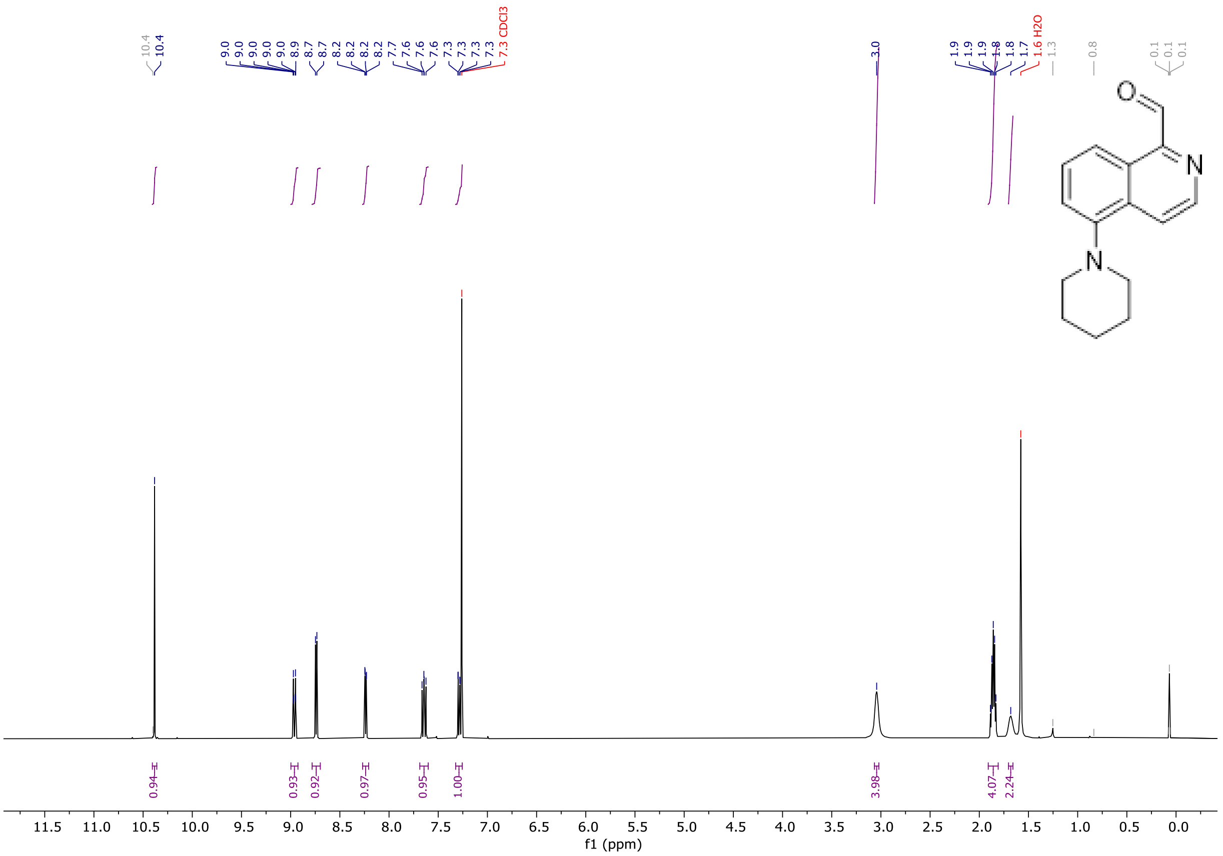

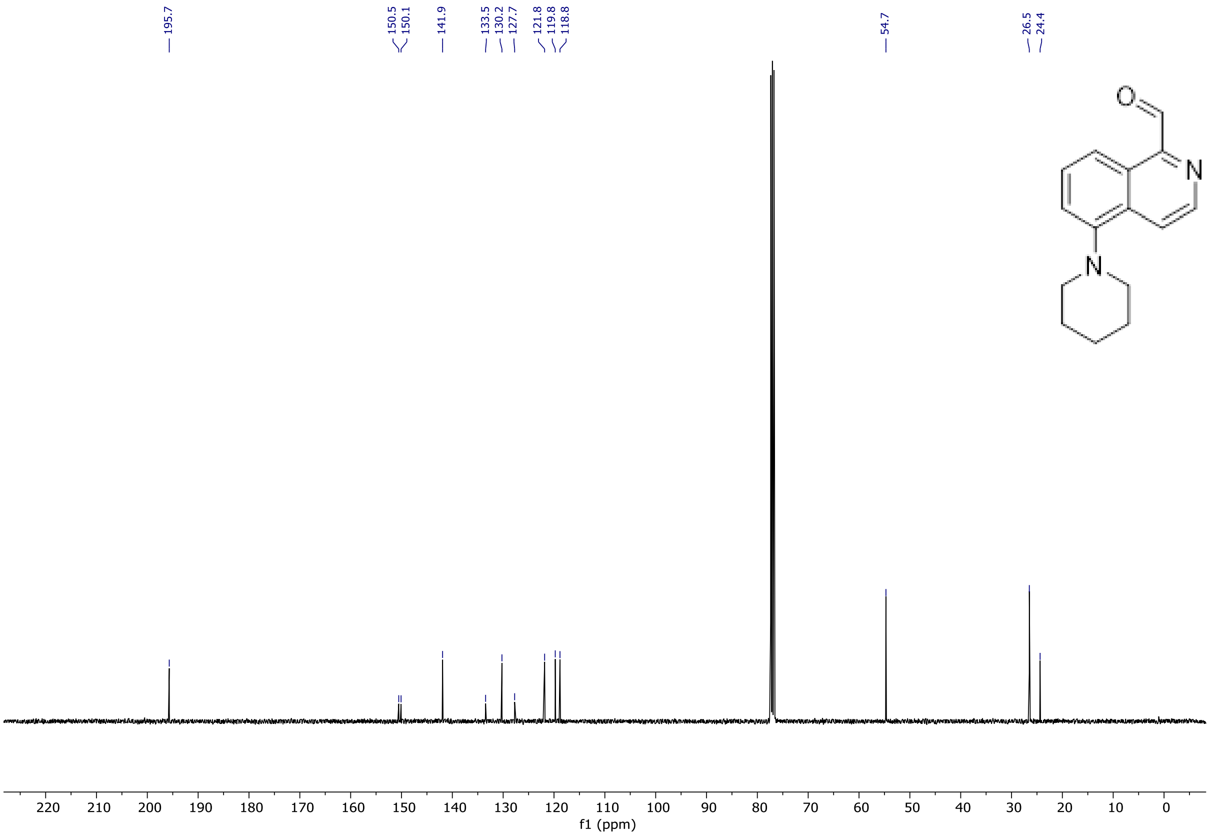

3b

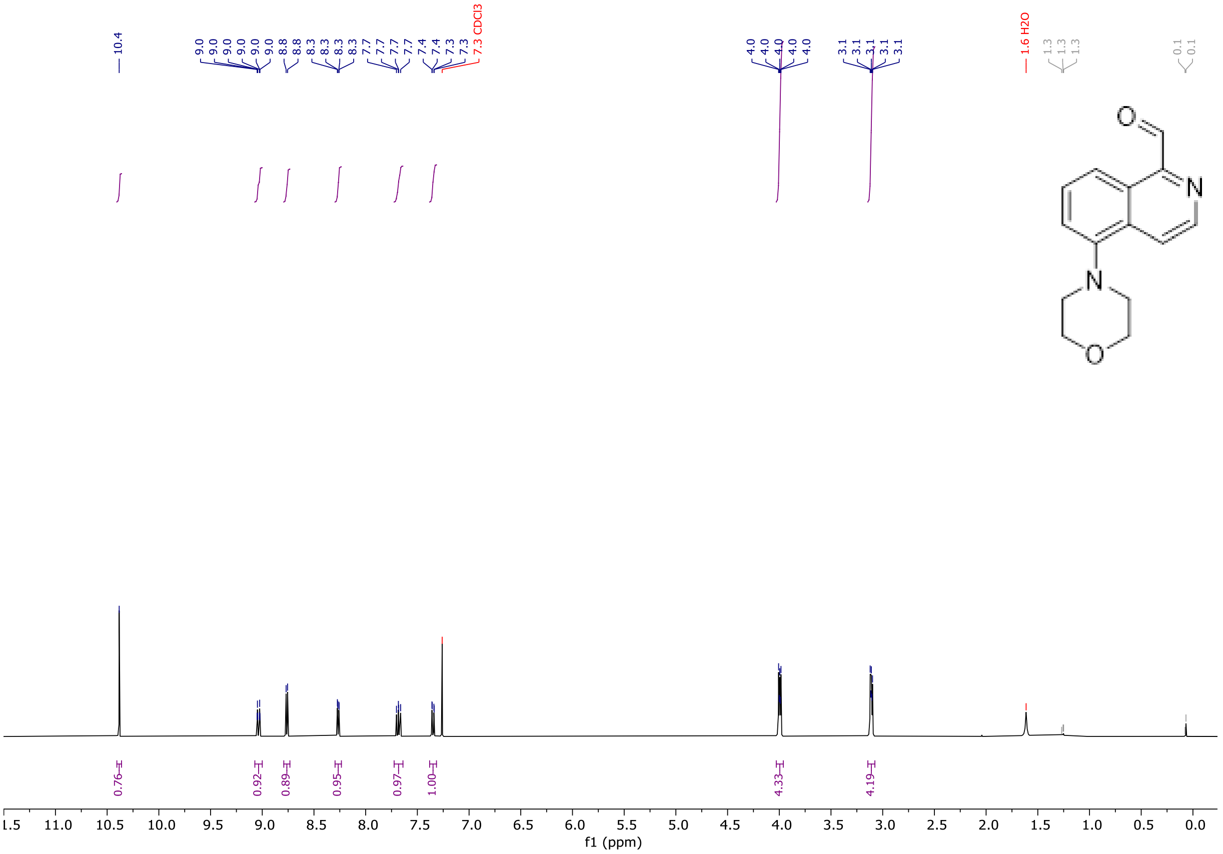
10.4
9.0
8.8
8.3
7.7
7.4
7.3
7.3 CDCl3
4.0
3.1
1.6 H2O
1.3
0.1
0.76
0.92
0.89
0.95
0.97
1.00
4.33
4.19
f1 (ppm)

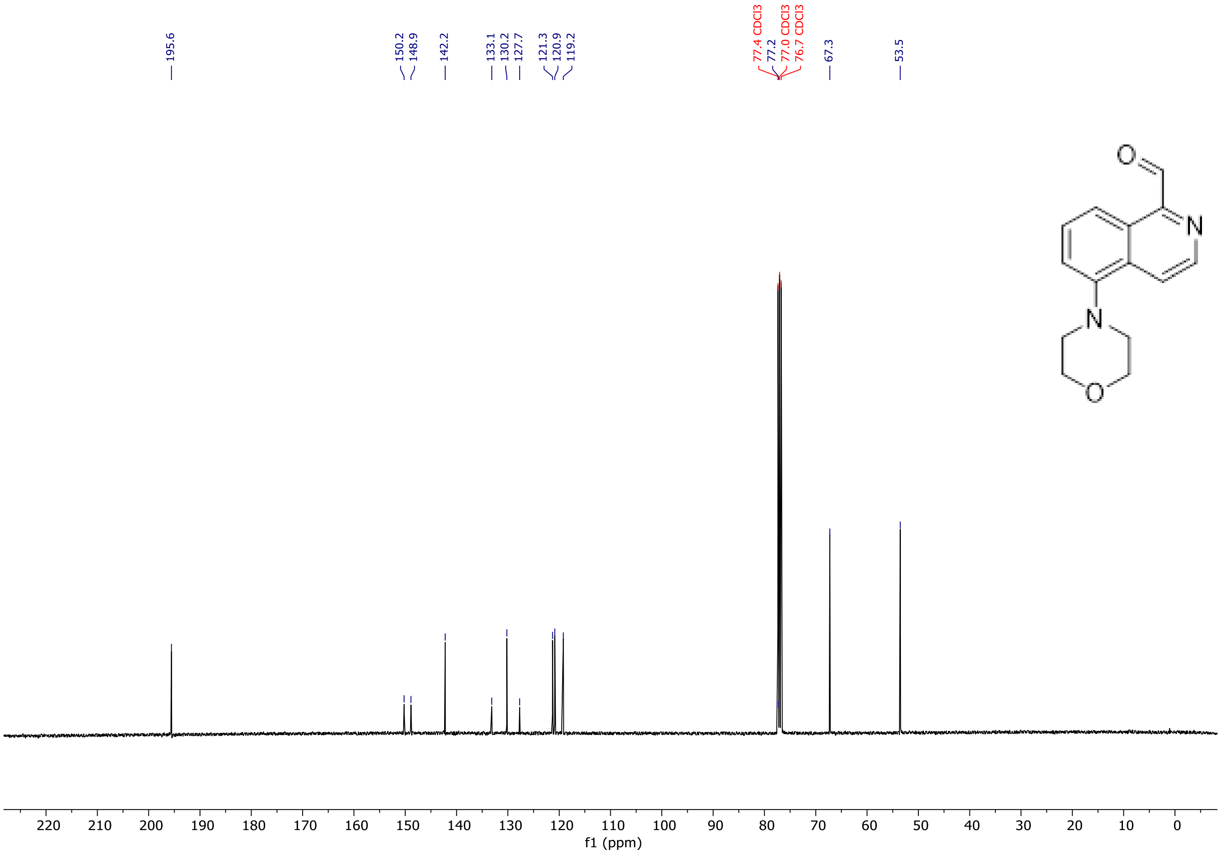
195.6
150.2
148.9
142.2
133.1
130.2
127.7
121.3
120.9
119.2
77.4 CDCl3
77.2
77.0 CDCl3
76.7 CDCl3
67.3
53.5
f1 (ppm)

3c

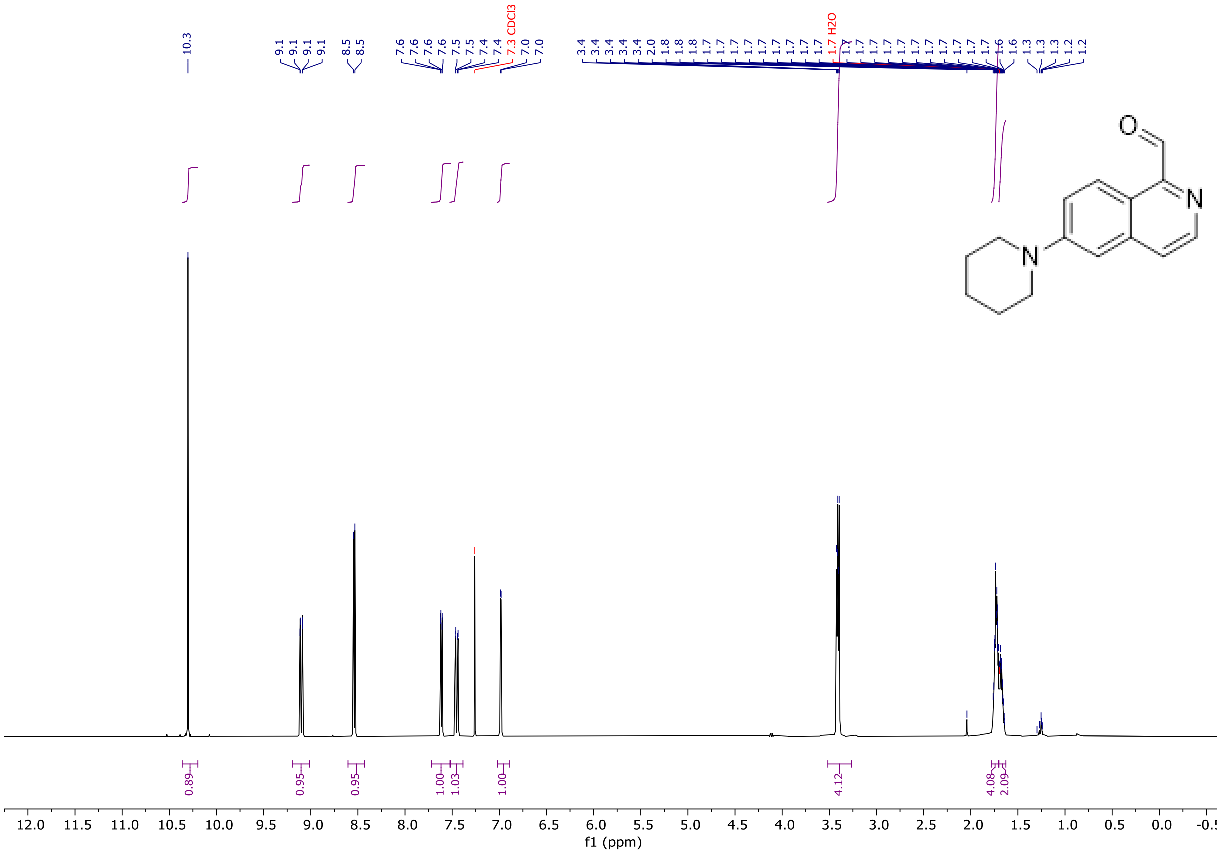

10.3
9.1
8.5
7.6
7.5
7.4
7.3 CDCl3
7.0
3.4
2.0
1.8
1.7
1.7 H2O
1.6
1.3
1.2
0.89
0.95
0.95
1.00
1.03
1.00
4.12
4.08
2.09
f1 (ppm)

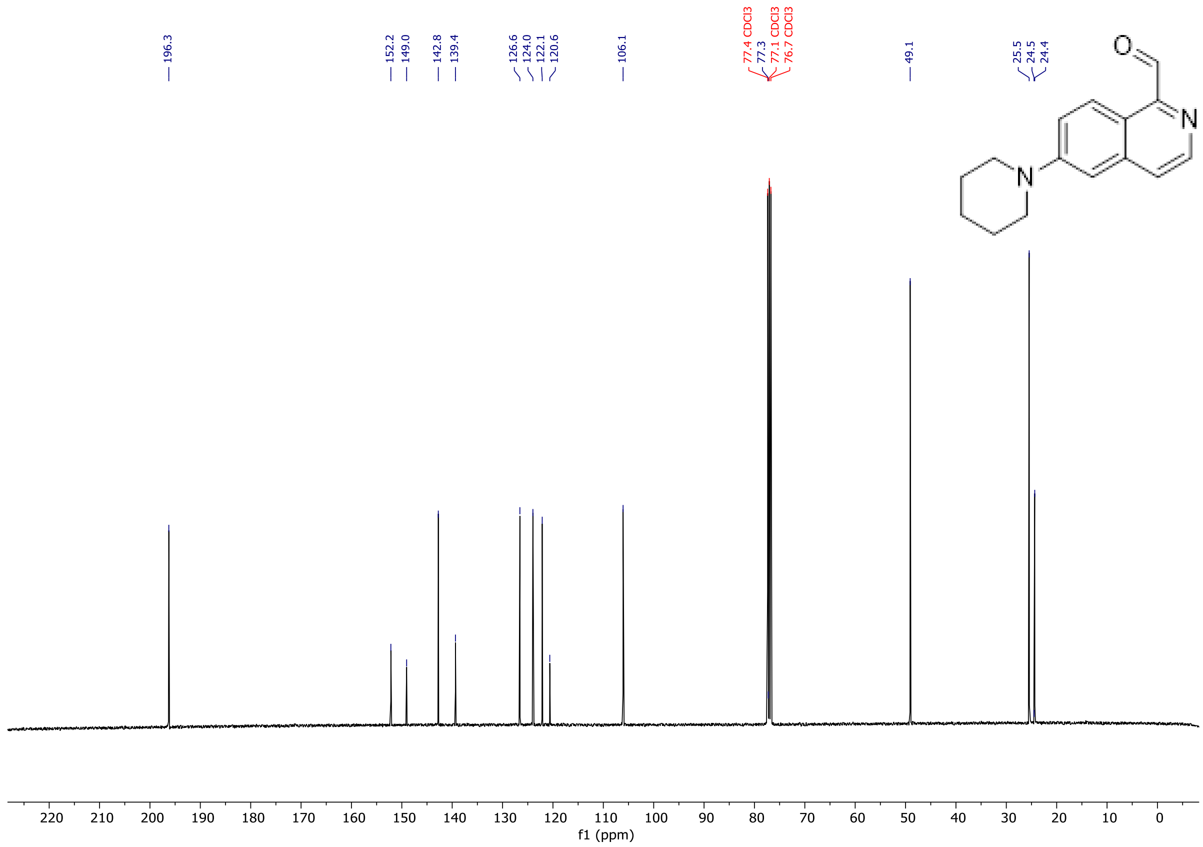

196.3
152.2
149.0
142.8
139.4
126.6
124.0
122.1
120.6
106.1
77.4 CDCl3
77.3
77.1 CDCl3
76.7 CDCl3
49.1
25.5
24.5
24.4
f1 (ppm)

3d

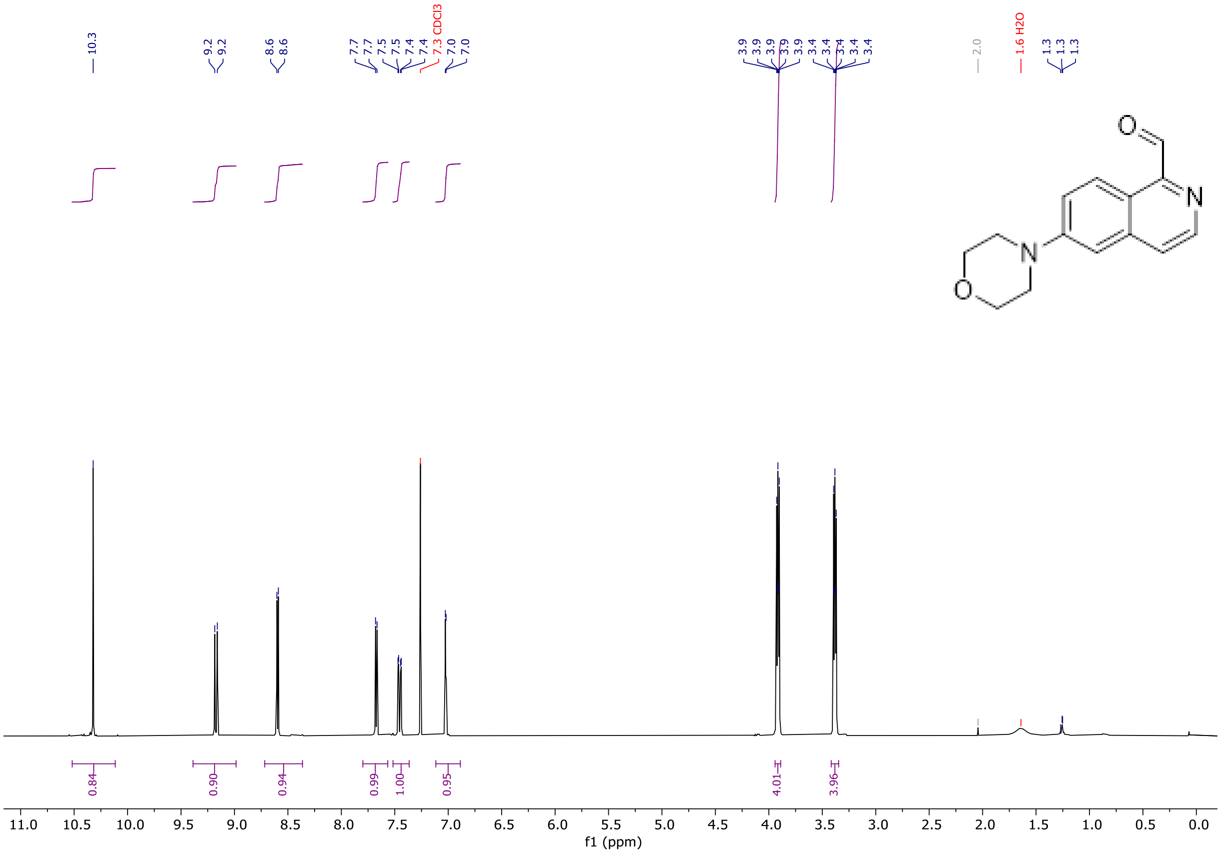

10.3
9.2
9.2
8.6
8.6
7.7
7.7
7.5
7.5
7.4
7.4
7.3 CDCl3
7.0
7.0
3.9
3.4
2.0
1.6 H2O
1.3
1.3
1.3
O
N
0.84
0.90
0.94
0.99
1.00
0.95
4.01
3.96
11.0 10.5 10.0 9.5 9.0 8.5 8.0 7.5 7.0 6.5 6.0 5.5 5.0 4.5 4.0 3.5 3.0 2.5 2.0 1.5 1.0 0.5 0.0
f1 (ppm)

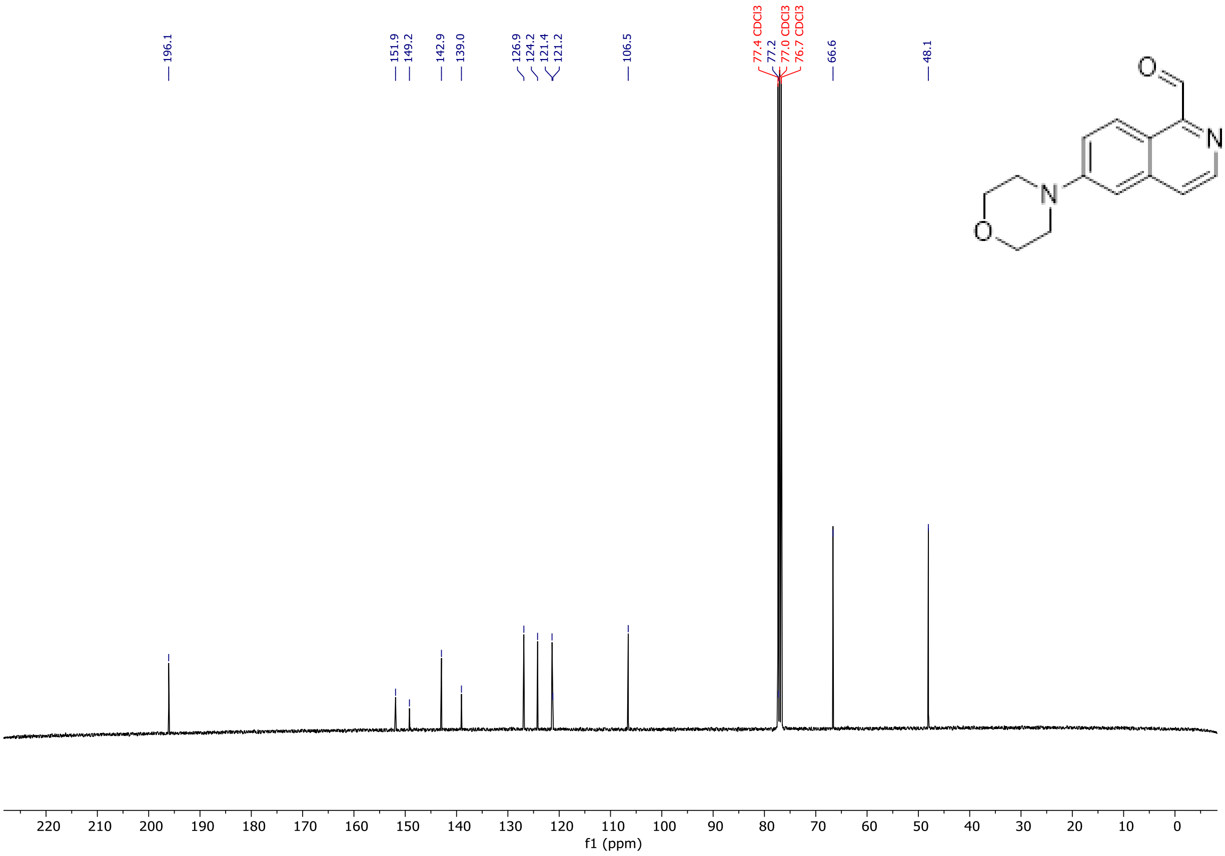

196.1
151.9
149.2
142.9
139.0
126.9
124.2
121.4
121.2
106.5
77.4 CDCl3
77.2
77.0 CDCl3
76.7 CDCl3
66.6
48.1
O
N
220 210 200 190 180 170 160 150 140 130 120 110 100 90 80 70 60 50 40 30 20 10 0
f1 (ppm)

3e

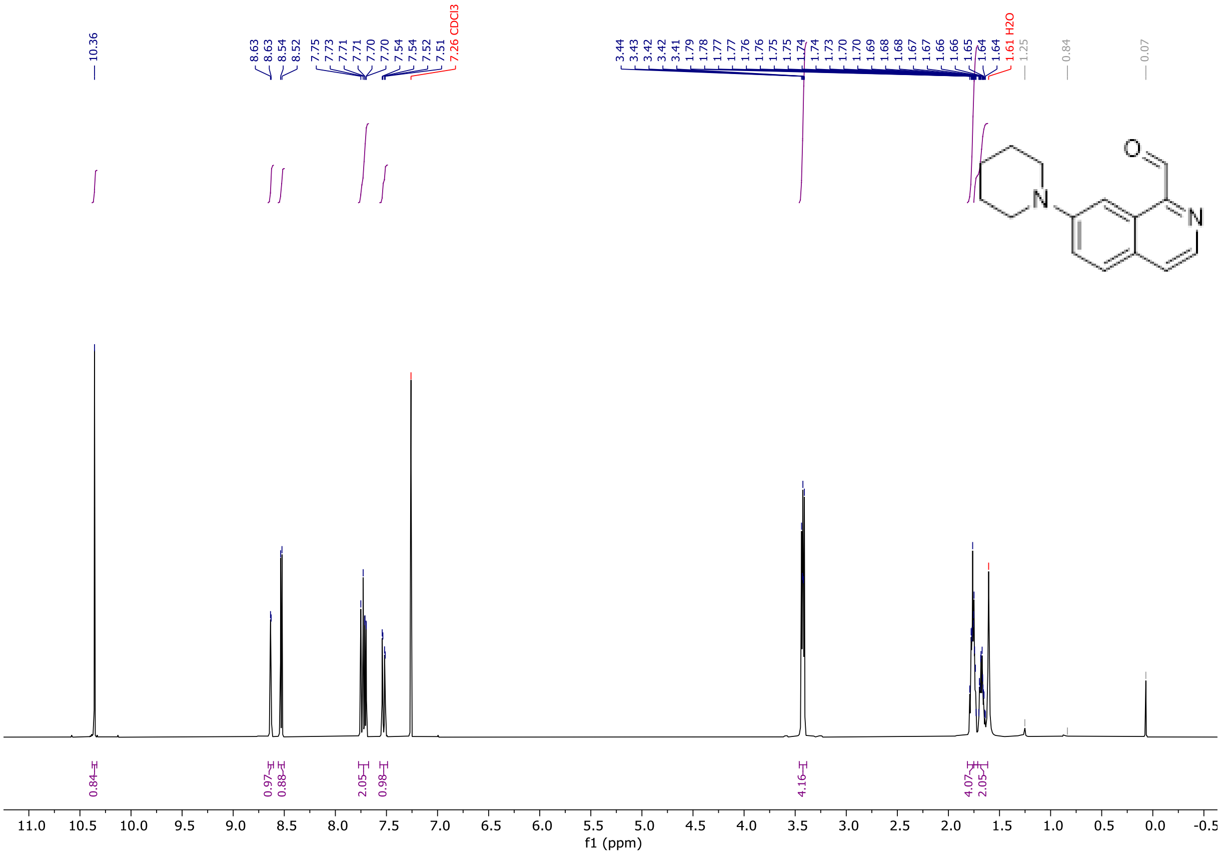

10.36
8.63
8.63
8.54
8.52
7.75
7.73
7.71
7.71
7.70
7.70
7.54
7.54
7.52
7.51
7.26 CDCl3
3.44
3.43
3.42
3.42
3.41
1.79
1.78
1.77
1.77
1.76
1.76
1.75
1.75
1.74
1.74
1.73
1.70
1.70
1.69
1.68
1.68
1.67
1.67
1.66
1.66
1.65
1.64
1.64
1.61 H2O
1.25
0.84
0.07
0.84
0.97
0.88
2.05
0.98
4.16
4.07
2.05
O
N
N
f1 (ppm)

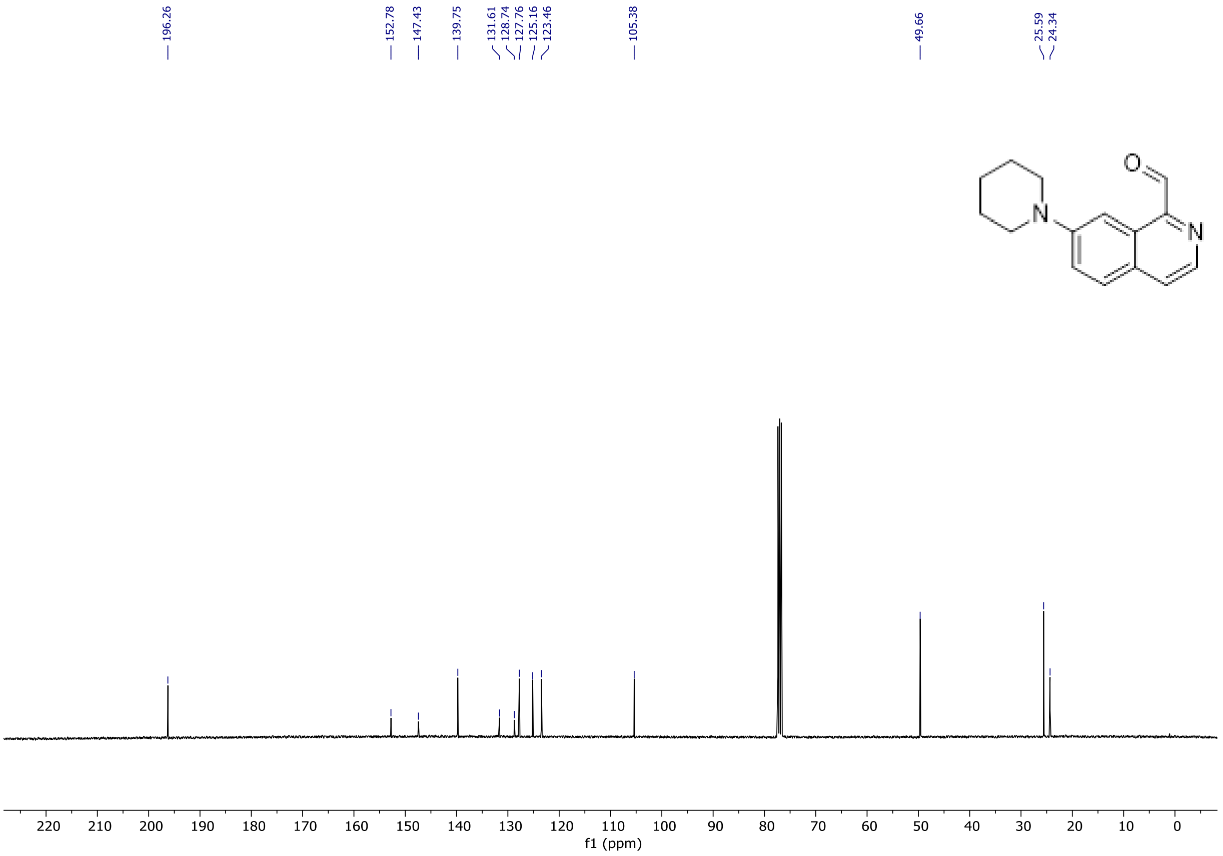

196.26
152.78
147.43
139.75
131.61
128.74
127.76
125.16
123.46
105.38
49.66
25.59
24.34
O
N
N
f1 (ppm)

3f

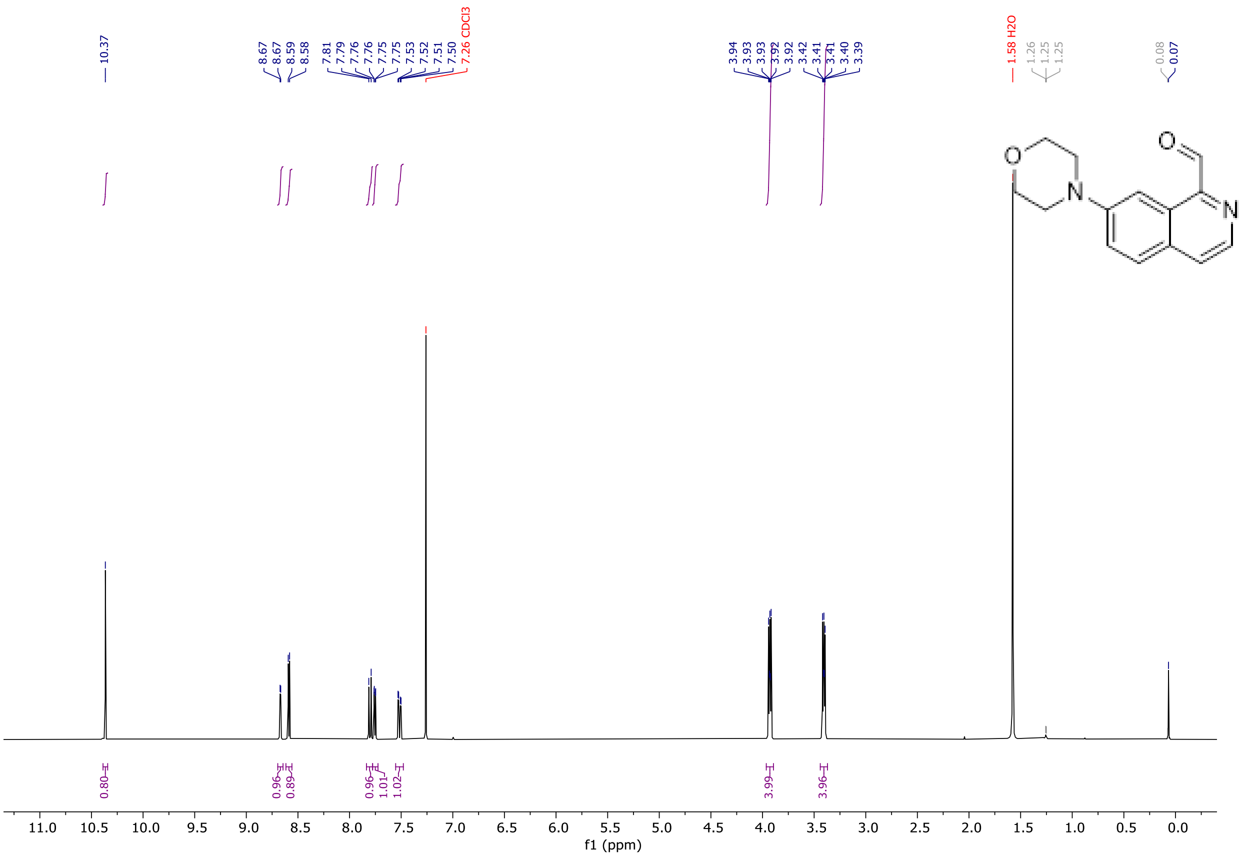
10.37
8.67
8.67
8.59
8.58
7.81
7.79
7.76
7.76
7.75
7.75
7.53
7.52
7.51
7.50
7.26 CDCl3
3.94
3.93
3.93
3.92
3.92
3.42
3.41
3.41
3.40
3.39
1.58 H2O
1.26
1.25
1.25
0.08
0.07
0.80
0.96
0.89
0.96
1.01
1.02
3.99
3.96
f1 (ppm)

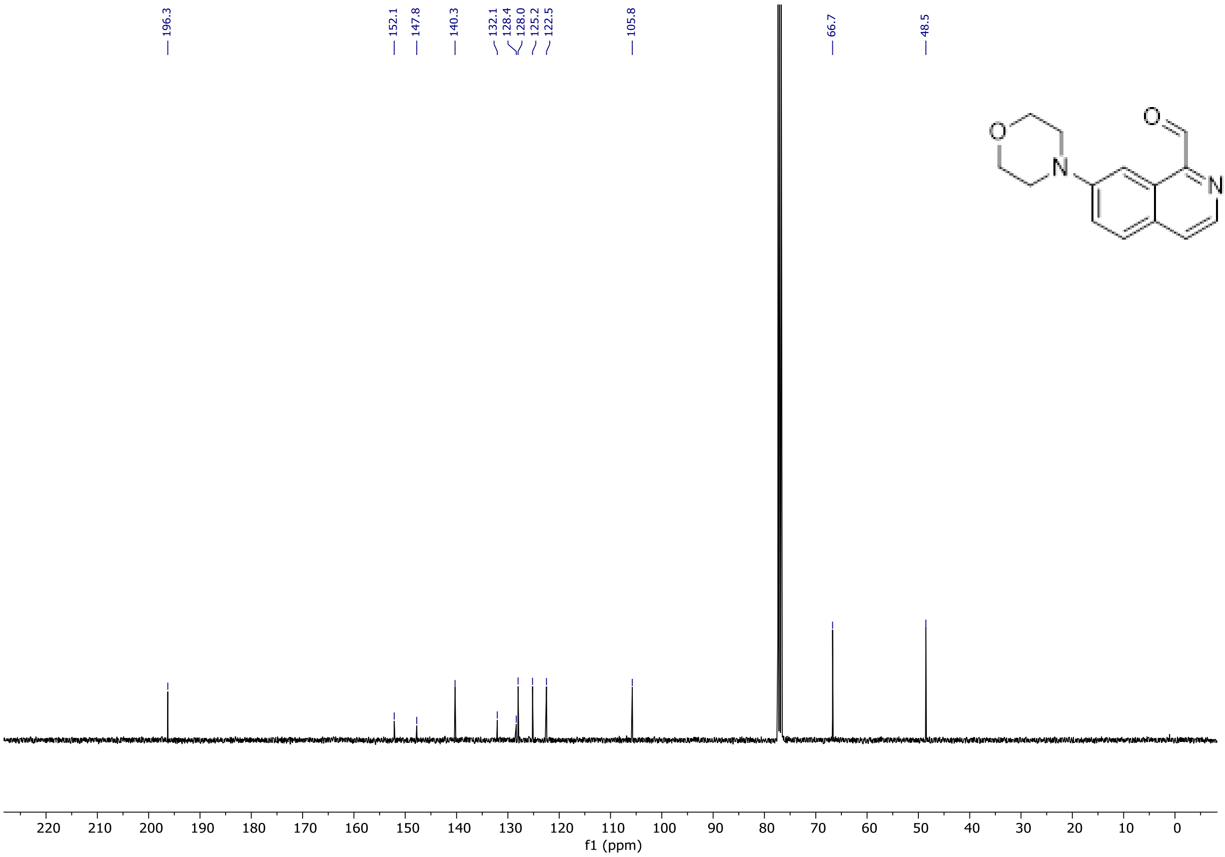
196.3
152.1
147.8
140.3
132.1
128.4
128.0
125.2
122.5
105.8
66.7
48.5
f1 (ppm)

3g

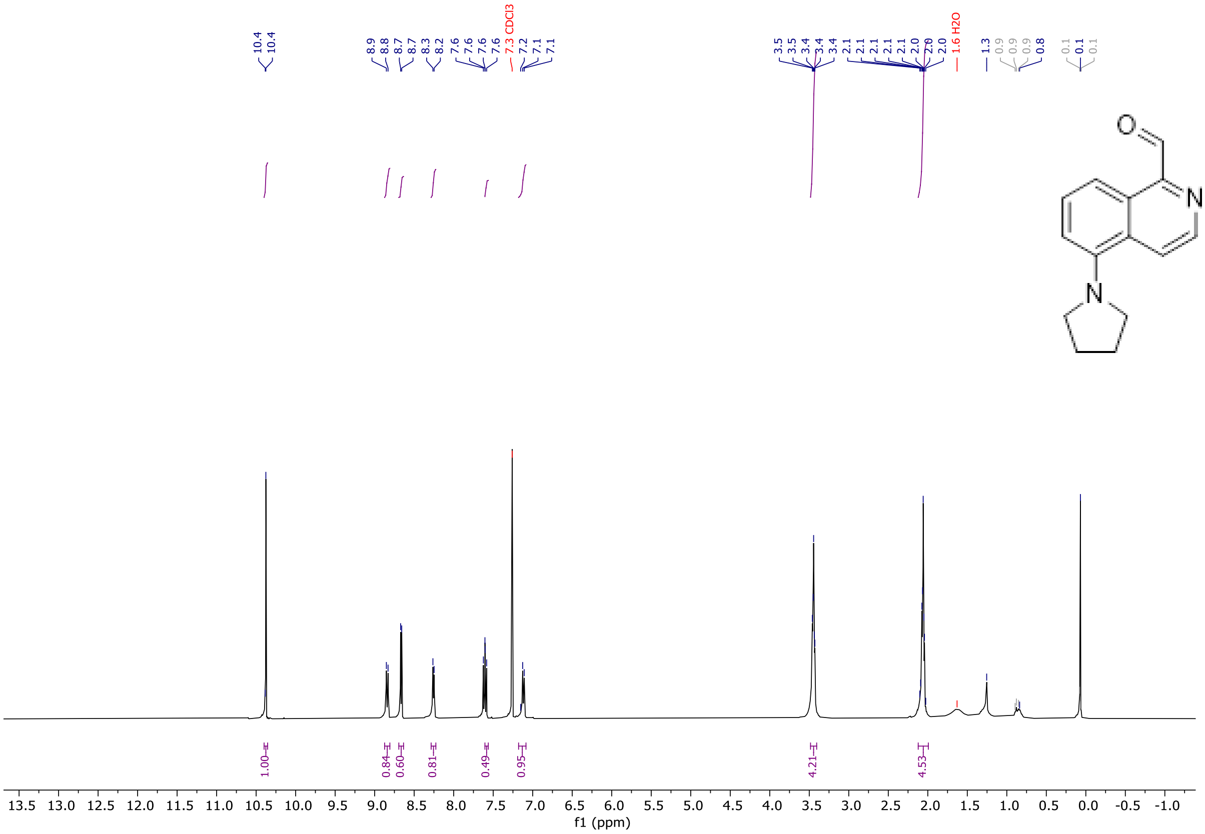
10.4
10.4
8.9
8.8
8.7
8.7
8.3
8.2
7.6
7.6
7.6
7.6
7.3 CDCl3
7.2
7.1
7.1
3.5
3.5
3.4
3.4
3.4
2.1
2.1
2.1
2.1
2.1
2.0
2.0
2.0
1.6 H2O
1.3
0.9
0.9
0.9
0.8
0.1
0.1
0.1
1.00
0.84
0.60
0.81
0.49
0.95
4.21
4.53
f1 (ppm)
O
N
N

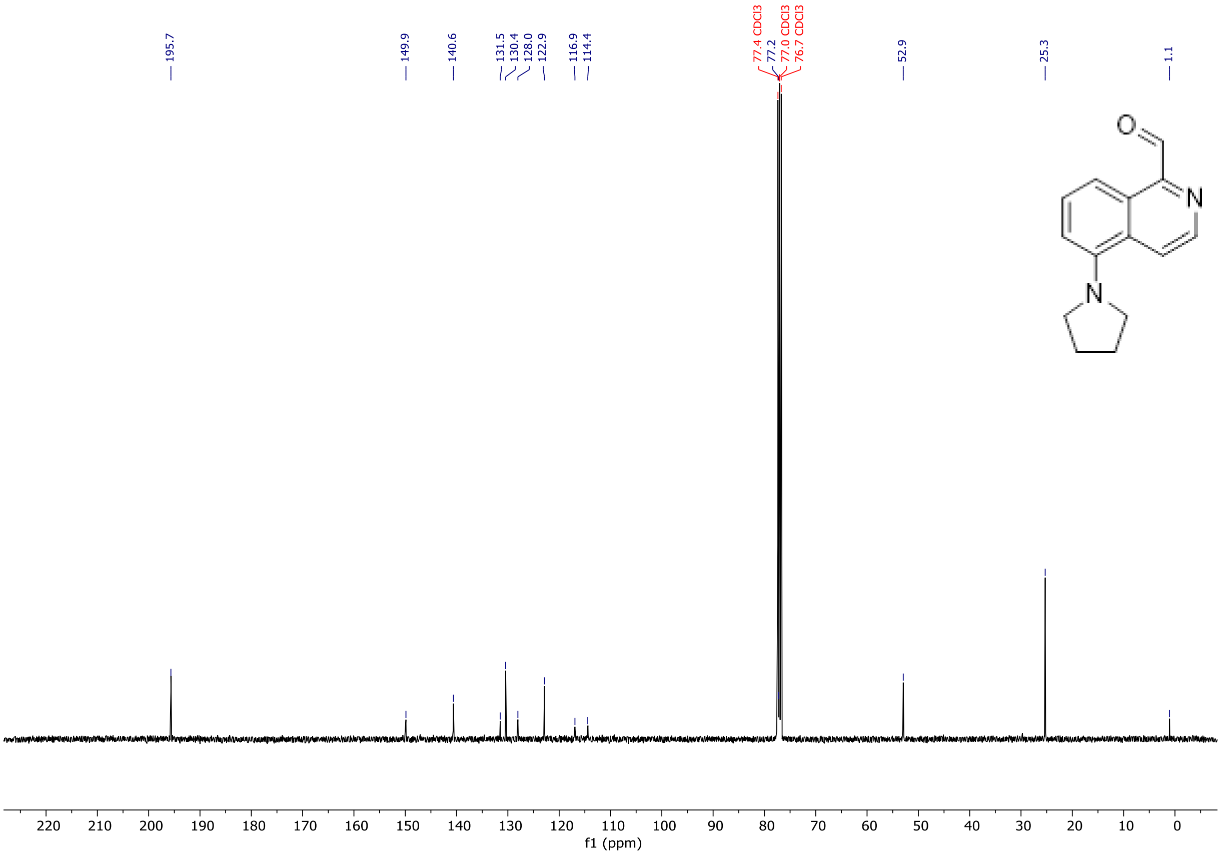
195.7
149.9
140.6
131.5
130.4
128.0
122.9
116.9
114.4
77.4 CDCl3
77.2
77.0 CDCl3
76.7 CDCl3
52.9
25.3
1.1
f1 (ppm)
O
N
N

3h

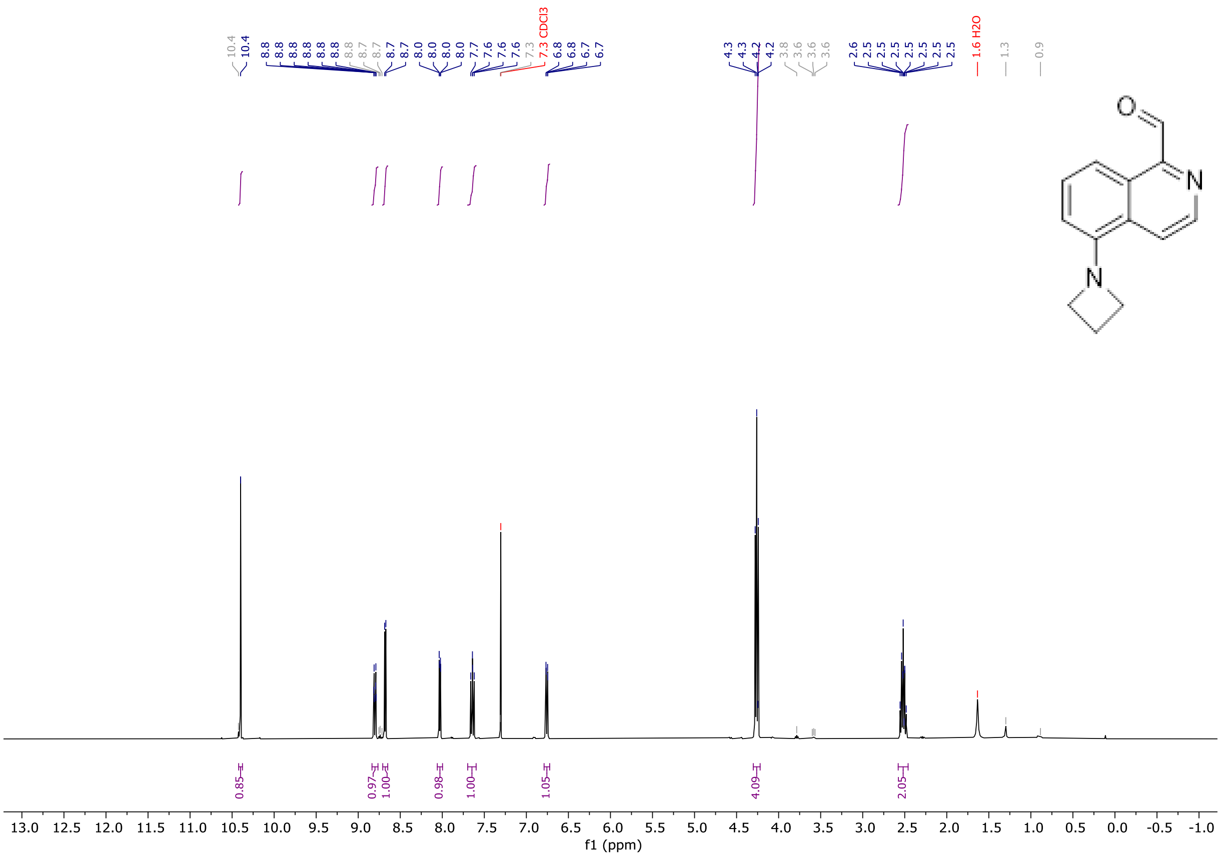

10.4
10.4
8.8
8.8
8.8
8.8
8.8
8.8
8.8
8.7
8.7
8.7
8.0
8.0
8.0
8.0
7.7
7.6
7.6
7.3
7.3 CDCl3
6.8
6.8
6.7
6.7
4.3
4.3
4.2
4.2
3.8
3.6
3.6
3.6
2.6
2.5
2.5
2.5
2.5
2.5
2.5
2.5
1.6 H2O
1.3
0.9
N
O
0.85
0.97
1.00
0.98
1.00
1.05
4.09
2.05
13.0 12.5 12.0 11.5 11.0 10.5 10.0 9.5 9.0 8.5 8.0 7.5 7.0 6.5 6.0 5.5 5.0 4.5 4.0 3.5 3.0 2.5 2.0 1.5 1.0 0.5 0.0 -0.5 -1.0
f1 (ppm)

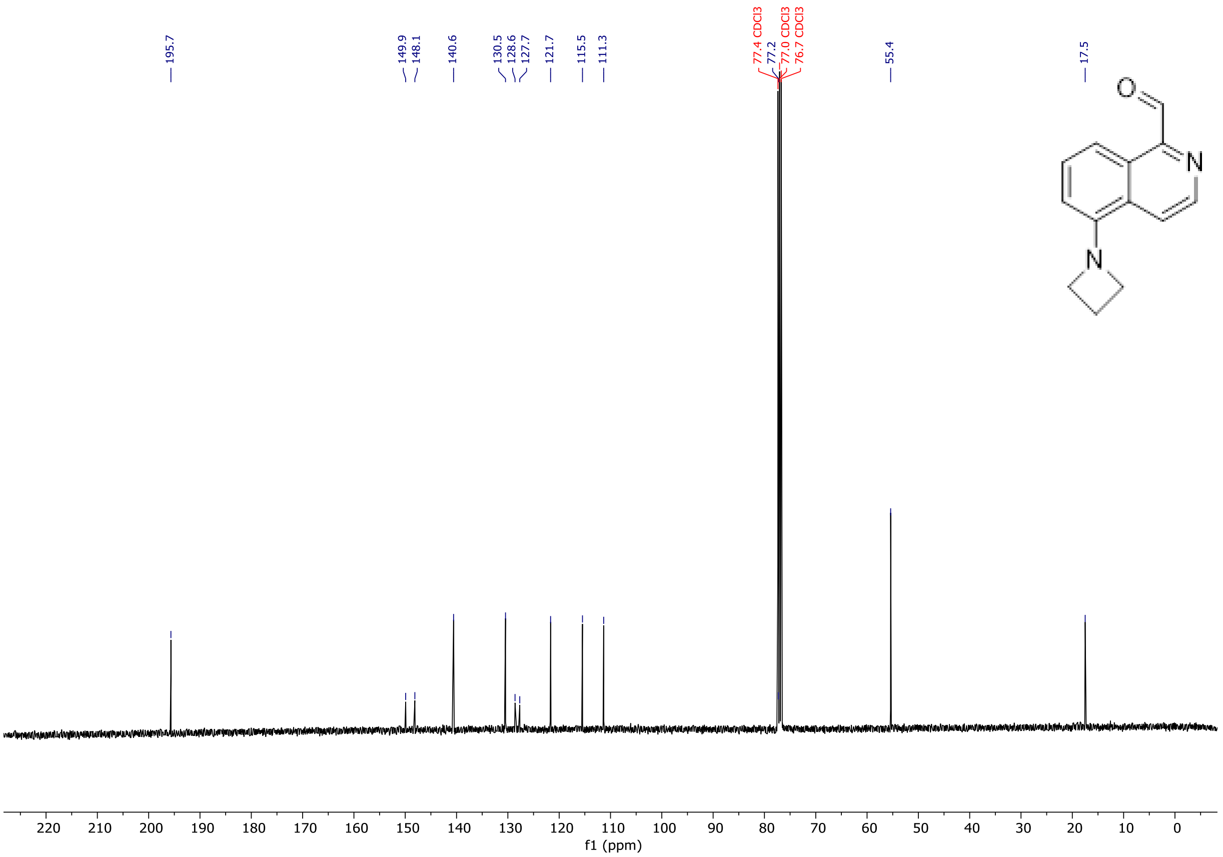

195.7
149.9
148.1
140.6
130.5
128.6
127.7
121.7
115.5
111.3
77.4 CDCl3
77.2
77.0 CDCl3
76.7 CDCl3
55.4
17.5
N
O
220 210 200 190 180 170 160 150 140 130 120 110 100 90 80 70 60 50 40 30 20 10 0
f1 (ppm)

3i

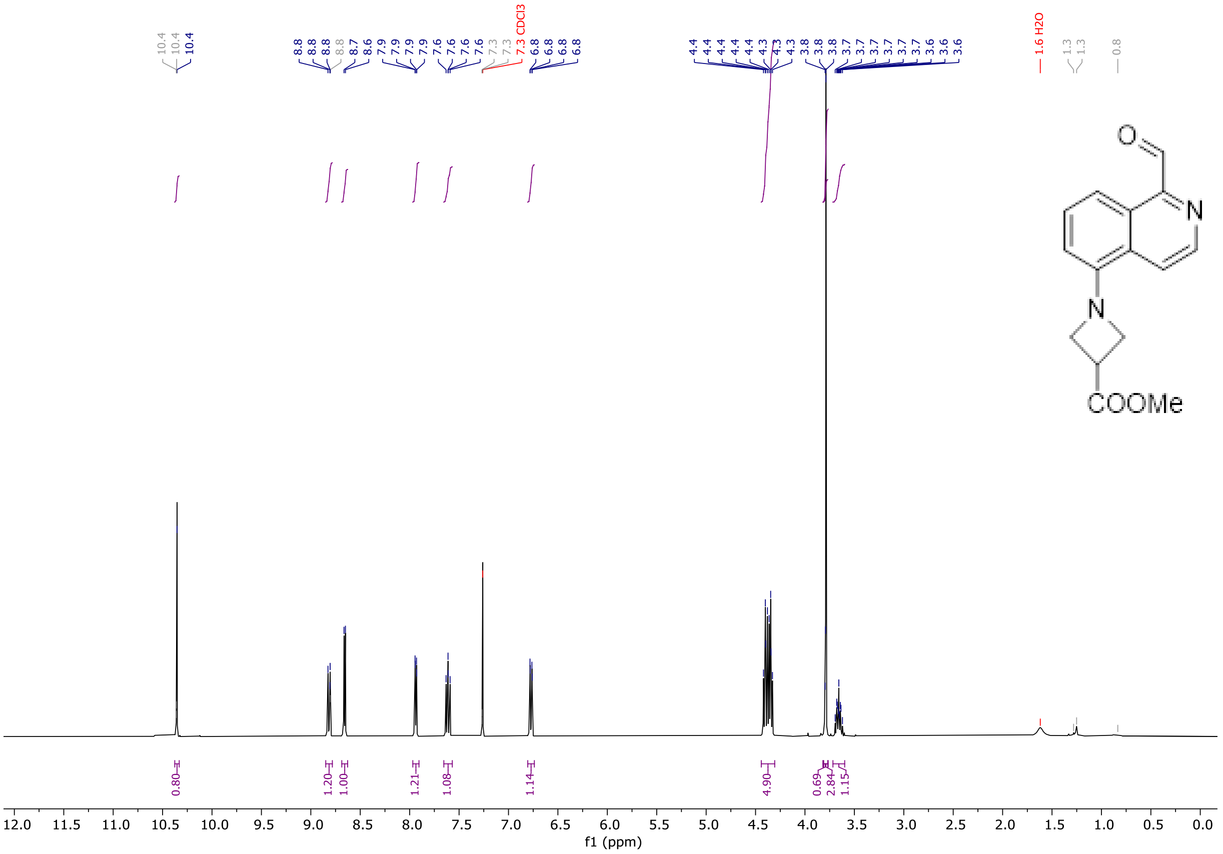
10.4
10.4
10.4
8.8
8.8
8.8
8.8
8.7
8.6
7.9
7.9
7.9
7.9
7.6
7.6
7.6
7.6
7.3
7.3
7.3 CDCl3
6.8
6.8
6.8
6.8
4.4
4.4
4.4
4.4
4.4
4.3
4.3
4.3
3.8
3.8
3.8
3.7
3.7
3.7
3.7
3.7
3.7
3.6
3.6
3.6
1.6 H2O
1.3
1.3
0.8
0.80
1.20
1.00
1.21
1.08
1.14
4.90
0.69
2.84
1.15
O
N
N
COOMe
f1 (ppm)

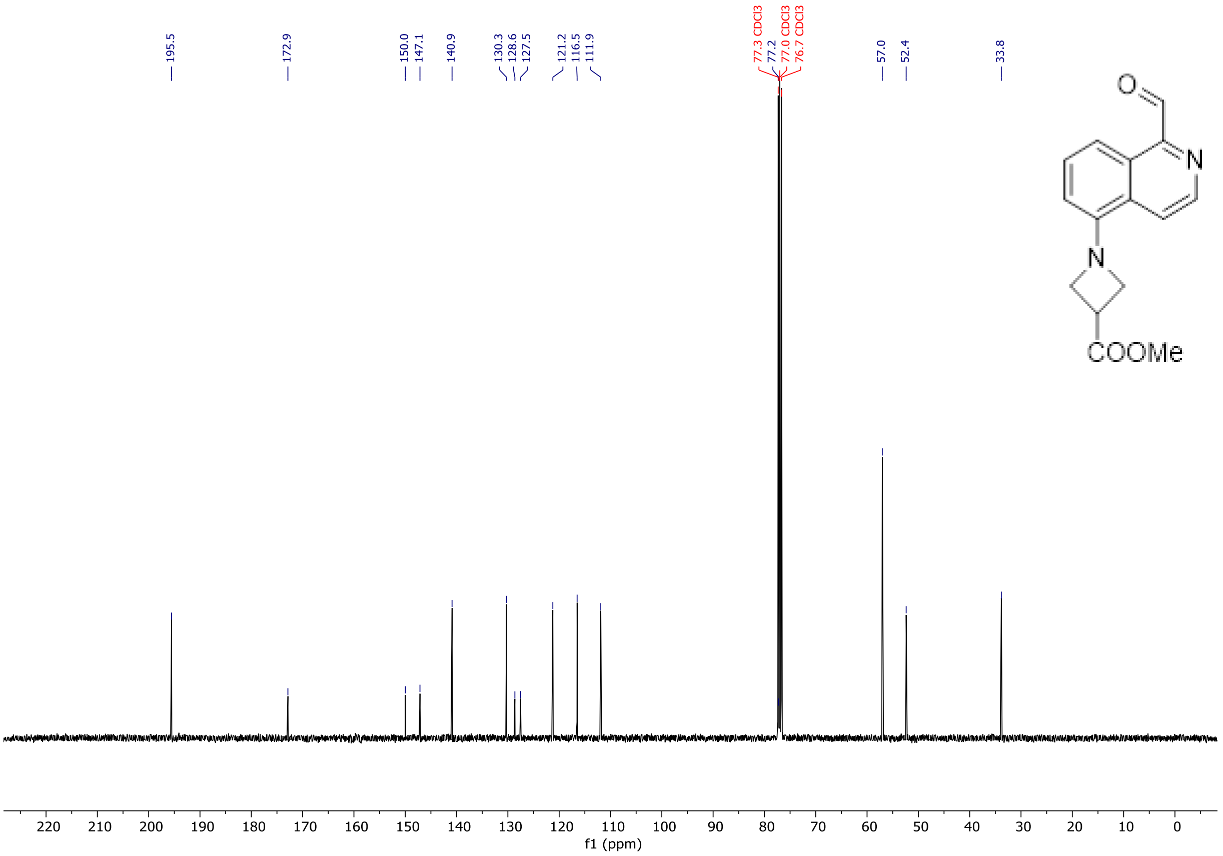
195.5
172.9
150.0
147.1
140.9
130.3
128.6
127.5
121.2
116.5
111.9
77.3 CDCl3
77.2
77.0 CDCl3
76.7 CDCl3
57.0
52.4
33.8
O
N
N
COOMe
f1 (ppm)

3j

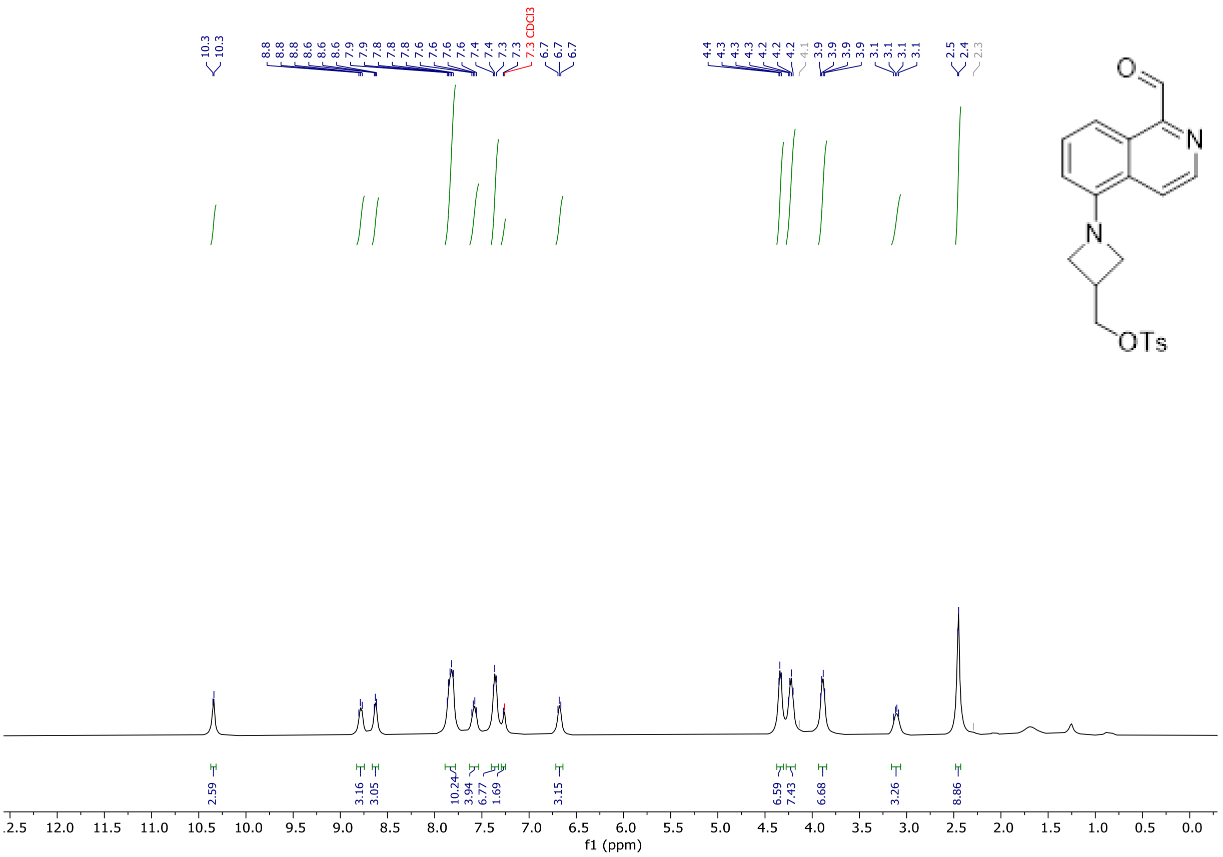
10.3
10.3
8.8
8.6
7.9
7.8
7.6
7.4
7.3
7.3 CDCl3
6.7
4.4
4.3
4.2
4.1
3.9
3.1
2.5
2.4
2.3
2.59
3.16
3.05
10.24
3.94
6.77
1.69
3.15
6.59
7.43
6.68
3.26
8.86
f1 (ppm)
O
N
OTs

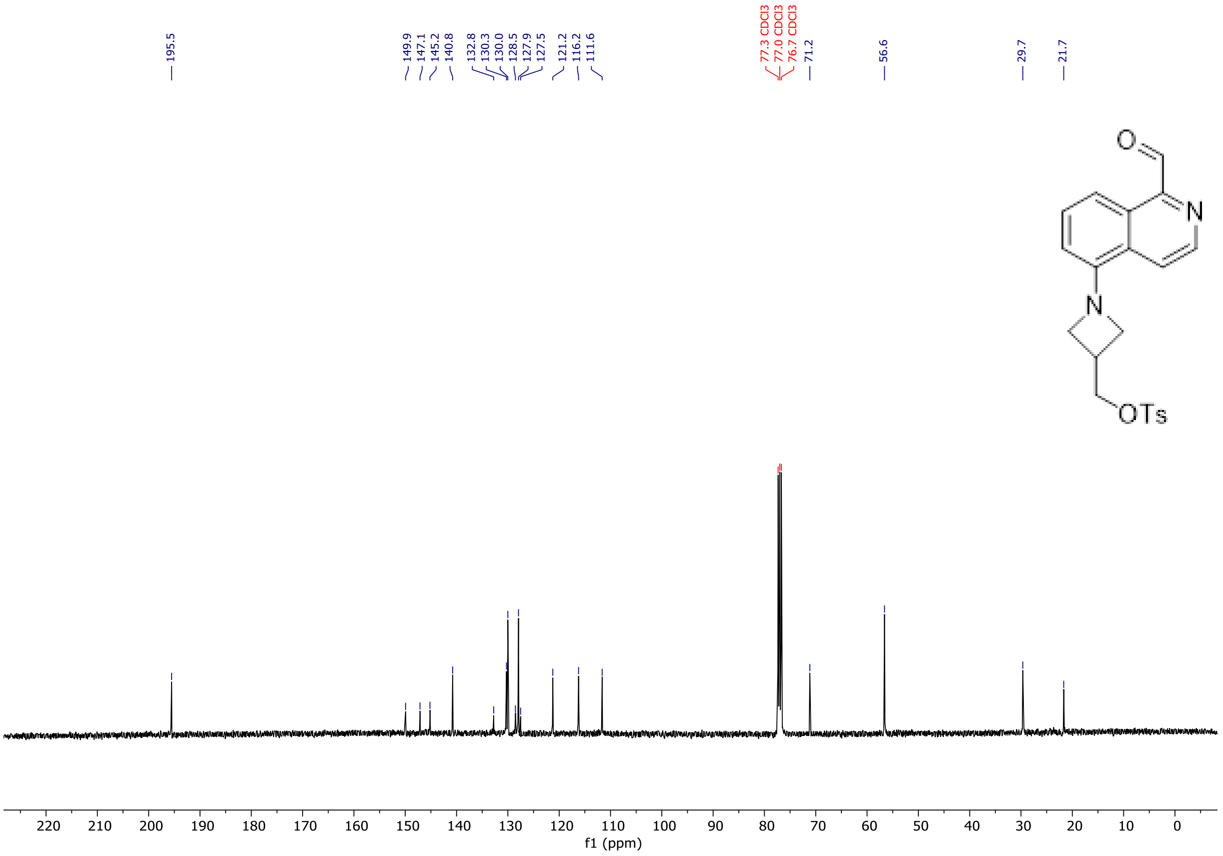
195.5
149.9
147.1
145.2
140.8
132.8
130.3
130.0
128.5
127.9
127.5
121.2
116.2
111.6
77.3 CDCl3
77.0 CDCl3
76.7 CDCl3
71.2
56.6
29.7
21.7
f1 (ppm)
O
N
OTs

3l

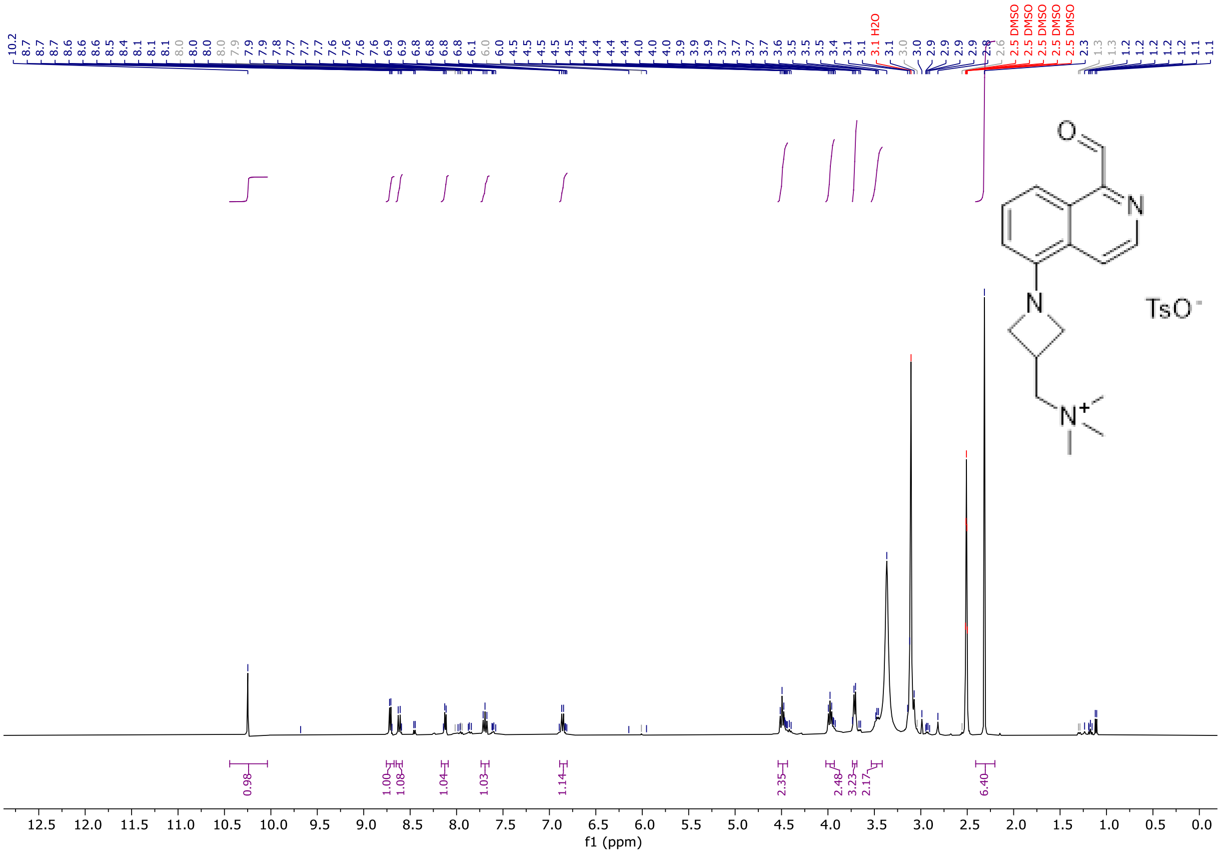
3.1 H2O
2.5 DMSO
TsO⁻
0.98
1.00
1.08
1.04
1.03
1.14
2.35
2.48
3.23
2.17
6.40
f1 (ppm)

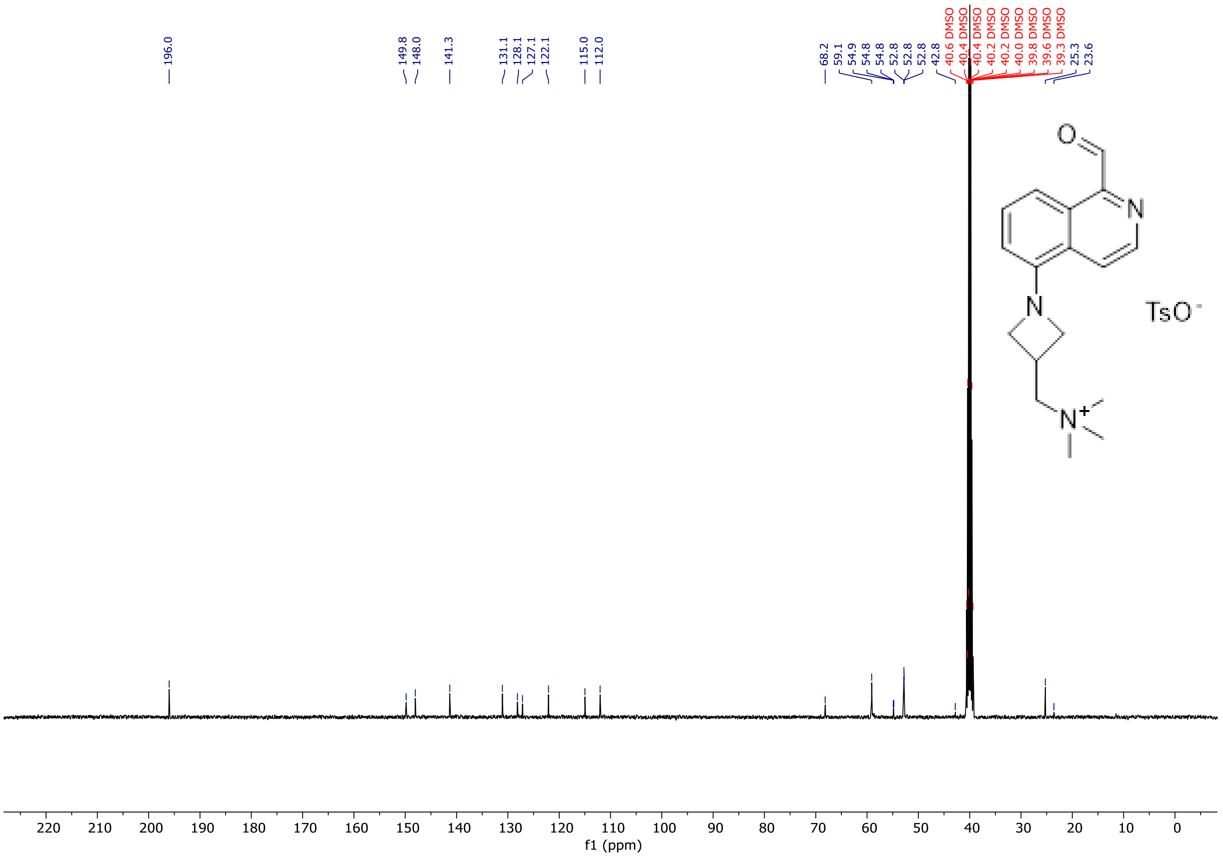
196.0
149.8
148.0
141.3
131.1
128.1
127.1
122.1
115.0
112.0
68.2
59.1
54.9
54.8
52.8
52.8
52.8
42.8
40.6 DMSO
40.4 DMSO
40.4 DMSO
40.2 DMSO
40.2 DMSO
40.0 DMSO
39.8 DMSO
39.6 DMSO
39.3 DMSO
25.3
23.6
TsO⁻
f1 (ppm)

3m

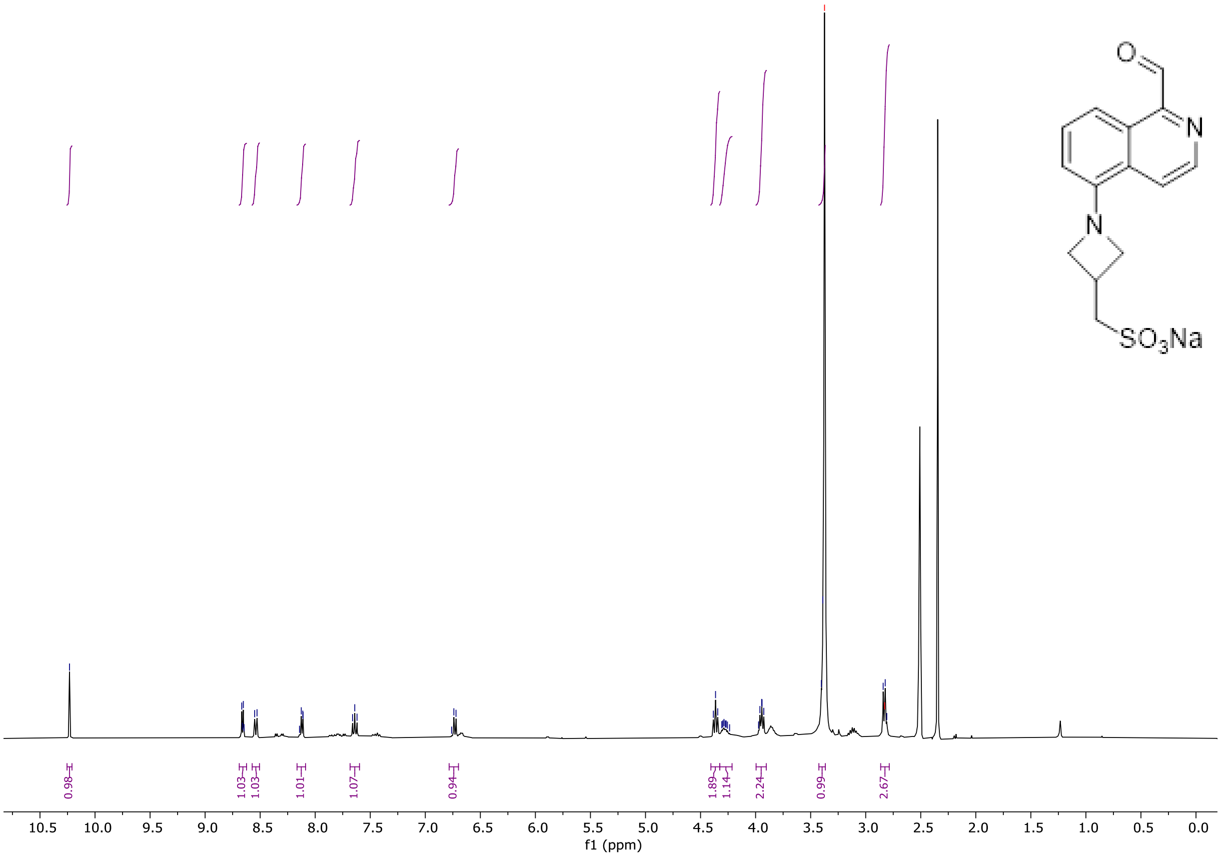

0.98
1.03
1.03
1.01
1.07
0.94
1.89
1.14
2.24
0.99
2.67
10.5 10.0 9.5 9.0 8.5 8.0 7.5 7.0 6.5 6.0 5.5 5.0 4.5 4.0 3.5 3.0 2.5 2.0 1.5 1.0 0.5 0.0
f1 (ppm)
O
N
N
SO3Na

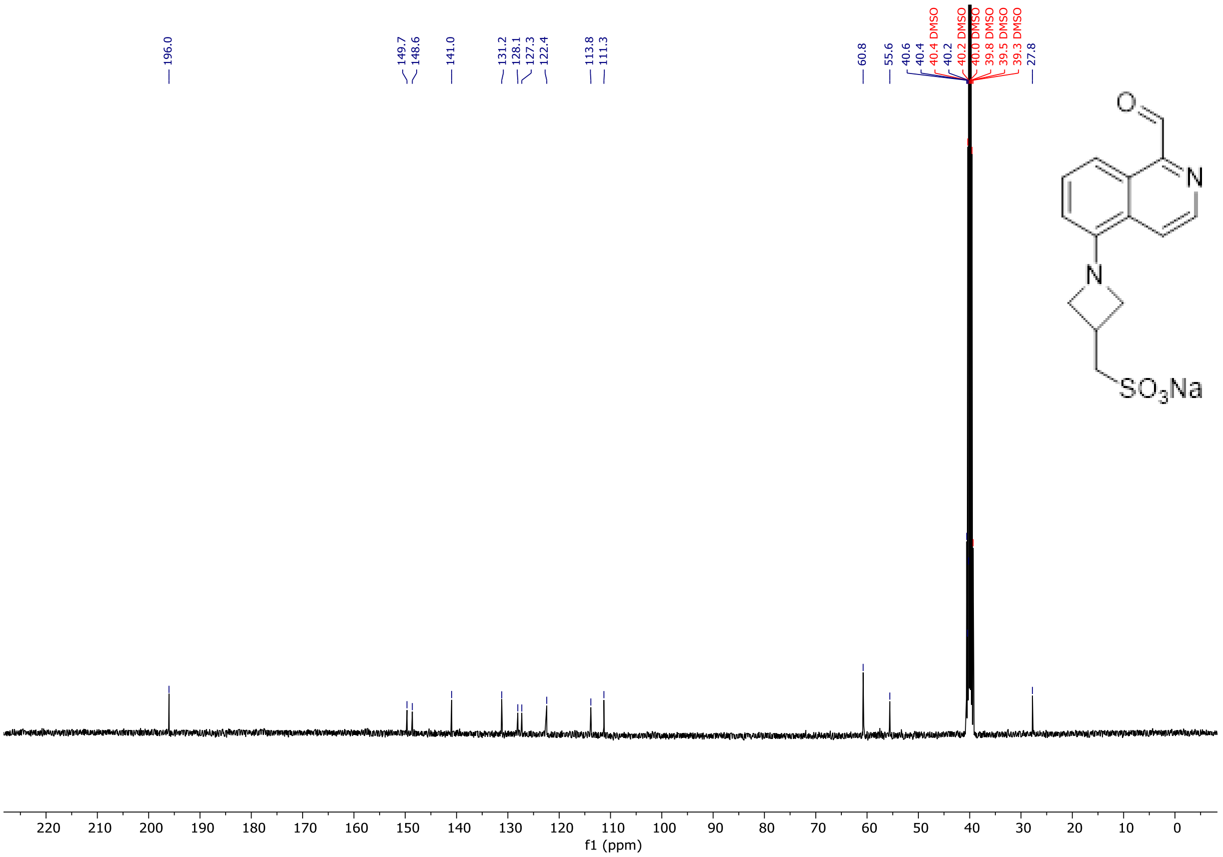

196.0
149.7
148.6
141.0
131.2
128.1
127.3
122.4
113.8
111.3
60.8
55.6
40.6
40.4
40.4 DMSO
40.2
40.2 DMSO
40.0 DMSO
39.8 DMSO
39.5 DMSO
39.3 DMSO
27.8
220 210 200 190 180 170 160 150 140 130 120 110 100 90 80 70 60 50 40 30 20 10 0
f1 (ppm)
O
N
N
SO3Na

Py-IP⁺

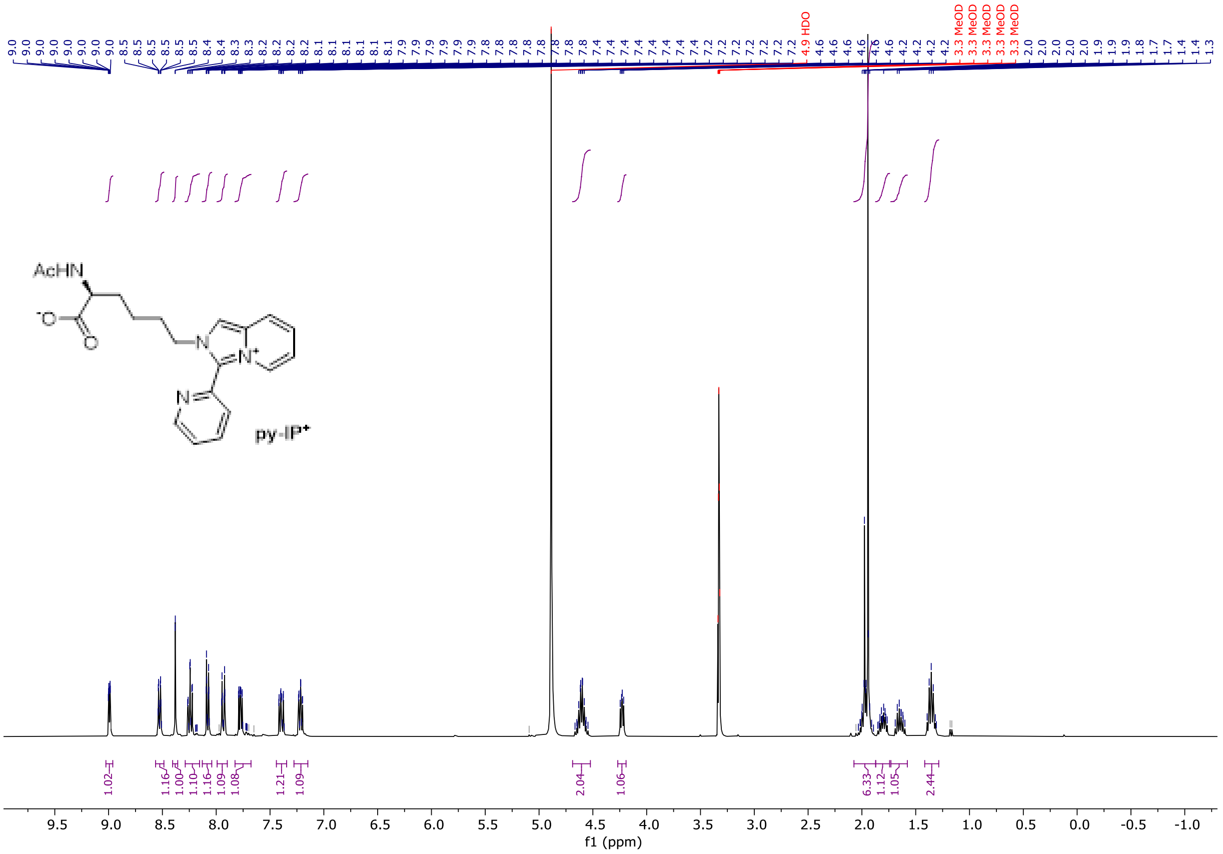

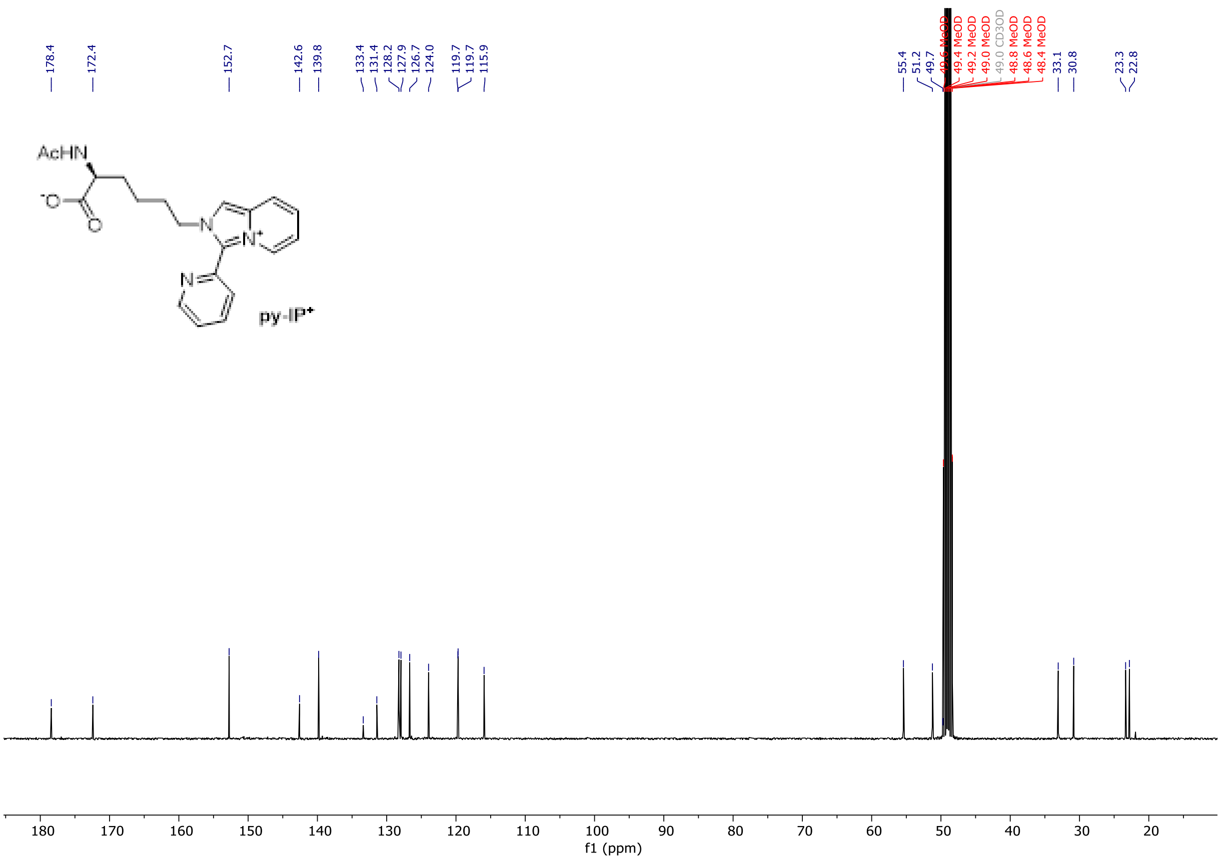

Qu-IP⁺

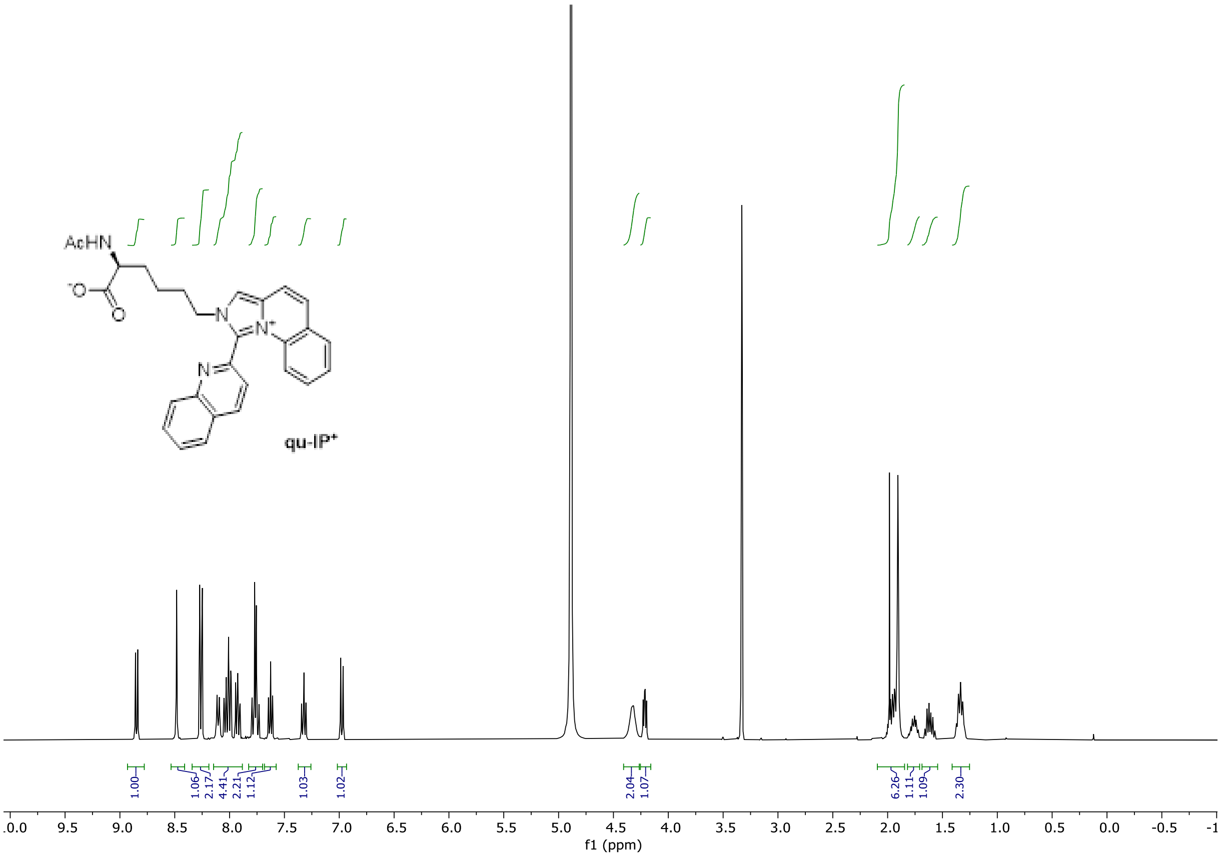

AcHN
qu-IP⁺
1.00
1.06
2.17
4.41
2.21
1.12
1.03
1.02
2.04
1.07
6.26
1.11
1.09
2.30
f1 (ppm)

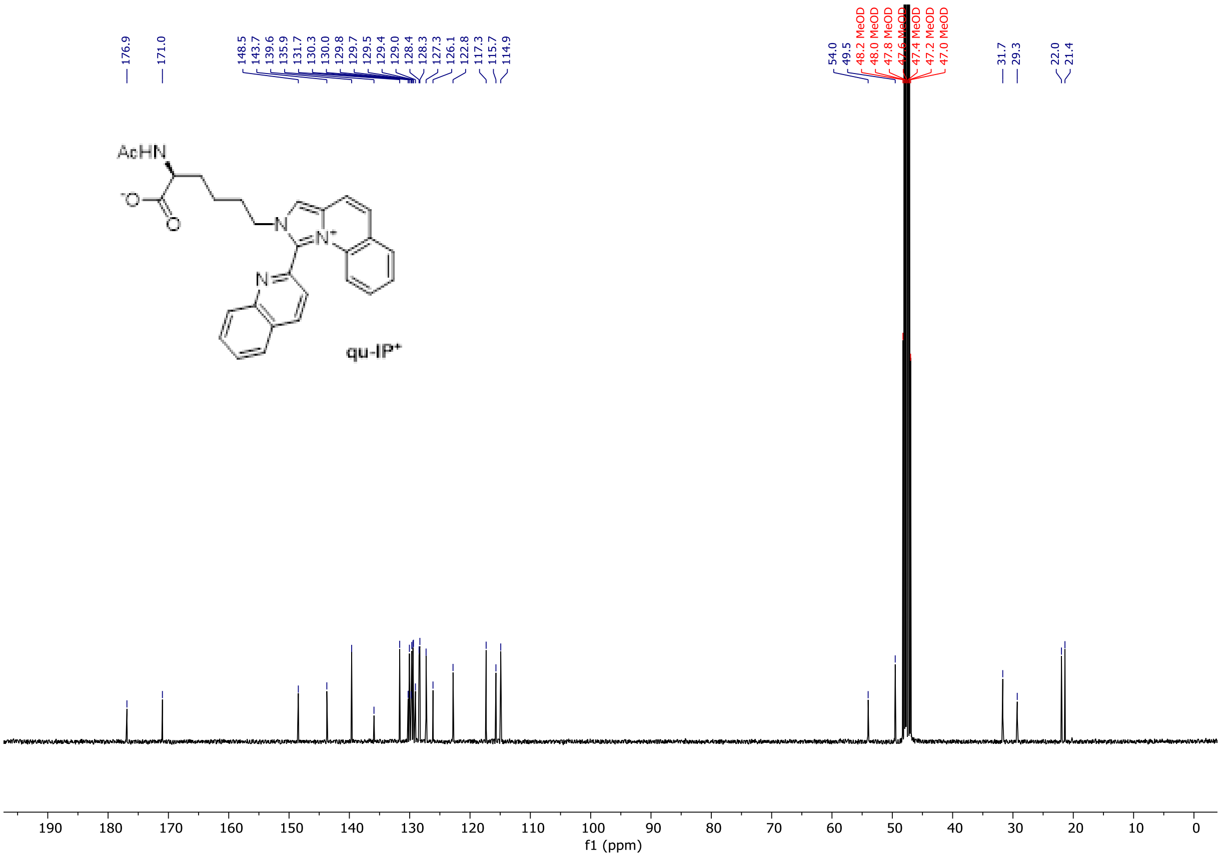

176.9
171.0
148.5
143.7
139.6
135.9
131.7
130.3
130.0
129.8
129.7
129.5
129.4
129.0
128.4
128.3
127.3
126.1
122.8
117.3
115.7
114.9
54.0
49.5
48.2 MeOD
48.0 MeOD
47.8 MeOD
47.6 MeOD
47.4 MeOD
47.2 MeOD
47.0 MeOD
31.7
29.3
22.0
21.4
AcHN
qu-IP⁺
f1 (ppm)

Iqu-lys

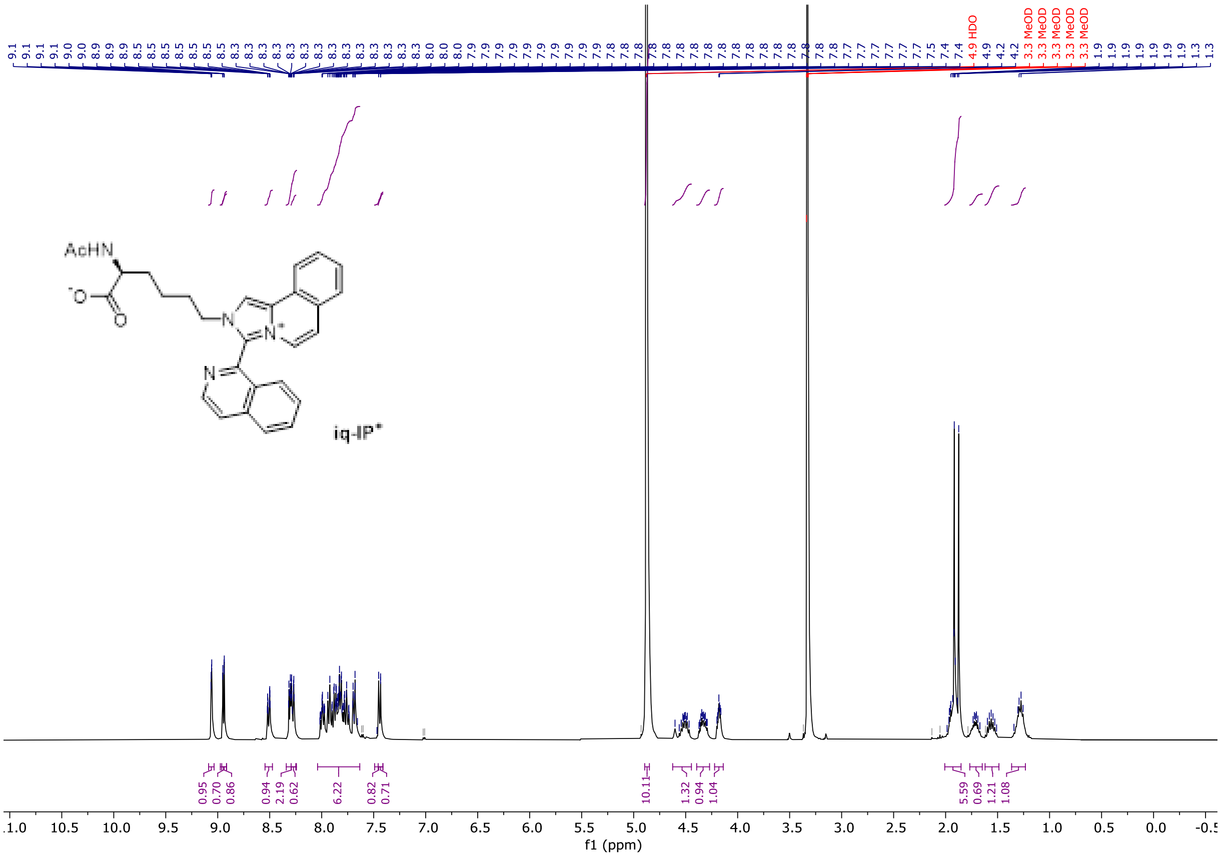

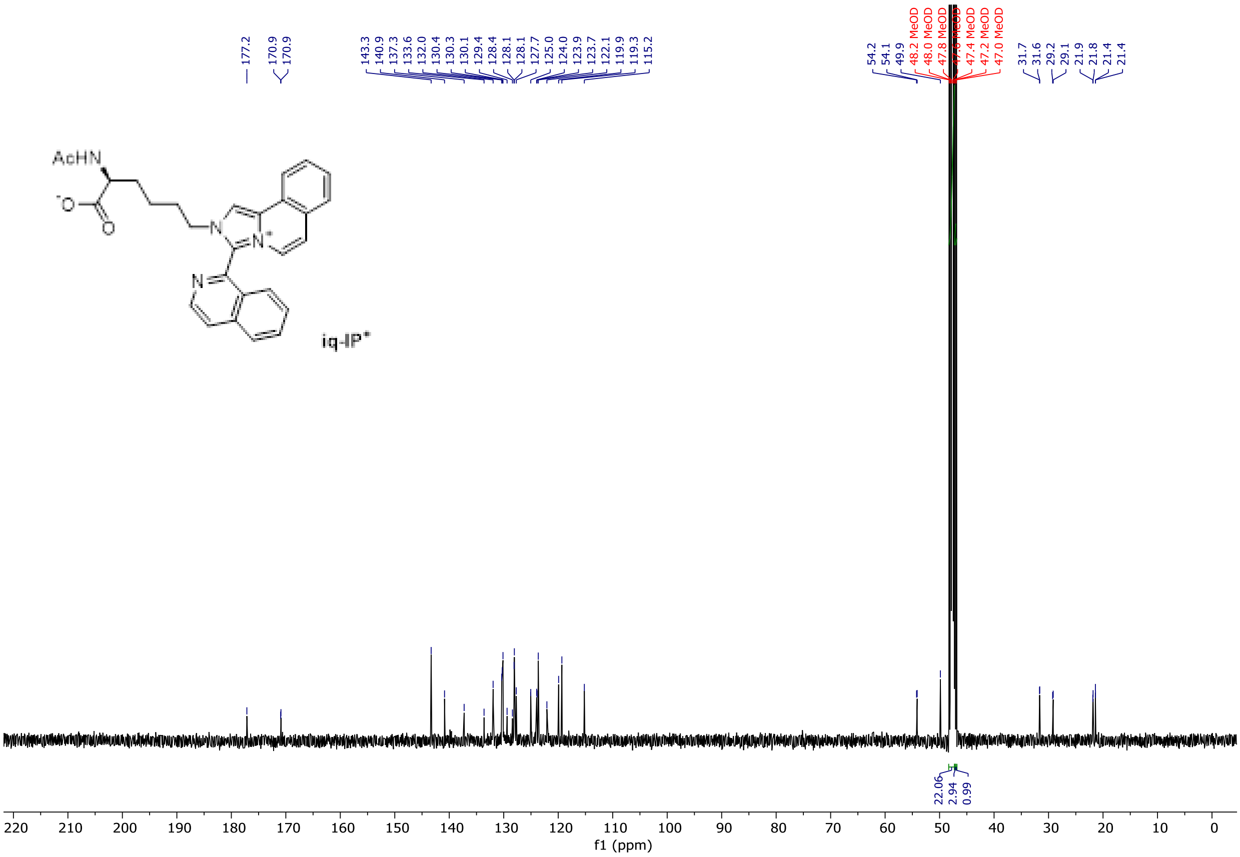

8a

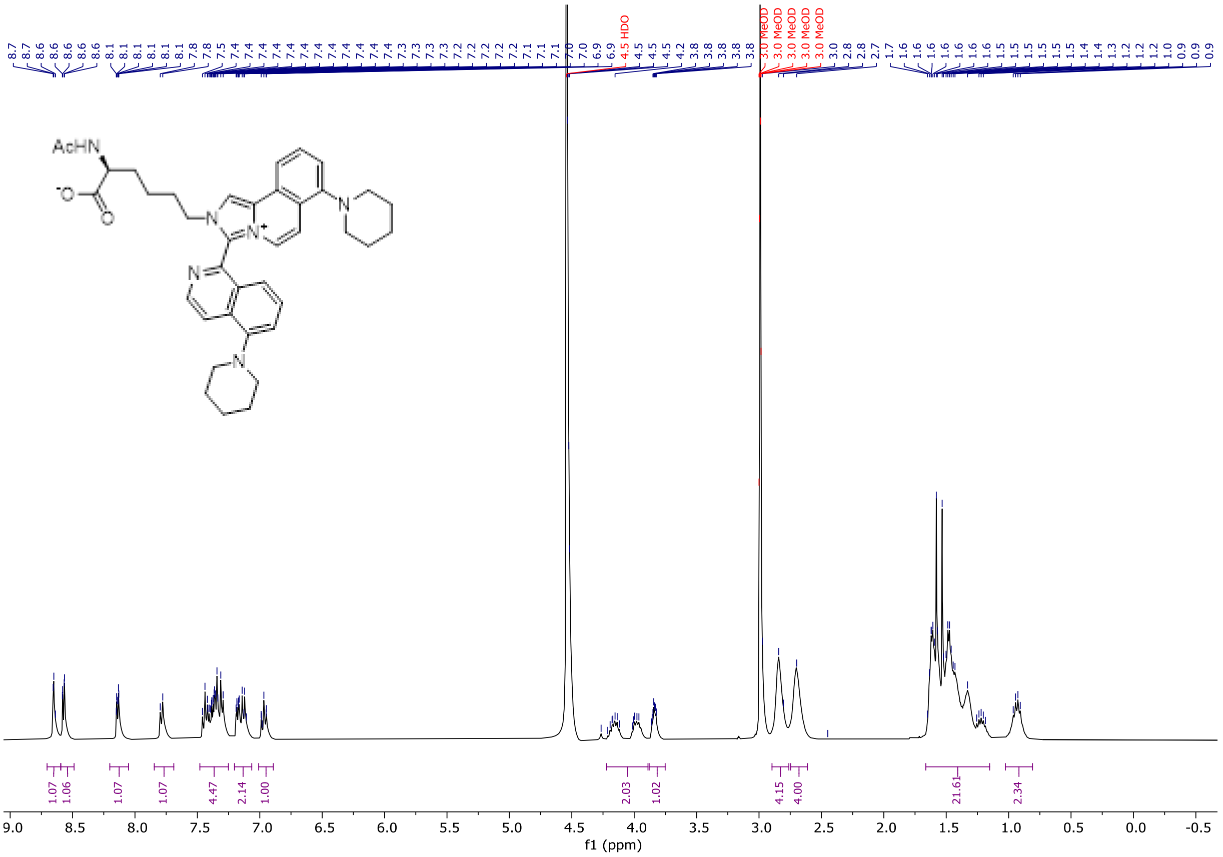

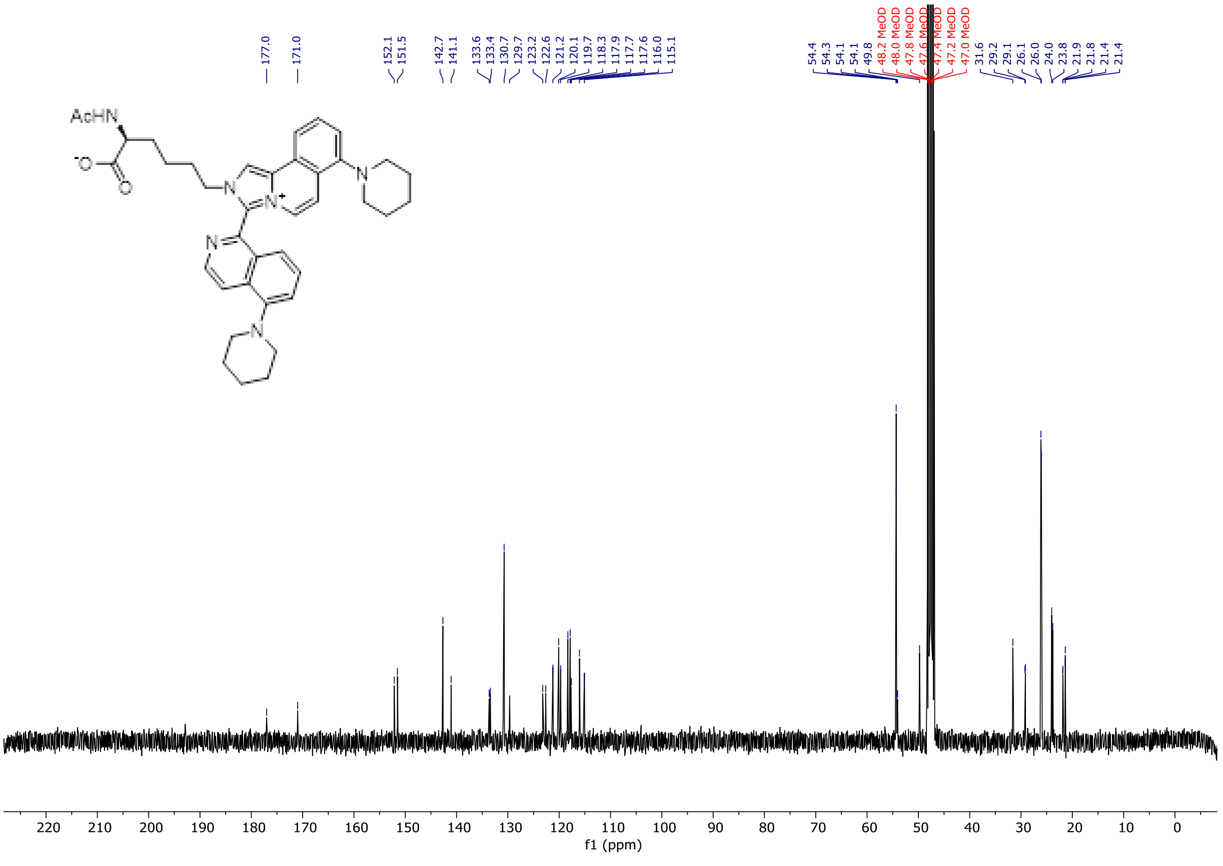

8b

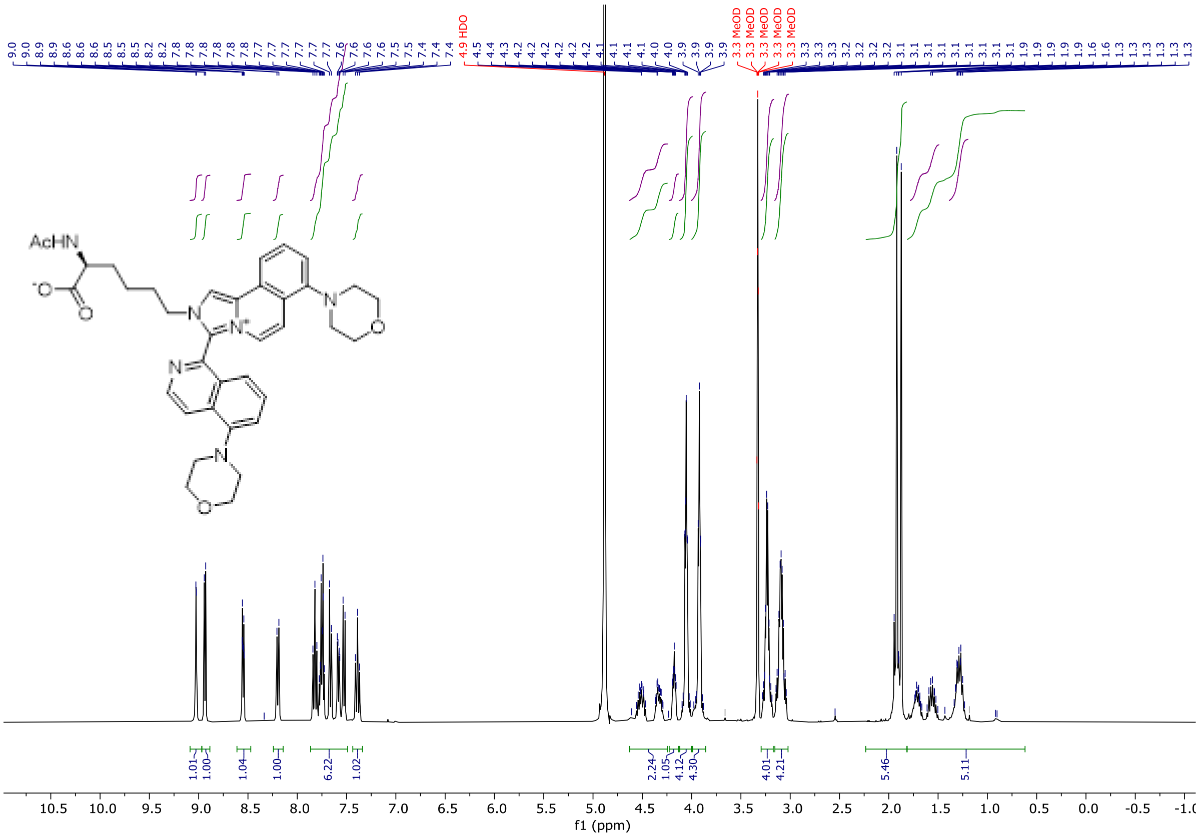

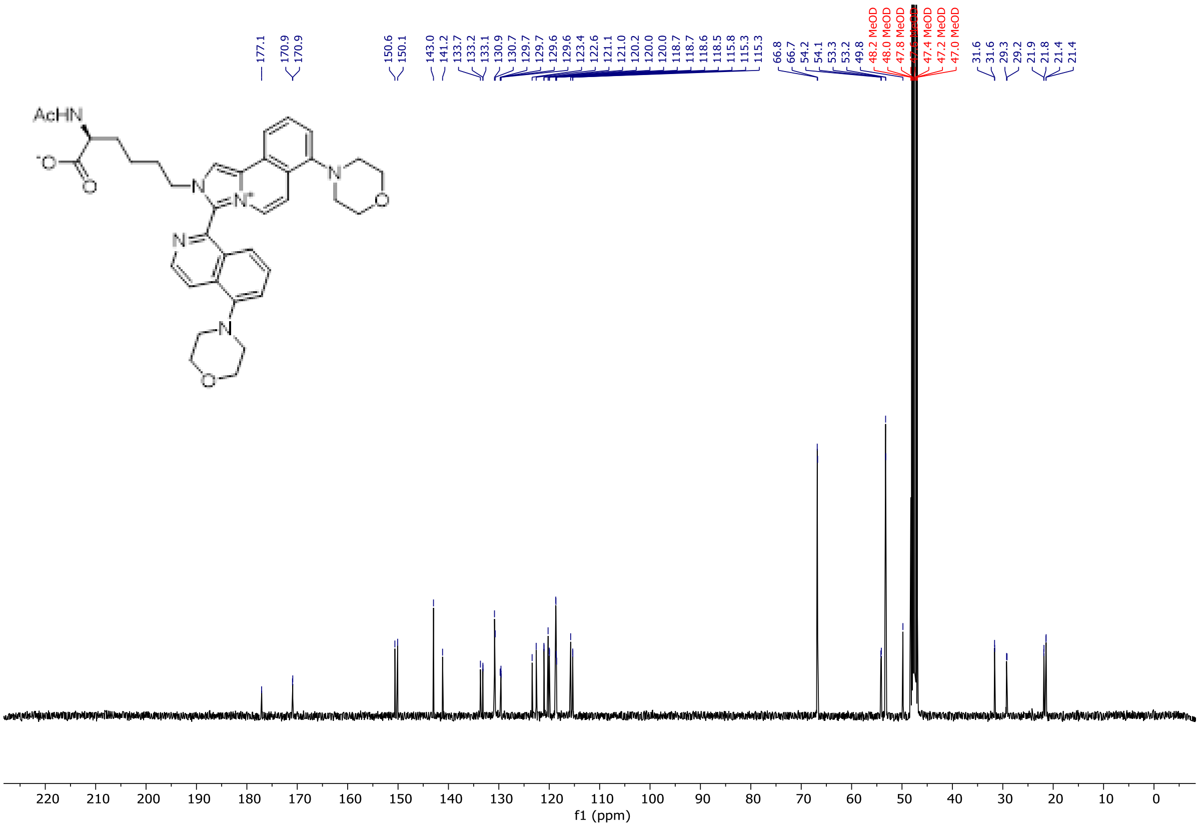

8c

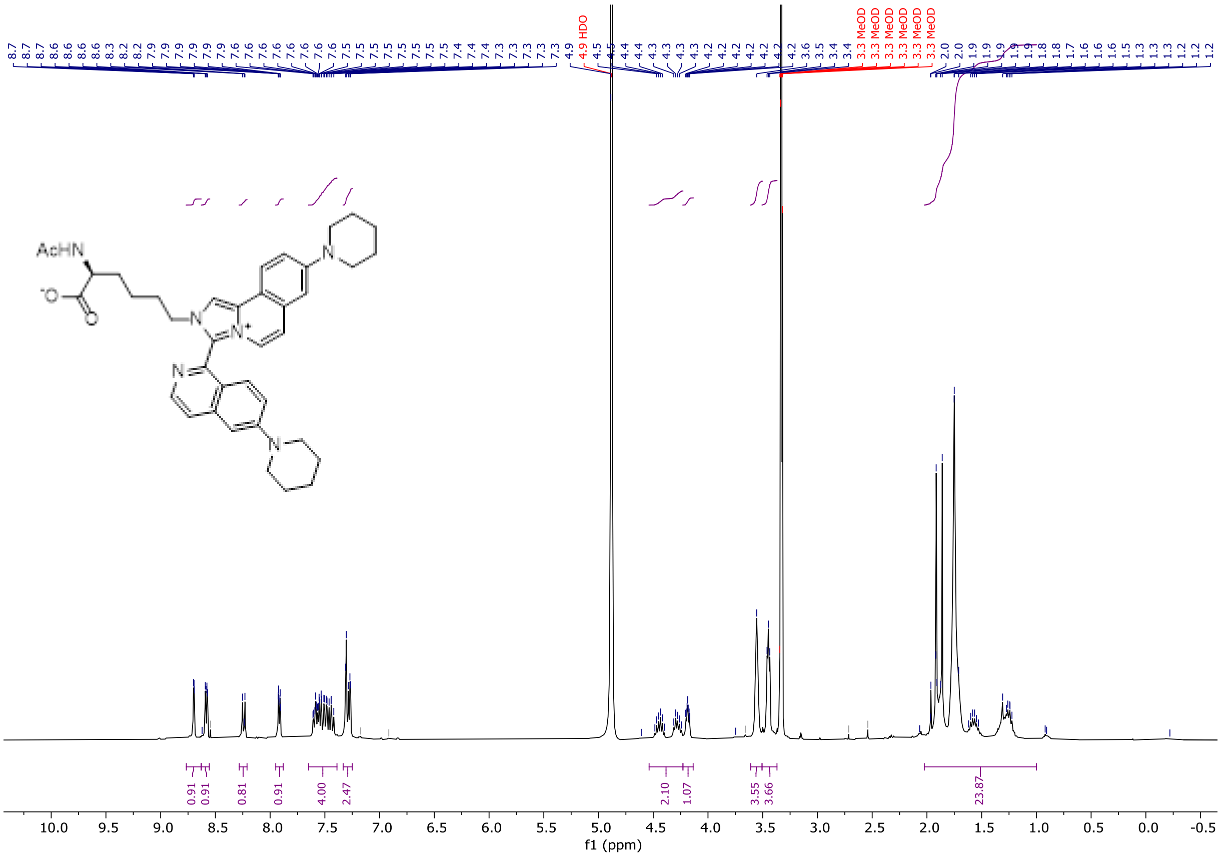

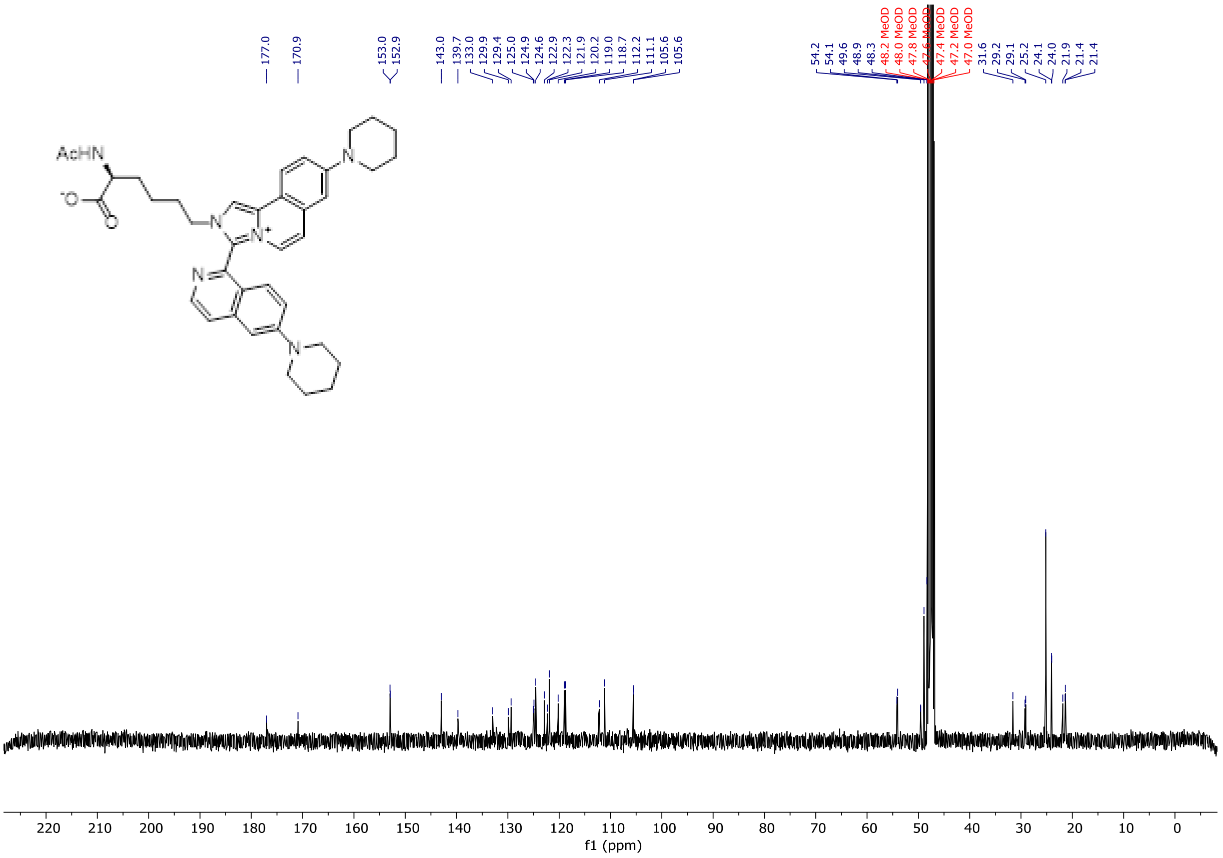

8d

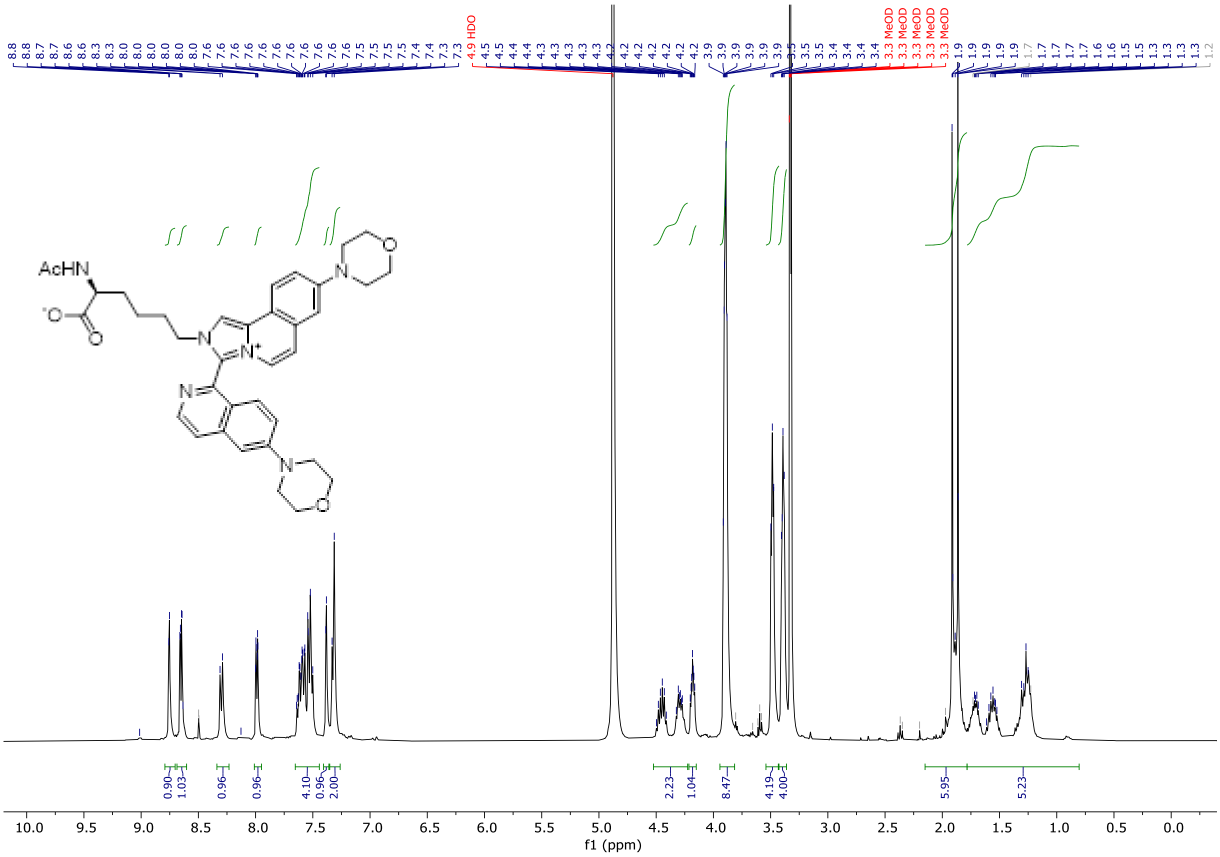

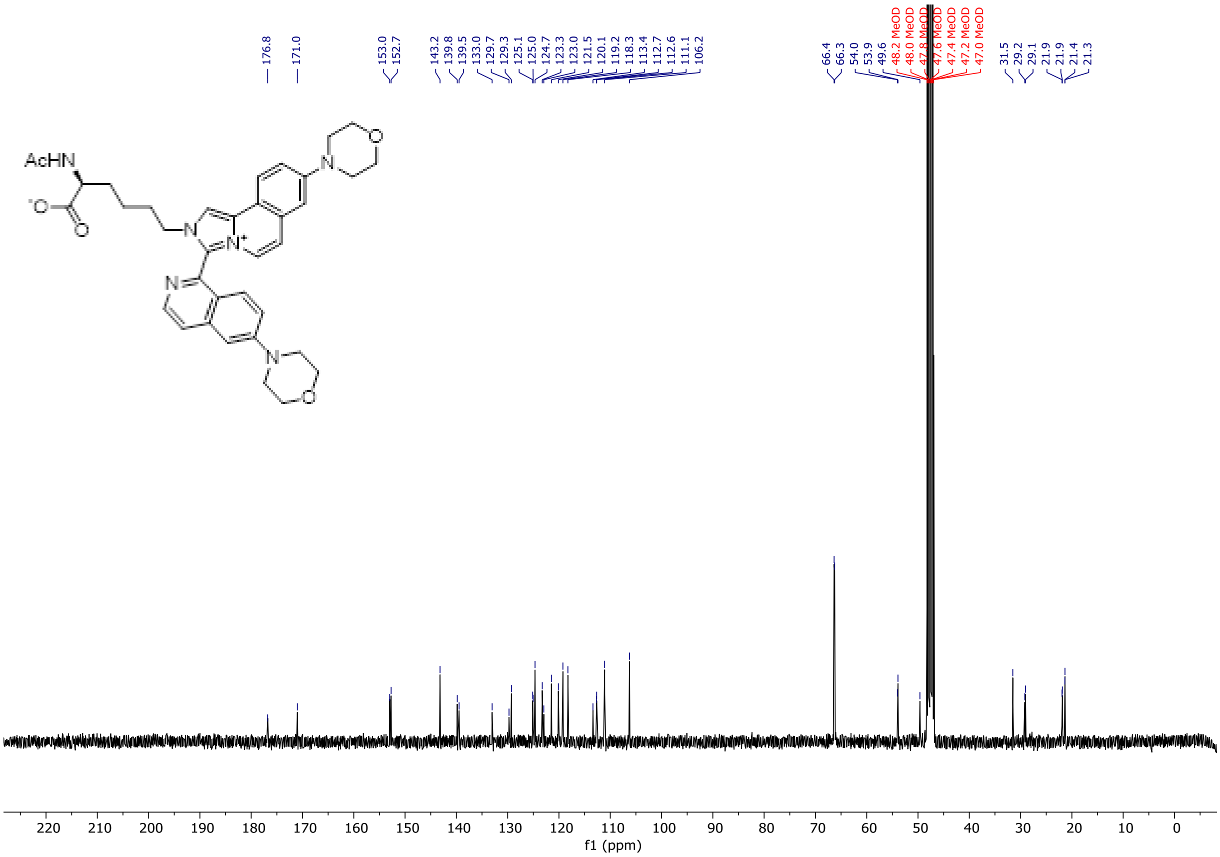

8e

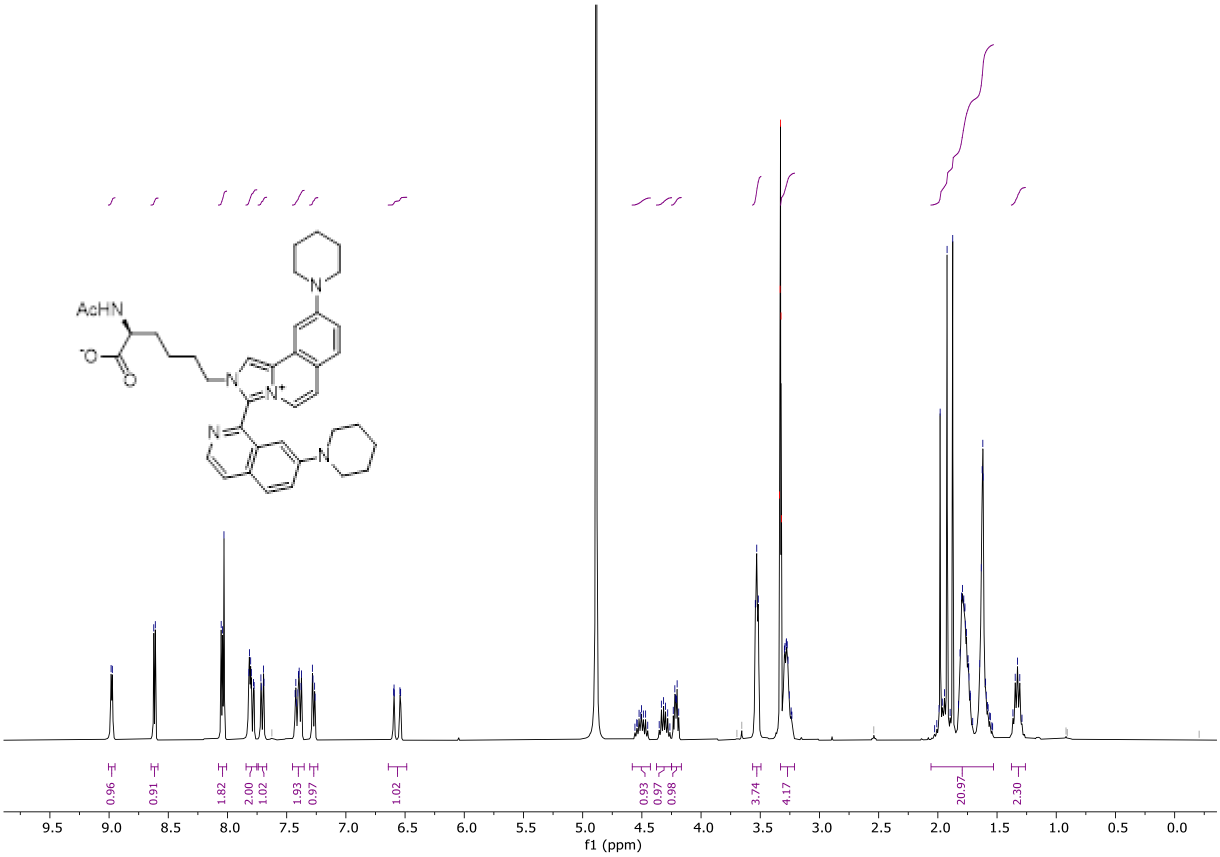

AcHN
N
N+
N
N
N
0.96
0.91
1.82
2.00
1.02
1.93
0.97
1.02
0.93
0.97
0.98
3.74
4.17
20.97
2.30
9.5 9.0 8.5 8.0 7.5 7.0 6.5 6.0 5.5 5.0 4.5 4.0 3.5 3.0 2.5 2.0 1.5 1.0 0.5 0.0
f1 (ppm)

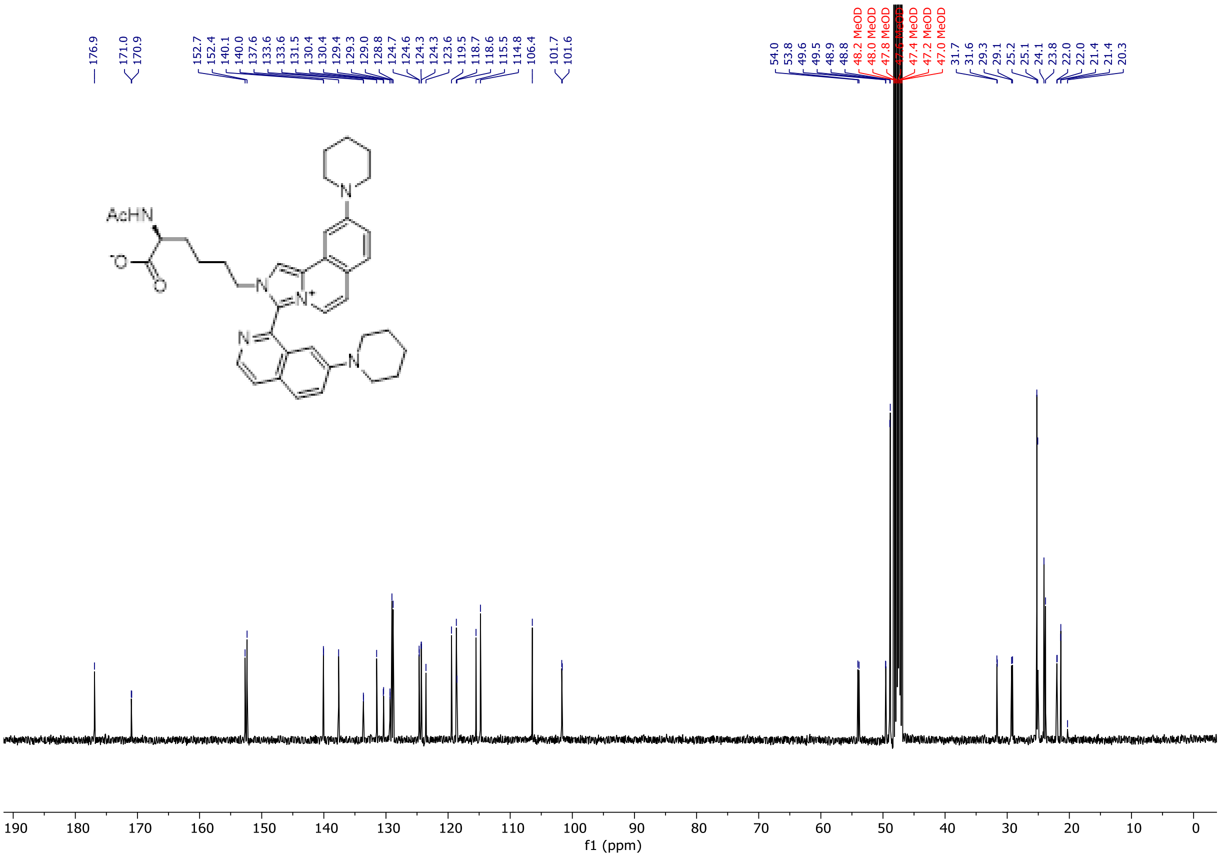

176.9
171.0
170.9
152.7
152.4
140.1
140.0
137.6
133.6
131.5
130.4
130.4
129.4
129.3
129.0
128.8
124.7
124.6
124.3
124.3
123.6
119.5
118.7
118.6
115.5
114.8
106.4
101.7
101.6
54.0
53.8
49.6
49.5
48.9
48.8
48.2 MeOD
48.0 MeOD
47.8 MeOD
47.4 MeOD
47.2 MeOD
47.0 MeOD
31.7
31.6
29.3
29.1
25.2
25.1
24.1
23.8
22.0
22.0
21.4
21.4
20.3
AcHN
N
N+
N
N
N
190 180 170 160 150 140 130 120 110 100 90 80 70 60 50 40 30 20 10 0
f1 (ppm)

8f

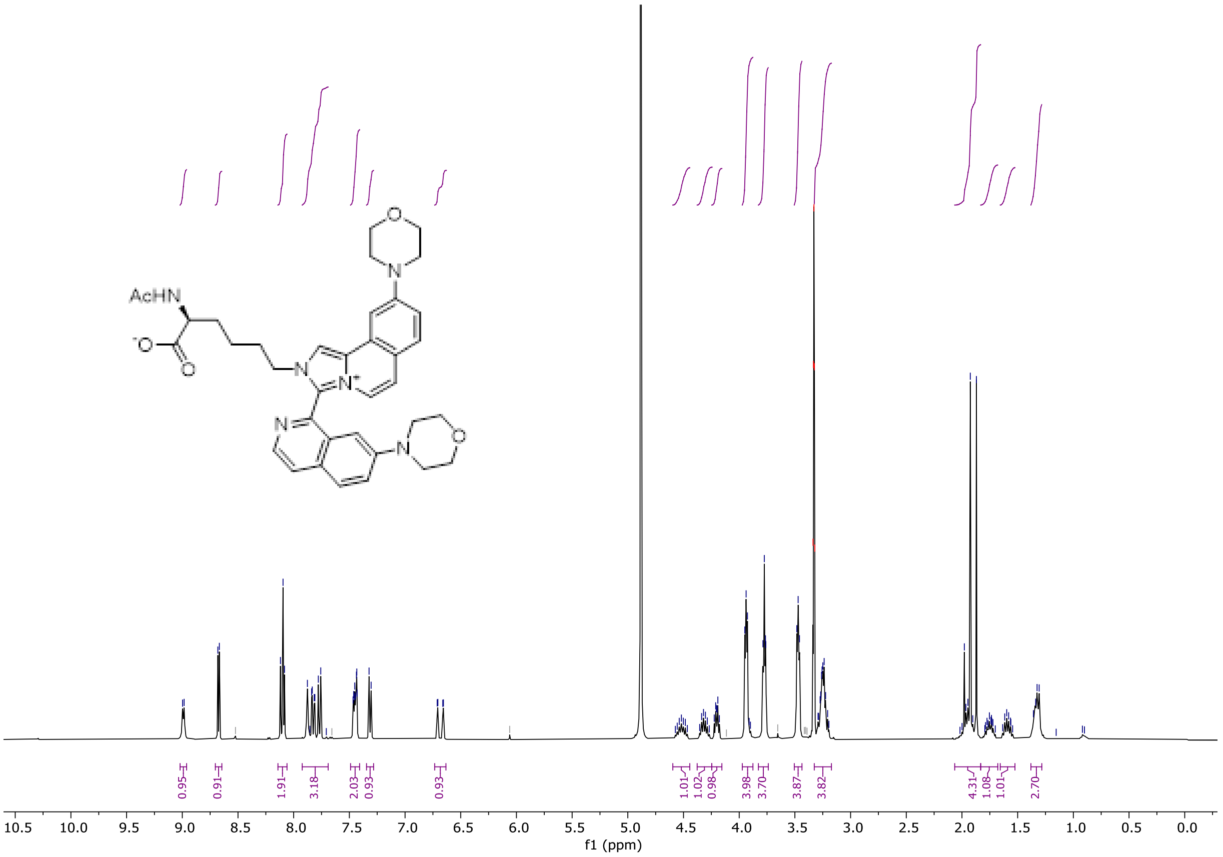

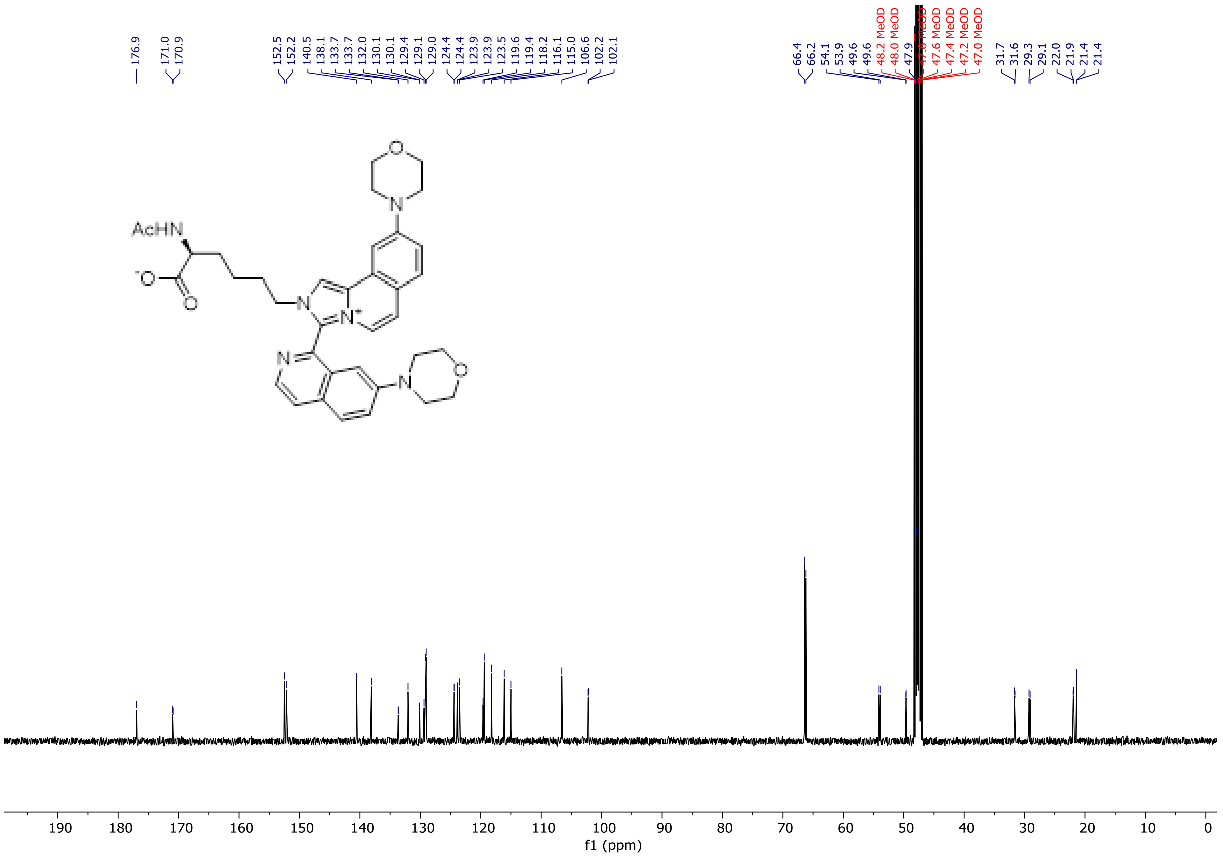

8g

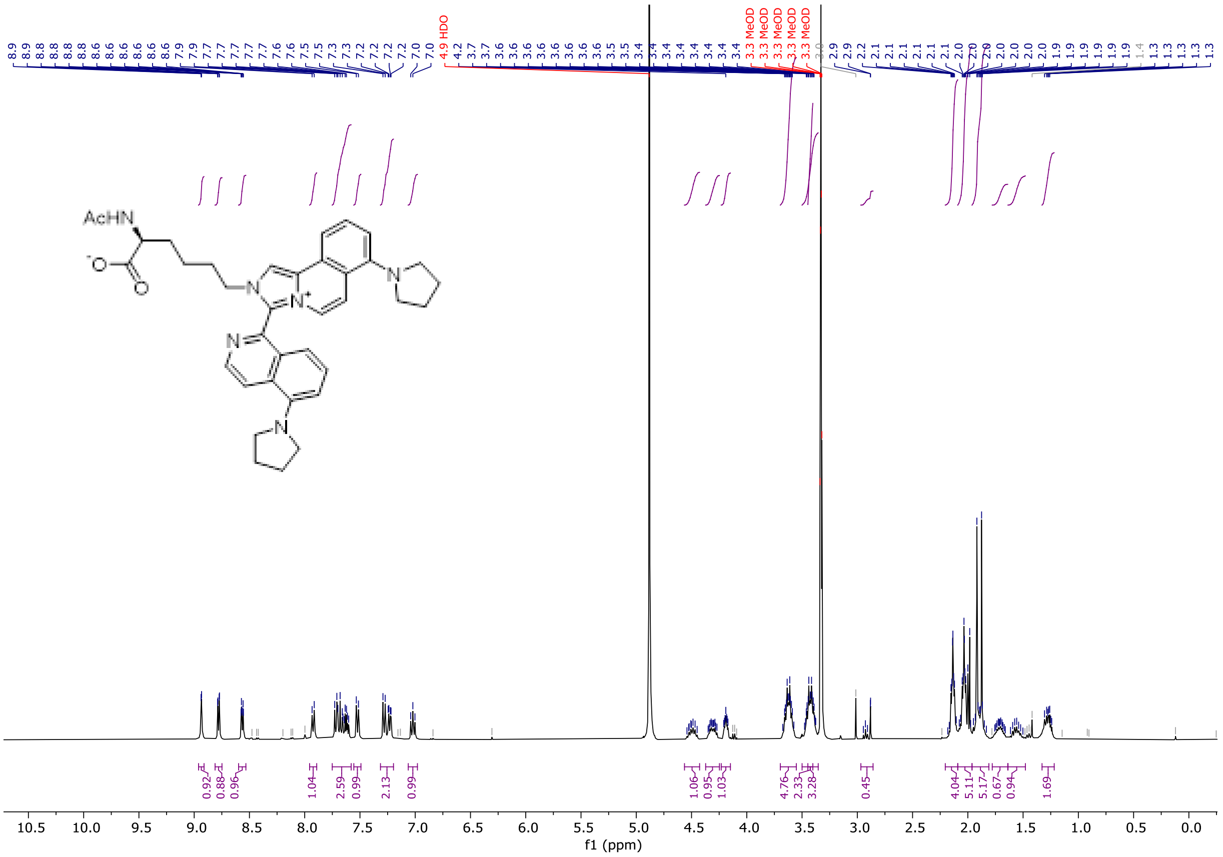

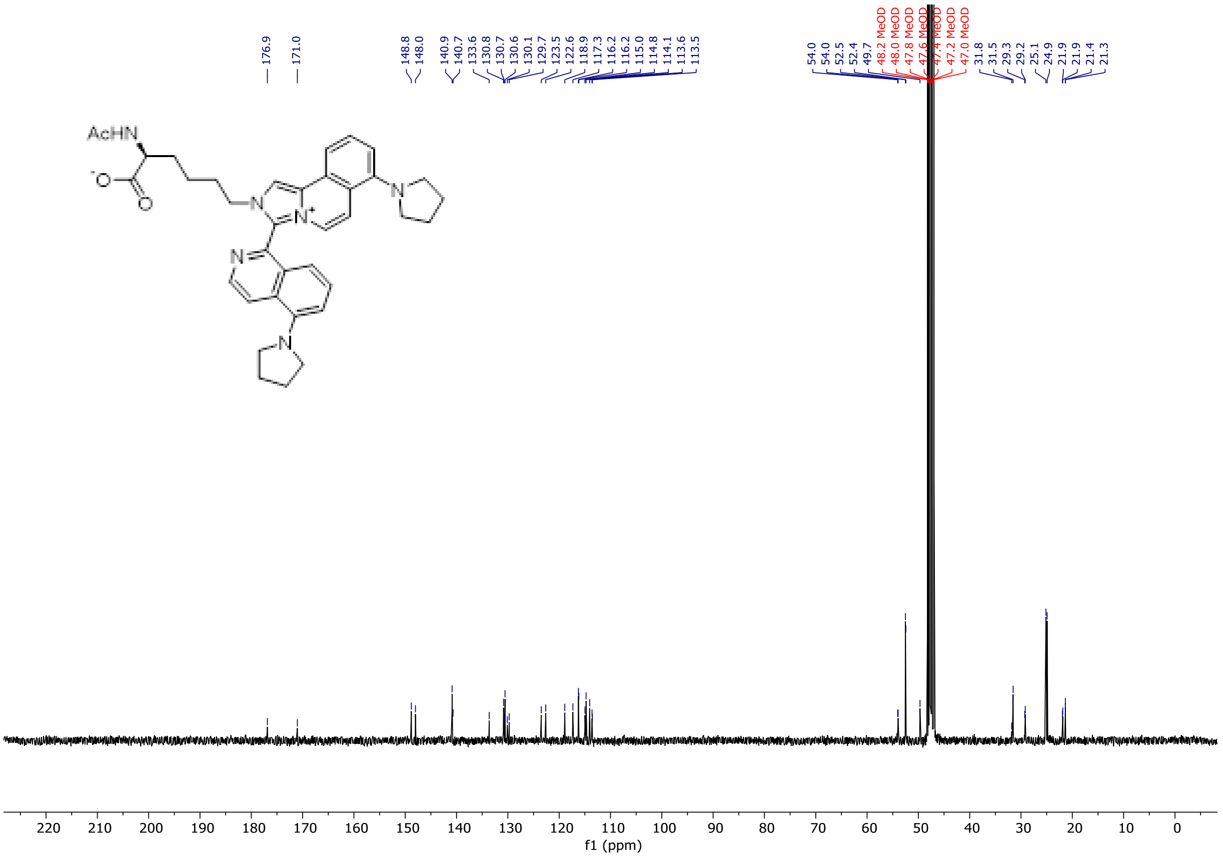

8h

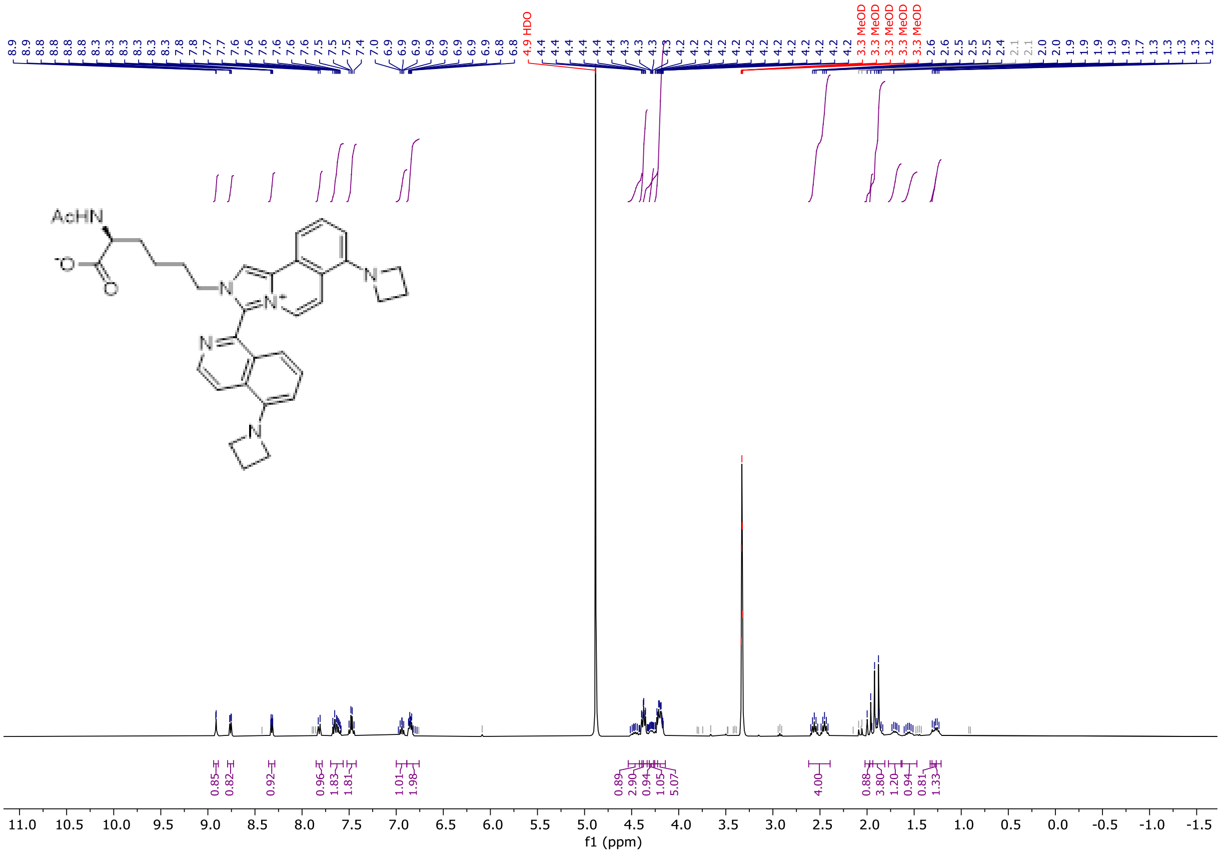

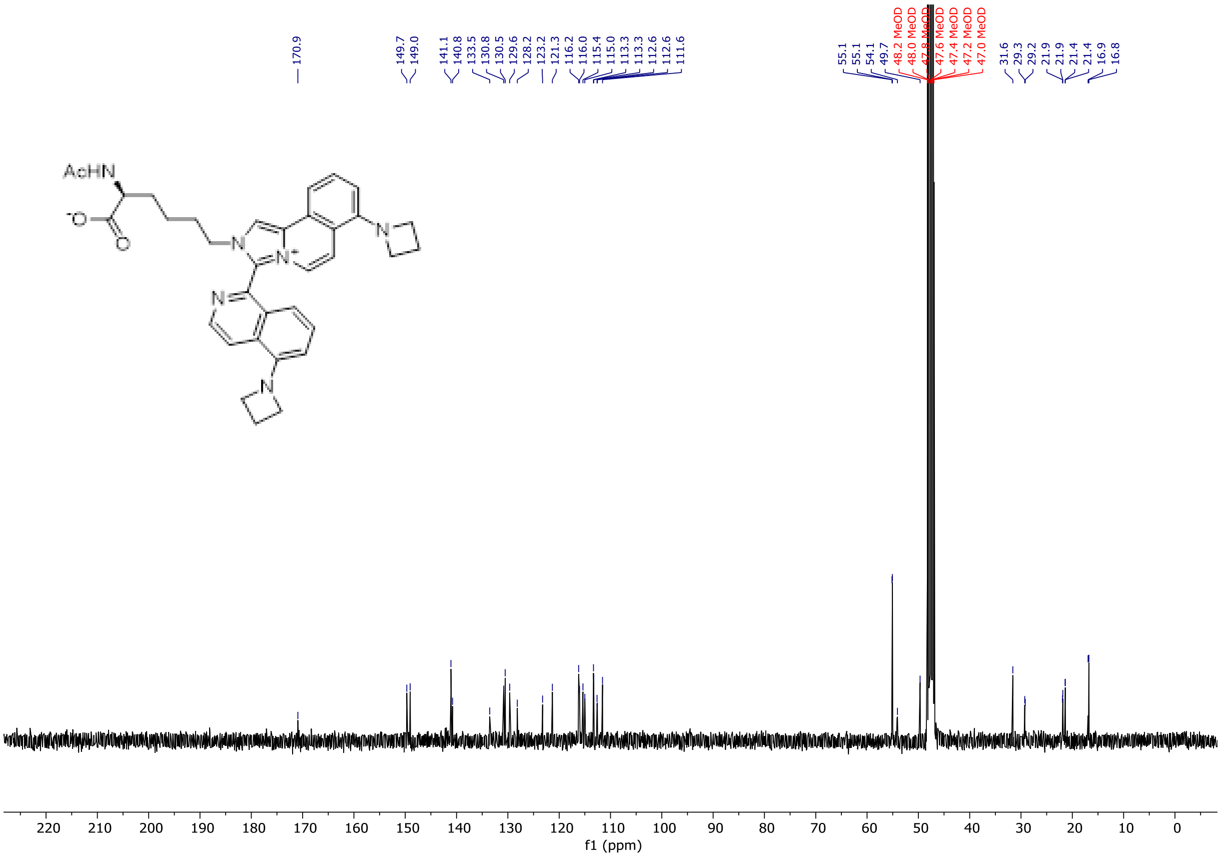

8i

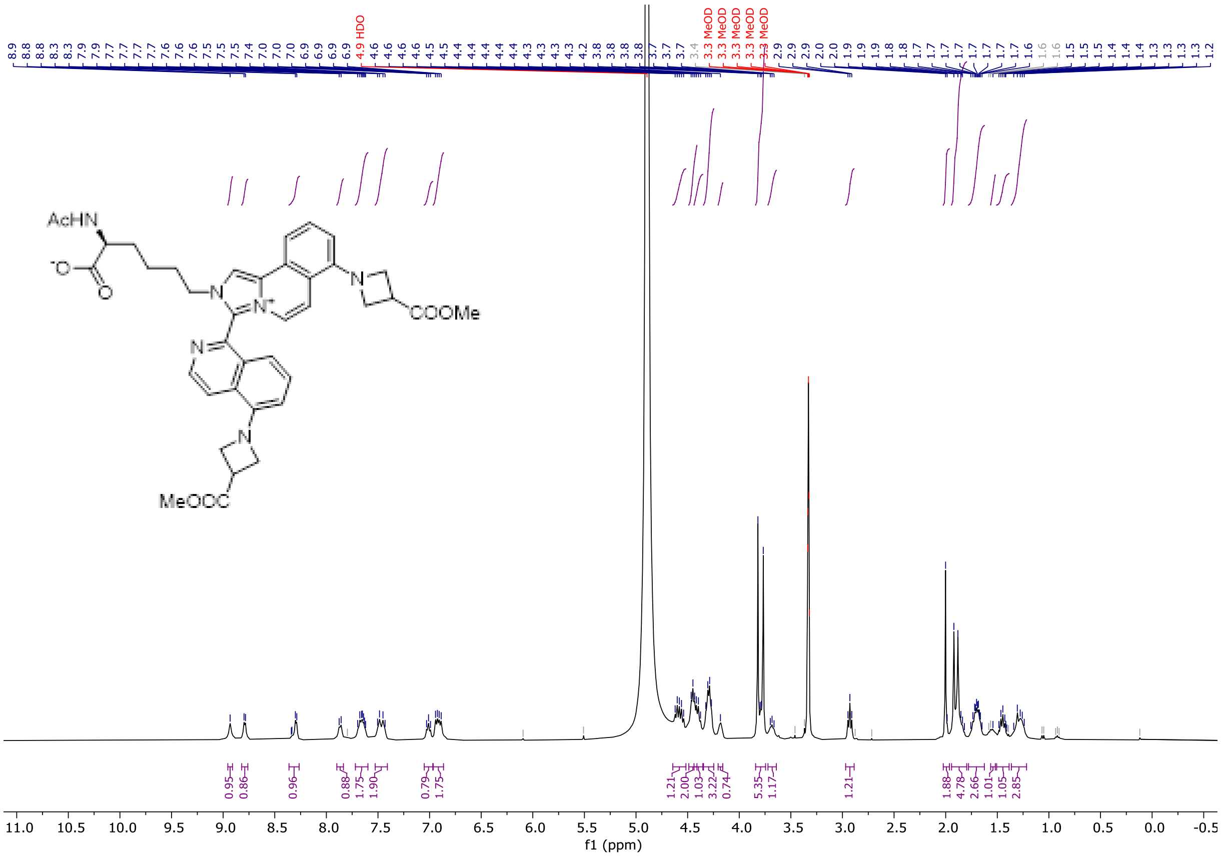
AcHN
COOMe
MeOOC
f1 (ppm)

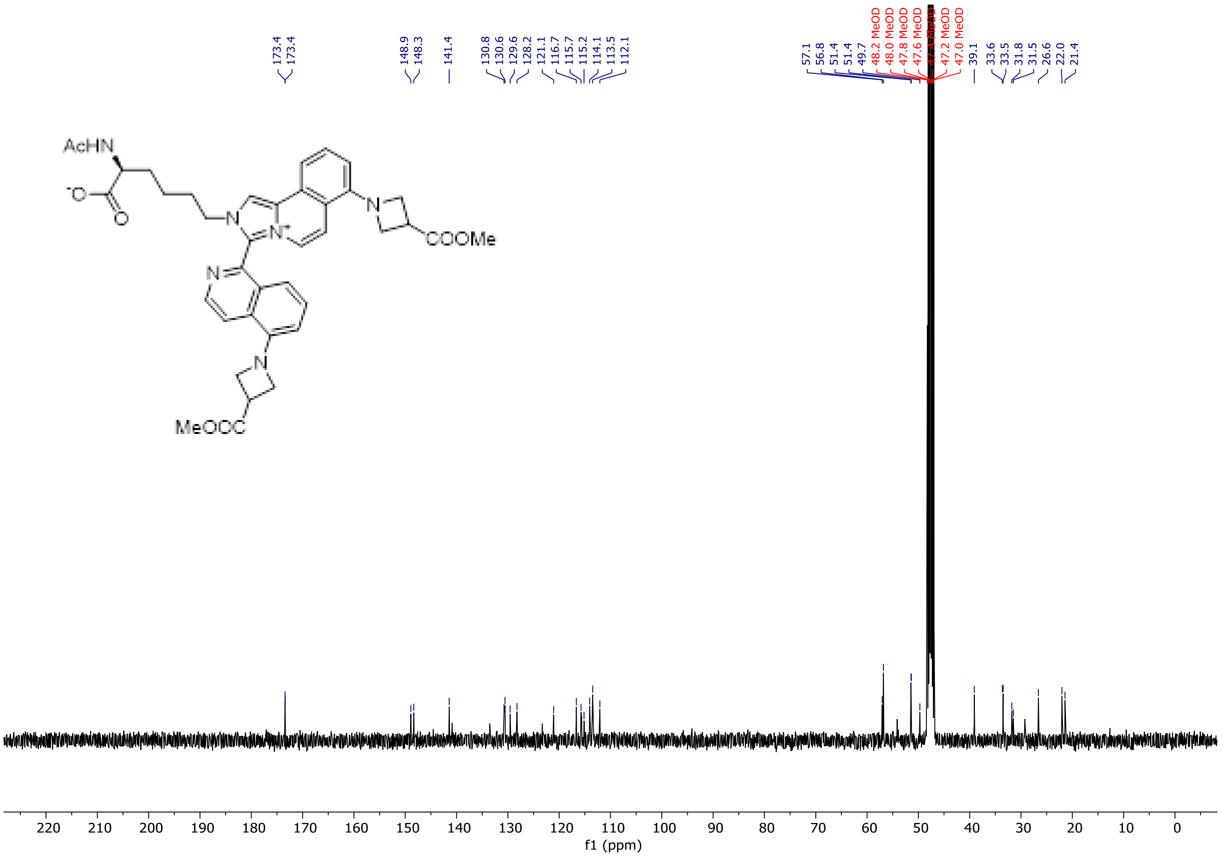
173.4
173.4
148.9
148.3
141.4
130.8
130.6
129.6
128.2
121.1
116.7
115.7
115.2
114.1
113.5
112.1
57.1
56.8
51.4
51.4
49.7
48.2 MeOD
48.0 MeOD
47.8 MeOD
47.6 MeOD
47.2 MeOD
47.0 MeOD
39.1
33.6
33.5
31.8
31.5
26.6
22.0
21.4
AcHN
COOMe
MeOOC
f1 (ppm)

8l

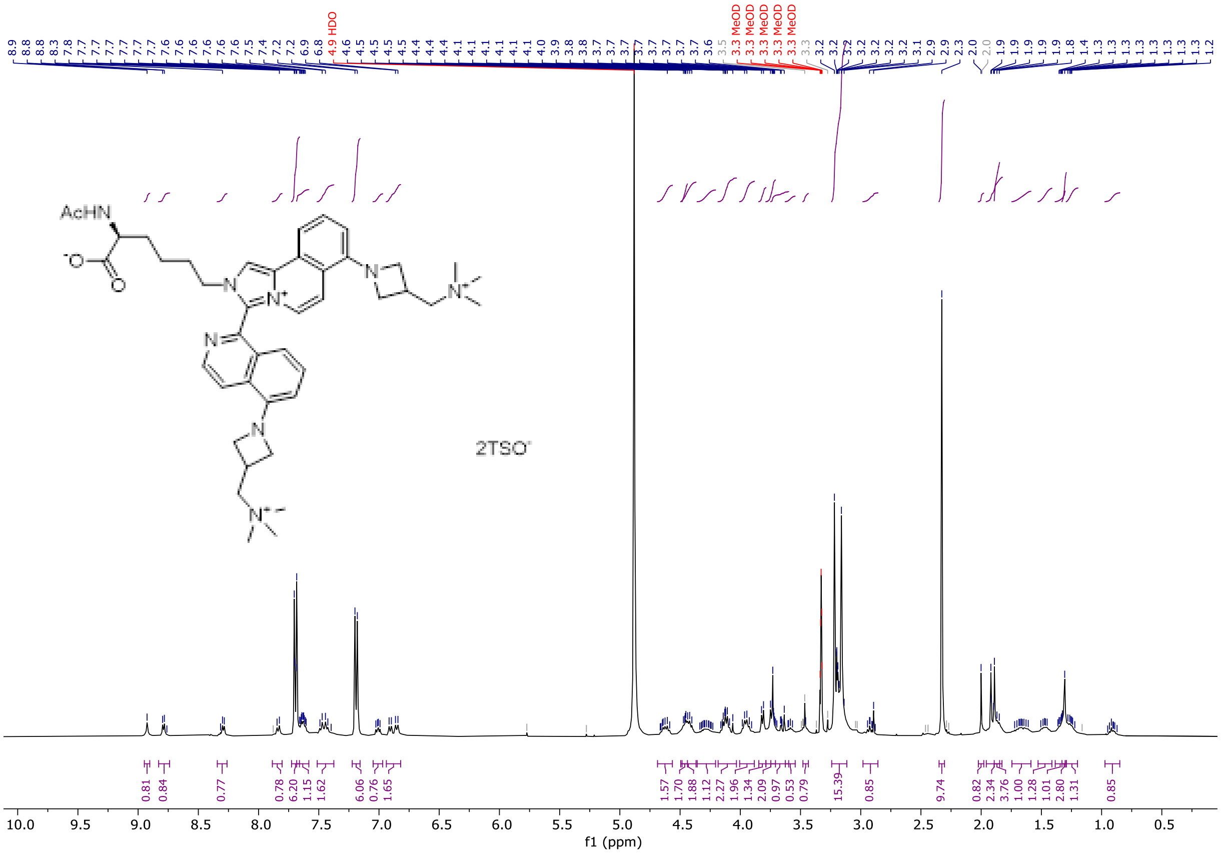

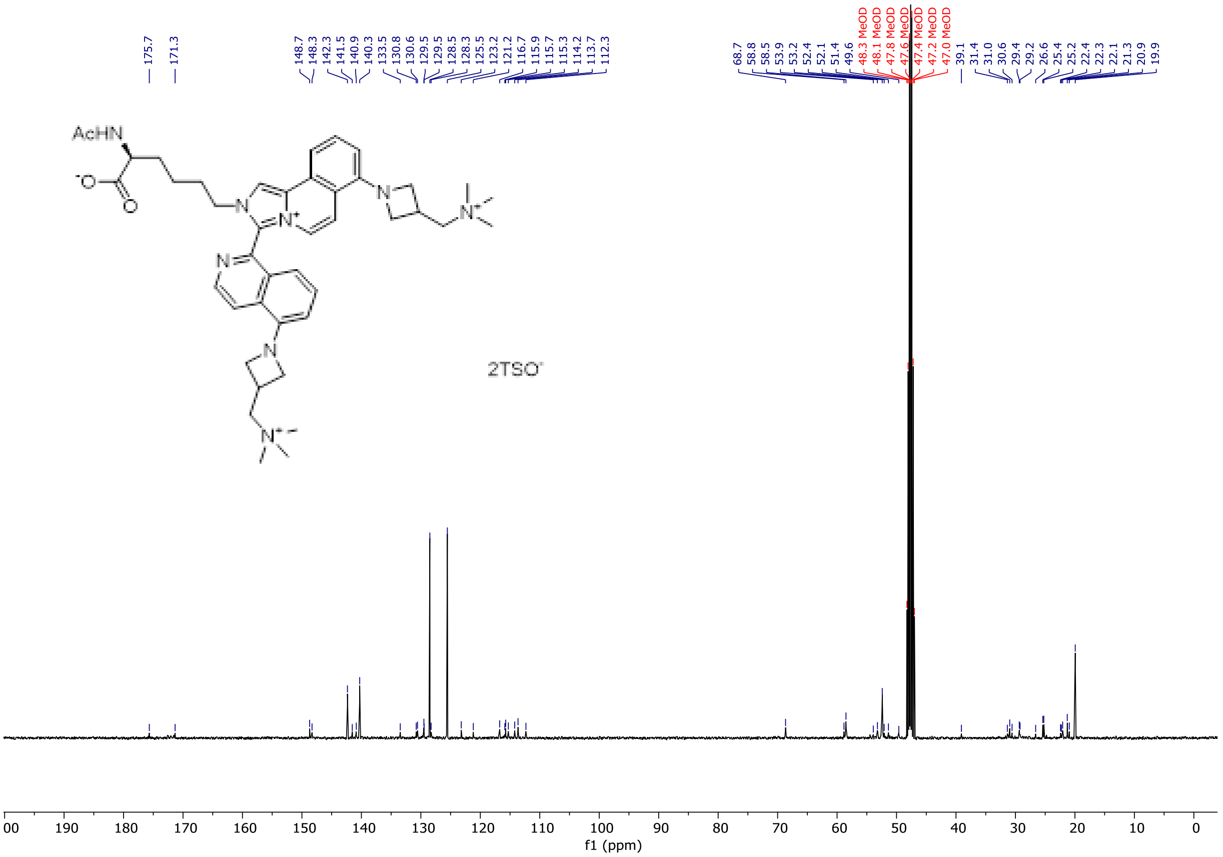

8m

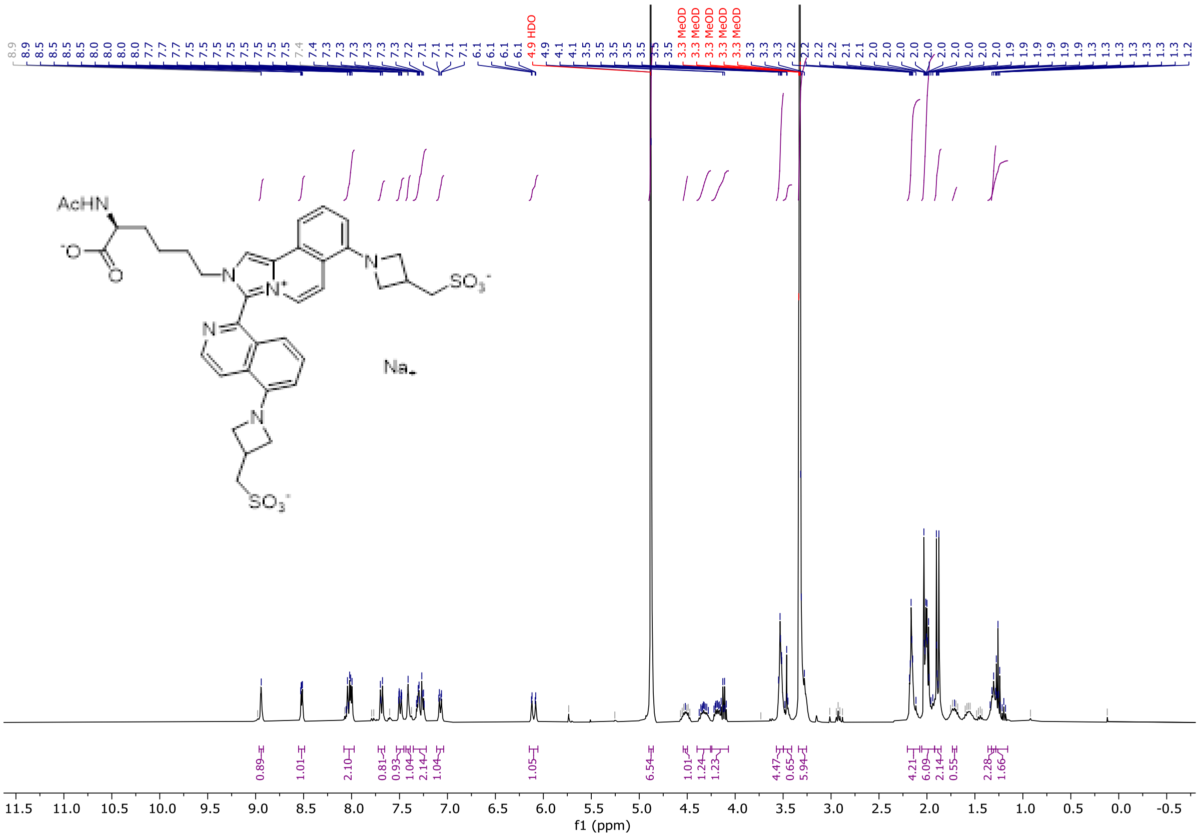

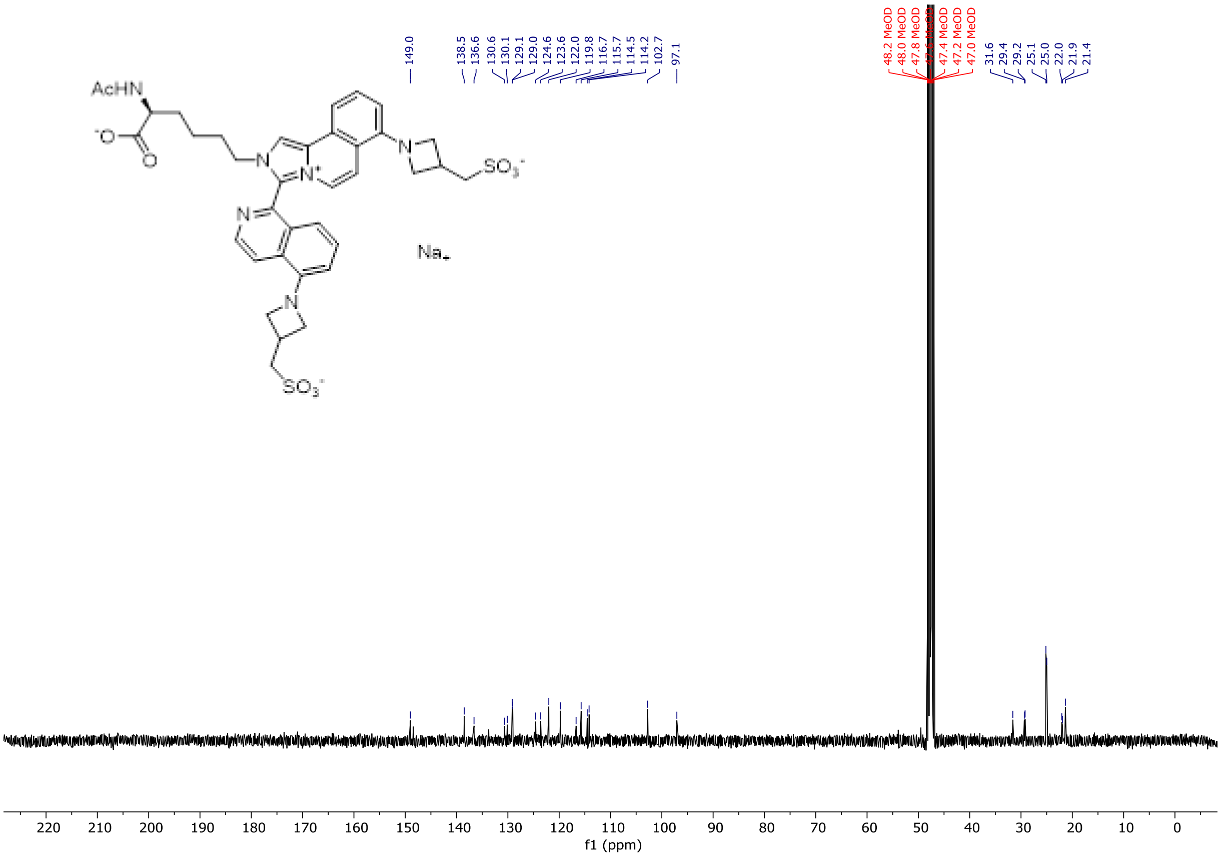

Halo-IP⁺

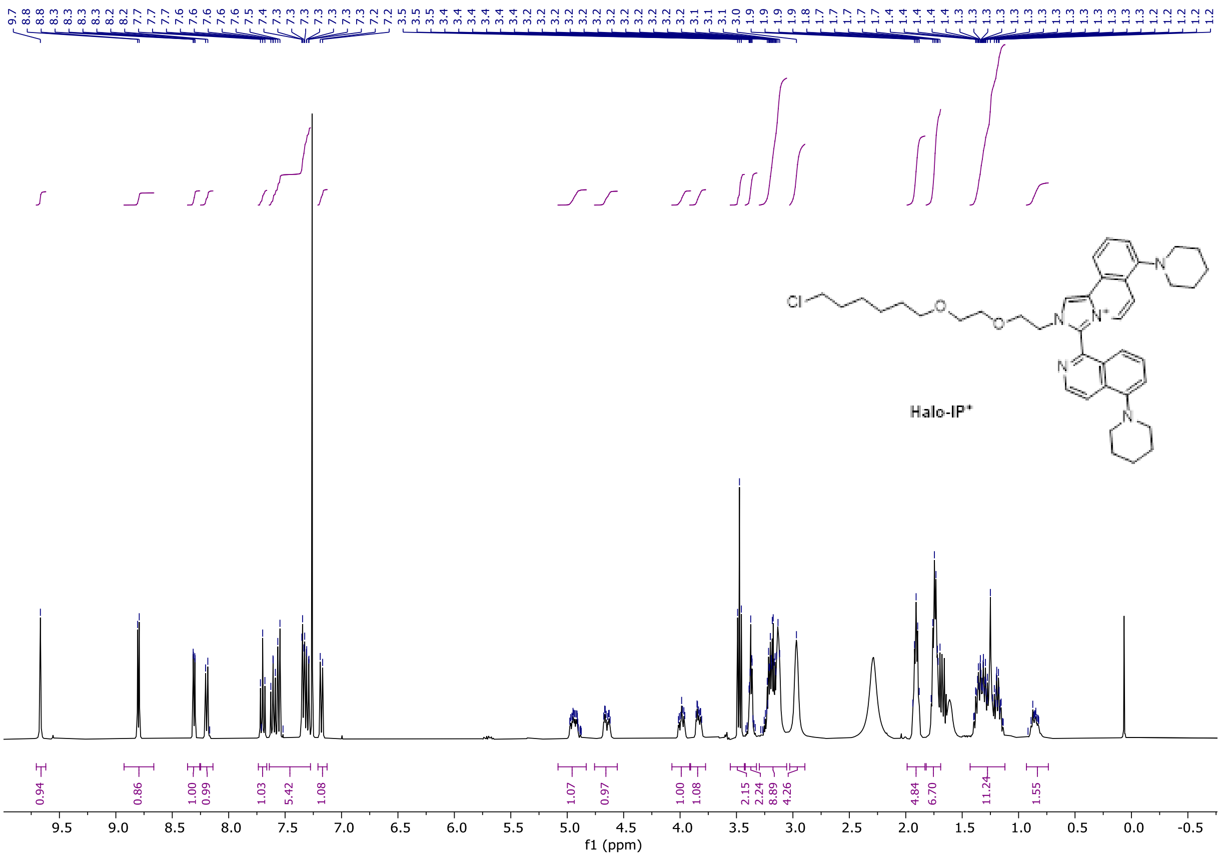

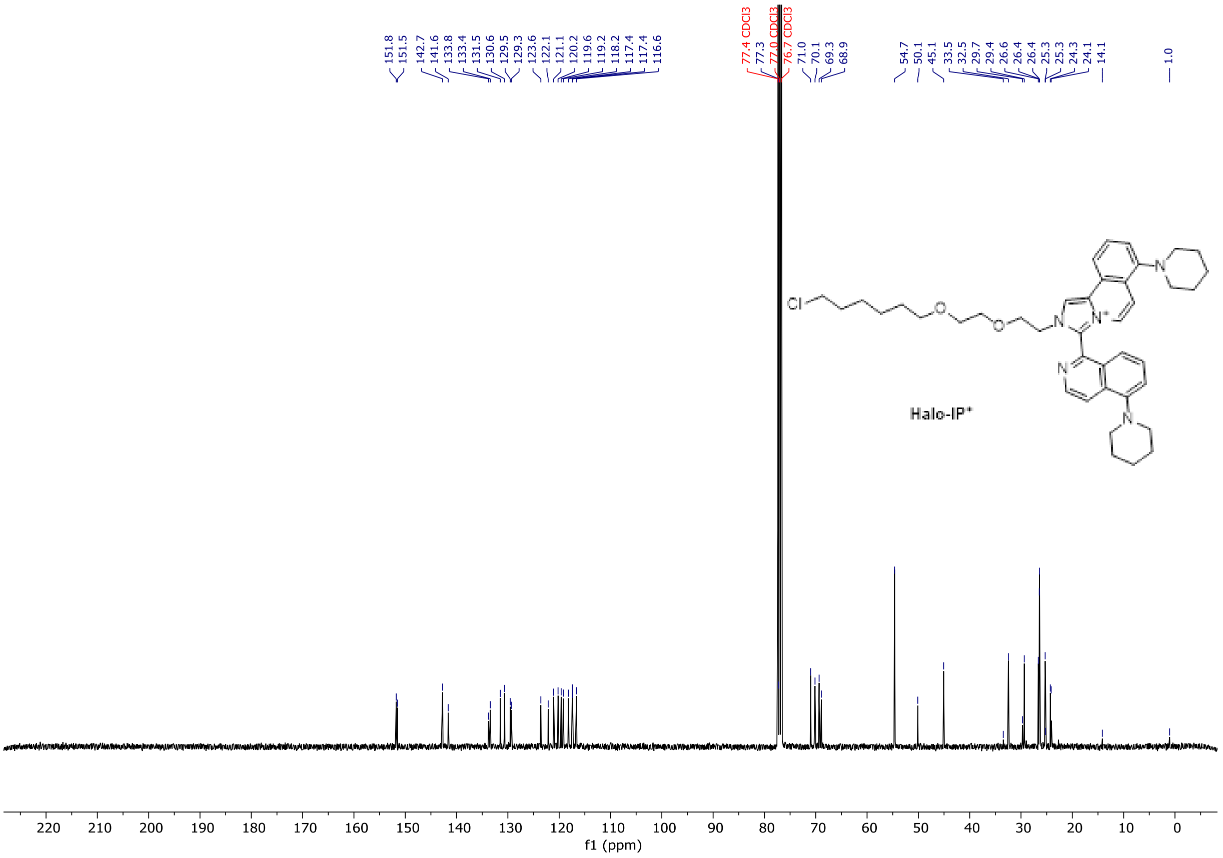

Halo-azIP⁺

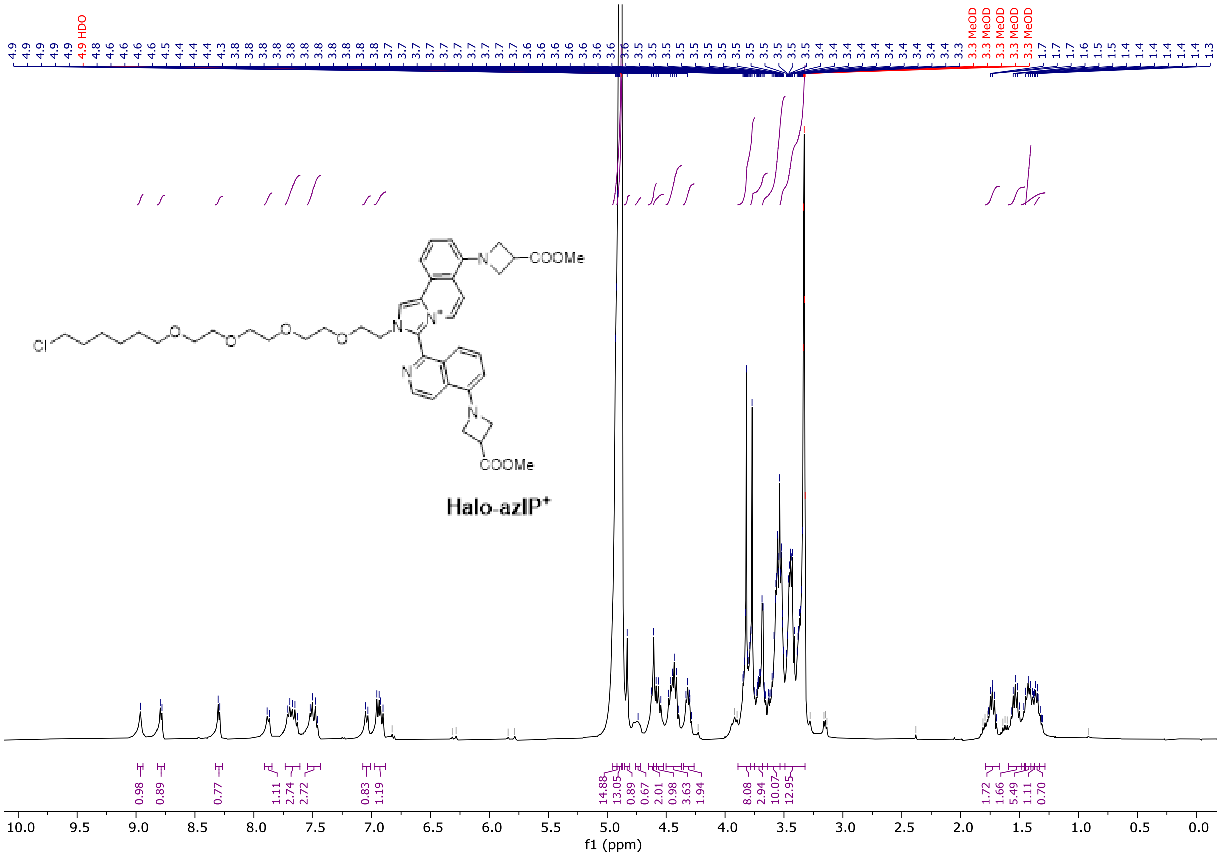

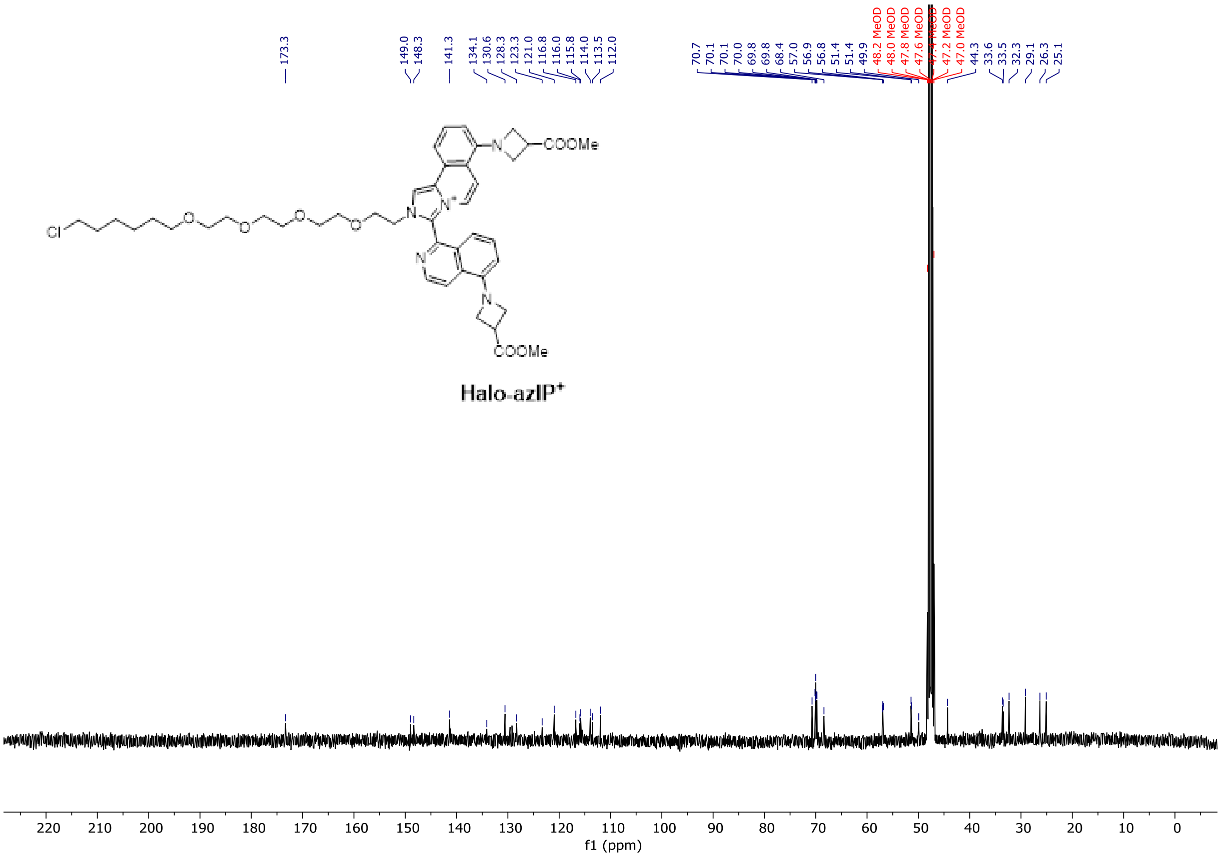

DOPE-IP+

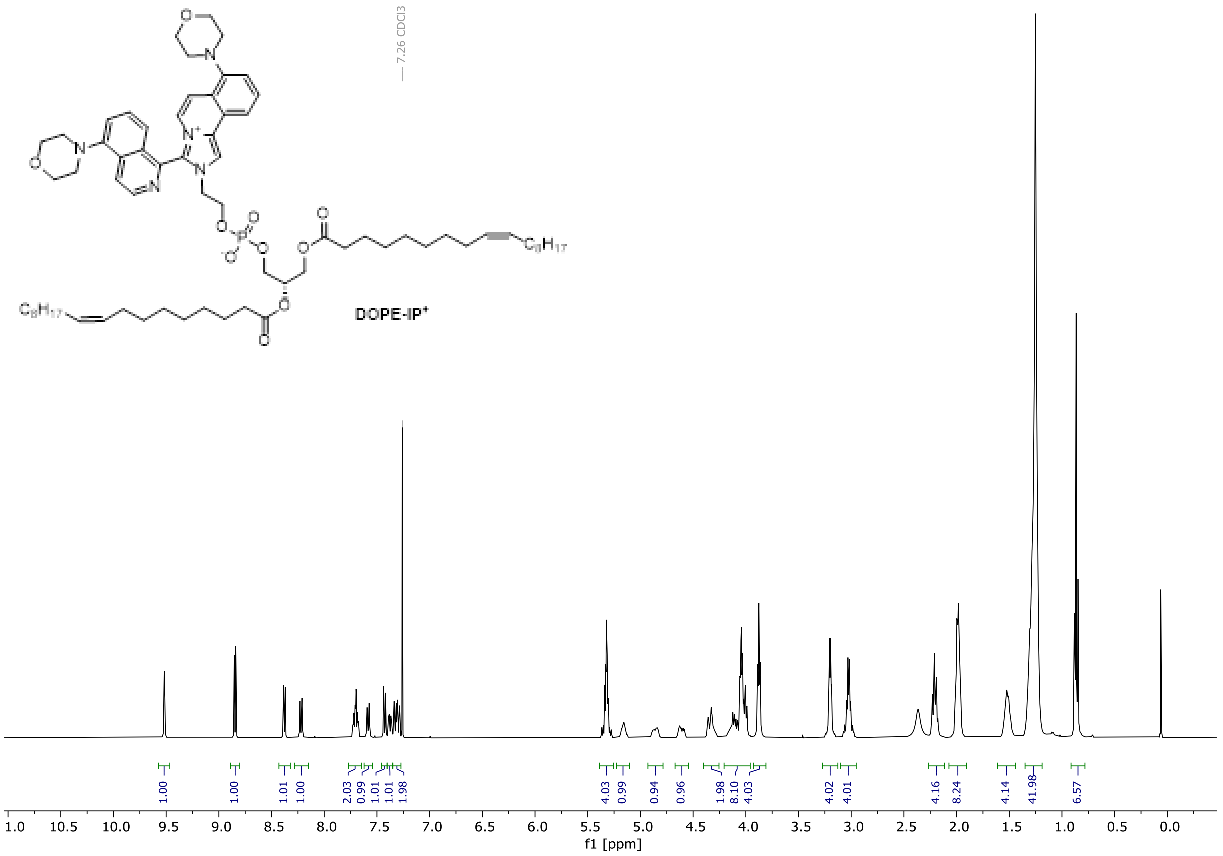

DOPE-IP+
7.26 CDCl3
1.00
1.00
1.01
1.00
2.03
0.99
1.01
1.01
1.98
4.03
0.99
0.94
0.96
1.98
8.10
4.03
4.02
4.01
4.16
8.24
4.14
41.98
6.57
f1 [ppm]

11. Peptide Analysis: HPLC Traces and MS Spectra

Fmoc-GSG-ALFA-OH

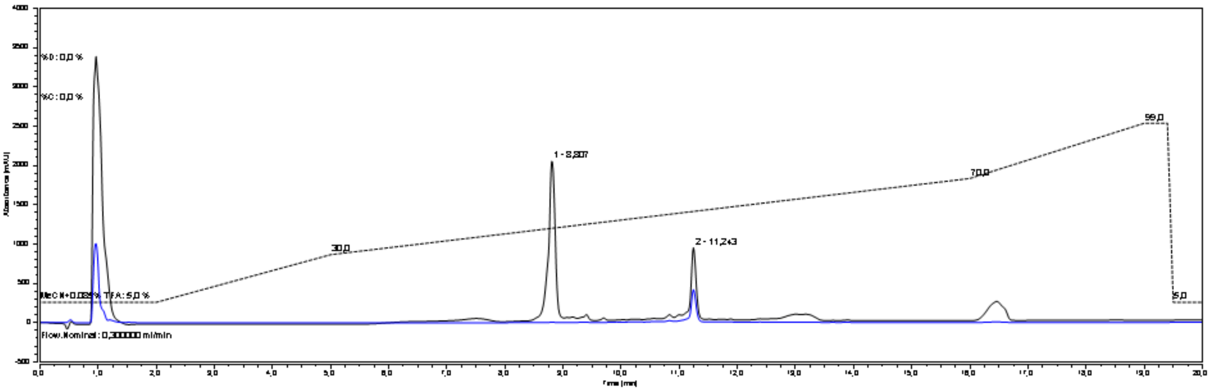

Figure S57. UHPLC trace of Fmoc-GSG-ALFA-OH (Rt = 8.807 min in 30-70 MeCN (+0.1% TFA) in MilliQ (+0.1% TFA) over 20 min). Overlapped spectra of absorbance at 215 nm (black) and 254 nm (blue). The second peak that also absorbs at 254 nm is the Fmoc protected ALFA. Both species are confirmed by MS.

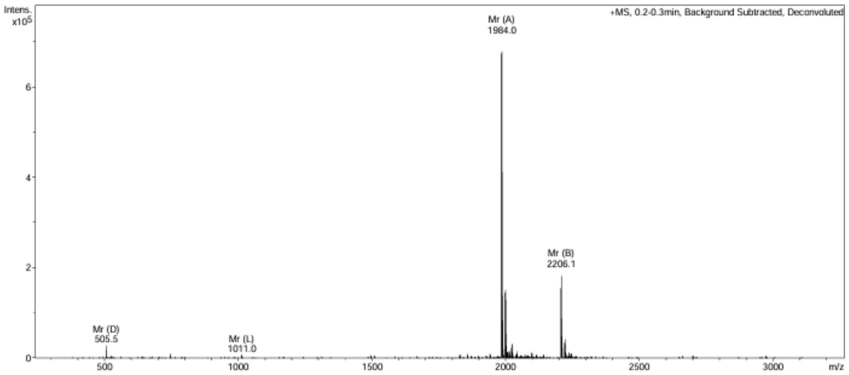

Figure S58. Full deconvoluted ESI(+)-MS spectrum of Fmoc-GSG-ALFA-OH peptide. Mr A corresponds to the Fmoc-deprotected peptide. Mr B correspond to the Fmoc-protected peptide.

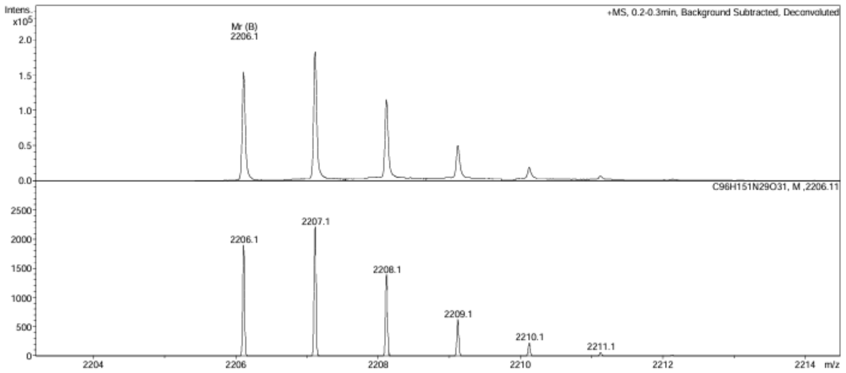

Figure S59. Isotope distribution of Fmoc-GSG-ALFA-OH peptide $(M+H)^+$ in ESI(+)-MS.

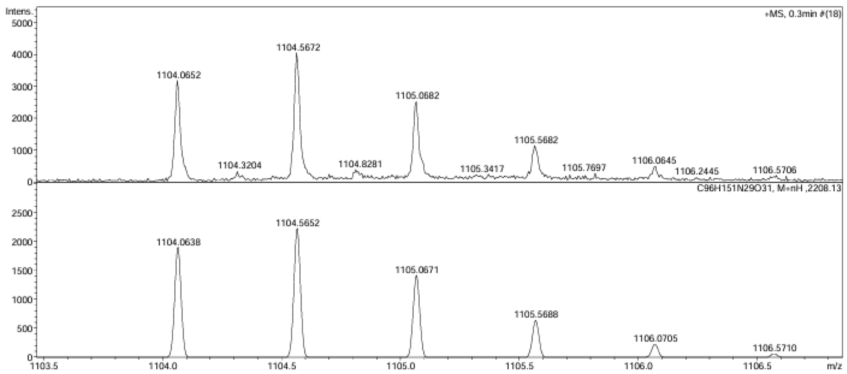

Figure S60. Isotope distribution of Fmoc-GSG-ALFA-OH peptide $(M+2H)^{+2}$ in ESI(+)-MS.

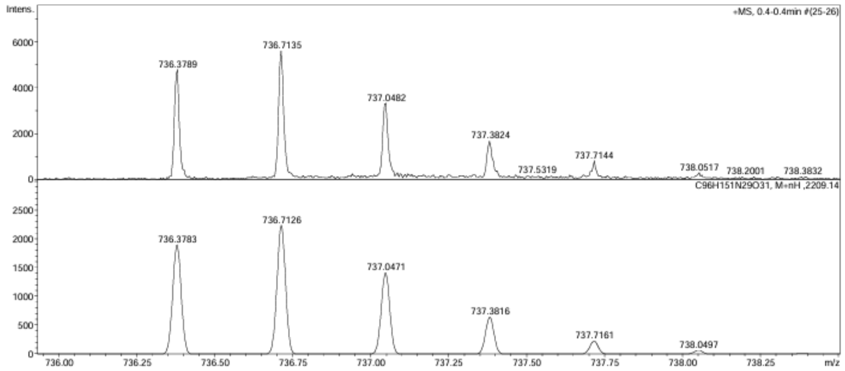

Figure S61. Isotope distribution of Fmoc-GSG-ALFA-OH peptide $(M+3H)^{+3}$ in ESI(+)-MS.

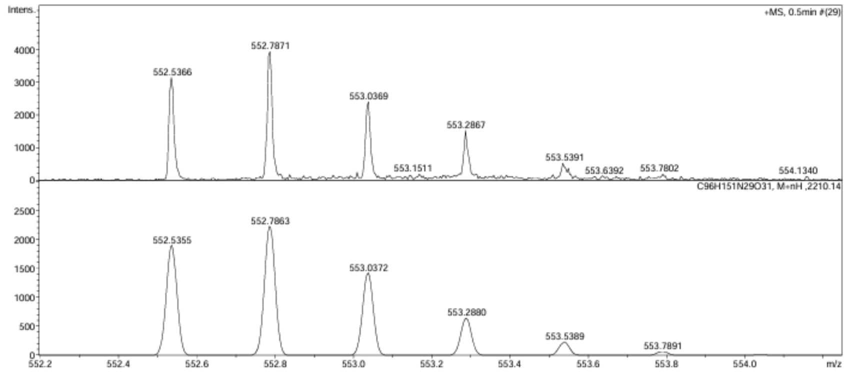

Figure S62. Isotope distribution of Fmoc-GSG-ALFA-OH peptide $(M+4H)^{+4}$ in ESI(+)-MS.

ALFA-IP⁺

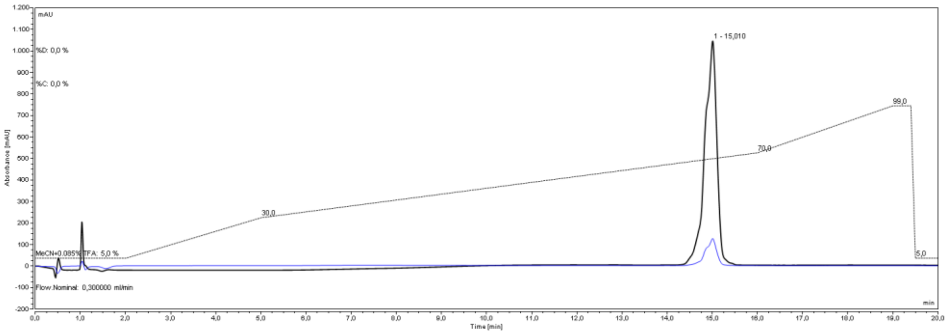

Figure S63. UHPLC trace of F-ALFA-OH (Rt = 15.010 min in 30-70 MeCN (+0.1% TFA) in MilliQ (+0.1% TFA) over 11 min). Overlapped spectra of absorbance at 220 nm (black) and 365 nm (blue).

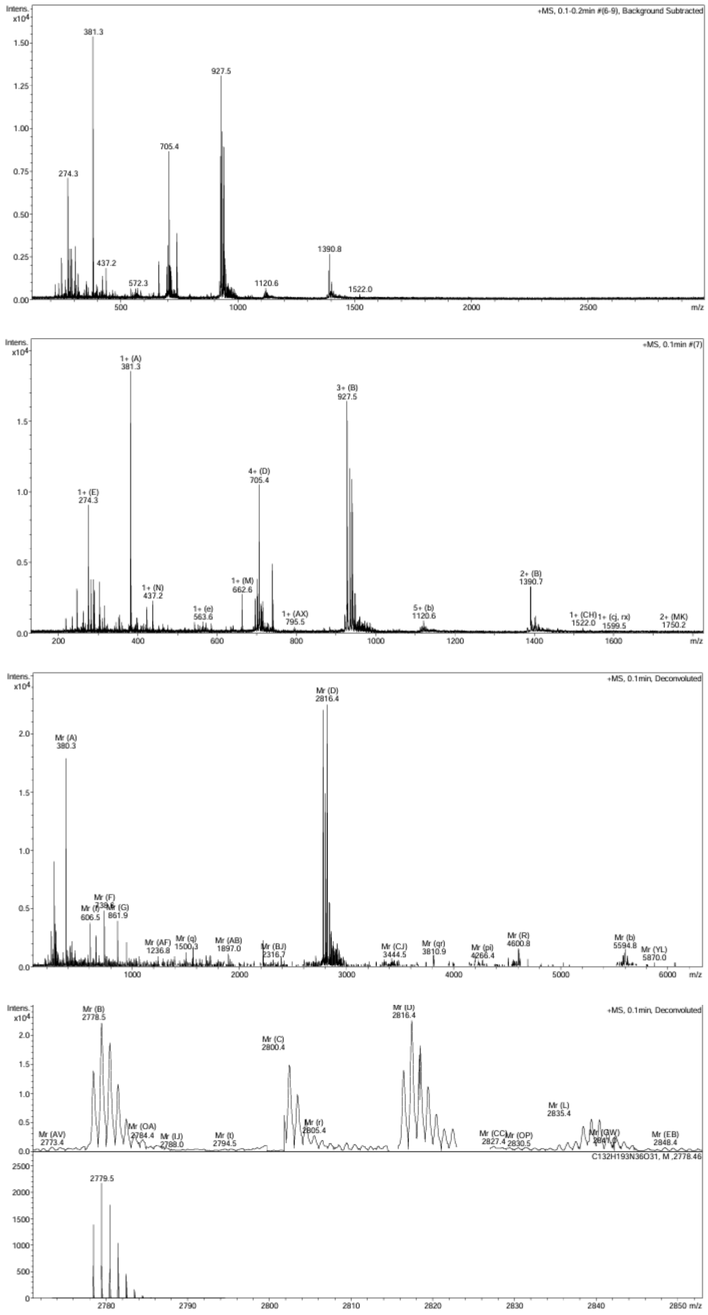

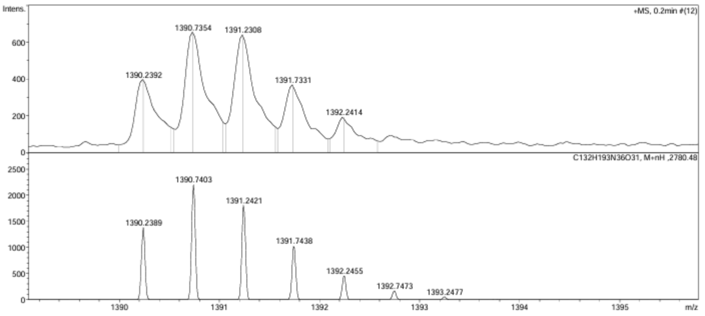
Intens.
+MS, 0.2min #(12)
1390.2392
1390.7354
1391.2308
1391.7331
1392.2414
C132H193N36O31, M+nH ,2780.48
1390.2389
1390.7403
1391.2421
1391.7438
1392.2455
1392.7473
1393.2477
m/z

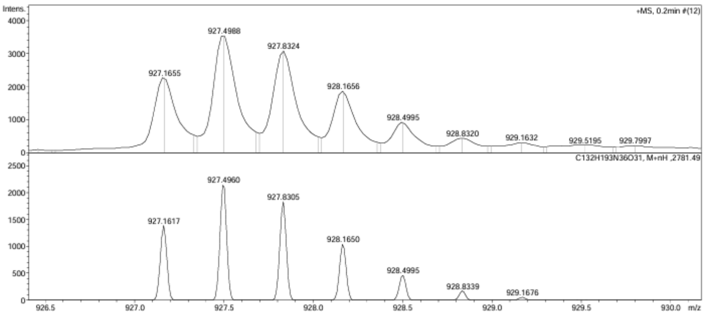
Intens.
+MS, 0.2min #(12)
927.1655
927.4988
927.8324
928.1656
928.4995
928.8320
929.1632
929.5195
929.7997
C132H193N36O31, M+nH ,2781.49
927.1617
927.4960
927.8305
928.1650
928.4995
928.8339
929.1676
m/z

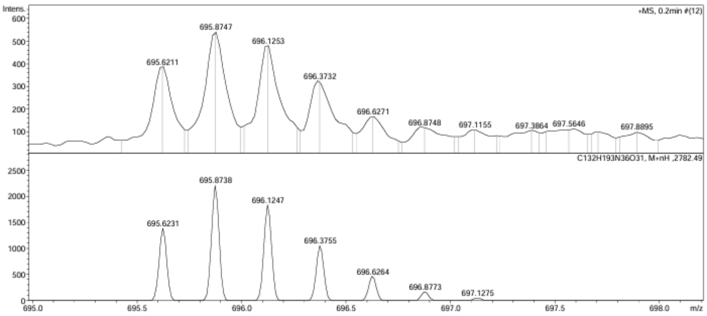
Intens.
+MS, 0.2min #(12)
695.6211
695.8747
696.1253
696.3732
696.6271
696.8748
697.1155
697.3864
697.5646
697.8895
C132H193N36O31, M+nH ,2782.49
695.6231
695.8738
696.1247
696.3755
696.6264
696.8773
697.1275
m/z

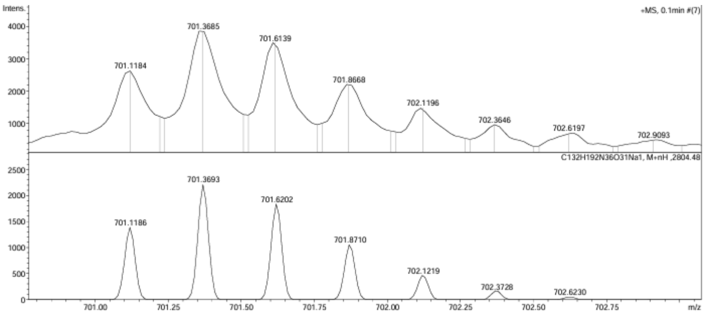
Intens.
+MS, 0.1min #(7)
701.1184
701.3685
701.6139
701.8668
702.1196
702.3646
702.6197
702.9093
C132H192N36O31Na1, M+nH ,2804.48
701.1186
701.3693
701.6202
701.8710
702.1219
702.3728
702.6230
m/z

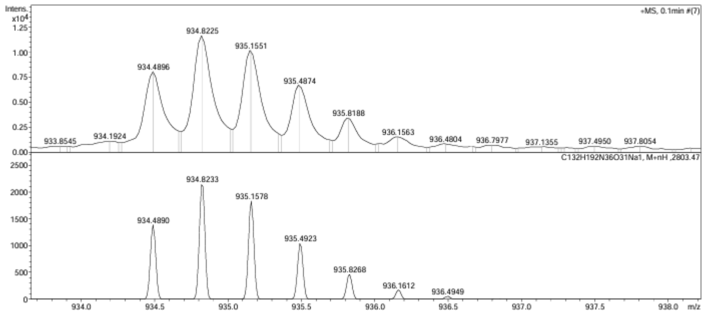
Intens.
x10^4
+MS, 0.1min #(7)
933.8545
934.1924
934.4896
934.8225
935.1551
935.4874
935.8188
936.1563
936.4804
936.7977
937.1355
937.4950
937.8054
C132H192N36O31Na1, M+nH ,2803.47
934.4890
934.8233
935.1578
935.4923
935.8268
936.1612
936.4949
m/z

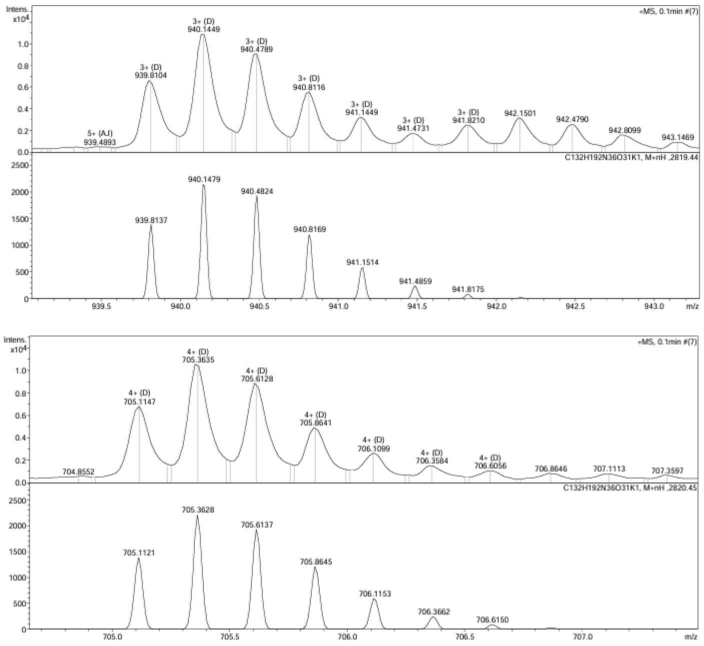

Figure S64. HRMS analysis of ALFA-IP⁺.

CP-IP⁺

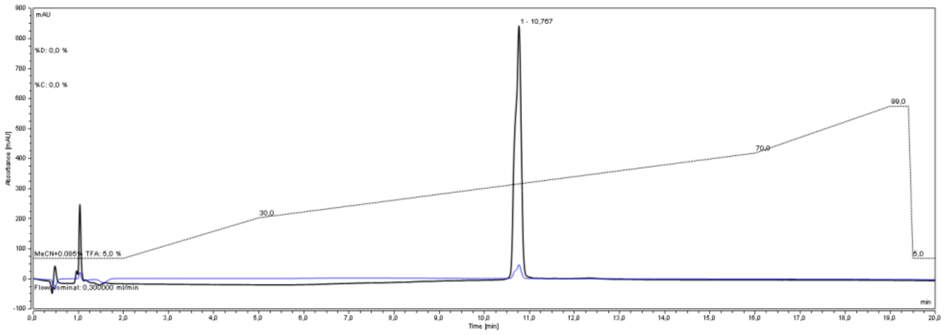

Figure S65. UHPLC trace of FCP (Rt = 10.767 min in 30-70 MeCN (+0.1% TFA) in MilliQ (+0.1% TFA) over 11 min). Overlapped spectra of absorbance at 220 nm (black) and 365 nm (blue).

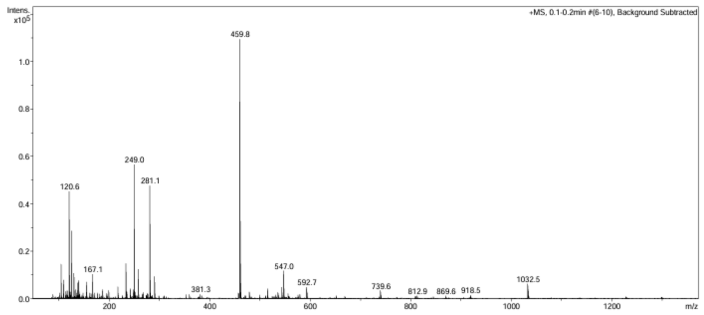

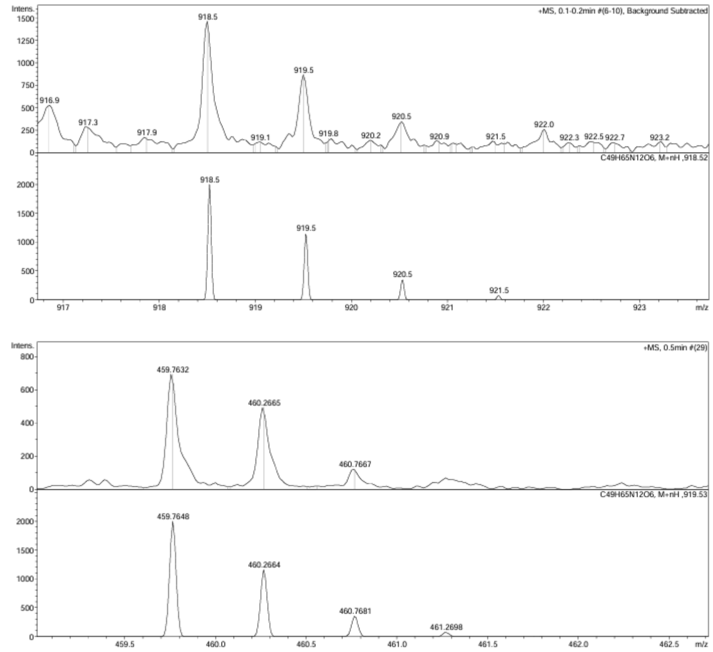

Figure S66. HRMS analysis of CP-IP⁺.

RGD-azIP⁺

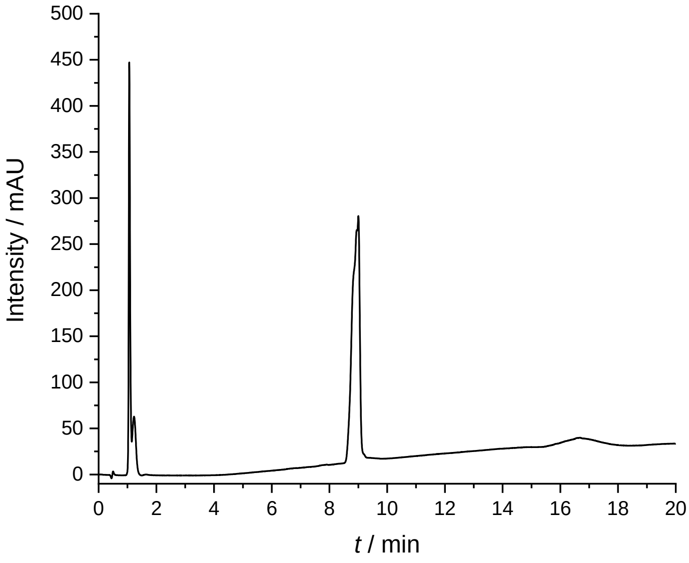

Figure S67. HPLC trace of RGD-azIP⁺.

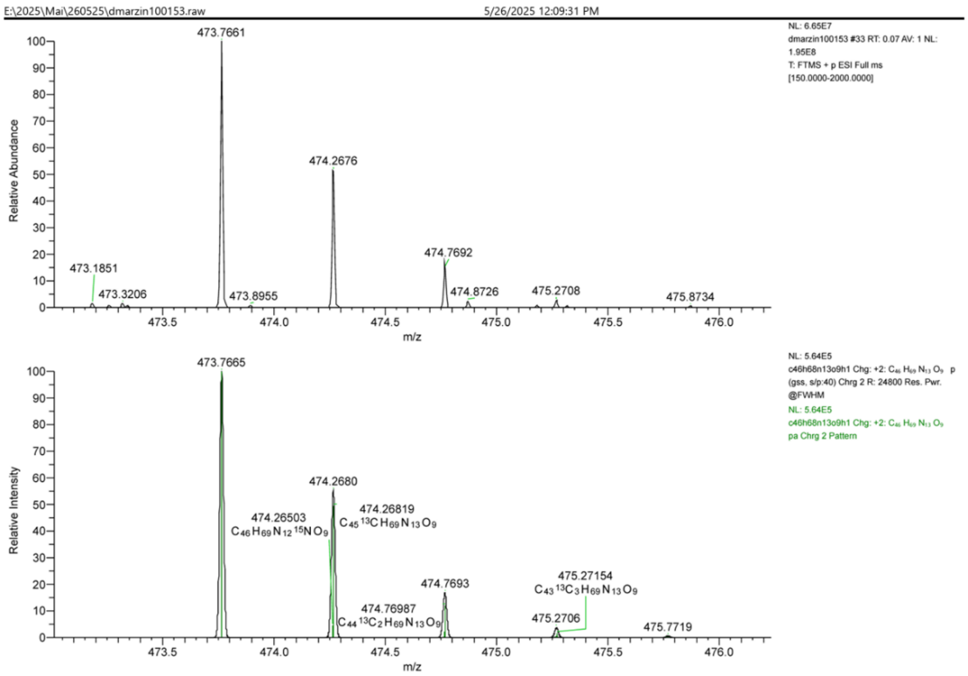

Figure S68. HRMS analysis of RGD-azIP⁺.

12. References

[S1] A. T. Londregan, K. Burford, E. L. Conn, K. D. Hesp, *Org. Lett.* **2014**, *16*, 3336-3339.

[S2] X. Liao, Y. Zhou, C. Ai, C. Ye, G. Chen, Z. Yan, S. Lin, *Tetrahedron Letters* **2021**, *84*, 153457.

[S3] M. Puthanveedu, V. Polychronidou, A. P. Antonchick, *Org. Lett.* **2019**, *21*, 3407-3411.

[S4] J. Jin, D. W. C. MacMillan, *Nature* **2015**, *525*, 87-90.

[S5] N. J. Webb, S. A. Raw, S. P. Marsden, *Tetrahedron* **2018**, *74*, 5200-5205.

[S6] B. Cao, Y. Wang, K. Ding, N. Neamati, Y.-Q. Long, *Org. Biomol. Chem.* **2012**, *10*, 1239-1245.

[S7] B. Li, J. Parker, J. Tong, T. Kodadek, *J. Am. Chem. Soc.* **2024**, *146*, 14633-14644.
